# Lactam Framework Editing via Formal Methylene Deletion

**DOI:** 10.1021/jacs.6c03051

**Published:** 2026-06-30

**Authors:** Nicholas D. D’Arcy-Evans, Gabriele Rossini, Benjamin D. A. Shennan, Darren J. Dixon

**Affiliations:** † Department of Chemistry, 6396 University of Oxford, Oxford, OX1 3TA, United Kingdom

## Abstract

Lactams are cyclic amide building blocks of fundamental importance within synthetic chemistry and drug discovery. Despite their ubiquity, general and convenient methods to interconvert between ring sizes are scarce. Herein, we disclose a new and general strategy enabling the direct dehomologation of lactams, via formal deletion of α-methylene units, streamlining access to a series of valuable medium-to-small-sized lactams from their homologues. This transformation is made possible in a one-pot, two-step sequence, comprising initial amide α-oxidation using an oxoammonium salt, followed by oxidative decarboxylation using readily available *m*-CPBA. The utility of this approach is demonstrated in the ring size scanning of several biologically relevant, drug-like examples, and is extended to the preparation of a diverse range of β-amino acid derivatives, through a net dehomologation-transamidation process from a simple lactam starting material.

## Graphical abstract

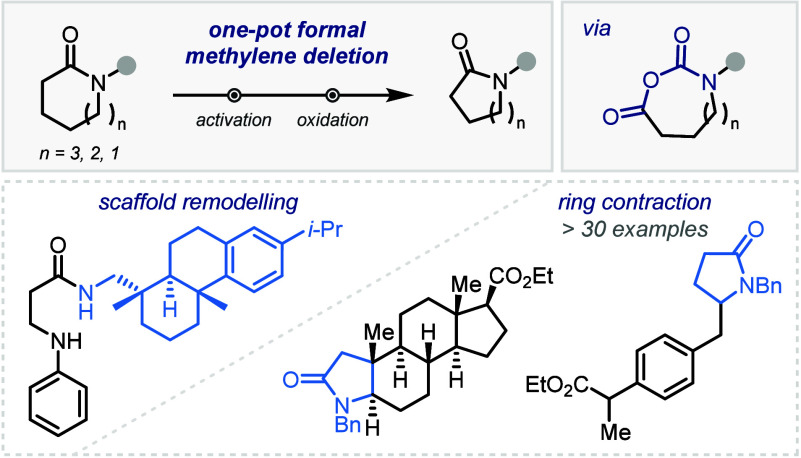

## Introduction

The ability to edit molecular frameworks selectively and precisely is a fundamental goal for contemporary synthetic organic chemistry.^1,2^ While powerful editing strategies have emerged targeting aromatic scaffolds,^3–8^ analogous approaches for ring size perturbation of saturated heterocycles remain comparatively underdeveloped. This limitation is particularly acute for lactams, cyclic amides of fundamental importance, owing to their occurrence as pharmacophores^9^ and utility as precursors for cyclic amines.^10^

Cyclic structures are ubiquitous in bioactive molecules and are essential for imparting three-dimensional structure and function. For example, introduction of a ring into a bioactive fragment is a foundational strategy employed by medicinal chemists to control scaffold flexibility and bias molecular conformation.^11^ Ring systems also boast well-defined exit vectors, allowing pharmacophores to be extended into specific regions in space, thereby enhancing steric and electronic complementarity with a potential binding target.^12,13^ Crucially, both conformational flexibility and the geometrical relationship between bond vectors can be carefully tuned through tailoring of ring size. Therefore, scrupulous examination of these properties through systematic interrogation of differing ring sizes, to the level of a single carbon atom, can unlock valuable new avenues within structure–activity–relationship (SAR) investigations, as demonstrated in several drug candidates (Figure 1B).^14–16^

**Figure 1:**
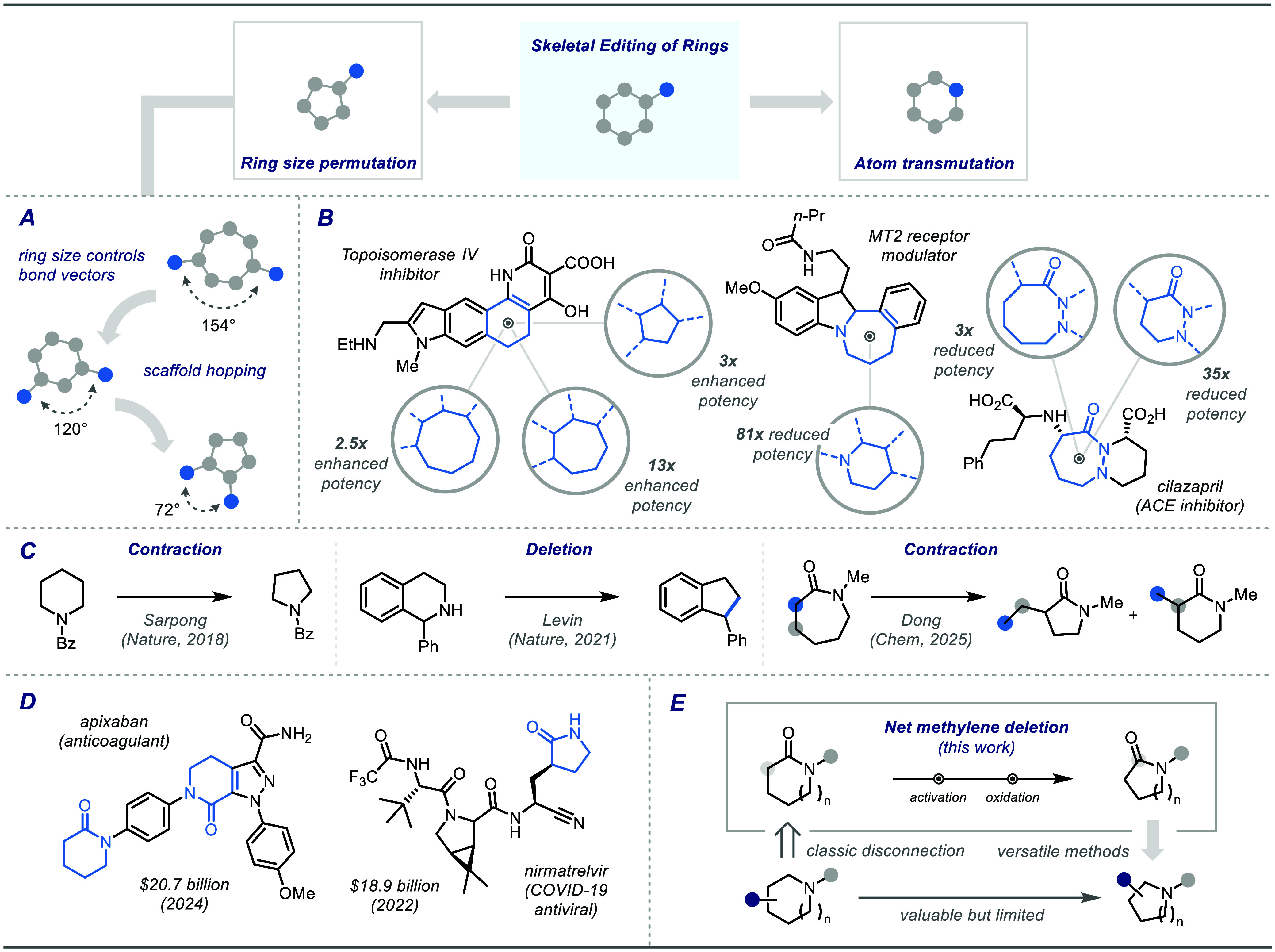
Background. (A) Changes to ring size controls scaffold flexibility and the geometrical relationship between substituents. (B) Systematic interrogation of ring size can reveal leaps in potency. (C) Selected contemporary skeletal editing strategies for ring size modulation. (D) Lactams as privileged motifs in blockbuster pharmaceuticals, including annual sales. (E) This work: one-pot formal methylene deletion from lactams.

Accordingly, to expedite the synthesis of ring size analogues, strategies for ring expansions and contractions have garnered significant attention in recent times (Figure 1C). Elegant methods for ring size modulation of saturated heterocycles have been reported using Ag­(I) salts under oxidative conditions,^17^ and by harnessing hydroxylamines,^18,19^ photochemical activation^20^ and isodiazene formation.^21,22^ Similarly, a plethora of methods for ring expansion of (hetero)­aromatic compounds proceeding through insertions of carbenes, nitrenes and carbon atom equivalents have recently emerged as useful methods for scaffold diversification.^23–29^

Despite the prevalence of lactams in bioactive compounds, available strategies targeting ring size perturbation of these privileged (semi)­saturated heterocycles are exceedingly limited, and classically require harsh, multistep sequences.^30–35^ To this end, Dong et al. recently developed a sophisticated two-stage method for decreasing the ring size of *N*-alkyl lactams (Figure 1C).^36^ Here, installation of an amino pyridine directing group to the amide carbonyl using TiCl_4_ precedes a Rh-catalyzed isomerization step, furnishing contracted α-alkylated lactams, following hydrolysis of the directing group. While impressive, several notable limitations exist, including the inability to tolerate pendant alkynes, aryl bromides, nitroarenes or *N*-aryl lactams. Furthermore, increasing steric bulk or incorporating simple ethers around the ring arrested or significantly impaired reactivity.

Strategically, straightforward methods facilitating general and direct skeletal modification, without additional peripheral alterations, are arguably of the greatest utility for discovery chemists seeking straightforward elucidation of ring size related SAR^2^. A convenient method meeting these requirements would greatly build upon Dong’s inspiring precedent.

Furthermore, lactams themselves serve as an invaluable gateway to α- and β-functionalized saturated cyclic amines – coveted motifs in drugs and natural products. As a result, a myriad of methods exist for the diversification of lactams, including Ni-catalyzed decarbonylation,^35^ 1,2-carbonyl transposition^34^ and a diverse array of reductive functionalization strategies exploiting Tf_2_O-mediated amide activation^37^ and Ir-silane reaction manifolds.^10,38^ In a retrosynthetic sense, ring size modification of these scaffolds at the oxidation level of the lactam, coupled with robust methods for downstream functionalization, offer innumerable possibilities for diversification and exploration of the chemical space surrounding cyclic amines, through strategic employment of lactams as surrogates (Figure 1E).

A mild and expedient approach for direct lactam-to-lactam homologue interconversion would therefore provide chemists with a powerful tool for manipulation of these ubiquitous motifs and their derivatives, with immediate and widespread utility in areas of drug discovery and synthesis.

Herein, we report a strategy for the one-pot, net methylene deletion of a broad array of lactams of differing ring sizes, decoration and *N*-group variation, and demonstrate application on complex, biologically important scaffolds, as well as use in a typical ring size SAR investigation on rolipram. Furthermore, the power of this approach for generating molecular diversity in a modular fashion is showcased through iterative ring contractions and a dehomologation-transamidation transformation, furnishing valuable linear β-amino amides and esters.

### Strategy

In order to meet the requirement for precise and predictable scaffold variation in drug discovery programmes, we opted for a single carbon deletion strategy that could allow for sequential application to a lactam of interest and thus expedite exploration of the associated chemical space.

Inspired by classical decarboxylation reactions as a means to excise a single carbon atom equivalent, we questioned whether the lactam scaffold could be activated in a similar fashion. For example, a 3-fold increase in oxidation state around the amide bond to generate a CO_2_-type synthon, reveals an *N*-carboxyanhydride (NCA) functional group. Discovered by Dieckmann in 1906, adduct formation between an isocyanate and carboxylic acid led to swift amide bond formation with concomitant CO_2_ extrusion, proceeding through an NCA intermediate (Figure 2A).^39,40^

**Figure 2:**
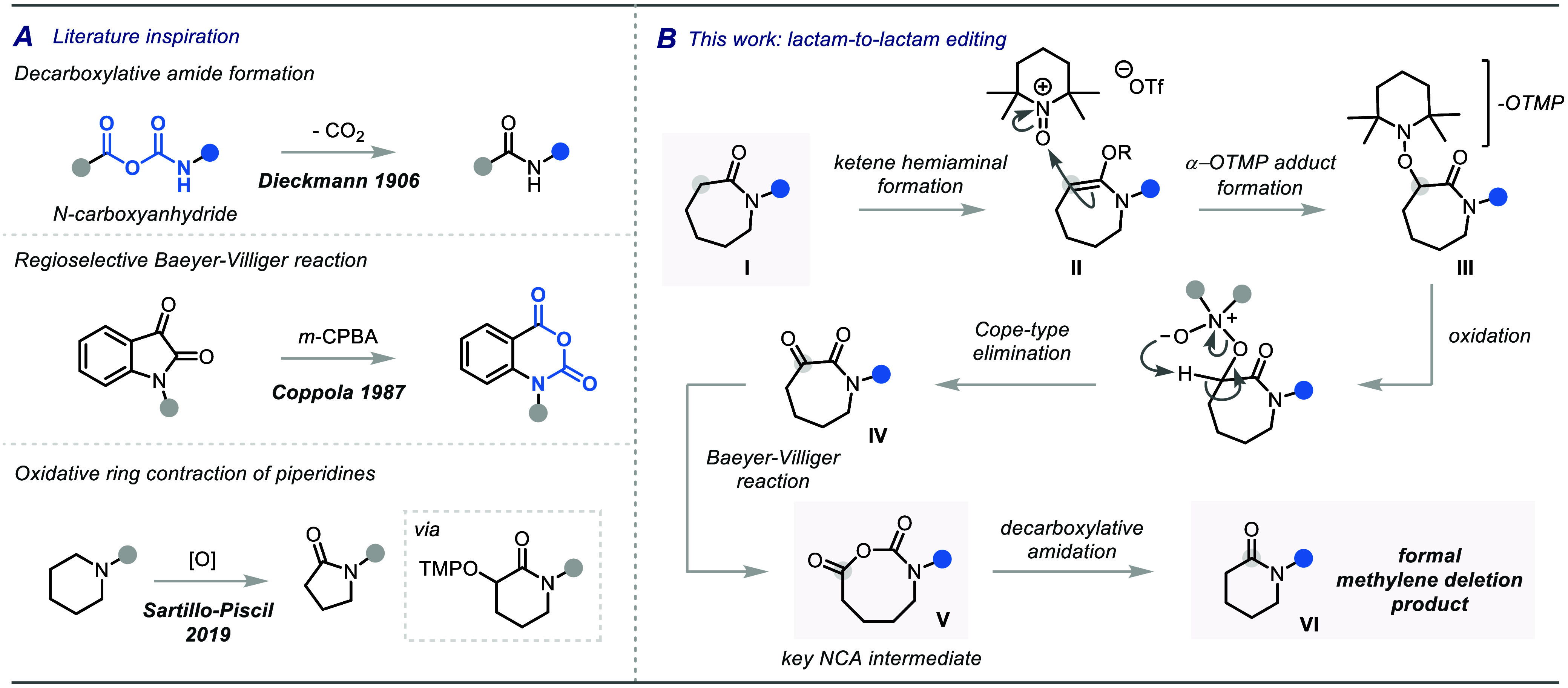
Conceptual outline. (A) Literature inspiration detailing key discovery of *N*-carboxyanhydrides, their generation using peracids, and oxidative ring contraction of piperidines via α-OTMP adducts. (B) Mechanistic hypothesis for the net-methylene deletion from lactams.

Similarly, a transient NCA intermediate was proposed in the decarboxylative cleavage of an imide-derived α-keto benzoylamide, following a regioselective Baeyer–Villiger reaction with peracetic acid.^41^ Preparation of NCAs has also been documented using *m*-CPBA to access isatoic anhydrides (benzo-fused NCAs) from the corresponding isatins,^42^ as well as in the Baeyer-Villiger oxidation of α-keto lactams.^43,44^

Importantly, Sartillo-Piscil et al. applied this reactivity to the preparation of a small scope of *N*-alkyl γ-lactams. In that work, parent *N*-alkyl piperidines were oxidized to α-*O*-2,2,6,6-tetramethylpiperidine (α-OTMP) δ-lactams using aqueous NaOCl, before oxidation by *m*-CPBA to furnish the corresponding γ-lactam products after decarboxylation of the intermediate NCA.^45–48^ While the scope was only demonstrated on alkyl decorated piperidines, notable examples bearing ester and ether functional groups were disclosed. Accordingly, a general synthetic protocol facilitating direct comparison of ring size SAR between two lactams across a broad range of functional groups remains unmet.

At the outset, we recognized that chemoselective activation of the amide in the presence of other reactive functional groups could present a challenging hurdle. However, inspired by pioneering developments from Maulide on the Tf_2_O-mediated formation of linear α-ketoamides using TEMPO,^49^ we questioned whether an alternative, milder approach could be employed to access the intermediate α-OTMP adducts (**III**) as a waypoint to α-ketoamides (**IV**) (Figure 2B). Our proposed approach therefore comprised an oxidative-decarboxylation strategy consisting of the following sequence: selective amide activation and subsequent α-oxidation of the lactam (**I**) to generate an α-ketoamide (**IV**), a regioselective Baeyer–Villiger reaction to access the NCA intermediate (**V**), followed by decarboxylative amidation to furnish the contracted lactam (**VI**) (Figure 2B).

## Results and Discussion

Investigation began with the development of conditions for the selective formation of the α-OTMP-lactam adducts (**III**). We reasoned that these products could be accessed from a nucleophilic ketene hemiaminal (**II**) in the presence of an oxoammonium salt (Figure 2B). Initial attempts focused on direct deprotonation of *N*-benzyl valerolactam with strong bases such as lithium diisopropylamine (LDA), followed by reaction of the anionic intermediate with the oxoammonium salt at cryogenic temperatures, however the results were suboptimal (Table S1).

We then considered whether the use of silyl triflates in the presence of a suitable organic base could offer a more robust approach.^50,51^ Continuing with *N*-benzyl caprolactam as a model substrate, different combinations of solvent, base, temperature and reagent stoichiometry were investigated and two sets of conditions were developed to afford the adduct (**III**) in near-quantitative yield (Table S2). The first set of conditions, utilizing pyridine as a base, was amenable to both secondary and tertiary lactams, but required extended reaction times compared to the second set of conditions employing DBU as the base, with the latter restricted to tertiary lactam substrates. Interestingly, both sets of conditions were tolerant of air and could be performed on moderate scale (2 mmol).

Subsequent oxidation of the α-OTMP adducts (**III**) to the corresponding ketoamide (**IV**) and decarboxylative amidation to **VI** following the Baeyer–Villiger reaction were investigated as a single reaction sequence, since it was anticipated that both steps could be performed with *m*-CPBA (Figure 2B). During the investigation, MeCN was determined to be the optimal solvent and a dual benefit was observed upon addition of Sc­(OTf)_3_. Specifically, in addition to the intended use as a Lewis acidic promoter, Sc­(III) was also found to attenuate side reactions that consumed the *m*-CPBA when pyridine was present in the reaction mixture (*e.g.*, formation of pyridine *N*-oxide) (Table S3).

Unification of the two optimized stages into a convenient one-pot procedure was readily achieved with some minor adjustments, and two sets of conditions were developed (Table S4). Employing the pyridine-based conditions for α-OTMP formation allowed the one-pot reaction to be performed without an inert atmosphere, but required Sc­(OTf)_3_ for efficient conversion in the peracid-mediated oxidation step. Addition of *m*-CPBA as a solution in MeCN at room temperature proved optimal, and furnished the net methylene deletion product **3a** in 65% yield (Table S4). In an analogous one-pot reaction, the DBU-based conditions boosted the yield of **3a** to 85%. However, it was found that the Sc­(III) additive was not necessary, but that it was vital to perform the reaction with rigorous exclusion of air.

### Reaction Scope

The scope of the dehomologation reaction from *N*-substituted lactams under these conditions is presented in Figure 3. Demonstrating utility for iterative application, both δ- and γ-lactam products (**3a**, **3b**) were prepared in good yields from model caprolactam substrate **2a**. High yields were maintained across a range of mono- and disubstituted *N*-benzyl δ-lactam products which included both electron-donating and electron-withdrawing substituents such as *p*-nitrile, *p*-nitro, *m*-methoxy and *m*-trifluoromethyl groups (**3c**–**e**, **3i**, **3j**). Similarly, halogens in *ortho*- and *para*- positions around the ring were tolerated, offering opportunity for downstream functionalization (**3f**–**h**).

**Figure 3:**
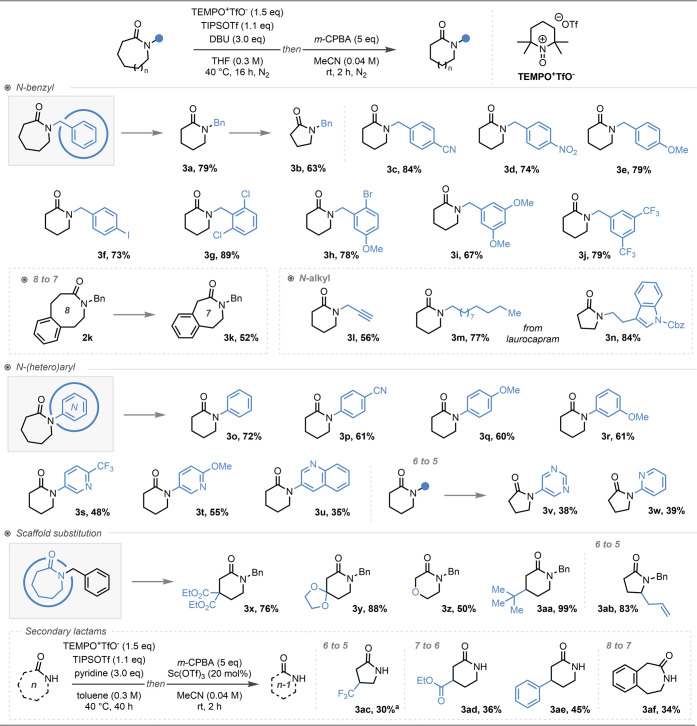
Scope of the lactam dehomologation reaction. *^a^m*-CPBA (3 equiv) added.

Pleasingly, extension to a larger 8-membered lactam was also possible; dehomologation of fused aryl lactam substrate **2k** afforded caprolactam **3k** in good yield. However, application of these conditions to access challenging ring-strained β-lactams was unsuccessful and necessitated development of an alternative synthetic procedure (*vide infra*).

Using this method, *N*-propargyl δ-lactam **3l** was smoothly prepared despite the potential incompatibility with oxidizing *m*-CPBA, and simple *N*-alkyl substitution was also well tolerated (**3m**). Crucially, electron-rich heteroaromatic functionality did not negatively impact the reaction outcome, furnishing γ-butyrolactam **3n** bearing a Cbz-protected indole in very good yield. Notably, the indole carbamate was stable under the reaction conditions, in contrast to *N*-Boc δ-valerolactam (**S1–6**), which was rapidly deprotected in the presence of TIPSOTf.

Importantly, our strategy facilitated smooth editing of *N*-aryl lactams, not possible using Dong or Sartillo-Piscil’s related methodology. Both electron-withdrawing and -donating substituents such as *p*-CN, as well as *p*- and *m*-OMe were well tolerated (**3o**–**r**). As valued motifs in medicinal chemistry, we were also pleased that dehomologation could be performed on lactams containing heteroaromatic rings such pyridine, pyrimidine and quinoline, albeit in slightly lower efficiency (**3s**–**w**).

The optimized conditions were also applied to a variety of lactams decorated with important functional groups. An ε-caprolactam substrate bearing geminal ethyl esters was smoothly converted into δ-valerolactam **3x**. Acid-sensitive groups such as a dioxolane ketal (**3y**), or ether functionality in the lactam skeleton (**3z**) were also well tolerated. Notably, a high degree of steric bulk around the lactam was not detrimental under these conditions as demonstrated by *t*-Bu product **3aa**, presenting the notable advantage of this strategy over related lactam-contraction methodology.^36^ We were pleased to see that a pendant alkene moiety was not oxidized by *m*-CPBA, and instead furnished the contracted allyl γ-butyrolactam **3ab** in very good yield.

However, substrates bearing tertairy amines were not compatible using this methodology. Despite detection of the intermediate α-OTMP adducts using HRMS, the presence of peracid oxidants led to competitive oxidation of the relatively less hindered tertairy amines over the necessary, but less accessible -OTMP group.

Importantly, the pyridine-based one-pot procedure was harnessed for dehomologation of secondary lactams. These alternative conditions were found to be amenable to all of the previously discussed ring sizes, and provided the corresponding contracted homologues in synthetically useful yields (**3ac**–**af**).

### Synthetic Applications

The optimized conditions were effectively demonstrated on complex, biologically relevant scaffolds (Figure 4). Following preparation of a single [7,5]-bicyclic lactam starting material (**4a**), the common scaffold of the lehmizidine alkaloids, iterative application of the optimized conditions allowed facile conversion to the two cores of the indolizidine (**4b**) and pyrrolizidine (**4c**) natural product classes in two simple steps, rather than the multistep synthetic sequences typically required to access these scaffolds individually (Figure 4A).

**Figure 4:**
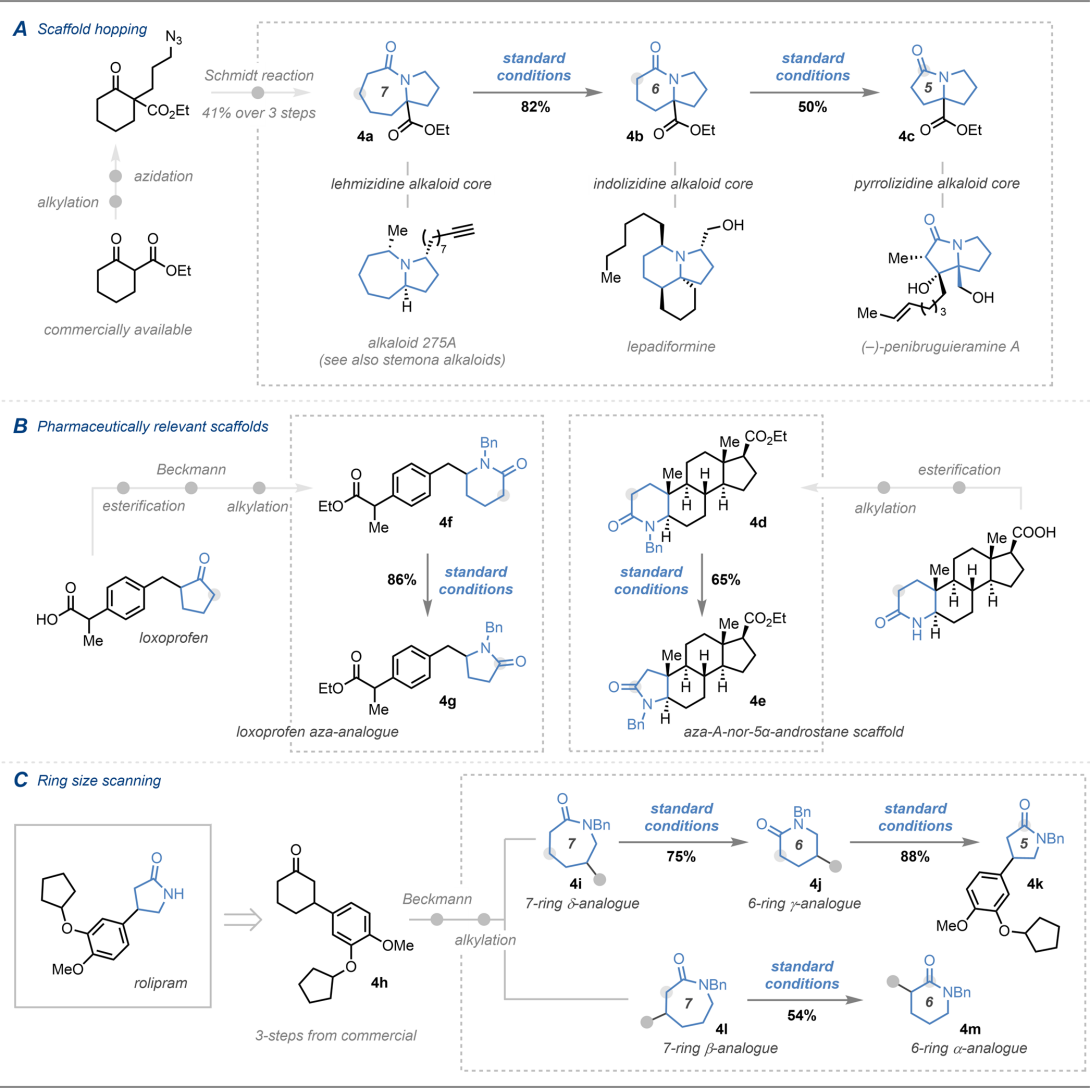
Synthetic applications. (A) Iterative dehomologation of fused bicyclic lactams, hopping between lehmizidine, indolizidine and pyrrolizidine natural product cores. (B) Dehomologation to afford rare *aza*-containing analogues of complex scaffolds. (C) Ring size scanning through iterative dehomologation of rolipram homologues.

Additionally, ring expansion of the nonsteroidal anti-inflammatory drug loxoprofen, via a Beckmann reaction, facilitated production of two novel lactam-containing derivatives (**4f**, **4g**), following dehomologation of the resulting δ-lactam (Figure 4B). Similarly, a rare aza-*A*-Nor-5α-androstane scaffold (**4e**) was obtained in a single step from the corresponding δ-lactam steroid (**4d**).

Further highlighting the potential of this strategy for adoption within medicinal chemistry programmes, we sought to showcase a ring size scanning sequence on analogues of rolipram (Figure 4C). Following a Beckmann reaction on readily available ketone **4h**, the resulting 1:1 mixture of isomeric lactam products were easily separated after benzylation, and subjected to our standard dehomologation conditions, rapidly generating an array of five, ring size analogues containing different substitution patterns, in good to excellent yield (**4i**–**m**). Such an approach, especially when coupled with a regiodivergent step, offers a powerful synthetic tool to survey ring size and bond vector SAR from a single ketone starting material.

The approach outlined here also provides utility beyond atom deletion, and can be leveraged as a means of generating molecular diversity. In what would constitute a formal ‘ring-opening-dehomologation-transamidation’ reaction or more simply a ‘chop and change’ sequence, interception of the intermediate cyclic NCA with an appropriate exogenous amine nucleophile would result in net cleavage of the lactam-amide bond, generating a linear species with one less carbon atom and a new amide linkage. Following this approach, lactam scaffolds may be utilized as building blocks to linear amino acids and their homologues.

Using our method, we found that the easily prepared α-OTMP adducts were excellent substrates for this process. Treatment of α-OTMP γ-lactam **5a** with *m*-CPBA at 40 °C for 1 h, followed by the addition of Et_2_NH, led to 69% of the linear, β-amino amide **5b** by ^1^H NMR analysis (Figure 5A). This procedure was applicable to an array of medicinally relevant primary and secondary amine nucleophiles, including an *N*-Boc-protected amino azetidine, leelamine and *O,O′*-dimethyldopamine, in good to excellent yields (**5e**–**i**) (Figure 5B).

**Figure 5:**
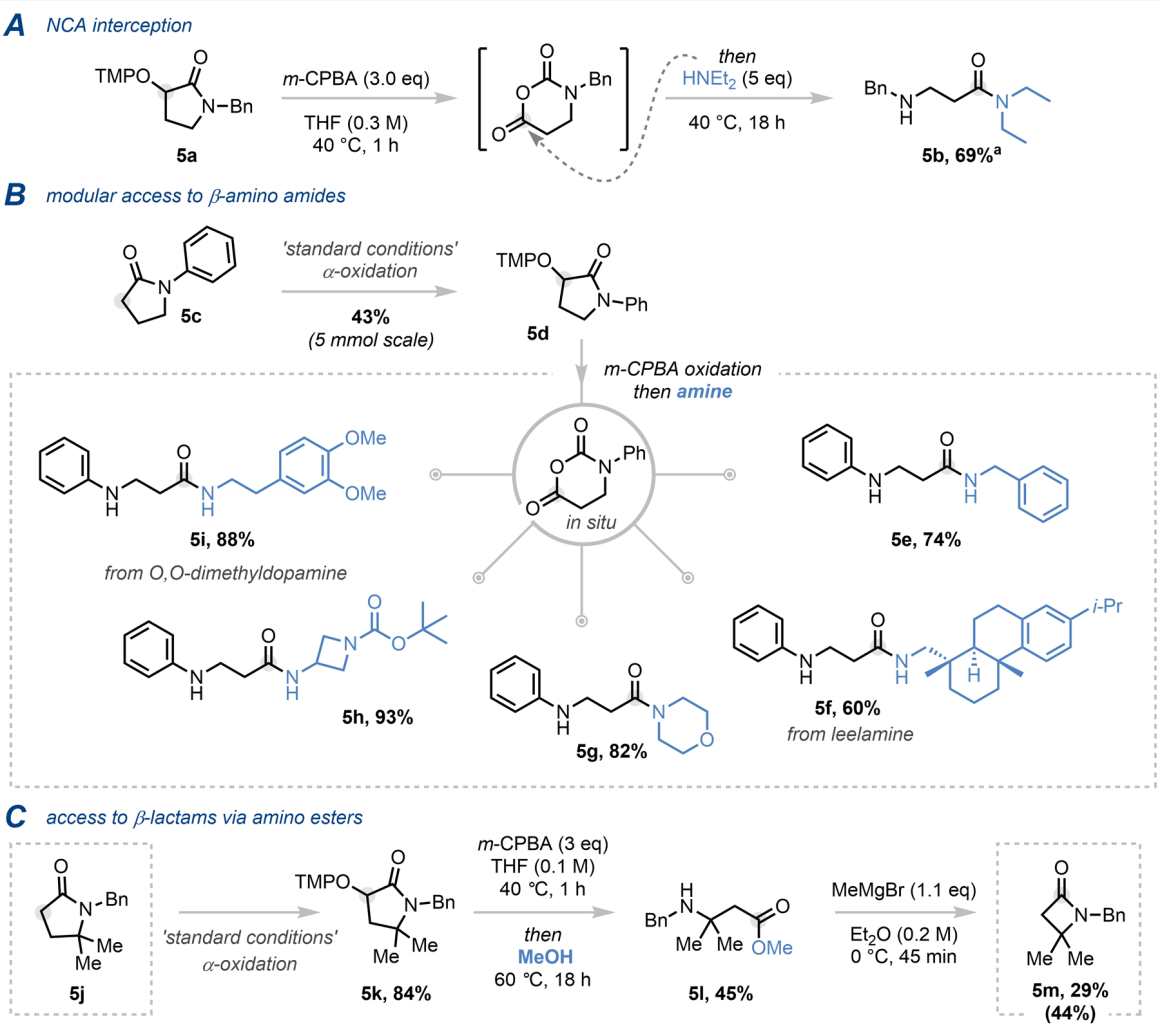
Further synthetic applications. (A) Interception of the *in situ* generated NCA. ^a^yield determined by ^1^H NMR. (B) Modular access to linear β-amino amides through a chop and change sequence originating at the parent γ-lactam. (C) Implementation of a methanolysis strategy to access β-lactams. ^1^H NMR yield shown in parentheses.

Alcohols could also be used as nucleophiles in an analogous chop and change sequence, provided an excess quantity was used. Specifically, addition of MeOH and heating to 60 °C for 18 h, following the peracid oxidation step, provided access to the linear β-amino methyl ester analogue of **5b**. We recognized that this methanolysis procedure could be exploited as a route to access β-lactams from their γ-lactam homologues, circumventing the issues encountered with oligomerization when seeking to access these highly strained small-rings directly under the standard dehomologation conditions. Specifically, the isolated β-amino methyl ester (**5l**), following dehomologation-alcoholysis of the corresponding *gem*-dimethyl γ-lactam (**5j**), was smoothly transformed into the desired β-lactam (**5m**) upon treatment with MeMgBr (Figure 5C).^200^

## Conclusion

Direct ring size alteration of (semi)­saturated heterocycles has remained a challenging aspiration for modern synthetic organic chemistry. The approach described here provides a general and operationally simple solution, permitting direct dehomologation of a range of secondary and tertiary lactams through a decarboxylative strategy. Importantly, in addition to the broad scope and application to pharmaceutically relevant compounds, the *N*-carboxyanhydride intermediate could be harnessed as a valuable means of generating modular molecular diversity, providing a new strategy to repurpose lactams as diverse building blocks.

Beyond its immediate synthetic utility, this work establishes previously ungrounded retrosynthetic logic whereby larger lactam homologues may function as precursors to diversified, ring-contracted cyclic amines, redefining how these cyclic scaffolds can be reconfigured. By enabling facile manipulation of these privileged heterocycles, this work presents a powerful approach for expediting the synthesis and investigation of bioactive molecules and natural products, where control of ring size and scaffold geometry are of crucial importance.

## Supplementary Material



## Notes

Additional optimization data, synthetic protocols and compound characterization data are available in the Supporting Information (PDF)

## ‡

Frick Chemistry Laboratory, Princeton University, Princeton, New Jersey, 08544, United States

## Abbreviations

SAR: structure–activity relationship
NCA: *N*-carboxyanhydride
OTMP: *O*-2,2,6,6-tetramethylpiperidine
TEMPO: 2,2,6,6-tetramethylpiperidine 1-oxyl
LDA: lithium diisopropylamine
DBU: 1,8-diazabicyclo­[5.4.0]­undec-7-ene
TIPSOTf: triisopropylsilyl trifluoromethanesulfonate
NMR: nuclear magnetic resonance
