# Supplementary material for: Lactam Framework Editing via Formal Methylene Deletion
Source: J Am Chem Soc. 2026 Jun 30;148(27):28293–301. doi: 10.1021/jacs.6c03051 (PMC13383639; doi:10.1021/jacs.6c03051)
Supplement: Supplementary file 1 [file ja6c03051_si_001.pdf]

Supporting Information

for

Lactam Framework Editing via Formal Methylene Deletion

Nicholas D. D'Arcy-Evans[1], Gabriele Rossini[1], Benjamin D. A. Shennan[1], Darren J. Dixon*[,1]

[1] Department of Chemistry, Chemistry Research Laboratory, University of Oxford, 12 Mansfield Road, Oxford, OX1 3TA, United Kingdom

Email: darren.dixon@chem.ox.ac.uk

Contents

General Experimental Considerations ... 3
Reaction Optimisation ... 4
Table S1. ... 4
Table S2. ... 5
Table S3. ... 6
Table S4. ... 7
Experimental ... 8
General Procedures ... 8
Reagent Preparation and Purification ... 11
Starting Material Preparation ... 12
Limitations ... 13
Compound Characterisation ... 14
Starting Materials ... 14
Reaction Intermediates (-OTMP adducts and ketoamides) ... 33
Methylene deletion products ... 35
Linear amide products ... 49
NMR Spectra ... 53
Supplementary References ... 145

General Experimental Considerations

Commercially available reagents were purchased from Sigma-Aldrich, Acros Organics, Alfa Aesar, Tokyo Chemical Industry or Fluorochem, and unless stated, were used without purification. Anhydrous solvents were obtained from either Acros Organics and stored under AcroSeal™ over molecular sieves, or from an MBraun SPS800 solvent drying system and stored over 3 Å molecular sieves under a nitrogen atmosphere. Deuterated solvents were used as supplied. All water used was obtained from a Merck Millipore reverse osmosis purification system. NMR spectra were recorded in deuterated solvents (referenced at 7.26 parts per million (ppm) and 77.16 ppm for ^{1}H and ^{13}C NMR, respectively, relative to tetramethyl silane) at room temperature (290–300 K) using a Bruker 400 MHz (^{1}H NMR at 400 MHz and ^{13}C NMR at 101 MHz, unless otherwise specified). Spectra are reported in the order: chemical shift in ppm (multiplicity, coupling constant(s) in Hz, number of nuclei by integration). Multiplicity given as br (broad), s (singlet), d (doublet), t (triplet), q (quartet), quint (quintet), h (hextet), m (multiplet), and combinations of these with '~' used to denote 'resemblance to'. High-resolution mass spectra (ESI) were recorded on an ACQUITY I-Class PLUS UPLC System (Waters, Milford, MA, USA) coupled to an ACQUITY RDa mass spectrometer (Waters, Milford, MA, USA) equipped with an ESI probe, in positive ion mode. Flow rate: 0.300 mL/min using a 50% methanol (aq) + 0.1% formic acid eluent. Scan parameters: analyzer mode, full scan; scan range, 50-2000 m/z; scan rate, 2 Hz; cone voltage, 30 V; capillary voltage, 1.5 kV; desolvation temperature, 550 °C; and intelligent data capture, on. Infrared spectra were taken on a Bruker Tensor 27 FT-IR spectrometer as a thin film. Selected maximum absorbances are reported (in νmax (cm^{-1})). A Leica Galen III Hotstage melting point apparatus and microscope on a Kofler hot block was used to obtain melting point data, and the measurements are reported uncorrected. Analytical thin-layer chromatography (TLC) was performed using Merck silica gel 60 F254 plates and visualized using UV light (254 nm) and basified using aq. $KMnO_4$. Silica gel column chromatography was performed using 60 Å silica gel 40-63 µm purchased from VWR. Isolated yields are given for samples obtained at >90% purity by NMR. Rotary evaporation was performed using Büchi Rotavapor apparatus. Reactions performed under an atmosphere of nitrogen are explicitly stated. Temperatures quoted are external to the reaction mixture. 'r.t refers to room temperature' – approximately 25 °C.

Reaction Optimisation

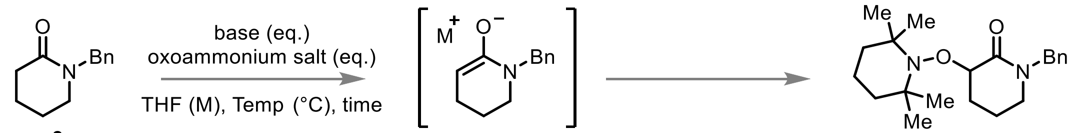

entry	Base (eq)	Oxoammonium salt (eq)	Conc. (M)	Temp. (°C)	Time (h)	Yield (%)	rsm (%)
1[a]	LDA (1.5)	$TEMPO^+BF_4^-$ (1.5)	0.5	-78	2	**31**	20
2	LDA (1.1)	$TEMPO^+BF_4^-$ (1.1)	0.5	-78 to r.t	16	**25**	21
3	LDA (3.0)	$TEMPO^+BF_4^-$ (3.0)	0.5	-78	2	**n.d**	11
4[b]	LDA (1.5)	$TEMPO^+BF_4^-$ (1.5)	0.5	-78	2	**25**	22
5	LDA (1.5)	Bobbitt's salt (1.5)	0.5	-78	2	**20**	30
6	LiHMDS (1.1)	$TEMPO^+BF_4^-$ (1.1)	0.5	-78 to r.t	16	**17**	42
7	KHMDS (1.1)	$TEMPO^+BF_4^-$ (1.1)	0.5	-78 to r.t	16	**8**	68

Table S1.

Reaction optimisation for the formation of -OTMP adducts from *N*-benzyl valerolactam using strong bases. Reactions performed on 0.2 mmol scale. Yield determined by ^{1}H NMR analysis using 1,3,5-trimethoxybenzene or 1,2,4,6-tetramethylbenzene as an internal standard. rsm – residual starting material as determined by ^{1}H NMR. Reactions performed under an atmosphere of N_2. [a]reaction conducted on 0.5 mmol scale; [b]LiCl (6 eq) added to reaction mixture.

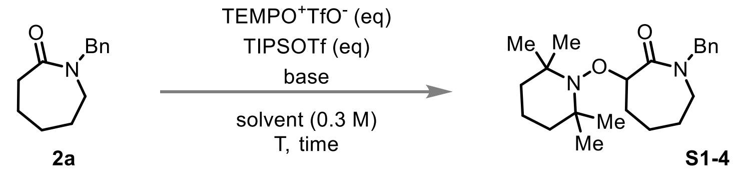

entry	TEMPO+TfO- (eq)	TIPSOTf (eq)	Base (eq)	Solvent	Temp. (°C)	Time (h)	Yield (%)	rsm (%)
1[a]	3	1.3	2,6-lutidine	PhMe	60	16	**10**	88
2	3	1.3	2,6-lutidine	PhMe	60	>48	**12**	88
3	3	1.3	pyridine	PhMe	60	16	**48**	47
4[a]	3	1.3	pyridine	PhMe	40	16	**63**	18
5	1.5	1.1	pyridine	PhMe	40	16	**77**	7
6	1.5	1.1	pyridine	PhMe	40	40	**90**	5
7	1.5	1.1	pyridine	PhMe	r.t	40	**84**	5
8	1.5	1.1	DBU	PhMe	40	16	**68**	14
9	1.5	1.1	DBU	MeCN	40	16	**38**	52
10[a]	1.5	1.1	DBU	THF	40	16	**92**	-
11[b]	1.5	1.1	DBU	THF	40	16	**90**	-
12[a,c]	1.5	1.1	DBU	THF	40	16	**97**	-
additional bases								
13	1.5	1.1	TMG	THF	40	16	**0**	91
14	1.5	1.1	Barton	THF	40	16	**72**	11
15	1.5	1.1	BEMP	THF	40	16	**0**	71
16	1.5	1.1	proton sponge	THF	40	16	**4**	87
17	1.5	1.1	DABCO	THF	40	16	**50**	49
18	1.5	1.1	DIPEA	THF	40	16	**-**	73
19	1.5	1.1	DMAP	THF	40	16	**-**	97
azepan-2-one as substrate								
20[d]	1.5	1.1	pyridine	toluene	40	16	**80**	8
21[d]	1.5	1.1	DBU	THF	40	16	**-**	58

Table S2.

Reaction optimisation for the formation of -OTMP adducts from *N*-benzyl caprolactam using silyl triflates (0.1 mmol scale). Yield determined by ^{1}H NMR analysis using 1,3,5-trimethoxybenzene or 1,2,4,6-tetramethylbenzene as an internal standard. rsm – residual starting material as determined by ^{1}H NMR. [a]reaction performed on 0.3 mmol scale; [b]isolated yield from a 3.5 mmol scale reaction; [c]reaction performed under an atmosphere of N_2; [d]reaction contained additional TEMPO (20 mol%).

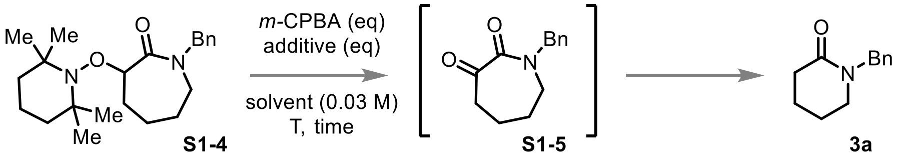

entry	*m*-CPBA (eq)	Solvent	Temp. (°C)	Time (h)	Additive (eq)	Yield **3a** (%)	**S1-5** (%)
1	3	THF	40	2	-	**19**	66
2	3	THF	40	48	-	**14**	74
3	2.1	THF	40	2	-	**6**	68
4	8	THF	40	2	-	**87**	-
5	3	THF	rt	2	-	**27**	59
6	3	THF	40	2	$Sc(OTf)_3$ (10 mol%)	**59**	25
7	3	MeCN	80	2	-	**47**	58
8	3	MeCN	40	2	-	**77**	9
9	3	MeCN	40	2	$Sc(OTf)_3$ (10 mol%)	**81**	8
10	3	MeCN	rt	2	-	**84**	3
11	3	MeCN	rt	2	pyridine (1.5 eq)	**23**	67
12	3	MeCN	rt	2	pyridine (1.5 eq), $Sc(OTf)_3$ (10 mol%)	**79**	14
13[a,b]	3	MeCN	rt	2	DBU (3 eq)	**n.d**	>99
14[a]	3	MeCN	rt	2	-	**>99**	-

Table S3.

Reaction optimisation for the oxidative ring contraction of -OTMP caprolactam adducts to the corresponding contracted δ-lactam, performed on 0.1 mmol scale. brsm – based on residual starting material. Yield determined by ^{1}H NMR analysis with 1,3,5-trimethoxybenzene as an internal standard. [a]reaction performed on 0.3 mmol scale, yield refers to conversion with respect to remaining starting material; [b]reaction performed under an atmosphere of N_2.

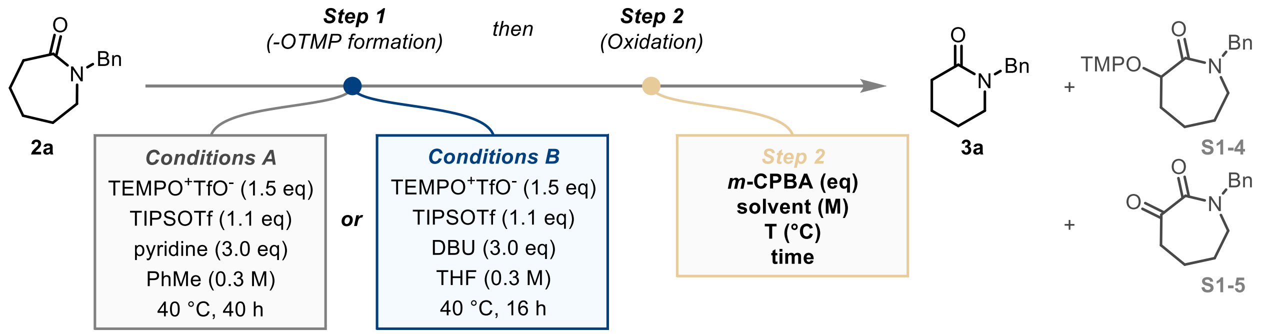

entry	Step 1 (conditions)	mCPBA (eq)	solvent	Conc. (M)	Temp. (°C)	Time (h)	Yield 3a (%)	S1-4 (%)	S1-5 (%)	rsm (%)
1	*A*	5	THF	0.03	40	24	**24**	-	*ca.* 50	9
2	*A*	8	THF	0.03	40	24	**33**	-	*n.d*	5
3	*A*	3	MeCN	0.03	rt	2	**35**	-	44	9
4	*A*	3	MeCN	0.03	rt	24	**37**	-	40	8
5[a]	*A*	3	MeCN	0.03	rt	24	**66**	-	10	6
6[a]	*A*	3	MeCN	0.03	rt	2	**65**	-	n.d	8
7	*B*	3	MeCN	0.03	rt	2	**-**	96	-	-
8[b]	*B*	3	MeCN	0.03	rt	2	**19**	43	39	-
9[a,b]	*B*	3	MeCN	0.03	rt	2	**14**	31	55	-
10[b,c]	*B*	3	MeCN	0.03	rt	2	**45**	-	42	-
11[b,c]	*B*	5	MeCN	0.04	rt	2	**81**	-	-	-

Table S4.

Reaction optimisation for the one-pot methylene deletion from *N*-benzyl caprolactam, reactions performed on a 0.1 mmol scale, yield determined by ^{1}H NMR analysis using 1,3,5-trimethoxybenzene as an internal standard. rsm – residual starting material. n.d – not determined. [a]addition of $Sc(OTf)_3$ (10 mol%) before addition of *m*-CPBA; [b]reaction performed on a 0.3 mmol scale; [c]reaction performed under an atmosphere of N_2.

Experimental

General Procedures

General Procedure A – Lactam benzylation

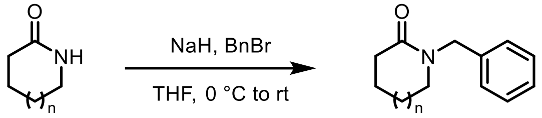

To a flame-dried, three-necked round bottom flask was added NaH (60% in mineral oil, 1.13 eq) and a magnetic stir bar. The flask was evacuated and backfilled with N_2 three times. The NaH was washed with hexane (3 × 20 mL) before THF (0.25 M) was added and the resulting mixture cooled to 0 °C. Separately, the lactam (10 mmol, 1.00 eq) was dissolved in a small amount of THF under an atmosphere of N_2, and added dropwise to the NaH suspension at 0 °C. The mixture was allowed to warm to ambient temperature and stirred for 1.5 h. The alkyl bromide (1.07 eq) was added and the reaction allowed to stir until completion as determined by TLC (18 – 36 h). Water (100 mL) was added carefully and the resulting biphasic mixture was extracted with EtOAc (3 × 100 mL). The combined organic layers were dried over $MgSO_2$, filtered and concentrated *in vacuo*. The crude material was purified using FCC (eluting with 0 – 60% EtOAc in pentane or 0 – 40% acetone in pentane).

General Procedure B – Lactam (hetero)arylation

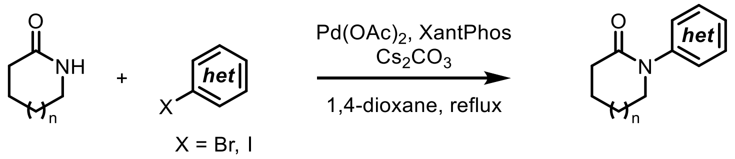

To a flame-dried, three-necked round bottom flask fitted with a condenser was added the lactam (1.3 eq), $Pd(OAc)_2$ (4 mol%), XantPhos (6 mol%), Cs_2CO_3 (1.4 eq) and a magnetic stir bar. The flask was evacuated and backfilled with N_2 three times. Anhydrous 1,4-dioxane (0.25 M) was added and the resulting mixture was stirred at room temperature for 5 min. The aryl halide was added (8.0 mmol, 1.0 eq) before heating the contents at reflux until completion as determined by TLC. The mixture was cooled to ambient temperature and filtered through a pad of celite. The residue was washed with EtOAc (*ca.* 100 mL) and the filtrate concentrated *in vacuo* before purification by FCC (eluting with 0 – 60% EtOAc in pentane or 0 – 40% acetone in pentane).

General Procedure C – Preparation of α-OTMP adducts from lactams

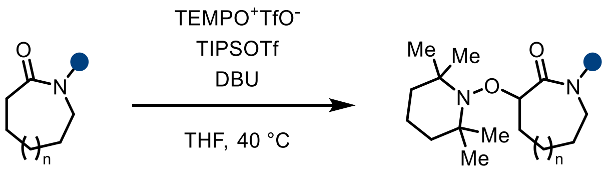

Tertiary Lactams:

A flame-dried, 25 mL round-bottom flask was charged with the lactam starting material (0.3 mmol, 1.0 eq.), THF (0.3 M), $TEMPO^{+}TfO^{-}$ (0.45 mmol, 1.5 eq) and a magnetic stir bar. With stirring, TIPSOTf (0.33 mmol, 1.1 eq) was added, followed by DBU (0.90 mmol, 3.0 eq.) to afford a homogenous solution. The reaction vessel was sealed and heated at 40 °C for 16 hours. Following

cooling to ambient temperature, the reaction mixture was transferred to a separating funnel containing saturated aqueous $NaHCO_3$ and the mixture was extracted with EtOAc (3 × 30 mL). The combined organic layers were dried over $MgSO_4$ before filtration and concentration *in vacuo*. The red/orange oil was then purified by FCC (eluting with 0 – 40% Et_2O in pentane).

Secondary Lactams:
To a flame-dried, 25 mL round-bottom flask was added the lactam starting material (0.3 mmol), toluene (0.3 M), $TEMPO^+TfO^-$ (1.5 eq) and a magnetic stir bar. The contents were then stirred to afford a yellow suspension, to which, TIPSOTf (1.1 eq) and pyridine (3.0 eq) were added. The mixture was heated to 40 °C for 40 h. A solution of saturated aqueous $NaHCO_3$ (30 mL) was added and the mixture extracted with EtOAc (3 × 30 mL). The combined organic layers were dried with $MgSO_4$ before filtration and concentration *in vacuo*. The red/orange oil was then purified by FCC (eluting with 0 – 40% acetone in pentane).

General Procedure D – Formal methylene deletion from tertiary lactams

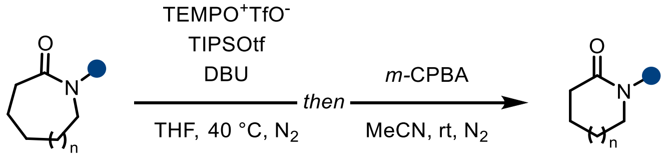

A flame-dried, 25 mL 3-neck round-bottom flask was charged with the oil/liquid lactam (0.30 mmol, 1 eq.) and a magnetic stir bar. The flask was sealed using a glass stopper, rubber septum and a swan neck connected to a Schlenk line. The flask was evacuated and backfilled with nitrogen three times. Dry, degassed THF (0.3 M) was added and the solution stirred until the lactam dissolved. Under a strong flow of nitrogen, $TEMPO^+TfO^-$ (0.45 mmol, 1.5 eq) was added to the flask, followed by TIPSOTf (0.33 mmol, 1.1 eq) and DBU (0.90 mmol, 3.0 eq). The brown/red homogeneous solution was heated to 40 °C for 16 hours.

NOTE: when the lactam starting material was solid, the dried reaction flask was charged with the lactam, $TEMPO^+TfO^-$ *and a magnetic stir bar, before three cycles of evacuation and backfilling with nitrogen gas.*

Separately, a solution of *m*-CPBA (1.5 mmol, 5 eq) in dry MeCN (0.2 M) was prepared under a nitrogen atmosphere and subsequently added to the reaction mixture at ambient temperature over 15 minutes. The resulting orange solution was stirred for a further 2 hours at room temperature. A solution of saturated aqueous $NaHCO_3$ (30 mL) was added and the mixture extracted with EtOAc (3 × 30 mL). The combined organic layers were dried with $MgSO_4$ before filtration and concentration *in vacuo*. The red/orange oil was then purified by FCC (eluting with 0 – 40% acetone in pentane).

General Procedure E – Formal methylene deletion from secondary lactams

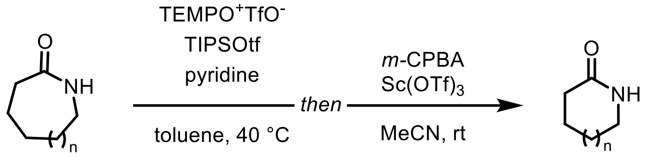

To a flame-dried round bottom flask was added the lactam starting material (0.3 mmol), toluene (0.3 M), $TEMPO^+TfO^-$ (1.5 eq) and a magnetic stir bar. The contents were then stirred to afford a yellow suspension, to which, TIPSOTf (1.1 eq) and pyridine (3.0 eq) were added. The mixture was heated to 40 °C for 40 h. The flask was cooled to room temperature before $Sc(OTf)_3$ (10 mol%) was added, followed by dropwise addition of purified *m*-CPBA (1.5 mmol, 5.0 eq) in MeCN (0.2 M) over 15 min. The resulting orange solution was stirred for 2 hours at room temperature. A solution of saturated aqueous $NaHCO_3$ (30 mL) was added and the mixture extracted with EtOAc (3 × 30 mL). The combined organic layers were dried with $MgSO_4$ before filtration and concentration *in vacuo*. The red/orange oil was then purified by FCC (eluting with 0 – 40% acetone in pentane).

General Procedure F – Dehomologation-transamidation (chop and change)

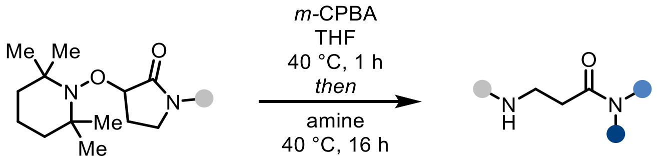

To a flame-dried reaction vessel was added the α-OTMP lactam adduct (0.1 mmol, 1 eq) and a magnetic stir bar. THF (0.3 M) was added and the mixture was stirred to give a homogeneous solution. Purified *m*-CPBA (0.3 mmol, 3 eq) was added as a solid. The vessel was sealed and heated to 40 °C for 1 h. The reaction mixture was briefly lifted from the heat source, before addition of the amine nucleophile (0.5 mmol, 5 eq) and continued heating at 40 °C for 18 h. Following cooling to ambient temperature, added to saturated aqueous $NaHCO_3$ (20 mL) and extracted with EtOAc (3×20 mL). The combined organic phases were dried over Na_2SO_3, filtered and concentrated *in vacuo*. The crude material was then purified by FCC (eluting with 20 – 40% EtOAc in pentane).

Reagent Preparation and Purification

2,2,6,6-tetramethyl-1-oxopiperidin-1-ium trifluoromethanesulfonate ($TEMPO^{+}TfO^{-}$)

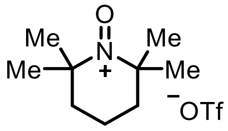

Prepared following a modified literature procedure [1] in which 2,2,2-trifluoromethane sulfonic acid (3.39 mL, 38.4 mmol, 1.2 eq) was added dropwise to a solution of TEMPO (5.0 g, 32 mmol, 1.0 eq) in Et_2O (128 mL, 0.25 M) at 0 °C. After stirring for 30 mins, a yellow precipitate had formed and NaOCl (≥ 8% active chlorine, 14.8 g, 0.5 eq) solution was added dropwise over 1 h. The mixture was allowed to stir at 0 °C for an additional 1 h after which the precipitate was filtered and washed with successive ice-cold solutions of aq. $NaHCO_3$ (5 % w/v), H_2O (100 mL) and Et_2O (200 mL). The yellow residue was left to dry under vacuum for 1 h before recrystallisation from the minimum volume of H_2O (24 mL) to yield the desired compound as fine, yellow crystals (4.67 g, 48%).

mp 119 – 122 °C
HRMS (ESI+) exact mass calculated for $[2M - OTf]^{+}$ ($C_{19}H_{36}F_3N_2O_5S$) requires m/z 461.2292, found m/z 461.2292.
HRMS data is in accordance with the literature [1].

Note: NaOTf can be added to help induce crystallisation. Similarly, best results were observed when using fresh NaOCl solution.

***m*-Chloroperbenzoic acid**

Following a previous literature report [2], *m*-CPBA was dissolved in Et_2O (1.4 M) and washed three times using a pH 7.5 buffer. The organic layer was collected, dried over Na_2SO_4 before filtration and concentration *in vacuo*. The purified *m*-CPBA was then dried on high vacuum for 18 h.

Caution: pure m-CPBA is shock sensitive and can explode without warning. Care must be taken when handling, and handling of large batches should be avoided.

Starting Material Preparation

The following known compounds were prepared according to our previous literature reports [3–6].

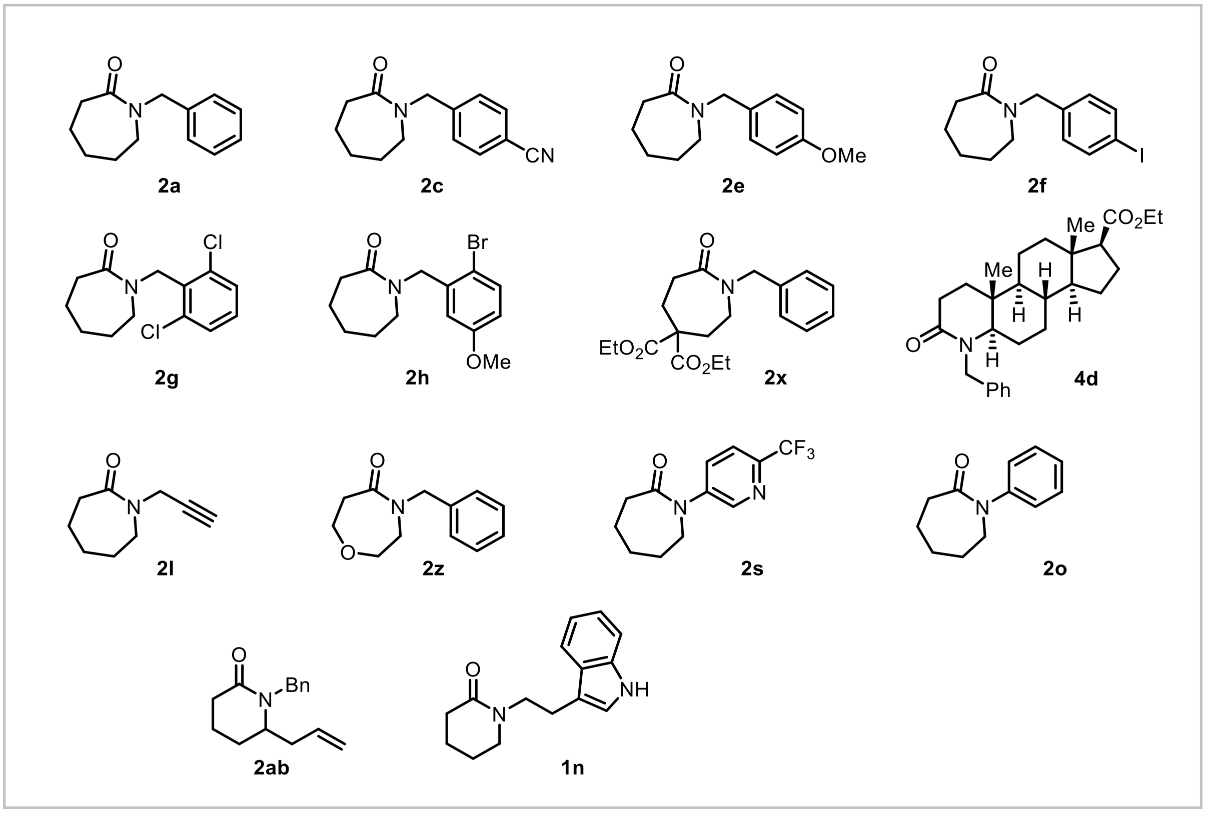

Limitations

Substrate	Comment	Substrate	Comment
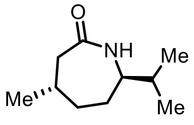	- challenging amide activation, leading to poor α-OTMP adduct formation	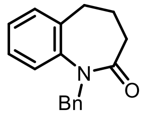	- challenging amide activation, leading to poor α-OTMP adduct formation
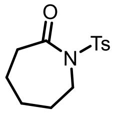	- challenging amide activation due to strongly electron-withdrawing Ts group	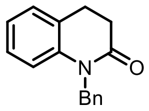	- elimination from the α-OTMP adduct under the reaction conditions led to formation of N-Bn 2-quinolone
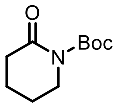	- Boc deprotection observed in the presence of TIPSOTf	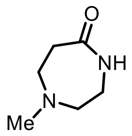	- 3° amine outcompetes TEMPO nitrogen during oxidation, leading to N-oxide α-OTMP adduct as the major species as detected by MS.
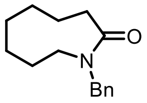	- reaction stalls at ketoamide	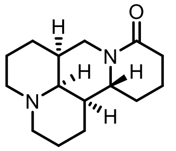	- presence of 3° amine leads to competing N-oxidation (see above)
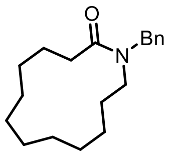	- challenging amide activation, leading to poor α-OTMP adduct formation	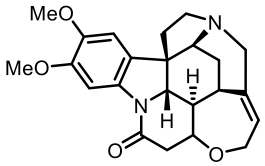	- similar to above

Compound Characterisation

Starting Materials

1-(4-nitrobenzyl)azepan-2-one (2d)

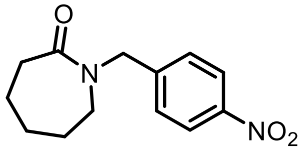

Prepared from ε-caprolactam (10 mmol) and *p*-nitrobenzyl bromide (10.07 mmol) according to General Procedure A to give the desired product as a yellow/red solid (305 mg, 12%).

mp 109 – 110 °C.
^{1}H NMR ($CDCl_3$, 400 MHz) δ 8.17 (d, *J* = 8.6 Hz, 2H), 7.42 (d, *J* = 8.7 Hz, 2H), 4.67 (s, 2H), 3.37 – 3.29 (m, 2H), 2.68 – 2.59 (m, 2H), 1.78 – 1.68 (m, 4H), 1.59 – 1.51 (m, 2H).
^{13}C NMR ($CDCl_3$, 101 MHz) δ 176.3, 147.4, 145.8, 128.8, 123.9, 51.1, 49.7, 37.1, 30.0, 28.4, 23.5.
IR (film) ν_{max}/cm^{-1} 2934, 1647, 1520, 1346.
HRMS (ESI+) exact mass calculated for $[M+H]^+$ ($C_{13}H_{17}N_2O_3$) requires m/z 249.1234, found m/z 249.1235.

1-(3,5-dimethoxybenzyl)azepan-2-one (2i)

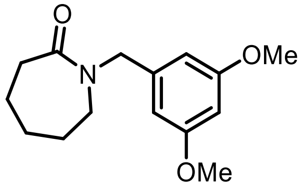

Prepared from ε-caprolactam (10 mmol) and 1-(bromomethyl)-3,5-dimethoxybenzene (10.7 mmol) according to General Procedure A to give the desired product as a red oil (963 mg, 45%).

^{1}H NMR ($CDCl_3$, 400 MHz) δ 6.39 (d, *J* = 2.2 Hz, 2H), 6.34 (t, *J* = 2.3 Hz, 1H), 4.50 (s, 2H), 3.75 (s, 6H), 3.33 – 3.22 (m, 2H), 2.67 – 2.54 (m, 2H), 1.74 – 1.63 (m, 4H), 1.56 – 1.44 (m, 2H).
^{13}C NMR ($CDCl_3$, 101 MHz) δ 176.1, 161.0, 140.4, 106.2, 99.3, 55.4, 51.2, 49.0, 37.2, 30.1, 28.2, 23.6.
IR (film) ν_{max}/cm^{-1} 2934, 1640, 1609, 1597, 1459.
HRMS (ESI+) exact mass calculated for $[M+K]^+$ ($C_{15}H_{21}NO_3K$) requires m/z 302.1153, found m/z 302.1167.

1-(3,5-bis(trifluoromethyl)benzyl)azepan-2-one (2j)

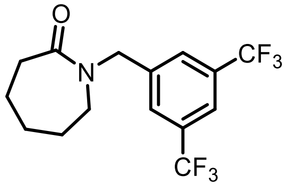

Prepared from ε-caprolactam (10 mmol) and 1-(bromomethyl)-3,5-bis(trifluoromethyl)benzene (10.7 mmol) according to General Procedure A to give the desired product as a colourless oil (3.05 g, 90%).

^{1}H NMR ($CDCl_3$, 400 MHz) δ 7.77 (s, 1H), 7.69 (s, 2H), 4.68 (s, 2H), 3.37 – 3.29 (m, 2H), 2.68 – 2.59 (m, 2H), 1.72 (m, 4H), 1.59 – 1.50 (m, 2H).
^{13}C NMR ($CDCl_3$, 101 MHz) δ 176.4, 141.0, 132.0 (q, *J* = 33.4 Hz), 128.1, 123.4 (q, *J* = 272.8 Hz), 121.5 (p, *J* = 3.8 Hz), 50.9, 49.6, 37.1, 30.0, 28.3, 23.5.
^{19}F NMR ($CDCl_3$, 377 MHz) δ -62.9.
IR (film) ν_{max}/cm^{-1} 2937, 1647, 1208.
HRMS (ESI+) exact mass calculated for $[M+H]^+$ ($C_{15}H_{16}F_6NO$) requires m/z 340.1131, found m/z 340.1134.

2,3,5,6-tetrahydrobenzo[*d*]azocin-4(1*H*)-one (1k)

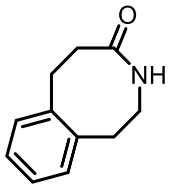

Prepared following modified literature procedures [7,8], 5,6,8,9-tetrahydro-7*H*-benzo[7]annulen-7-one (5 mmol, 1 eq) and hydroxylamine.HCl (7.5 mmol, 1.5 eq) were dissolved in a solution of EtOH:H_2O (5:1, 0.16 M). Sodium acetate (7.5 mmol, 1.5 eq) was added and the resulting mixture was heated to reflux with stirring for 18 h. Following cooling to ambient temperature, the volatiles were removed under reduced pressure. The concentrated crude was diluted with EtOAc and washed with pH 7.5 buffer. The organic phase was collected, dried over Na_2SO_4 and concentrated *in vacuo* to give a white solid which was used in the next step without further purification.

To a flame-dried round bottom flask was added the oxime (5 mmol,1 eq), 1,1,1,3,3,3-hexafluoroisopropanol (20 mL, 0.25 M) and hexachlorophosphazene (5 mol%). The resulting mixture was heated to 70 °C for 16 h. The crude mixture was concentrated under reduced pressure before *t*-BuOH (0.5 M), H_2O (25 mmol, 5 eq) and MsOH (12.5 mol%) were added and heated to 80 °C for 2 h. Following cooling to rt, the concentrated crude was purified by FCC (eluting with 0 – 5% MeOH in EtOAc) to afford the lactam product as an off-white solid (370 mg, 42%).

^{1}H NMR (400 MHz, $CDCl_3$) δ 7.22 – 7.11 (m, 3H), 7.11 – 7.02 (m, 1H), 5.85 (s, 1H), 3.61 – 3.46 (m, 2H), 3.16 – 2.98 (m, 4H), 2.77 (t, *J* = 7.1 Hz, 2H).
^{13}C NMR (101 MHz, $CDCl_3$) δ 176.4, 139.2, 137.2, 130.8, 130.3, 127.4, 127.2, 41.5, 36.2, 36.1, 30.4.
Spectral data are consistent with the literature [9].

3-benzyl-2,3,5,6-tetrahydrobenzo[*d*]azocin-4(1*H*)-one (2k)

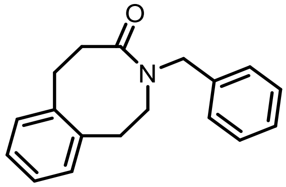

Prepared from **1k** (1.4 mmol) and BnBr (1.5 mmol) following General Procedure A, and purified by FCC, (eluting with 40% EtOAc in pentane) to afford the title compound as a white solid (315 mg, 83%).

^{1}H NMR (400 MHz, $CDCl_3$) δ 7.33 – 7.22 (m, 3H), 7.22 – 7.07 (m, 5H), 6.95 – 6.86 (m, 1H), 4.30 (s, 2H), 3.64 (t, *J* = 6.9 Hz, 2H), 3.08 – 2.91 (m, 6H).
^{13}C NMR (101 MHz, $CDCl_3$) δ 173.0, 138.4, 137.5, 136.2, 130.8, 130.2, 128.6, 128.5, 127.5, 127.4, 127.1, 49.5, 45.5, 37.0, 34.2, 30.5.
IR (film) ν_{max}/cm^{-1} 2936, 1635, 1493, 1429, 1157.
HRMS (ESI+) exact mass calculated for $[M+Na]^+$ ($C_{18}H_{19}NONa$) requires m/z 288.1359, found m/z 288.1371.
mp 143 – 144 °C.

benzyl 3-(2-(2-oxopiperidin-1-yl)ethyl)-1H-indole-1-carboxylate (2n)

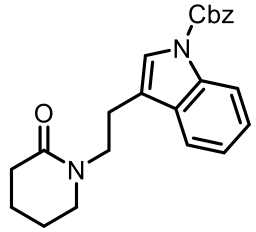

A two-neck round-bottom flask was charged with and stir bar and NaH (60% dispersion in mineral oil) (74 mg, 1.86 mmol, 1.5 eq), before the flask was sealed with a rubber septum and subjected to three cycles of evacuation and backfilling with N_2 gas. The NaH was washed with hexane (2 × 5 mL), before a small amount of THF was added. The mixture was cooled to 0 °C before a solution of 1-(2-(1H-indol-3-yl)ethyl)piperidin-2-one (300 mg, 1.24 mol, 1 eq) was added dropwise at 0 °C with stirring. Note: a few drops of DMF were added to aid solubility. The mixture was stirred at room temperature for 30 minutes, after which, benzyl chloroformate (350 µL, 2.48 mmol, 2.0 eq) was added at 0 °C. The mixture was left to stir at room temperature overnight, quenched with H_2O (50 mL) and the organics extracted with EtOAc (3 × 50 mL). The combined organic layers were dried over Na_2SO_4, filtered, concentrated and purified using FCC (eluting with 100% EtOAc) to afford the product as a thick, yellow oil (396 mg, 84%).

^{1}H NMR ($CDCl_3$, 400 MHz) δ 8.18 (s, 1H), 7.64 (d, *J* = 7.0 Hz, 1H), 7.49 – 7.44 (m, 3H), 7.43 – 7.36 (m, 3H), 7.32 (t, *J* = 7.0 Hz, 1H), 7.26 (t, *J* = 6.8 Hz, 1H), 5.42 (s, 2H), 3.60 (t, *J* = 7.6 Hz, 2H), 3.14 (t, *J* = 5.7 Hz, 2H), 2.99 – 2.89 (m ~ t, 2H), 2.37 (t, *J* = 6.5 Hz, 2H), 1.75 – 1.60 (m, 4H).
^{13}C NMR ($CDCl_3$, 101 MHz) δ 169.6, 150.6, 135.4, 135.1, 130.5, 128.6, 128.6, 128.3, 124.6, 122.8, 122.5, 119.1, 119.0, 115.1, 68.4, 48.6, 47.7, 32.3, 23.1, 22.7, 21.2.
HRMS (ESI+) exact mass calculated for $[M+H]^+$ ($C_{23}H_{25}N_2O_3$) requires m/z 377.1860, found m/z 377.1871.

4-(2-oxoazepan-1-yl)benzonitrile (2p)

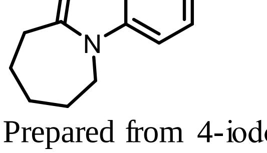

Prepared from 4-iodobenzonitrile (10 mmol) and ε-caprolactam (12 mmol) according to General Procedure B to give the desired product as a brown solid (1.94 g, 91%).

^{1}H NMR ($CDCl_3$, 400 MHz) δ 7.64 (d, *J* = 8.8 Hz, 2H), 7.36 (d, *J* = 8.8 Hz, 2H), 3.86 – 3.74 (m, 2H), 2.79 – 2.65 (m, 2H), 1.89 – 1.81 (m, 6H).

^{13}**C NMR** ($CDCl_3$, 101 MHz) δ 175.5, 148.3, 133.0, 126.7, 118.7, 109.6, 52.6, 37.9, 29.7, 29.1, 23.5.
IR (film) ν_{max}/cm^{-1} 2936, 2227, 1661, 1560, 1506.
HRMS (ESI+) exact mass calculated for $[M+H]^+$ ($C_{13}H_{15}N_2O$) requires m/z 215.1179, found m/z 215.1177.
mp 84 – 86 °C.

1-(4-methoxyphenyl)azepan-2-one (2q)

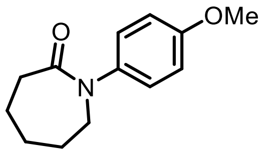

Prepared from 4-bromoanisole (10 mmol) and ε-caprolactam (12 mmol) according to General Procedure B to give the desired product as a yellow oil (608 mg, 28%).

^{1}**H NMR** ($CDCl_3$, 400 MHz) δ 7.11 (d, *J* = 8.9 Hz, 2H), 6.88 (d, *J* = 8.9 Hz, 2H), 3.79 (s, 3H), 3.74 – 3.68 (m, 2H), 2.68 (m, 2H), 1.86 – 1.76 (m, 6H).
^{13}**C NMR** ($CDCl_3$, 101 MHz) δ 175.8, 158.0, 137.7, 127.4, 114.5, 55.5, 53.4, 37.7, 30.0, 28.9, 23.7.
Spectral data are consistent with the literature [10].

1-(3-methoxyphenyl)azepan-2-one (2r)

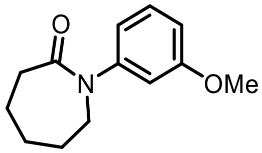

Prepared from 3-bromoanisole (10 mmol) and ε-caprolactam (12 mmol) following General Procedure B to give the desired product as a pale, yellow oil (1.56 g, 71%).

^{1}**H NMR** ($CDCl_3$, 400 MHz) δ 7.27 (td, *J* = 7.9, 0.8 Hz, 1H), 6.82 – 6.75 (m, 3H), 3.79 (s, 3H), 3.77 – 3.69 (m, 2H), 2.75 – 2.65 (m, 2H), 1.87 – 1.77 (m, 6H).
^{13}**C NMR** ($CDCl_3$, 101 MHz) δ 175.6, 160.2, 145.8, 129.9, 118.6, 112.4, 112.3, 55.5, 53.2, 37.8, 30.0, 29.0, 23.7.
IR (film) ν_{max}/cm^{-1} 2932, 1660, 1601, 1488.
HRMS (ESI+) exact mass calculated for $[M+Na]^+$ ($C_{13}H_{17}NO_2Na$) requires m/z 242.1152, found m/z 242.1151.

1-(6-methoxypyridin-3-yl)azepan-2-one (2t)

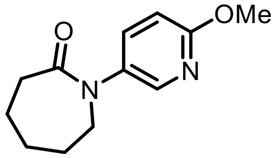

To a flame-dried reaction flask was added, CuI (480 mg, 0.5 eq, 2.5 mmol), K_3PO_4 (2.12 g, 10.0 mmol, 2.0 eq) and ε-caprolactam (570 mg, 5.0 mmol, 1 eq). The vessel was sealed and subjected to three cycles of evacuation and backfilling with argon. Sequentially, *N,N*-dimethylethylenediamine (400 µL, 3.8 mmol, 0.75 eq) and 5-bromo-2-methoxypyridine (970 µL, 7.5 mmol, 1.5 eq) were added. The reaction mixture was heated to 110 °C for 24 h, before cooling

to ambient temperature and filtered through a short pad of Celite, following dilution with CH_2Cl_2. The crude residue was purified using FCC (eluting with 50-80% EtOAc in pentane) to afford the title compound as a brown solid (620 mg, 57%).

^{1}H NMR (400 MHz, $CDCl_3$) δ 7.99 (dd, *J* = 2.7, 0.7 Hz, 1H), 7.43 (dd, *J* = 8.8, 2.7 Hz, 1H), 6.72 (dd, *J* = 8.8, 0.7 Hz, 1H), 3.90 (s, 3H), 3.74 – 3.65 (m, 2H), 2.71 – 2.63 (m, 2H), 1.80 (s, 6H).
^{13}C NMR (101 MHz, $CDCl_3$) δ 176.1, 162.2, 143.9, 137.4, 135.1, 111.0, 53.7, 53.3, 37.5, 29.9, 29.0, 23.6.
IR (film) ν_{max}/cm^{-1} 3456, 2932, 1656, 1606, 1444.
HRMS (ESI+) exact mass calculated for $[M+Na]^+$ ($C_{12}H_{16}N_2O_2Na$) requires m/z 243.1104, found m/z 243.1113.
mp 101 – 102 °C.

1-(quinolin-3-yl)azepan-2-one (2u)

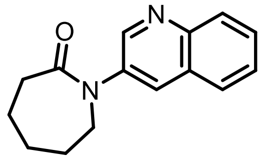

To a solution of ε-caprolactam (200 mg, 1.8 mmol, 1 eq) in 1,4-dioxane was added 3-bromoquinoline (450 mg, 2.2 mmol, 1.2 eq), XantPhos (510 mg, 0.8 mmol, 0.5 eq), $Pd_2(dba)_3$ (160 mg, 0.18 mmol, 0.1 eq) and Cs_2CO_3 (860 mg, 2.7 mmol, 1.5 eq). The reaction vessel was sealed and degassed by bubbling N_2 through the reaction mixture for 20 minutes. The mixture was heated to 120 °C for 6 h. Following cooling to ambient temperature and dilution with brine, the mixture was extracted with EtOAc (3 times) and the combined organic layers dried over Mg_2SO_4 and concentrated *in vacuo*. The resulting residue was purified using FCC (eluting with 0-5% MeOH in DCM) to provide the title compound as a brown solid (301 mg, 71%).

^{1}H NMR (400 MHz, $CDCl_3$) δ 8.82 (d, *J* = 2.5 Hz, 1H), 8.09 (d, *J* = 8.5 Hz, 1H), 7.98 (d, *J* = 2.5 Hz, 1H), 7.78 (dd, *J* = 8.2, 1.4 Hz, 1H), 7.69 (ddd, *J* = 8.4, 6.9, 1.5 Hz, 1H), 7.58 – 7.50 (m, 1H), 3.90 – 3.84 (m, 2H), 2.84 – 2.70 (m, 2H), 1.96 – 1.84 (m, 6H).
^{13}C NMR (101 MHz, $CDCl_3$) δ 176.1, 149.6, 146.3, 138.0, 131.2, 129.3, 129.2, 128.1, 127.7, 127.1, 53.1, 37.7, 29.9, 29.2, 23.6.
IR (film) ν_{max}/cm^{-1} 2933, 1803, 1726, 1657, 1467, 1440, 1408.
HRMS (ESI+) exact mass calculated for $[M+H]^+$ ($C_{15}H_{17}N_2O$) requires m/z 241.1335, found m/z 241.1329.
mp 109 – 112 °C.

1-(pyrimidin-5-yl)piperidin-2-one (2v)

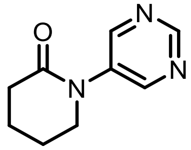

To a flame-dried reaction flask was added, CuI (480 mg, 0.5 eq, 2.5 mmol), K_3PO_4 (2.12 g, 10.0 mmol, 2.0 eq) and δ-valerolactam (500 mg, 5.0 mmol, 1 eq). The vessel was sealed and subjected to three cycles of evacuation and backfilling with argon. Sequentially, *N,N*-dimethylethylenediamine (400 µL, 3.8 mmol, 0.75 eq) and 5-bromopyrimidine (1.20 g, 7.5 mmol, 1.5 eq) were added. The reaction mixture was heated to 110 °C for 24 h, before cooling to ambient

temperature and filtered through a short pad of Celite, following dilution with CH_2Cl_2. The crude residue was purified using FCC (eluting with 50-80% EtOAc in pentane) to afford the title compound as a brown solid (440 mg, 49%).

^{1}H NMR (400 MHz, $CDCl_3$) δ 9.00 (s, 1H), 8.73 (s, 2H), 3.69 (t, *J* = 5.9 Hz, 2H), 2.56 (t, *J* = 6.4 Hz, 2H), 2.07 – 1.86 (m, 4H).
^{13}C NMR (101 MHz, $CDCl_3$) δ 170.3, 155.6, 153.4, 138.2, 50.7, 32.8, 23.3, 21.3.
IR (film) ν_{max}/cm^{-1} 2957, 1637, 1560, 1486, 1428, 1415, 1311.
HRMS (ESI+) exact mass calculated for $[M+Na]^+$ ($C_9H_{11}N_3ONa$) requires m/z 200.0794, found m/z 200.0785.
mp 100 – 102 °C

1-(pyridin-2-yl)piperidin-2-one (2w)

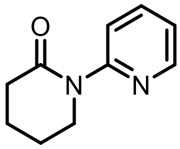

Prepared from 2-bromopyridine (8 mmol) and δ-valerolactam (10.4 mmol) according to General Procedure A to give the desired product as a yellow oil (1.11 g, 79%).

^{1}H NMR ($CDCl_3$, 400 MHz) δ 8.41 (ddd, *J* = 4.9, 2.0, 0.9 Hz, 1H), 7.75 dt, *J* = 8.3, 1.0 Hz, 1H), 7.66 (ddd, *J* = 8.3, 7.2, 2.0 Hz, 1H), 7.07 (ddd, *J* = 7.2, 4.9, 1.1 Hz, 1H), 3.97 – 3.88 (m, 2H), 2.64 – 2.52 (m, 2H), 1.99 – 1.86 (m, 4H).
^{13}C NMR ($CDCl_3$, 101 MHz) δ 171.0, 154.7, 147.9, 137.1, 120.9, 120.7, 47.9, 33.7, 23.3, 21.2.
Spectral data is consistent with the literature [11].

1,4-dioxa-8-azaspiro[4.6]undecan-9-one (1y)

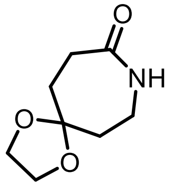

Following modified literature conditions [7,12], 1,4-dioxaspiro[4.5]decan-8-one (10 mmol, 1 eq) and hydroxylamine.HCl (15 mmol, 1.5 eq) was dissolved in a mixture of EtOH:H_2O (5:1, 0.16 M). Sodium acetate (15 mmol, 1.5 eq) was added and the resulting mixture was heated to reflux for 18 h. Following cooling to rt, the volatiles were removed under reduced pressure. The concentrated crude was diluted with EtOAc and washed with pH 7.5 buffer. The organic phase was collected, dried over Na_2SO_4 and concentrated *in vacuo* to give a white solid which was used in the next step without further purification.

To a flame-dried Schlenk tube was added the oxime (4.08 mmol, 1 eq) and THF (0.5 M). The Schlenk tube was placed in a water bath at rt and diethylaminosulfur trifluoride (6.12 mmol, 1.5 eq) was added before stirring at rt for 1 h. Carefully, H_2O was added to quench the reaction and the aqueous phase extracted with CH_2Cl_2 (3 × 10 mL). The combined organic layer was dried over Na_2SO_4, filtered and concentrated under reduced pressure and the resulting crude material was purified using FCC (eluting with 0-10% MeOH in EtOAc) to afford the desired product as an off-white solid (330 mg, 47%).

^{1}H NMR (400 MHz, $CDCl_3$) δ 6.57 (s, 1H), 3.96 (s, 4H), 3.26 (q, J = 5.7 Hz, 2H), 2.54 – 2.44 (m, 2H), 1.87 – 1.81 (m, 2H), 1.81 – 1.76 (m, 2H).
^{13}C NMR (101 MHz, $CDCl_3$) δ 178.3, 109.3, 64.7, 39.5, 37.9, 33.1, 30.8.
IR (film) ν_{max}/cm^{-1} 3295 (br), 2956, 2886, 1664 (s), 1481, 1444.
HRMS (ESI+) exact mass calculated for $[M+H]^+$ ($C_8H_{14}NO_3$) requires m/z 172.0968, found m/z 172.0966.
mp 100–102 °C.

8-benzyl-1,4-dioxa-8-azaspiro[4.6]undecan-9-one (2y)

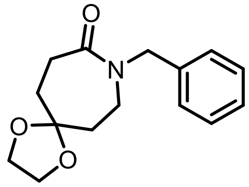

Prepared following General Procedure A from 1,4-dioxa-8-azaspiro[4.6]undecan-9-one (**1y**) (1.69 mmol, 1 eq) and benzyl bromide (1.80 mmol, 1.07 eq) to afford the desired product as a colourless oil (420 mg, 96%).

^{1}H NMR (400 MHz, $CDCl_3$) δ 7.35 – 7.29 (m, 2H), 7.29 – 7.23 (m, 3H), 4.60 (s, 2H), 4.00 – 3.85 (m, 4H), 3.41 – 3.27 (m, 2H), 2.71 – 2.61 (m, 2H), 1.91 – 1.79 (m, 2H), 1.66 – 1.57 (m, 2H).
^{13}C NMR (101 MHz, $CDCl_3$) δ 175.2, 137.7, 128.7, 128.2, 127.6, 109.0, 64.7, 51.1, 44.0, 38.1, 33.3, 31.8.
IR (film) ν_{max}/cm^{-1} 2957, 1643, 1483, 1453, 1114.
HRMS (ESI+) exact mass calculated for $[M+Na]^+$ ($C_{15}H_{19}NO_3Na$) requires m/z 284.1257, found m/z 284.1260.

1,4-oxazepan-5-one (1z)

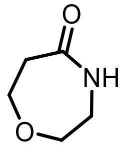

Prepared following modified literature procedures [7,13], tetrahydro-4H-pyran-4-one (10 mmol, 1 eq) and hydroxylamine.HCl (15 mmol, 1.5 eq) were dissolved in a solution of EtOH:H_2O (5:1, 0.16 M). NaOAc (15 mmol, 1.5 eq) was added and the resulting mixture was heated to reflux for 18 h. Following cooling to rt, the volatiles were removed under reduced pressure. The concentrated crude was diluted with EtOAc and washed with pH 7.5 buffer. The organic phase was collected, dried over Na_2SO_4 and concentrated *in vacuo* to give a white solid which was used in the next step without further purification.

The oxime (7.8 mmol, 1 eq) was dissolved in THF (1 M) under an atmosphere N_2 gas and T3P (1.17 mmol, 15mol%, 50% in EtOAc) was added before heating the mixture to reflux for 4 h. The solution was cooled to rt before concentration under educed pressure. The resulting oil was diluted with H_2O and extracted with EtOAc (3 × 50 mL). The combined organic layers were washed with a saturated aqueous solution of $NaHCO_3$, dried over $MgSO_4$, filtered and concentrated under reduced pressure. The crude material was purified using FCC (eluting with 20-40% acetone in pentane) to afford the desired product as an off-white solid (208 mg, 23%).

^{1}H NMR (400 MHz, $CDCl_3$) δ 3.80 (t, *J* = 5.7 Hz, 2H), 3.75 (t, *J* = 5.8 Hz, 2H), 2.67 (t, *J* = 5.8 Hz, 2H), 2.37 (t, *J* = 5.6 Hz, 2H).
^{13}C NMR (101 MHz, $CDCl_3$) δ 156.1, 68.3, 66.8, 32.4, 26.1.
IR (film) ν_{max}/cm^{-1} 3288, 2972, 2859, 1769, 1662, 1440, 1229.
HRMS (ESI+) exact mass calculated for $[M+H]^+$ ($C_5H_{10}NO_2$) requires m/z 116.0706, found m/z 116.0705.
mp 84 – 86 °C.

5-(*tert*-butyl)azepan-2-one (1aa)

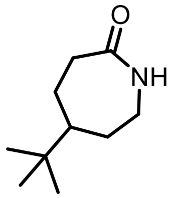

Following modified literature procedures [7,8], 4-(tert-butyl)cyclohexan-1-one (10 mmol, 1 eq) and hydroxylamine.HCl (15 mmol, 1.5 eq) were dissolved in a mixture of EtOH:H_2O (5:1, 0.16 M). Sodium acetate (15 mmol, 1.5 eq) was added and the resulting mixture was heated to reflux for 18 h. Following cooling to rt, the volatiles were removed under reduced pressure. The concentrated crude was diluted with EtOAc and washed with pH 7.5 buffer. The organic phase was collected, dried over Na_2SO_4 and concentrated *in vacuo* to give a white solid which was used in the next step without further purification.

To a flame-dried round bottom flask was added the oxime (500 mg, 3 mmol,1 eq), 1,1,1,3,3,3-hexafluoroisopropanol (12 mL, 0.25 M) and hexachlorophosphazene (5 mol%). The resulting mixture was heated to 70 °C for 16 h. The crude mixture was concentrated under reduced pressure before *t*-BuOH (0.5 M), H_2O (5 mmol, 5 eq) and MsOH (12.5 mol%) were added and heated to 80 °C for 2 h. Following cooling to rt, the concentrated crude was purified by FCC to afford the lactam product as a white solid (315 mg, 62%).

^{1}H NMR (400 MHz, $CDCl_3$) δ 6.42 – 6.03 (m, 1H), 3.32 – 3.12 (m, 2H), 2.56 – 2.34 (m, 2H), 2.08 – 1.87 (m, 2H), 1.36 – 1.12 (m, 3H), 0.87 (s, 9H).
^{13}C NMR (126 MHz, $CDCl_3$) δ 179.2, 52.4, 42.4, 35.9, 33.4, 30.8, 27.7, 24.1.
IR (film) ν_{max}/cm^{-1} 3206, 2954, 1668, 1485, 1438, 1365, 1354.
HRMS (ESI+) exact mass calculated for $[M+H]^+$ ($C_{10}H_{19}NONa$) requires m/z 192.1359, found m/z 192.1362.
mp 130 °C (decomposition)

1-benzyl-5-(*tert*-butyl)azepan-2-one (2aa)

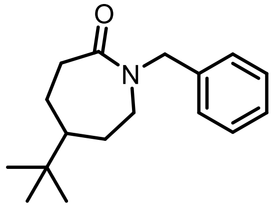

Prepared from 5-(tert-butyl)azepan-2-one (1.47 mmol, 1 eq) and benzyl bromide (1.57 mmol, 1.07 eq) following General Procedure A to afford the titled compound as an off-white solid (380 mg, 85%).

^{1}H NMR (400 MHz, $CDCl_3$) δ 7.36 – 7.28 (m, 2H), 7.28 – 7.23 (m, 3H), 4.76 (d, *J* = 14.7 Hz, 1H), 4.41 (d, *J* = 14.7 Hz, 1H), 3.36 (dd, *J* = 15.4, 10.6 Hz, 1H), 3.22 (ddd, *J* = 15.3, 6.7, 2.0 Hz, 1H), 2.66 (ddd, *J* = 14.1, 7.7, 1.7 Hz, 1H), 2.54 (ddd, *J* = 13.9, 11.4, 2.1 Hz, 1H), 2.06 – 1.94 (m, 1H), 1.80 (dddt, *J* = 15.3, 6.6, 3.4, 1.7 Hz, 1H), 1.72 (s, 1H), 1.34 – 1.14 (m, 2H), 1.03 (dddd, *J* = 14.2, 12.3, 10.6, 2.2 Hz, 1H), 0.83 (s, 9H).
^{13}C NMR (101 MHz, $CDCl_3$) δ 176.0, 138.0, 128.7, 128.3, 127.5, 51.6, 51.0, 48.3, 36.4, 33.2, 29.1, 27.7, 24.3.
Spectral data are consistent with the literature [14].

ethyl 7-oxoazepane-4-carboxylate (2ad)

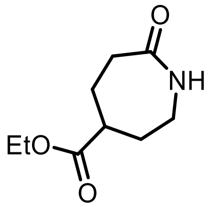

Following modified literature procedures [7,12], ethyl 4-oxocyclohexane-1-carboxylate (10 mmol, 1 eq) and hydroxylamine.HCl (15 mmol, 1.5 eq) was dissolved in a mixture of EtOH:H_2O (5:1, 0.16 M). Sodium acetate (15 mmol, 1.5 eq) was added and the resulting mixture was heated to reflux for 18 h. Following cooling to rt, the volatiles were removed under reduced pressure. The concentrated crude was diluted with EtOAc and washed with pH 7.5 buffer. The organic phase was collected, dried over Na_2SO_4 and concentrated *in vacuo* to give a white solid which was used in the next step without further purification.

To a flame-dried Schlenk tube was added the oxime (3.78 mmol, 1 eq) and THF (0.5 M). The Schlenk tube was placed in a water bath at rt and diethylaminosulfur trifluoride (5.67 mmol, 1.5 eq) was added before stirring at rt for 1 h. Carefully, H_2O was added to quench the reaction and the aqueous phase extracted with CH_2Cl_2 (3 × 10 mL). The combined organic layer was dried over Na_2SO_4, filtered and concentrated under reduced pressure and the resulting crude material was purified using FCC (eluting with 0-5% MeOH in EtOAc) to yield the desired product as a brown solid (340 mg, 49%).

^{1}H NMR (400 MHz, $CDCl_3$) δ 6.69 (s, 1H), 4.13 (q, *J* = 7.1 Hz, 2H), 3.32 (dt, *J* = 13.9, 6.9 Hz, 1H), 3.19 (ddd, *J* = 15.2, 9.9, 5.2 Hz, 1H), 2.66 – 2.50 (m, 2H), 2.49 – 2.38 (m, 1H), 2.13 – 1.96 (m, 2H), 1.91 – 1.72 (m, 2H), 1.24 (t, *J* = 7.1 Hz, 3H).
^{13}C NMR (101 MHz, $CDCl_3$) δ 178.2, 174.4, 60.8, 46.2, 40.7, 34.4, 31.9, 25.3, 14.3.
Spectral data are consistent with the literature [15].

5-phenylazepan-2-one (2ae)

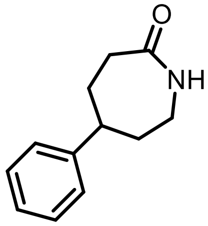

According to a modified literature procedure [16], 4-phenylcyclohexan-1-one (5 mmol, 1 eq) was dissolved in formic acid (2 M). Hydroxylamine-*O*-sulfonic acid (7.5 mmol, 1.5 eq) was added before the mixture was heated at reflux for 6 h. After cooling to rt, the mixture was quenched with H_2O and neutralized with a 5% (w/v) NaOH solution (aq). The aqueous phase was extracted three times using $CHCl_3$ and the combined organic phases dried over Na_2SO_4, filtered, concentrated under reduced pressure and purified using FCC (eluting with 0-5% MeOH in EtOAc) to afford the target compound as a brown solid (770 mg, 81%).

^{1}H NMR (400 MHz, $CDCl_3$) δ 7.34 – 7.27 (m, 2H), 7.24 – 7.19 (m, 1H), 7.19 – 7.15 (m, 2H), 6.66 (s, 1H), 3.45 – 3.24 (m, 2H), 2.76 (tt, *J* = 12.2, 3.5 Hz, 1H), 2.69 – 2.50 (m, 2H), 2.06 – 1.96 (m, 2H), 1.88 – 1.65 (m, 2H).
^{13}C NMR (101 MHz, $CDCl_3$) δ 178.6, 146.5, 128.8, 126.8, 126.6, 49.0, 42.2, 37.5, 36.0, 30.7.
Spectral data are consistent with the literature [17].

ethyl 5-oxohexahydro-1H-pyrrolo[1,2-a]azepine-9a(5H)-carboxylate (4a)

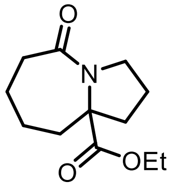

Prepared following the literature procedure below [18]:

Alkylation

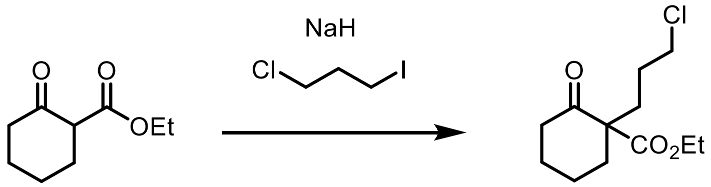

To a flame-dried, three-neck round bottom flask was added NaH (60 % in mineral oil, 15.5 mmol, 1.35 eq). The flask was evacuated and backfilled with N_2 three times. The NaH was washed with hexane (3 × 10 mL) before anhydrous 1,2-dimethoxyethane (16 mL, 0.9 M) was added. Ethyl 2-oxocyclohexane-1-carboxylate (2.26 mL, 14.1 mmol, 1.23 eq) was added dropwise. After gas evolution has ceased, and a precipitate formed, 1-chloro-3-iodo-propane (1.9 mL, 17.6 mmol, 1.53 eq) was added and the resulting mixture heated to reflux for 20 h. Following cooling to r.t, diethyl ether (200 mL) was added and the mixture extracted with $NaHCO_3$ (0.65 M, 1 × 100 mL) and brine (1 × 50 mL). The organic layer was dried with $MgSO_4$ and concentrated *in vacuo*.

Azidation

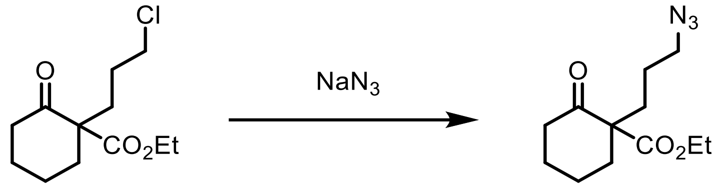

The crude oil was dissolved in DMF (16 mL, 0.9 M) and NaN_3 (743.6 mg, 11.5 mmol, 1.0 eq) was added carefully. The mixture was heated to 80 °C for 3 h. Diethyl ether (400 mL) was added, washed with H_2O (1 × 50 mL) and brine (1 × 50 mL), and then dried over $MgSO_4$ and concentrated *in vacuo*. The crude oil was purified by FCC (eluting with 30% Et_2O in pentane) to afford the ketoazide (1.91 g, 66%).

Schmidt rearrangement

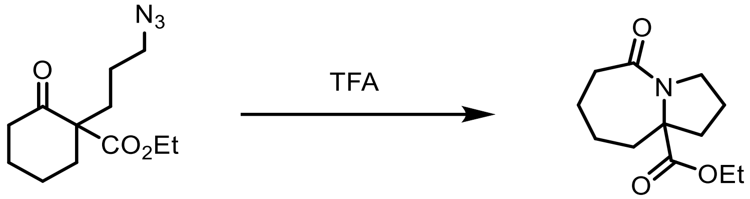

A portion of the ketoazide (1.19 g, 4.7 mmol) was dissolved in 2,2,2-trifluoroacetic acid and stirred for 1-2 h at ambient temperature (*ca.* 25 °C). Excess toluene was added the resulting solution concentrated under reduced pressure. Diethyl ether (200 mL) was added to the resulting oil, which was washed with saturated $NaHCO_3$ (aq.) (1 × 50 mL) and brine (1 × 50 mL). The organic layer was dried over $MgSO_4$ and concentrated *in vacuo*. The crude mixture was purified by FCC (eluting with 70% acetone in pentane) to give the titled product as a brown oil (490 mg, 62%).

^{1}H NMR ($CDCl_3$, 400 MHz) δ 4.23 (m, 2H), 3.79 (dddd, *J* = 11.6, 7.9, 3.6, 1.0 Hz, 1H), 3.54 (ddd, *J* = 11.8, 9.0, 7.1 Hz, 1H), 2.56 (dd, *J* = 14.7, 7.0 Hz, 1H), 2.53 – 2.47 (m, 1H), 2.34 (ddd, *J* = 12.6, 6.2, 3.8 Hz, 1H), 2.25 (m ~ t, 1H), 2.03 (ddd, *J* = 13.0, 11.1, 6.8 Hz, 1H), 1.85 – 1.80 (m, 1H), 1.79 (dd, *J* = 6.9, 3.4 Hz, 1H), 1.69 – 1.77 (m, 2H), 1.60 – 1.40 (m, 3H), 1.28 (t, *J* = 7.1 Hz, 3H).
^{13}C NMR ($CDCl_3$, 101 MHz) δ 174.5, 173.5, 69.0, 61.8, 49.2, 42.9, 38.3, 38.1, 26.7, 23.0, 21.6, 14.4.
Spectral data are consistent with the literature [18].

ethyl 2-(4-((2-oxocyclopentyl)methyl)phenyl)propanoate (S1-1)

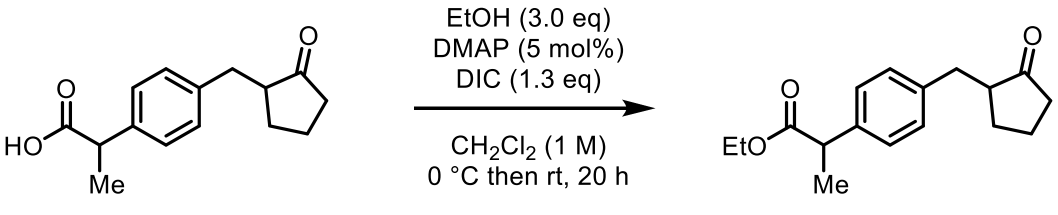

A round bottom flask was charged with 2-(4-((2-oxocyclopentyl)methyl)phenyl)propanoic acid (loxoprofen) (5.0 g, 20.2 mmol, 1 eq) and CH_2Cl_2 (1 M). DMAP (123 mg, 5 mol%), EtOH (3.5 mL, 60.6 mmol, 3 eq) and DIC (4.15 mL, 26.3 mmol, 1.3 eq) were added at 0 °C before stirring the reaction mixture at rt for 20 h. The volatiles were removed under reduced pressure and the

mixture diluted with CH_2Cl_2 (200 ml) and sequentially washed with HCl (0.5 M) aq. and sat. $NaHCO_3$ aq. solutions. The organic layer was dried over Na_2SO_4 and concentrated *in vacuo* and the resulting crude material purified using FCC (eluting with 5-20% EtOAc in pentane) to afford the product as a colourless oil (5.4 g, 96%).

^{1}H NMR (400 MHz, $CDCl_3$) δ 7.21 (d, *J* = 8.1 Hz, 2H), 7.11 (d, *J* = 8.1 Hz, 2H), 4.21 – 4.03 (m, 2H), 3.67 (q, *J* = 7.2 Hz, 1H), 3.12 (dd, *J* = 13.9, 4.1 Hz, 1H), 2.50 (dd, *J* = 13.9, 9.5 Hz, 1H), 2.41 – 2.26 (m, 2H), 2.19 – 2.03 (m, 2H), 2.02 – 1.89 (m, 1H), 1.82 – 1.65 (m, 1H), 1.60 – 1.51 (m, 1H), 1.47 (d, *J* = 7.2 Hz, 3H), 1.21 (t, *J* = 7.1 Hz, 3H).
^{13}C NMR (101 MHz, $CDCl_3$) δ 220.3, 174.8, 138.9, 138.7, 129.2, 127.6, 60.8, 51.1, 45.3, 38.3, 35.3, 29.4, 20.7, 18.7, 14.3.
HRMS (ESI+) exact mass calculated for $[M+NH_4]^+$ ($C_{17}H_{26}NO_3$) requires m/z 292.1907, found m/z 292.1896.
Spectral data are consistent with the literature [19].

ethyl 2-(4-((1-benzyl-6-oxopiperidin-2-yl)methyl)phenyl)propanoate (4f)

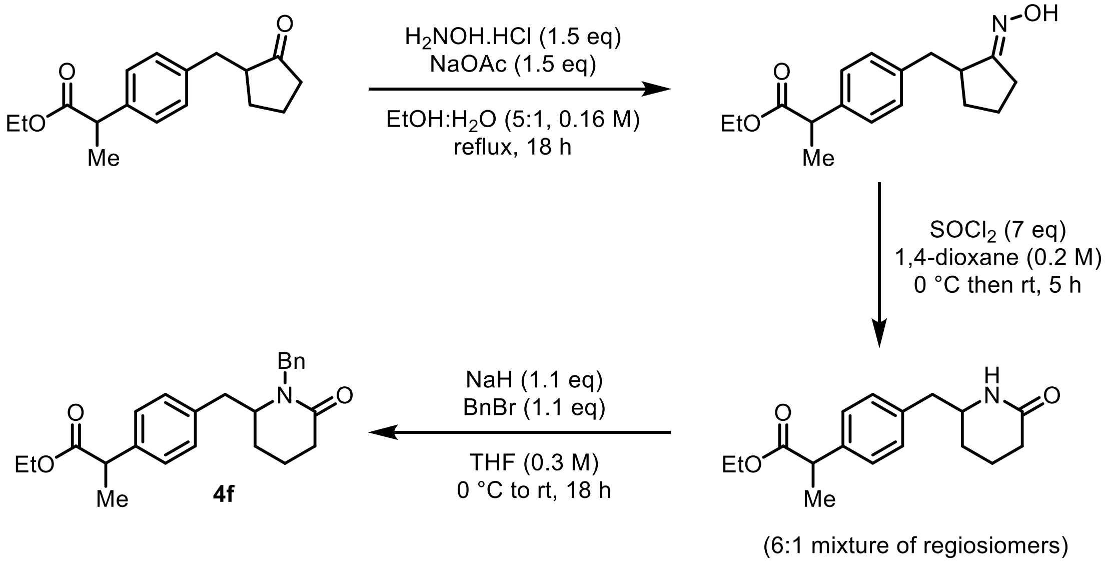

Oxime synthesis:
Following modified literature conditions [7,12], ethyl 2-(4-((2-oxocyclopentyl)methyl)phenyl)propanoate (15 mmol, 1 eq) and hydroxylamine.HCl (22.5 mmol, 1.5 eq) was dissolved in a mixture of $EtOH:H_2O$ (5:1, 0.16 M). Sodium acetate (22.5 mmol, 1.5 eq) was added and the resulting mixture was heated to reflux for 18 h. Following cooling to rt, the volatiles were removed under reduced pressure. The concentrated crude was diluted with EtOAc and washed with pH 7.5 buffer. The organic phase was collected, dried over Na_2SO_4 and concentrated *in vacuo* to give a colourless oil which was used in the next step without further purification.

Beckmann rearrangement:
To a solution of the oxime (7.3 mmol, 1 eq) in anhydrous 1,4-dioxane (0.2 M) at 0 °C was added freshly distilled thionyl chloride (50 mmol, 7 eq) dropwise. The reaction mixture was allowed to warm to ambient conditions before stirring for 5 h. Following quenching with H_2O, the mixture was extracted with EtOAc (3 × 150 mL). The combined organic layers were dried over Na_2SO_4, before concentration *in vacuo* and purification using FCC (eluting with 0-5% MeOH in EtOAc) to afford a black oil (1.45 g, 70%) as 6:1 mixture of regioisomers.

Benzylation:
Following General Procedure A, to a round bottom flask containing washed NaH (60 wt% in mineral oil) (5.0 mmol, 1.1 eq) under an atmosphere of N_2, was added a mixture of the regiosiomers of X (1.3 g, 4.5 mmol, 1.0 eq) at 0 °C. The reaction contents were stirred at rt for 1.5 h before the addition of BnBr (5.0 mmol, 1.1 eq) and further stirring at rt for 18 h. The mixture was quenched with H_2O, extracted with EtOAc (3 × 80 mL) and the combined organic layers dried over Na_2SO_4 and purified using FCC (eluting with 20-50% acetone in pentane) to afford the title compound **4f** as an orange oil (538 mg, 32%).

^{1}H NMR (400 MHz, $CDCl_3$) δ 7.32 (t, *J* = 7.1 Hz, 2H), 7.29 – 7.23 (m, 3H), 7.21 (d, *J* = 8.1 Hz, 2H), 7.01 (d, *J* = 8.2 Hz, 2H), 5.47 (d, *J* = 15.1 Hz, 1H), 4.18 – 4.02 (m, 2H), 3.97 (d, *J* = 15.2 Hz, 1H), 3.66 (q, *J* = 7.2 Hz, 1H), 3.54 – 3.43 (m, 1H), 3.06 (dd, *J* = 13.6, 4.3 Hz, 1H), 2.65 (dd, *J* = 13.6, 10.1 Hz, 1H), 2.56 – 2.46 (m, 2H), 2.07 – 1.89 (m, 1H), 1.80 – 1.68 (m, 1H), 1.68 – 1.52 (m, 2H), 1.46 (d, *J* = 7.1 Hz, 3H), 1.19 (t, *J* = 7.1 Hz, 3H).
^{13}C NMR (101 MHz, $CDCl_3$) δ 174.6, 170.5, 139.2, 137.6, 137.0, 129.4, 128.8, 128.0, 127.9, 127.5, 60.9, 57.0, 48.0, 45.3, 38.3, 31.9, 25.7, 18.7, 17.0, 14.2.
IR (film) ν_{max}/cm^{-1} 2941, 1730, 1638, 1513, 1495, 1415, 1174.
HRMS (ESI+) exact mass calculated for $[M+Na]^+$ ($C_{24}H_{29}NO_3Na$) requires m/z 402.2040, found m/z 402.2054.

3-(3-(cyclopentyloxy)-4-methoxyphenyl)cyclohexan-1-one (4h)

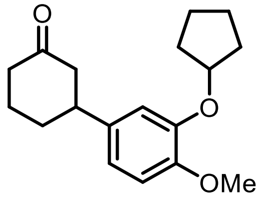

Prepared following the synthetic sequence below.

Williamson etherification:

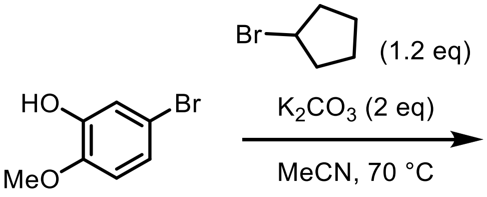

S1-2

A round-bottom flask was charged with a magnetic stir bar, 5-bromo-2-methoxyphenol (10.0 g, 49 mmol, 1 eq), K_2CO_3 (13.5 g, 98 mmol, 2 eq) and MeCN (160 mL, 0.3 M). To the stirred mixture

was added bromocyclopentane (6.3 mL, 59 mmol, 1.2 eq). The mixture was heated under reflux for 48 hrs. Following cooling, the mixture was diluted with H_2O and extracted with EtOAc (3 × 300 mL). The combined organic layers were dried over Na_2SO_4 and concentrated *in vacuo*, before purification using FCC (eluting with 0 – 20% EtOAc in pentane) to afford the product as a colourless oil (11.0 g, 83%).

4-bromo-2-(cyclopentyloxy)-1-methoxybenzene (S1-2)

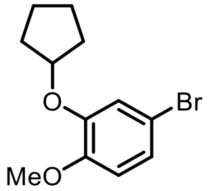

^{1}H NMR (400 MHz, $CDCl_3$) δ 7.02 – 6.96 (m, 2H), 6.72 (d, *J* = 8.29 Hz, 1H), 4.73 (tt, *J* = 6.38, 3.09 Hz, 1H), 3.81 (s, 3H), 2.03 – 1.73 (m, 6H), 1.68 – 1.53 (m, 2H).
^{13}C NMR (101 MHz, $CDCl_3$) δ 149.4, 148.7, 123.3, 118.1, 113.4, 112.8, 80.9, 56.3, 32.9, 24.2.

Spectral data are consistent with the literature.[20]

Alkylation:

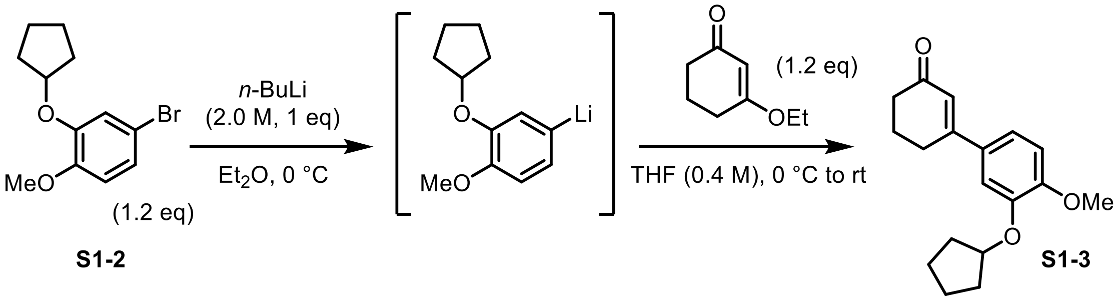

S1-2 **S1-3**

To a three-neck round-bottom flask was added the aryl bromide (10.5 g, 38.7 mmol, 1.2 eq) and a magnetic stir bar. The flask was sealed and subject to three cycles of evacuation and backfilling with N_2 gas before dry Et_2O (0.2 M) was added and the resulting solution cooled to 0 °C. A solution of *n*-BuLi (16.2 mL, 2.0 M in hexane, 32.3 mmol, 1 eq) was added slowly and the mixture allowed to stir at 0 °C for 30 min. Separately, solution of 3-ethoxycyclohex-2-en-1-one (5.65 mL, 38.7 mmol, 1.2 eq) in THF (0.4 M) was prepared under inert conditions and subsequently added dropwise to the now cloudy-white aryl lithium mixture at 0 °C. The combined mixture was stirred at rt for 18 h. To the clear yellow reaction solution was added aqueous HCl (50 mL, 2 N) and the mixture left to stir for an additional 6 h at rt. The mixture was neutralised with saturated $NaHCO_3$ (aq.) and extracted with Et_2O (3 × 200 mL) before the combined organic layers were dried over Na_2SO_4 and concentrated *in vacuo*. The resulting residue was recrystallised from Et_2O to afford the desired α,β-unsaturated ketone **S1-3** as white crystals (6.64 g, 72%).

3'-(cyclopentyloxy)-4'-methoxy-5,6-dihydro-[1,1'-biphenyl]-3(4H)-one (S1-3)

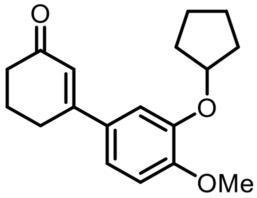

^{1}H NMR (400 MHz, $CDCl_3$) δ 7.13 (dd, *J* = 8.4, 2.1 Hz, 1H), 7.09 (d, *J* = 2.3 Hz, 1H), 6.87 (d, *J* = 8.4 Hz, 1H), 6.38 (d, *J* = 1.6 Hz, 1H), 4.83 – 4.74 (m, 1H), 3.87 (s, 3H), 2.75 (t, *J* = 6.0 Hz, 2H), 2.47 (t, *J* = 6.6 Hz, 2H), 2.13 (p, *J* = 6.2 Hz, 2H), 2.02 – 1.77 (m, 6H), 1.67 – 1.56 (m, 2H).
^{13}C NMR (101 MHz, $CDCl_3$) δ 200.1, 159.5, 152.1, 147.8, 131.1, 123.9, 119.5, 112.7, 111.6, 80.7, 56.2, 37.3, 32.9, 28.0, 24.1, 22.9.
IR (film) νmax/cm^{-1} 2951, 1653, 1590, 1574, 1253.
HRMS (ESI+) exact mass calculated for $[M+Na]^+$ ($C_{18}H_{22}O_3Na$) requires m/z 309.1461, found m/z 309.1466.
mp 90 – 92 °C

Hydrogenation:

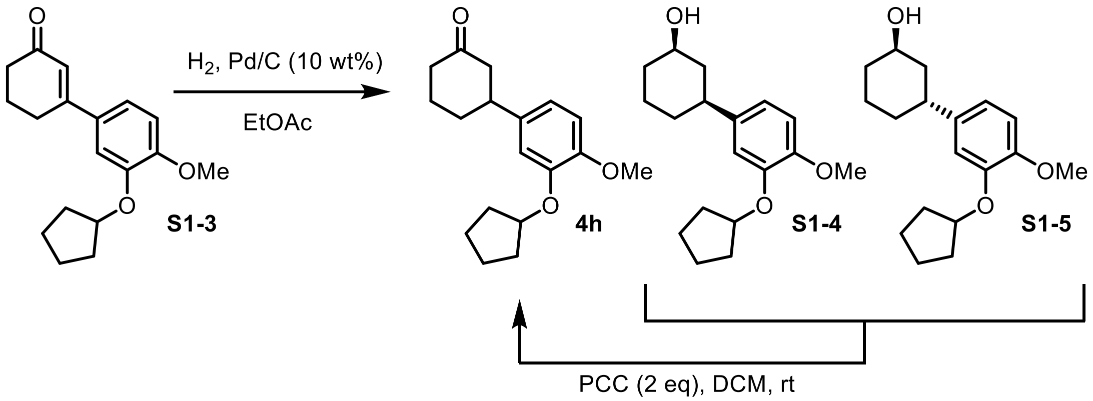

To a 3-necked round bottom flask was added a magnetic stir bar, 10% Pd/C (650 mg, 10% wt) and the α,β-unsaturated ketone starting material **S1-3** (6.5 g, 22.7 mmol, 1 eq). The flask was sealed and subject to three cycles of evacuation and backfilling with N_2. A small quantity of EtOAc was added through a rubber septum (*ca.* 5 mL). The flask was then subject to 5 cycles of evacuation and backfilling with H_2 gas, introduced using a balloon. EtOAc (140 mL) was then added and the resulting mixture stirred at room temperature for 3 h. Upon full consumption of the staring material, as determined by TLC, the mixture was filtered through a pad of Celite, washing the residue with EtOAc. The filtrate was concentrated under reduced pressure and purified using FCC (eluting with 0 – 40% EtOAc in pentane) to afford the desired ketone **4h** as a colourless oil (3.49 g, 56%), the *cis*-alcohol **S1-4** as a white solid (2.17 g, 33%) and the *trans*-alcohol **S1-5** as a white solid (300 mg, 5%).

The two 'over-reduced' alcohol products were subsequently oxidised to the desired ketone using the following procedure.
The *cis*-alcohol **S1-4** (2.0 g, 6.9 mmol, 1 eq) was dissolved in CH_2Cl_2 (0.4 M) followed by addition of Celite (*ca.* 3 g, 100 wt% of PCC). To the slurry was added pyridinium chlorochromate (2.97 g, 13.8 mmol, 2 eq), which was then stirred for 3 h, until full consumption of the starting alcohol, as determined by TLC. The brown mixture was filtered through a pad of Celite and the residue concentrated under reduced pressure and purified using FCC (eluting with 10 – 30% EtOAc in pentane) to afford the desired ketone **4h** as a colourless oil (1.7 g, 88%).
This procedure was repeated for the *trans*-alcohol **S1-5** (300 mg, 1.0 mmol, 1 eq) to afford the identical ketone product **4h** (270 mg, 91%).

3-(3-(cyclopentyloxy)-4-methoxyphenyl)cyclohexan-1-one (4h)

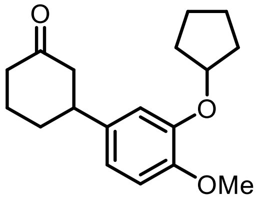

^{1}H NMR (400 MHz, $CDCl_3$) δ 6.82 (d, *J* = 8.8 Hz, 1H), 6.77 – 6.71 (m, 2H), 4.76 (tt, *J* = 5.7, 3.6 Hz, 1H), 3.83 (s, 3H), 2.95 (tt, *J* = 11.5, 3.9 Hz, 1H), 2.67 – 2.54 (m, 1H), 2.53 – 2.47 (m ~ d, 1H), 2.47 – 2.30 (m, 2H), 2.19 – 2.03 (m, 2H), 1.97 – 1.71 (m, 8H), 1.68 – 1.53 (m, 2H).
^{13}C NMR (126 MHz, $CDCl_3$) δ 211.3, 148.9, 147.8, 137.1, 118.4, 114.1, 112.3, 80.6, 56.3, 49.4, 44.4, 41.3, 33.0, 32.9, 25.6, 24.1.
IR (film) vmax/cm^{-1} 2955, 1710, 1589, 1514, 1262, 1246.
HRMS (ESI+) exact mass calculated for $[M+H]^+$ ($C_{18}H_{25}O_3$) requires m/z 289.1798, found m/z 289.1798.

(±)-(*cis*)-3-(3-(cyclopentyloxy)-4-methoxyphenyl)cyclohexan-1-ol (S1-4)

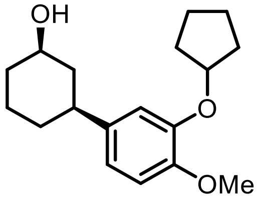

^{1}H NMR (400 MHz, $CDCl_3$) δ 6.80 (d, *J* = 8.0 Hz, 1H), 6.75 – 6.70 (m, 2H), 4.77 (tt, *J* = 5.7, 3.8 Hz, 1H), 3.82 (s, 3H), 3.73 (tq, *J* = 10.9, 4.3 Hz 1H), 2.51 (tt, *J* = 12.2, 3.4 Hz, 1H), 2.23 – 2.11 (m, 1H), 2.11 – 1.99 (m, 1H), 1.98 – 1.76 (m, 8H), 1.68 – 1.54 (m, 2H), 1.48 (d, *J* = 4.5 Hz, 1H), 1.47 – 1.37 (m, 2H), 1.34 – 1.18 (m, 2H).
^{13}C NMR (101 MHz, $CDCl_3$) δ 148.5, 147.7, 139.1, 118.6, 114.3, 112.2, 80.5, 71.2, 56.3, 43.6, 42.4, 35.5, 33.8, 33.0, 24.6, 24.2.
IR (film) vmax/cm^{-1} 3272 (br), 2926, 1589, 1518, 1258.
HRMS (ESI+) exact mass calculated for $[M+Na]^+$ ($C_{18}H_{26}O_3Na$) requires m/z 313.1774, found m/z 313.1777.
mp 64 – 65 °C

(±)-(*trans*)-3-(3-(cyclopentyloxy)-4-methoxyphenyl)cyclohexan-1-ol (S1-5)

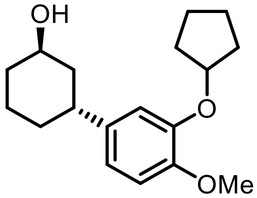

^{1}H NMR (400 MHz, $CDCl_3$) δ 6.80 (d, *J* = 8.1 Hz, 1H), 6.77 – 6.69 (m, 2H), 4.82 – 4.72 (m, 1H), 4.26 – 4.19 (m, 1H), 3.81 (s, 3H), 2.93 (tt, *J* = 12.3, 3.5 Hz, 1H), 2.02 – 1.76 (m, 10H), 1.71 – 1.51 (m, 6H), 1.51 – 1.35 (m, 1H).
^{13}C NMR (101 MHz, $CDCl_3$) δ 148.3, 147.6, 139.9, 118.6, 114.5, 112.2, 80.5, 67.0, 56.3, 40.8, 37.1, 34.1, 32.9, 32.9, 32.6, 24.1, 20.6.
IR (film) vmax/cm^{-1} 3293 (br), 2925, 1586, 1518, 1236.
HRMS (ESI+) exact mass calculated for $[M+Na]^+$ ($C_{18}H_{26}O_3Na$) requires m/z 313.1774, found m/z 313.1769.

mp 64 – 66 °C

1-benzyl-6-(3-(cyclopentyloxy)-4-methoxyphenyl)azepan-2-one (4i) and **1-benzyl-6-(3-(cyclopentyloxy)-4-methoxyphenyl)azepan-2-one (4l)**

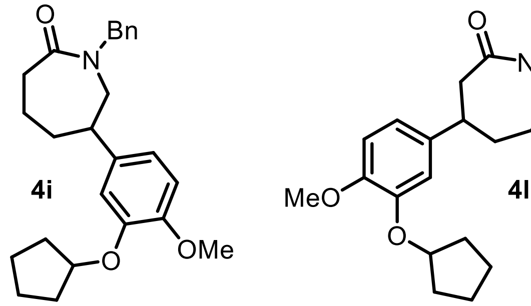

Compounds **4i** and **4l** were prepared from ketone **4h** following the sequence below.

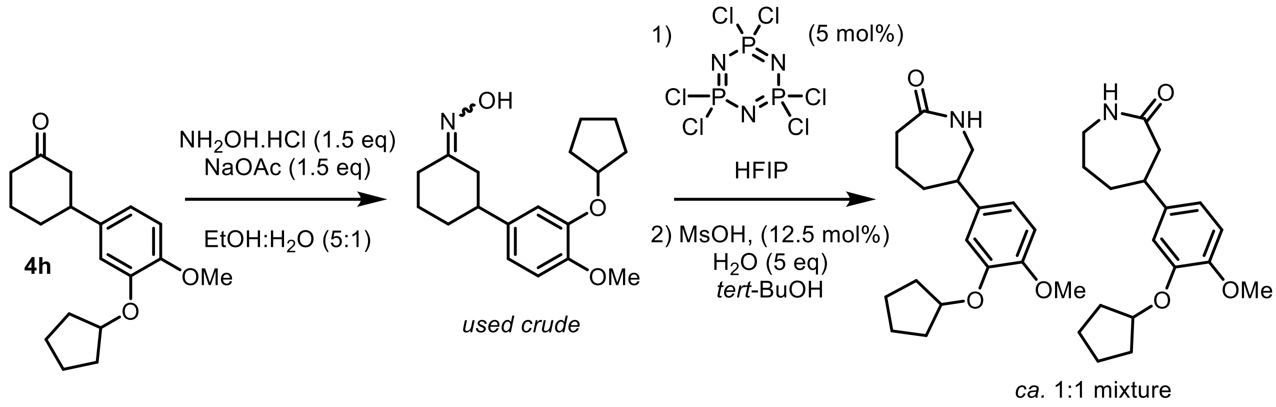

Oxime formation

Following modified literature conditions [7,12], ketone **4h** (5.87 g, 20.4 mmol, 1 eq) and EtOH:H_2O (5:1, 0.2 M) were added to a round bottom flask with a magnetic stir bar. Following dissolution, NH_2OH.HCl (2.13 g, 30.6 mmol, 1.5 eq) and NaOAc (2.51 g, 30.6 mmol, 1.5 eq) were added and the resulting mixture heated to reflux for 18 h. Following removal of the volatiles under reduced pressure, the resulting residue was redissolved in EtOAc and washed with saturated $NaHCO_3$ (aq). The organic layer was dried over Na_2SO_4 and concentrated *in vacuo* to give a white solid (6.07 g, 98%) as a 1:1 mixture of diastereomers, which were used in the next step without purification.

Beckmann rearrangement

Prepared following modified literature procedures [7,8], the white solid (5.99 g, 19.7 mmol, 1 eq) was dissolved in 1,1,1,3,3,3-hexafluoro-2-propanol (HFIP) (80 mL, 0.25 M) and hexachlorophosphazene (340 mg, 5 mol%) added, before the resulting solution was heated to reflux for 18 h. Following cooling to ambient temperature, the solvent was removed *in vacuo* and the resulting residue redissolved in *t*-BuOH (80 mL, 0.25 M) along with the addition of H_2O (1.80 mL, 98.5 mmol, 5 eq) and MsOH (160 µL, 2.46 mmol, 12.5 mol%). The mixture was heated at 80 °C for 2 h before cooling and removal of the solvent under reduced pressure. The crude material was purified by FCC (eluting with 100% EtOAc) to afford the desired (inseparable) lactams as an off-white solid (4.26 g, 71%) in a *ca.* 1:1 ratio.

Benzylation

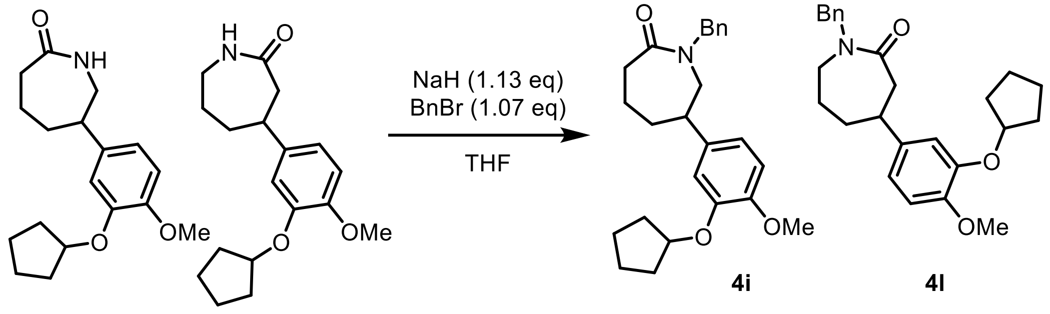

ca. 1:1 mixture

According to General Procedure A, a 1:1 mixture of the lactams (1.0 g, 3.29 mmol, 1 eq) was reacted with NaH (60% dispersion in mineral oil) (1.13 eq) and BnBr (1.07 eq) in THF (0.3 M) and purified by FCC (eluting with 20 – 50% EtOAc in petroleum ether) to afford the desired benzylated lactams **4i** (630 mg, 49%) and **4l** (600 mg, 47%) as thick, pale-yellow oils.

1-benzyl-6-(3-(cyclopentyloxy)-4-methoxyphenyl)azepan-2-one (4i)

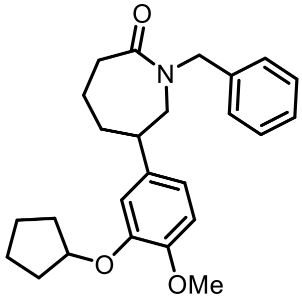

^{1}H NMR (400 MHz, $CDCl_3$) δ 7.38 – 7.25 (m, 5H), 6.73 (d, *J* = 8.2 Hz, 1H), 6.44 (dd, *J* = 8.2, 2.1 Hz, 1H), 6.35 (d, *J* = 2.1 Hz, 1H), 4.87 (d, *J* = 14.5 Hz, 1H), 4.69 – 4.62 (m, 1H), 4.34 (d, *J* = 14.4 Hz, 1H), 3.79 (s, 3H), 3.63 (dd, *J* = 15.0, 9.9 Hz, 1H), 3.16 (d, *J* = 15.0 Hz, 1H), 2.76 – 2.64 (m, 2H), 2.31 (t, *J* = 10.6 Hz, 1H), 2.09 – 1.96 (m, 2H), 1.94 – 1.77 (m, 6H), 1.75 – 1.55 (m, 4H).
^{13}C NMR (101 MHz, $CDCl_3$) δ 175.7, 148.9, 147.7, 138.0, 137.3, 128.7, 128.7, 127.6, 118.3, 114.2, 112.2, 80.5, 56.2, 55.7, 51.5, 45.2, 37.7, 37.1, 33.0, 32.9, 24.1, 24.1, 23.7.
IR (film) vmax/cm^{-1} 2969, 1643, 1514, 1480, 1453.
HRMS (ESI+) exact mass calculated for $[M+H]^+$ ($C_{25}H_{32}NO_3$) requires m/z 394.2377, found m/z 394.2376.

1-benzyl-4-(3-(cyclopentyloxy)-4-methoxyphenyl)azepan-2-one (4l)

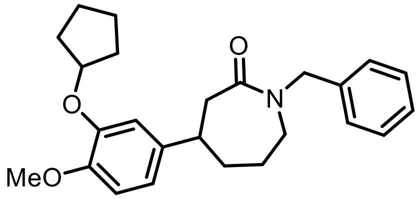

^{1}H NMR (400 MHz, $CDCl_3$) δ 7.39 – 7.27 (m, 5H), 6.80 (d, *J* = 8.6 Hz, 1H), 6.74 – 6.67 (m, 2H), 4.83 – 4.69 (m, 2H), 4.50 (d, *J* = 14.6 Hz, 1H), 3.82 (s, 3H), 3.50 (dd, *J* = 15.2, 10.9 Hz, 1H), 3.27 (dd, *J* = 15.2, 6.1 Hz, 1H), 2.99 (dd, *J* = 13.3, 11.3 Hz, 1H), 2.87 – 2.71 (m, 2H), 2.10 – 1.99 (m, 1H), 1.99 – 1.74 (m, 7H), 1.74 – 1.53 (m, 3H), 1.53 – 1.37 (m, 1H).

^{13}C NMR (101 MHz, $CDCl_3$) δ 174.4, 148.7, 147.7, 139.8, 137.9, 128.7, 128.4, 127.5, 118.3, 114.1, 112.3, 80.6, 56.3, 51.3, 48.7, 44.6, 40.5, 39.1, 33.0, 32.9, 28.3, 24.2.
IR (film) vmax/cm^{-1} 2933, 1639, 1514, 1483, 1452, 1440, 1264.
HRMS (ESI+) exact mass calculated for $[M+H]^+$ ($C_{25}H_{32}NO_3$) requires m/z 394.2377, found m/z 394.2380.

***tert*-butyl 2-oxopiperidine-1-carboxylate (S1-6)**

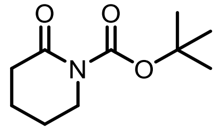

Prepared following a modified literature procedure [21]. To a solution of δ-valerolactam (790 mg, 8 mmol, 1.0 eq) in MeCN (0.3 M) was added DMAP (98 mg, 0.8 mmol, 10 mol%). The solution was cooled to 0 °C and di-*tert*-butyl dicarbonate (2.60 g, 12 mmol, 1.5 eq) was added. The contents were stirred at r.t for 4 h before concentration *in vacuo*. The concentrated crude was purified using FCC (eluting with 5-40% EtOAc in pentane) to yield the product as a white solid (1.52 g, 96%).

^{1}H NMR ($CDCl_3$, 400 MHz) δ 3.61 (t, *J* = 6.4 Hz, 2H), 2.52 – 2.43 (m, 2H), 1.82 – 1.75 (m, 4H), 1.49 (s, 9H).
^{13}C NMR ($CDCl_3$, 101 MHz) δ 171.4, 152.8, 82.8, 46.3, 34.9, 28.1, 22.8, 20.6.
Spectral data are in agreement with the literature [22].

1-tosylazepan-2-one (S1-7)

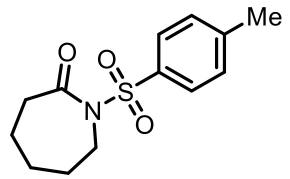

Prepared from ε-caprolactam (10 mmol) and tosyl chloride (10.07 mmol) according to General Procedure A to give the desired product as a white solid (325 mg, 12%).

^{1}H NMR ($CDCl_3$, 400 MHz) δ 7.88 (d, *J* = 8.4 Hz, 2H), 7.33 – 7.27 (d, *J* = 8.3 Hz, 2H), 4.09 – 3.96 (m, 2H), 2.57 – 2.52 (m, 2H), 2.42 (s, 3H), 1.90 – 1.79 (m, 2H), 1.79 – 1.64 (m, 4H).
^{13}C NMR ($CDCl_3$, 101 MHz) δ 174.9, 144.6, 136.7, 129.4, 128.7, 46.6, 38.9, 29.5, 29.4, 23.1, 21.8.
Spectral data are consistent with the literature [23].

1-benzyl-5,5-dimethylpyrrolidin-2-one (5j)

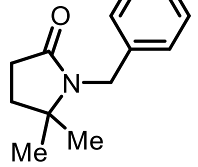

Prepared from 5,5-dimethylpyrrolidin-2-one (4 mmol) and benzyl bromide (4.5 mmol) according to General Procedure A to afford to desired product as a colourless oil (379 mg, 47%).

^{1}H NMR ($CDCl_3$, 400 MHz) δ 7.31 – 7.19 (m, 5H), 4.41 (s, 2H), 2.48 (t, *J* = 7.9 Hz, 2H), 1.87 (t, *J* = 7.9 Hz, 2H), 1.12 (s, 6H).

^{13}C NMR ($CDCl_3$, 101 MHz) δ 174.9, 139.1, 128.5, 127.8, 127.1, 61.1, 42.9, 34.7, 29.8, 27.0.
Spectral data are consistent with the literature [24].

Reaction Intermediates (-OTMP adducts and ketoamides)

1-benzyl-3-((2,2,6,6-tetramethylpiperidin-1-yl)oxy)pyrrolidin-2-one (5a)

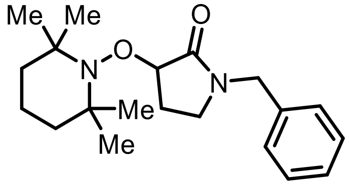

A dry screw-cap vial was charged with 1-benzylpyrrolidin-2-one (350 mg, 2.0 mmol, 1.0 eq), TEMPO (63 mg, 0.4 mmol, 20 mol%) and $TEMPO^{+}TfO^{-}$ (916 mg, 3.0 mmol, 1.5 eq). Anhydrous THF (6.6 mL, 0.3 M) was added to afford a heterogeneous mixture. TIPSOTf (590 µL, 2.2 mmol, 1.1 eq) was added followed by DBU (910 µL, 6.0 mmol, 3.0 eq) which resulted in a clear, red solution which turned black over time. The contents were heated to 40 °C for 16 – 24 h, until the reaction had reached completion as determined by TLC. Saturated aqueous NH_4Cl was added (100 mL) and the biphasic mixture was extracted with EtOAc (3 × 100 mL). The combined organic phases were dried over $MgSO_4$ and concentrated *in vacuo*. The resulting red oil was purified by FCC (eluting with 20% EtOAc in pentane) to yield the desired product as an off-white solid (611 mg, 93%).

^{1}H NMR ($CDCl_3$, 500 MHz) δ 7.39 – 7.33 (m, 2H), 7.33 – 7.28 (m, 1H), 7.28 – 7.24 (m, 2H), 4.67 (dd, *J* = 9.9, 7.8 Hz, 1H), 4.57 (d, *J* = 14.6 Hz, 1H), 4.36 (d, *J* = 14.6 Hz, 1H), 3.16 (td, *J* = 9.3, 1.6 Hz, 1H), 3.02 (td, *J* = 9.5, 6.4 Hz, 1H), 2.49 (dddd, *J* = 12.3, 8.0, 6.4, 1.6 Hz, 1H), 2.03 (dq, *J* = 12.3, 9.4 Hz, 1H), 1.61 (s, 1H), 1.57 – 1.40 (m, 7H), 1.40 – 1.24 (m, 4H), 1.25 – 1.09 (m, 6H).
^{13}C NMR ($CDCl_3$, 126 MHz) δ 172.2, 136.3, 128.8, 128.4, 127.7, 83.3, 61.3, 59.2, 47.1, 42.2, 40.6, 40.5, 34.5, 32.6, 29.1, 20.5, 20.3, 17.3.
Spectral data are consistent with the literature [25].

1-phenyl-3-((2,2,6,6-tetramethylpiperidin-1-yl)oxy)pyrrolidin-2-one (5d)

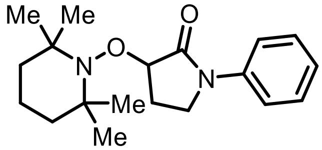

Prepared according to General Procedure C from 1-phenylpyrrolidin-2-one (800 mg, 5.0 mmol) to afford the titled compound as an off-white solid (668 mg, 43%).

^{1}H NMR (400 MHz, $CDCl_3$) δ 7.67 (dd, *J* = 8.8, 1.2 Hz, 2H), 7.38 – 7.32 (m, 2H), 7.13 (t, *J* = 7.4 Hz, 1H), 4.74 (dd, *J* = 10.7, 7.7 Hz, 1H), 3.68 (td, *J* = 9.2, 1.5 Hz, 1H), 3.61 (td, *J* = 9.8, 5.9 Hz, 1H), 2.67 (dddd, *J* = 12.0, 7.6, 6.0, 1.5 Hz, 1H), 2.13 (dtd, *J* = 12.1, 10.3, 8.8 Hz, 1H), 1.59 (s, 1H), 1.54 – 1.45 (m, 4H), 1.40 (s, 3H), 1.31 (s, 4H), 1.21 (s, 3H), 1.15 (s, 3H).
^{13}C NMR (101 MHz, $CDCl_3$) δ 171.5, 139.5, 128.9, 124.6, 119.5, 84.0, 61.4, 59.3, 43.5, 40.6, 40.5, 34.6, 32.5, 29.1, 20.5, 20.4, 17.3.
IR (film) ν_{max}/cm^{-1} 2931, 1710, 1599, 1502, 1398, 1312.
HRMS (ESI+) exact mass calculated for $[M+Na]^{+}$ ($C_{19}H_{28}N_2O_2Na$) requires m/z 339.2043, found m/z 339.2037.
mp 121 – 123 °C.

1-benzyl-5,5-dimethyl-3-((2,2,6,6-tetramethylpiperidin-1-yl)oxy)pyrrolidin-2-one (5k)

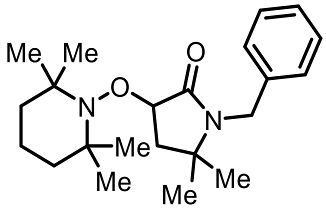

Prepared according to General Procedure C from 1-benzyl-5,5-dimethylpyrrolidin-2-one (1.5 mmol, 1 eq) and purified using FCC (eluting with 30% Et_2O/pentane) to afford the title compound as an off-white solid (450 mg, 83%).

^{1}H NMR (400 MHz, $CDCl_3$) δ 7.34 – 7.25 (m, 4H), 7.25 – 7.18 (m, 1H), 4.71 (dd, *J* = 9.8, 7.8 Hz, 1H), 4.49 (d, *J* = 15.2 Hz, 1H), 4.36 (d, *J* = 15.2 Hz, 1H), 2.40 (dd, *J* = 12.2, 7.8 Hz, 1H), 1.88 (dd, *J* = 12.1, 9.8 Hz, 1H), 1.49 (s, 8H), 1.28 (s, 4H), 1.22 – 1.08 (m, 9H), 1.03 (s, 3H).
^{13}C NMR (101 MHz, $CDCl_3$) δ 172.4, 139.0, 128.6, 128.1, 127.2, 82.7, 61.4, 59.3, 57.1, 45.0, 43.2, 40.6, 40.6, 34.7, 32.7, 28.7, 26.5, 20.6, 20.4, 17.3.
IR (film) ν_{max}/cm^{-1} 3006, 2996, 1698, 1454, 1406.
HRMS (ESI+) exact mass calculated for $[M+Na]^+$ ($C_{22}H_{34}N_2O_2Na$) requires m/z 381.2512, found m/z 381.2527.
mp 92 – 94 °C

1-benzyl-3-((2,2,6,6-tetramethylpiperidin-1-yl)oxy)azepan-2-one (S1-8)

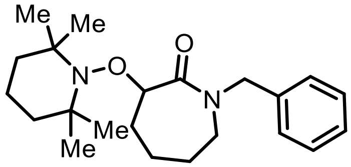

Prepared according to General Procedure C from *N*-benzyl caprolactam (712 mg, 3.5 mmol) to afford the desired product as an off-white solid (1.13 g, 90%).

^{1}H NMR (400 MHz, $CDCl_3$) δ 7.32 – 7.22 (m, 5H), 4.61 (d, *J* = 14.3 Hz, 1H), 4.58 (d, *J* = 9.3 Hz, 1H), 4.49 (d, *J* = 14.4 Hz, 1H), 3.94 (dd, *J* = 14.5, 10.1 Hz, 1H), 3.10 (dd, *J* = 14.8, 5.9 Hz, 1H), 2.28 – 2.18 (m, 1H), 1.98 – 1.82 (m, 1H), 1.71 – 1.53 (m, 3H), 1.53 – 1.41 (m, 5H), 1.30 (s, 1H), 1.27 – 1.16 (m, 4H), 1.16 – 1.07 (m, 9H).
^{13}C NMR (126 MHz, $CDCl_3$) δ 174.7, 137.9, 128.8, 128.5, 127.4, 88.5, 59.9, 59.5, 51.3, 46.6, 40.2, 33.5, 33.5, 28.4, 27.9, 23.6, 21.2, 20.6, 17.2.
HRMS (ESI+) exact mass calculated for $[M+Na]^+$ ($C_{22}H_{34}N_2O_2Na$) requires m/z 381.2512, found m/z 381.2530.
Spectral data are consistent with the literature [26].

1-benzylazepane-2,3-dione (S1-9)

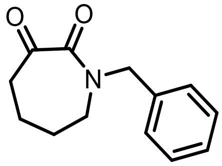

Prepared from 1-benzyl-3-((2,2,6,6-tetramethylpiperidin-1-yl)oxy)azepan-2-one (35.8 mg, 0.1 mmol) when treated with *m*-CPBA in THF as part of the optimisation studies detailed in Table S3. Isolated as a collation of separate experiments due to its presence as a common intermediate – yield therefore not determined. Compound is an off-white solid.

^{1}H NMR (400 MHz, $CDCl_3$) δ 7.40 – 7.27 (m, 5H), 4.61 (s, 2H), 3.34 – 3.25 (m, 2H), 2.61 – 2.50 (m, 2H), 1.94 – 1.81 (m, 2H), 1.65 – 1.52 (m, 2H).
^{13}C NMR ($CDCl_3$, 101 MHz) δ 204.6, 167.3, 136.2, 128.9, 128.6, 128.1, 49.5, 45.5, 38.7, 25.8, 22.0.
IR (film) ν_{max}/cm^{-1} 2947, 1720, 1658, 1496, 1431.
HRMS (ESI+) exact mass calculated for $[M+H]^+$ ($C_{13}H_{16}NO_2$) requires m/z 218.1176, found m/z 218.1176.
mp 75 – 78 °C.

Methylene deletion products

1-benzylpiperidin-2-one (3a)

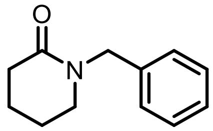

Prepared from 1-benzylazepan-2-one (61.0 mg, 0.3 mmol) according to General Procedure D to give the desired product as a yellow oil (44.8 mg, 79%).

^{1}H NMR ($CDCl_3$, 400 MHz) δ 7.35 – 7.23 (m, 5H), 4.60 (s, 2H), 3.22 – 3.16 (m, 2H), 2.47 (t, J = 6.3 Hz, 2H), 1.84 – 1.71 (m, 4H).
^{13}C NMR (101 MHz, $CDCl_3$) δ 169.9, 137.4, 128.6, 128.1, 127.4, 50.2, 47.3, 32.5, 23.3, 21.5.
Spectral data are consistent with the literature [27].

1-benzylpyrrolidin-2-one (3b)

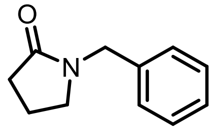

Prepared from 1-benzylpiperidin-2-one (56.8 mg, 0.3 mmol) according to General Procedure D to give the desired product as a colourless oil (33.1 mg, 87%).

^{1}H NMR (400 MHz, $CDCl_3$) δ 7.42 – 7.17 (m, 5H), 4.44 (s, 2H), 3.32 – 3.19 (m, 2H), 2.43 (t, J = 8.1 Hz, 2H), 2.06 – 1.91 (m ~ p, 2H).
^{13}C NMR (101 MHz, $CDCl_3$) δ 175.1, 136.6, 128.7, 128.2, 127.6, 46.7, 46.6, 31.0, 17.8.
Spectral data are consistent with the literature [28].

4-((2-oxopiperidin-1-yl)methyl)benzonitrile (3c)

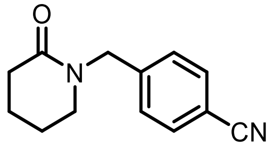

Prepared from 4-((2-oxoazepan-1-yl)methyl)benzonitrile (**2c**) (68.5 mg, 0.3 mmol) according to General Procedure D to give the desired product as an off-white solid (54.3 mg, 84%).

^{1}H NMR ($CDCl_3$, 400 MHz) δ 7.60 (d, *J* = 8.6 Hz, 2H), 7.34 (d, *J* = 8.7 Hz, 2H), 4.62 (s, 2H), 3.24 – 3.18 (m, 2H), 2.46 (t, *J* = 6.1 Hz, 2H), 1.86 – 1.75 (m, 2H).
^{13}C NMR ($CDCl_3$, 101 MHz) δ 170.2, 143.0, 132.5, 128.6, 118.8, 111.3, 50.1, 47.9, 32.4, 23.3, 21.4.
Spectral data are consistent with the literature [29].

1-(4-nitrobenzyl)piperidin-2-one (3d)

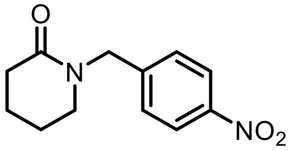

Prepared from 1-(4-nitrobenzyl)azepan-2-one (**2d**) (74.5 mg, 0.3 mmol) according to General Procedure D to give the desired product as a yellow solid (51.7 mg, 74%).

^{1}H NMR ($CDCl_3$, 400 MHz) δ 8.16 (d, *J* = 8.7 Hz, 2H), 7.40 (d, *J* = 8.9 Hz, 2H), 4.66 (s, 2H), 3.26 – 3.19 (m, 2H), 2.51 – 2.44 (m, 2H), 1.89 – 1.73 (m, 4H).
^{13}C NMR ($CDCl_3$, 101 MHz) δ 170.3, 147.4, 145.1, 128.7, 123.9, 50.0, 48.0, 32.4, 23.3, 21.4.
IR (film) ν_{max}/cm^{-1} 2947, 1641, 1519, 1345.
HRMS (ESI+) exact mass calculated for $[M+H]^+$ ($C_{12}H_{15}N_2O_3$) requires m/z 235.1077, found 235.1084.
mp 82 – 86 °C.

1-(4-methoxybenzyl)piperidin-2-one (3e)

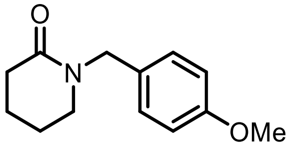

Prepared from 1-(4-methoxybenzyl)azepan-2-one (**2e**) (70.0 mg, 0.3 mmol) according to General Procedure D to give the desired product (52.2 mg, 79%).

^{1}H NMR ($CDCl_3$, 400 MHz) δ 7.18 (d, *J* = 8.6 Hz, 2H), 6.84 (d, *J* = 8.6 Hz, 2H), 4.52 (s, 2H), 3.78 (s, 3H), 3.17 (t, *J* = 5.5 Hz, 2H), 2.44 (t, *J* = 6.3 Hz, 2H), 1.82 – 1.70 (m, 4H).
^{13}C NMR ($CDCl_3$, 101 MHz) δ 170.0, 159.0, 129.6, 129.5, 114.0, 55.4, 49.6, 47.2, 32.5, 23.3, 21.4.
Spectral data are consistent with the literature [30].

1-(4-iodobenzyl)piperidin-2-one (3f)

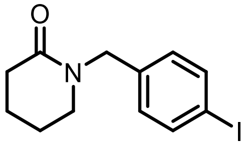

Prepared from 1-(4-iodobenzyl)azepan-2-one (**2f**) (98.8 mg, 0.3 mmol) according to General Procedure D to give the desired product as a colourless oil (69.0 mg, 73%).

^{1}H NMR ($CDCl_3$, 400 MHz) δ 7.63 (d, *J* = 8.3 Hz, 2H), 7.00 (d, *J* = 8.3 Hz, 2H), 4.51 (s, 2H), 3.17 (t, *J* = 5.5 Hz, 2H), 2.44 (t, *J* = 6.3 Hz, 2H), 1.83 – 1.71 (m, 4H).
^{13}C NMR ($CDCl_3$, 101 MHz) δ 170.0, 137.7, 137.2, 130.2, 92.8, 49.8, 47.5, 32.5, 23.3, 21.5.
IR (film) ν_{max}/cm^{-1} 2946, 1637, 1493.
HRMS (ESI+) exact mass calculated for $[M+H]^+$ ($C_{12}H_{15}INO$) requires m/z 316.0193, found m/z 316.0204.

1-(2,6-dichlorobenzyl)piperidin-2-one (3g)

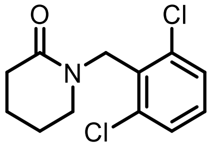

Prepared from 1-(2,6-dichlorobenzyl)azepan-2-one (**2g**) (81.7 mg, 0.3 mmol) according to General Procedure D to give the desired product as a white solid (68.7 mg, 89%).

^{1}H NMR ($CDCl_3$, 400 MHz) δ 7.31 (d, *J* = 7.7 Hz, 2H), 7.17 (dd, *J* = 8.6, 7.4 Hz, 1H), 5.00 (s, 2H), 2.99 (t, *J* = 5.5 Hz, 2H), 2.44 (t, *J* = 6.3 Hz, 2H), 1.80 – 1.66 (m, 4H).
^{13}C NMR ($CDCl_3$, 101 MHz) δ 169.7, 137.0, 132.1, 129.6, 128.6, 45.7, 44.6, 32.6, 23.2, 21.3.
IR (film) ν_{max}/cm^{-1} 2946, 1644, 1490, 1436.
HRMS (ESI+) exact mass calculated for $[M+H]^+$ ($C_{12}H_{14}Cl_2NO$) requires m/z 258.0447, found m/z 258.0448.
mp 94 – 98 °C.

1-(2-bromo-5-methoxybenzyl)piperidin-2-one (3h)

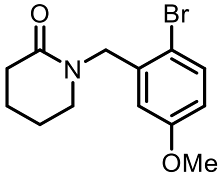

Prepared from 1-(2-bromo-5-methoxybenzyl)azepan-2-one (**2h**) (93.7 mg, 0.3 mmol) according to General Procedure D to give the desired product as a colourless oil (70.1 mg, 78%).

^{1}H NMR ($CDCl_3$, 400 MHz) δ 7.40 (d, *J* = 8.7 Hz, 1H), 6.75 (d, *J* = 3.1 Hz, 1H), 6.67 (dd, *J* = 8.7, 3.1 Hz, 1H), 4.66 (s, 2H), 3.75 (s, 3H), 3.28 – 3.18 (m, 2H), 2.51 – 2.44 (m, 2H), 1.88 – 1.75 (m, 4H).
^{13}C NMR ($CDCl_3$, 101 MHz) δ 170.1, 159.4, 137.3, 133.4, 114.6, 114.3, 114.2, 55.5, 50.1, 47.9, 32.5, 23.4, 21.5.
IR (film) ν_{max}/cm^{-1} 2947, 1644, 1447.
HRMS (ESI+) exact mass calculated for $[M+H]^+$ ($C_{13}H_{17}BrNO_2$) requires m/z 298.0437, found m/z 298.0433.

1-(3,5-dimethoxybenzyl)piperidin-2-one (3i)

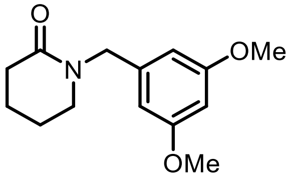

Prepared from 1-(3,5-dimethoxybenzyl)azepan-2-one (**2i**) (79.0 mg, 0.3 mmol) according to General Procedure D to give the desired product as a red oil (50.2 mg, 67%).

^{1}H NMR ($CDCl_3$, 400 MHz) δ 6.39 (d, *J* = 2.3 Hz, 2H), 6.35 (t, *J* = 2.3 Hz, 1H), 4.52 (s, 2H), 3.76 (s, 6H), 3.19 (t, *J* = 5.5 Hz, 2H), 2.46 (t, *J* = 6.3 Hz, 2H), 1.84 – 1.71 (m, 4H).
^{13}C NMR ($CDCl_3$, 101 MHz) δ 170.0, 161.1, 139.8, 106.1, 99.3, 55.4, 50.3, 47.4, 32.5, 23.3, 21.5.
IR (film) ν_{max}/cm^{-1} 2947, 1638, 1610 (2 bands), 1465.
HRMS (ESI+) exact mass calculated for $[M+Na]^+$ ($C_{14}H_{19}NO_3Na$) requires m/z 272.1257, found m/z 272.1266.

1-(3,5-bis(trifluoromethyl)benzyl)piperidin-2-one (3j)

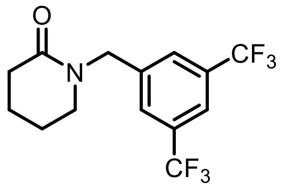

Prepared from 1-(3,5-bis(trifluoromethyl)benzyl)azepan-2-one (**2j**) (101.8 mg, 0.3 mmol) according to General Procedure D to give the desired product as a colourless oil (76.9 mg, 79%).

^{1}H NMR ($CDCl_3$, 400 MHz) δ 7.77 (s, 1H), 7.69 (s, 2H), 4.68 (s, 2H), 3.27 – 3.19 (m, 2H), 2.52 – 2.45 (m, 2H), 1.88 – 1.78 (m, 4H).
^{13}C NMR ($CDCl_3$, 101 MHz) δ 170.4, 140.3, 132.1 (q, *J* = 33.6 Hz), 128.2, 123.3 (d, *J* = 273.0 Hz), 121.6 (hept, *J* = 3.8 Hz), 49.8, 48.0, 32.4, 23.3, 21.4.
^{19}F NMR ($CDCl_3$, 377 MHz) δ -62.9.
IR (film) ν_{max}/cm^{-1} 2952, 1647, 1497, 1381, 1281.
HRMS (ESI+) exact mass calculated for $[M+H]^+$ ($C_{14}H_{14}F_6NO$) requires m/z 326.0974, found m/z 326.0980.

3-benzyl-1,3,4,5-tetrahydro-2*H*-benzo[*d*]azepin-2-one (3k)

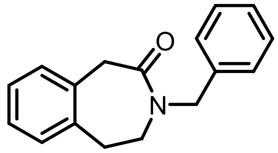

Prepared from 3-benzyl-2,3,5,6-tetrahydrobenzo[*d*]azocin-4(1*H*)-one (**2k**) (79.6 mg, 0.3 mmol) according to General Procedure D to give the desired compound as an off-white solid (39.4 mg, 52%).

^{1}H NMR (400 MHz, $CDCl_3$) δ 7.36 – 7.23 (m, 5H), 7.21 – 7.13 (m, 3H), 7.08 – 7.00 (m, 1H), 4.65 (s, 2H), 3.99 (s, 2H), 3.70 – 3.62 (m, 2H), 3.03 – 2.94 (m, 2H).
^{13}C NMR (101 MHz, $CDCl_3$) δ 172.0, 137.7, 136.1, 131.8, 131.1, 130.3, 128.8, 128.3, 127.6, 127.3, 126.7, 50.0, 46.0, 43.3, 32.4.
Spectral data are consistent with the literature [31].

1-(prop-2-yn-1-yl)piperidin-2-one (3l)

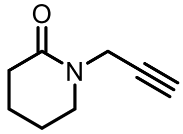

Prepared from 1-(prop-2-yn-1-yl)azepan-2-one (**2l**) (45.4 mg, 0.3 mmol) according to General Procedure D to give the desired product as a yellow oil (22.9 mg, 56%).

^{1}H NMR ($CDCl_3$, 400 MHz) δ 4.23 (d, *J* = 2.4 Hz, 2H), 3.39 (t, *J* = 5.8 Hz, 2H), 2.39 (t, *J* = 6.4 Hz, 2H), 2.18 (t, *J* = 2.6 Hz, 1H), 1.90 – 1.74 (m, 4H).
^{13}C NMR ($CDCl_3$, 126 MHz) δ 169.6, 78.8, 71.8, 47.3, 35.7, 32.4, 23.2, 21.4.
Spectral data are in accordance with the literature [32].

1-dodecylpiperidin-2-one (3m)

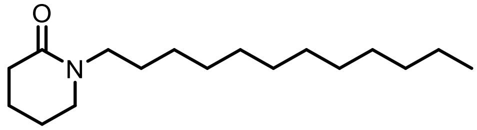

Prepared from 1-dodecylazepan-2-one (0.3 mmol, 1 eq) following General Procedure X to afford the titled compound as a colourless oil (61.7 mg, 77%).

^{1}H NMR (400 MHz, $CDCl_3$) δ 3.33 – 3.25 (m, 2H), 3.24 – 3.18 (m, 2H), 2.37 – 2.28 (m, 2H), 1.79 – 1.68 (m, 4H), 1.53 – 1.43 (m, 2H), 1.29 – 1.15 (m, 18H), 0.87 – 0.78 (m, 3H).
^{13}C NMR (101 MHz, $CDCl_3$) δ 169.5, 47.8, 47.3, 32.4, 31.9, 29.7, 29.7, 29.6, 29.6, 29.5, 29.4, 27.1, 27.0, 23.3, 22.7, 21.5, 14.1.
IR (film) ν_{max}/cm^{-1} 2924, 2853, 1644, 1493, 1466, 1329.
HRMS (ESI+) exact mass calculated for $[M+H]^+$ ($C_{17}H_{34}NO$) requires m/z 268.2635, found m/z 268.2627.

benzyl 3-(2-(2-oxopyrrolidin-1-yl)ethyl)-1H-indole-1-carboxylate (3n)

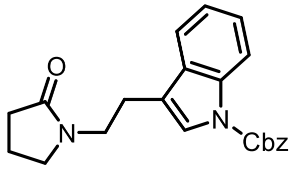

Prepared from **2n** according to General Procedure D to give the desired product as a pale, yellow oil (90.8 mg, 84%).

^{1}H NMR (400 MHz, $CDCl_3$) δ 8.17 (s, 1H), 7.56 (d, *J* = 7.6 Hz, 1H), 7.52 – 7.46 (m, 3H), 7.45 – 7.35 (m, 3H), 7.33 (t, *J* = 7.7 Hz, 1H), 7.29 – 7.22 (m, 1H), 5.43 (s, 2H), 3.60 (t, *J* = 7.4 Hz, 2H), 3.30 (t, *J* = 7.0 Hz, 2H), 2.92 (t, *J* = 7.5 Hz, 2H), 2.36 (t, *J* = 8.1 Hz, 2H), 1.93 (p, *J* = 7.5 Hz, 2H).
^{13}C NMR (101 MHz, $CDCl_3$) δ 175.2, 150.9, 135.8, 135.3, 130.6, 128.9, 128.8, 128.6, 125.0, 123.1, 122.7, 119.0, 118.8, 115.5, 68.7, 47.9, 42.6, 31.1, 23.3, 18.1.
HRMS (ESI+) exact mass calculated for $[M+Na]^+$ ($C_{22}H_{22}N_2O_3Na$) requires m/z 385.1523, found m/z 385.1524.

1-phenylpiperidin-2-one (3o)

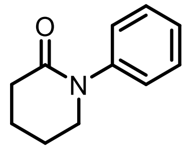

Prepared from 1-phenylazepan-2-one (**2o**) (56.8 mg, 0.3 mmol) according to General Procedure D to give the desired product (38.0 mg, 72%).

^{1}H NMR ($CDCl_3$, 400 MHz) δ 7.41 – 7.35 (m, 2H), 7.24 (m, 3H), 3.67 – 3.58 (m, 2H), 2.60 – 2.51 (m, 2H), 1.99 – 1.88 (m, 4H).
^{13}C NMR ($CDCl_3$, 101 MHz) δ 170.1, 143.5, 129.3, 126.8, 126.3, 51.8, 33.0, 23.7, 21.6.
Spectral data are consistent with the literature [33].

4-(2-oxopiperidin-1-yl)benzonitrile (3p)

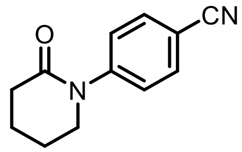

Prepared from 4-(2-oxoazepan-1-yl)benzonitrile (**2o**) (64.2 mg, 0.3 mmol) according to General Procedure D to give the desired product (36.7 mg, 61%).

^{1}H NMR ($CDCl_3$, 400 MHz) δ 7.66 (d, *J* = 8.8 Hz, 2H), 7.42 (d, *J* = 8.8 Hz, 2H), 3.72 – 3.63 (m, 2H), 2.58 (t, *J* = 6.3 Hz, 2H), 2.04 – 1.88 (m, 4H).
^{13}C NMR ($CDCl_3$, 101 MHz) δ 170.4, 147.3, 133.0, 126.3, 118.6, 109.8, 51.0, 33.1, 23.5, 21.3.
Spectral data are consistent with the literature [34].

1-(4-methoxyphenyl)piperidin-2-one (3q)

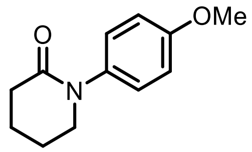

Prepared from 1-(4-methoxyphenyl)azepan-2-one (**2p**) (65.7 mg, 0.3 mmol) according to General Procedure D to give the desired product (37.1 mg, 60%).

^{1}H NMR ($CDCl_3$, 400 MHz) δ 7.15 (d, *J* = 8.9 Hz, 2H), 6.90 (d, *J* = 9.0 Hz, 2H), 3.79 (s, 3H), 3.63 – 3.54 (m, 2H), 2.58 – 2.51 (m, 2H), 1.92 (m, 4H).
^{13}C NMR ($CDCl_3$, 101 MHz) δ 170.4, 158.2, 136.4, 127.5, 114.6, 55.5, 52.1, 32.9, 23.7, 21.6.
Spectral data are consistent with the literature [35].

1-(3-methoxyphenyl)piperidin-2-one (3r)

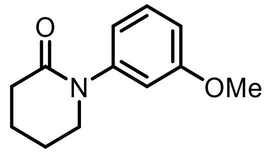

Prepared from 1-(3-methoxyphenyl)azepan-2-one (**2q**) (65.8 mg, 0.3 mmol) according to General Procedure D to give the desired product (37.7 mg, 61%).

^{1}H NMR (400 MHz, $CDCl_3$) δ 7.29 (dd, *J* = 9.0, 7.8 Hz, 1H), 6.86 – 6.78 (m, 3H), 3.79 (s, 3H), 3.67 – 3.56 (m, 2H), 2.60 – 2.50 (m, 2H), 1.99 – 1.87 (m, 4H).
^{13}C NMR ($CDCl_3$, 101 MHz) δ 170.5, 160.3, 144.5, 130.0, 118.6, 112.8, 112.3, 55.4, 51.9, 32.8, 23.6, 21.4.
Spectral data are consistent with the literature [36].

1-(6-(trifluoromethyl)pyridin-3-yl)piperidin-2-one (3s)

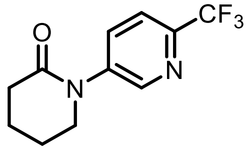

Prepared from 1-(6-(trifluoromethyl)pyridin-3-yl)azepan-2-one (**2r**) (77.5 mg, 0.3 mmol) according to General Procedure D to give the desired product as a colourless oil (35.1 mg, 48%).

^{1}H NMR ($CDCl_3$, 500 MHz) δ 8.68 (d, *J* = 2.4 Hz, 1H), 7.86 (dd, *J* = 8.4, 2.5 Hz, 1H), 7.69 (d, *J* = 8.4 Hz, 1H), 3.76 – 3.71 (m, 2H), 2.60 (t, *J* = 6.6 Hz, 2H), 1.99 (m, 4H).
^{13}C NMR ($CDCl_3$, 126 MHz) δ 170.6, 146.9, 145.2 (q, *J* = 35.0 Hz), 142.1, 134.1, 121.5 (q, *J* = 274.0 Hz), 120.6 (q, *J* = 2.8 Hz), 51.0, 33.0, 23.5, 21.4.
^{19}F NMR ($CDCl_3$, 377 MHz) δ -67.6.
IR (film) ν_{max}/cm^{-1} 2957, 1647, 1589, 1343.
HRMS (ESI+) exact mass calculated for $[M+H]^+$ ($C_{11}H_{12}F_3N_2O$) requires m/z 245.0896, found m/z 245.0892.
mp 98 – 102 °C.

1-(6-methoxypyridin-3-yl)piperidin-2-one (3t)

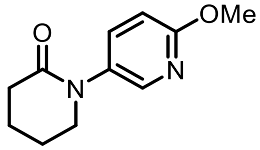

Prepared from 1-(6-methoxypyridin-3-yl)azepan-2-one (**2s**) (66.1 mg, 0.3 mmol) according to General Procedure D to give the desired product as an off-white solid (36.5 mg, 55%).

^{1}H NMR ($CDCl_3$, 400 MHz) δ 8.04 (dd, *J* = 2.7, 0.7 Hz, 1H), 7.48 (dd, *J* = 8.8, 2.7 Hz, 1H), 6.74 (dd, *J* = 8.8, 0.7 Hz, 1H), 3.91 (s, 3H), 3.62 – 3.58 (m, 2H), 2.58 – 2.51 (m, 2H), 2.02 – 1.89 (m, 4H).
^{13}C NMR ($CDCl_3$, 101 MHz) δ 170.5, 162.5, 144.2, 137.4, 133.8, 111.2, 53.8, 52.1, 32.9, 23.6, 21.5.
IR (film) ν_{max}/cm^{-1} 2948, 1652, 1494.
HRMS (ESI+) exact mass calculated for $[M+Na]^+$ ($C_{11}H_{14}N_2O_2Na$) requires m/z 229.0948, found m/z 229.0950.
mp 88 – 90 °C.

1-(quinolin-3-yl)piperidin-2-one (3u)

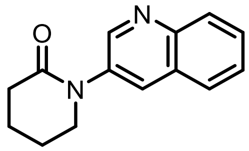

Prepared from 1-(quinolin-3-yl)azepan-2-one (**2t**) (72.1 mg, 0.3 mmol) according to General Procedure D to give the desired product as a brown solid (28.3 mg, 35%).

^{1}H NMR ($CDCl_3$, 400 MHz) δ 8.87 (d, J = 2.4 Hz, 1H), 8.09 (dd, J = 8.5, 1.0 Hz, 1H), 8.00 (dd, J = 2.6, 0.8 Hz, 1H), 7.77 (dd, J = 8.2, 1.4 Hz, 1H), 7.68 (ddd, J = 8.4, 6.9, 1.5 Hz, 1H), 7.53 (ddd, J = 8.2, 6.9, 1.2 Hz, 1H), 3.81 – 3.72 (m, 2H), 2.62 (t, J = 6.3 Hz, 2H), 2.07 – 1.94 (m, 4H).
^{13}C NMR ($CDCl_3$, 101 MHz) δ 170.7, 149.6, 146.4, 136.9, 129.4, 129.3, 128.1, 127.7, 127.1, 51.7, 33.0, 23.6, 21.6.
IR (film) ν_{max}/cm^{-1} 2941, 1638, 1482, 1412.
HRMS (ESI+) exact mass calculated for $[M+H]^+$ ($C_{14}H_{15}N_2O$) requires m/z 227.1179, found m/z 227.1184.
mp 138 – 140 °C.

1-(pyrimidin-5-yl)pyrrolidin-2-one (3v)

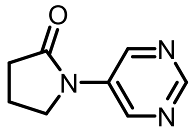

Prepared from 1-(pyrimidin-5-yl)piperidin-2-one (**2u**) (53.2 mg, 0.3 mmol) according to General Procedure D to give the desired product (18.5 mg, 38%).

^{1}H NMR ($CDCl_3$, 400 MHz) δ 9.10 (s, 2H), 8.97 (s, 1H), 3.90 – 3.86 (m, 2H), 2.63 (t, J = 8.1 Hz, 2H), 2.32 – 2.20 (m, 2H).
^{13}C NMR ($CDCl_3$, 101 MHz) δ 175.0, 154.0, 147.0, 134.8, 47.0, 31.9, 18.2.
Spectral data are consistent with the literature [37].

1-(pyridin-2-yl)pyrrolidin-2-one (3w)

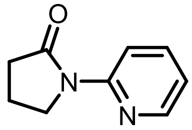

Prepared from 1-(pyridin-2-yl)piperidin-2-one (**2v**) (52.9 mg, 0.3 mmol) according to General Procedure D to give the desired product (19.1 mg, 39%).

^{1}H NMR ($CDCl_3$, 400 MHz) δ 8.39 (dt, J = 8.5, 1.0 Hz, 1H), 8.35 (ddd, J = 5.0, 2.0, 0.9 Hz, 1H), 7.68 (ddd, J = 8.5, 7.3, 2.0 Hz, 1H), 7.02 (ddd, J = 7.3, 4.9, 1.0 Hz, 1H), 4.14 – 4.07 (m ~ t, 2H), 2.70 – 2.62 (m ~ t, 2H), 2.19 – 2.07 (m ~ p, 2H).
^{13}C NMR ($CDCl_3$, 101 MHz) δ 175.1, 152.0, 147.6, 137.6, 119.5, 114.8, 47.4, 33.8, 17.8.
Spectral data are consistent with the literature [38].

diethyl 1-benzyl-2-oxopiperidine-4,4-dicarboxylate (3x)

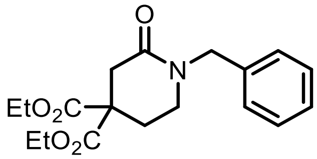

Prepared from diethyl 1-benzyl-7-oxoazepane-4,4-dicarboxylate (**2w**) (104.2 mg, 0.3 mmol) according to General Procedure D to give the desired product as a pale, yellow oil (75.8 mg, 76%).

^{1}H NMR ($CDCl_3$, 400 MHz) δ 7.35 – 7.20 (m, 5H), 4.60 (s, 2H), 4.19 (q, *J* = 7.2 Hz, 4H), 3.22 – 3.14 (m, 2H), 2.91 (s, 2H), 2.29 (t, *J* = 6.3 Hz, 2H), 1.23 (t, *J* = 7.1 Hz, 6H).
^{13}C NMR ($CDCl_3$, 101 MHz) δ 169.9, 166.8, 136.7, 128.7, 128.1, 127.6, 62.1, 53.1, 50.1, 43.5, 37.2, 28.0, 14.0.
IR (film) ν_{max}/cm^{-1} 2982, 1732, 1651, 1496, 1453.
HRMS (ESI+) exact mass calculated for $[M+H]^+$ ($C_{18}H_{24}NO_5$) requires m/z 334.1649, found m/z 334.1659.

8-benzyl-1,4-dioxa-8-azaspiro[4.5]decan-7-one (3y)

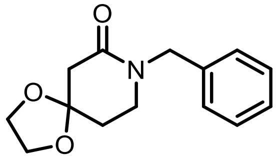

Prepared from 8-benzyl-1,4-dioxa-8-azaspiro[4.6]undecan-9-one (**2x**) (0.3 mmol, 1 eq) following General Procedure D, to afford the titled compound as a pale, yellow oil (65.2 mg, 88%).

^{1}H NMR (400 MHz, $CDCl_3$) δ 7.28 – 7.21 (m, 2H), 7.21 – 7.14 (m, 3H), 4.54 (s, 2H), 3.98 – 3.83 (m, 4H), 3.25 – 3.16 (m ~ t, 2H), 2.63 (s, 2H), 1.91 – 1.78 (m, 2H).
^{13}C NMR (101 MHz, $CDCl_3$) δ 167.8, 136.9, 128.7, 127.9, 127.4, 106.1, 64.7, 49.7, 43.7, 42.6, 32.0.
IR (film) ν_{max}/cm^{-1} 2886, 1643 (s), 1495, 1452.
HRMS (ESI+) exact mass calculated for $[M+Na]^+$ ($C_{14}H_{17}NO_3Na$) requires m/z 270.1101, found m/z 270.1099.

4-benzylmorpholin-3-one (3z)

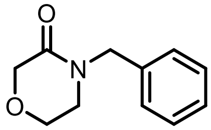

Prepared from 4-benzyl-1,4-oxazepan-5-one (**2y**) (0.3 mmol, 1 eq.) following General Procedure D, to afford the titled compound as a colourless oil (33.2 mg, 58%).

^{1}H NMR (400 MHz, $CDCl_3$) δ 7.36 – 7.30 (m, 2H), 7.32 – 7.24 (m, 1H), 7.29 – 7.22 (m, 2H), 4.61 (s, 2H), 4.23 (s, 2H), 3.88 – 3.76 (m, 2H), 3.29 – 3.20 (m, 2H).
^{13}C NMR (101 MHz, $CDCl_3$) δ 166.9, 136.2, 128.8, 128.3, 127.8, 68.2, 64.0, 49.5, 45.5.
Spectral data are consistent with the literature [28].

1-benzyl-4-(*tert*-butyl)piperidin-2-one (3aa)

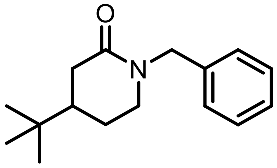

Prepared from 1-benzyl-5-(*tert*-butyl)azepan-2-one (**2z**) (0.3 mmol, 1 eq) following General Procedure D to afford the titled compound as a yellow oil (73.1 mg, 99%).

^{1}H NMR (400 MHz, $CDCl_3$) δ 7.35 – 7.29 (m, 2H), 7.29 – 7.22 (m, 3H), 4.69 (d, *J* = 14.6 Hz, 1H), 4.52 (d, *J* = 14.6 Hz, 1H), 3.24 (ddd, *J* = 12.0, 5.6, 2.2 Hz, 1H), 3.15 (td, *J* = 12.0, 4.2 Hz,

1H), 2.58 (ddd, J = 17.3, 4.9, 2.6 Hz, 1H), 2.20 (dd, J = 17.3, 12.5 Hz, 1H), 1.86 (ddt, J = 13.0, 4.8, 2.4 Hz, 1H), 1.55 (tdd, J = 12.4, 4.9, 2.5 Hz, 1H), 1.46 – 1.31 (m, 1H), 0.88 (s, 9H).
^{13}C NMR (101 MHz, $CDCl_3$) δ 170.6, 137.2, 128.6, 128.1, 127.4, 50.0, 47.2, 43.2, 34.3, 32.0, 26.8, 24.6.
Spectral data are consistent with the literature [39].

5-allyl-1-benzylpyrrolidin-2-one (3ab)

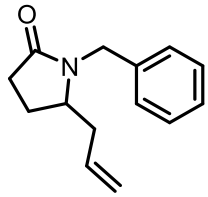

Prepared from 6-allyl-1-benzylpiperidin-2-one (**2ab**) following General Procedure D to afford the titled compound as a colourless oil (53.6 mg, 83%).

^{1}H NMR (400 MHz, $CDCl_3$) δ 7.37 – 7.22 (m, 5H), 5.73 – 5.58 (m, 1H), 5.15 – 5.08 (m, 2H), 5.03 (d, J = 15.1 Hz, 1H), 4.00 (d, J = 15.1 Hz, 1H), 3.52 (tdd, J = 8.1, 4.8, 3.5 Hz, 1H), 2.55 – 2.44 (m, 1H), 2.44 – 2.34 (m, 2H), 2.24 – 2.14 (m, 1H), 2.12 – 2.00 (m, 1H), 1.83 – 1.72 (m, 1H).
^{13}C NMR (101 MHz, $CDCl_3$) δ 175.3, 136.7, 132.7, 132.6, 128.7, 128.0, 127.5, 118.8, 56.3, 44.2, 37.2, 30.1, 23.3.

Spectral data is consistent with the literature [40].

4-(trifluoromethyl)pyrrolidin-2-one (3ac)

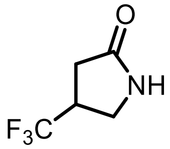

Prepared from 5-(trifluoromethyl)piperidin-2-one (50.1 mg, 0.3 mmol) according to a modification of General Procedure E. A flame-dried, 25 mL round-bottom flask was charged with the solid lactam (0.3 mmol, 1 eq.), $TEMPO^{+}TfO^{-}$ (137.4 mg, 0.45 mmol, 1.5 eq) and a magnetic stir bar. The flask was evacuated and backfilled with nitrogen three times. Anhydrous toluene (0.3 M) was added and the heterogeneous mixture was stirred until the lactam dissolved. TIPSOTf (88.7 µL, 0.33 mmol, 1.1 eq) and pyridine (72.6 µL, 0.9 mmol, 3.0 eq) were added consecutively. The brown/red homogeneous solution was heated to 40 °C for 40 hours.
The flask was cooled to room temperature before a solution of *m*-CPBA (1.5 mmol, 5 eq) in dry, degassed MeCN (0.21 M) was added dropwise over 15 minutes. The resulting orange solution was stirred for a further 2 hours at room temperature. A solution of saturated aqueous $NaHCO_3$ (30 mL) was added and the mixture extracted with CH_2Cl_2 (3 × 30 mL). The combined organic layers were dried with $MgSO_4$ before filtration and concentration *in vacuo*. The red/orange oil was then purified by FCC (eluting with 0 – 40% acetone in pentane) to afford the product as a white solid (13.7 mg, 30%).

^{1}H NMR ($CDCl_3$, 400 MHz) δ 6.70 (s, 1H), 3.61 (t, J = 9.7 Hz, 1H), 3.51 (dd, J = 10.4, 6.0 Hz, 1H), 3.28 – 3.11 (m, 1H), 2.62 – 2.43 (m, 2H).
^{13}C NMR ($CDCl_3$, 101 MHz) δ 175.4, 126.6 (q, J = 277.0 Hz), 41.4 (q, J = 3.4 Hz), 38.2 (q, J = 29.8 Hz), 30.1 (q, J = 2.7 Hz).

^{19}F NMR ($CDCl_3$, 377 MHz) δ -73.1 (d, *J* = 8.7 Hz).
Spectral data are consistent with the literature [41].

ethyl 2-oxopiperidine-4-carboxylate (3ad)

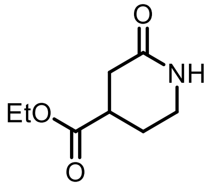

Prepared from ethyl 7-oxoazepane-4-carboxylate (**2ab**) (0.3 mmol, 1 eq) following General Procedure E, to afford the titled compound as an off-white solid (20.0 mg, 39%).

^{1}H NMR (400 MHz, $CDCl_3$) δ 6.85 (s, 1H), 4.17 (q, *J* = 7.1 Hz, 2H), 3.46 – 3.27 (m, 2H), 2.87 – 2.76 (m, 1H), 2.61 – 2.56 (m, 2H), 2.20 – 2.06 (m, 1H), 1.99 – 1.82 (m, 1H), 1.26 (t, *J* = 7.1 Hz, 3H).
^{13}C NMR (101 MHz, $CDCl_3$) δ 173.3, 171.3, 61.2, 40.7, 38.0, 33.3, 25.1, 14.3.
IR (film) ν_{max}/cm^{-1} 2982, 1732, 1651, 1496, 1453.
HRMS (ESI+) exact mass calculated for $[M+Na]^+$ ($C_8H_{13}NO_2Na$) requires m/z 194.0788, found m/z 194.0788.
mp 103 – 105 °C.

4-phenylpiperidin-2-one (3ae)

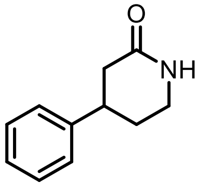

Prepared from 5-phenylazepan-2-one (**2ac**) (0.3 mmol, 1 eq) following General Procedure E, to afford the titled compound as an off-white solid (25.6 mg, 49%).

^{1}H NMR (400 MHz, $CDCl_3$) δ 7.34 (d, *J* = 7.4 Hz, 2H), 7.28 – 7.18 (m, 3H), 7.06 (br s, 1H), 3.51 – 3.30 (m, 2H), 3.10 (tdd, *J* = 11.2, 5.3, 3.1 Hz, 1H), 2.68 (ddd, *J* = 17.6, 5.3, 1.8 Hz, 1H), 2.48 (dd, *J* = 17.6, 11.0 Hz, 1H), 2.15 – 2.01 (m, 1H), 2.01 – 1.83 (m, 1H).
^{13}C NMR (101 MHz, $CDCl_3$) δ 172.3, 143.7, 128.9, 127.0, 126.7, 41.5, 38.9, 38.5, 29.7.
Spectral data are consistent with the literature [42].

1,3,4,5-tetrahydro-2*H*-benzo[*d*]azepin-2-one (3af)

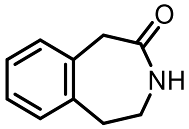

Prepared from 2,3,5,6-tetrahydrobenzo[*d*]azocin-4(1*H*)-one (**1k**) (0.3 mmol, 1 eq) following General Procedure E, to afford the desired product as a white-solid (16.6 mg, 34%).

^{1}H NMR (400 MHz, $CDCl_3$) δ 7.23 – 7.08 (m, 4H), 6.62 (br s, 1H), 3.83 (s, 2H), 3.59 – 3.52 (m, 2H), 3.15 – 3.07 (m ~ t, 2H).
^{13}C NMR (101 MHz, $CDCl_3$) δ 173.9, 137.0, 131.9, 130.5, 130.0, 127.4, 126.9, 42.6, 41.5, 33.4.

Spectral data are consistent with the literature [43].

ethyl 5-oxohexahydroindolizine-8a(1*H*)-carboxylate (4b)

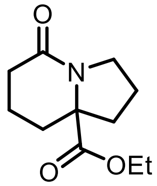

Prepared from diethyl ethyl 5-oxohexahydro-1H-pyrrolo[1,2-a]azepine-9a(5H)-carboxylate (**4a**) (202.8 mg, 0.9 mmol) according to General Procedure D to give the desired product as a brown oil (156.5 mg, 82%).

^{1}H NMR ($CDCl_3$, 400 MHz) δ 3.99 (qd, *J* = 7.1, 2.5 Hz, 2H), 3.55 – 3.43 (m, 1H), 3.37 – 3.27 (m, 1H), 2.36 – 2.29 (m, 1H), 2.29 – 2.19 (m, 2H), 2.17 – 2.03 (m, 1H), 1.77 – 1.63 (m, 2H), 1.62 – 1.53 (m, 1H), 1.52 – 1.43 (m, 1H), 1.41 – 1.25 (m, 2H), 1.06 (t, *J* = 7.1 Hz, 3H).
^{13}C NMR ($CDCl_3$, 101 MHz) δ 173.0, 169.0, 69.3, 61.3, 44.8, 37.7, 31.7, 29.9, 20.1, 18.4, 13.9.
Spectral data are consistent with the literature [44].

ethyl 3-oxotetrahydro-1H-pyrrolizine-7a(5*H*)-carboxylate (4c)

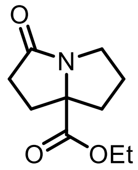

Prepared from ethyl 5-oxohexahydroindolizine-8a(1H)-carboxylate (**4b**) (126.8 mg, 0.6 mmol) according to General Procedure D to give the desired product as an orange oil (60.2 mg, 50%).

^{1}H NMR ($CDCl_3$, 400 MHz) δ 4.18 (q, *J* = 7.1 Hz, 2H), 3.69 – 3.59 (m, 1H), 3.18 – 3.08 (m, 1H), 2.84 – 2.70 (m, 1H), 2.52 (ddd, *J* = 13.1, 8.9, 1.6 Hz, 1H), 2.45 – 2.33 (m, 2H), 2.11 – 1.99 (m, 3H), 1.64 (dt, *J* = 12.7, 9.4 Hz, 1H), 1.25 (t, *J* = 7.1 Hz, 3H).
^{13}C NMR ($CDCl_3$, 101 MHz) δ 175.0, 173.8, 73.6, 61.7, 41.7, 36.1, 34.4, 31.7, 26.1, 14.2.
IR (film) ν_{max}/cm^{-1} 2982, 1734, 1701, 1395.
HRMS (ESI+) exact mass calculated for $[M+H]^+$ ($C_{10}H_{16}NO_3$) requires m/z 198.1125, found m/z 198.1124.

Compound 4e - (ethyl (3a*R*,3b*S*,5a*S*,6*S*,8a*S*,8b*S*,10a*R*)-1-benzyl-3a,5a-dimethyl-2-oxohexadecahydroindeno[5,4-*e*]indole-6-carboxylate)

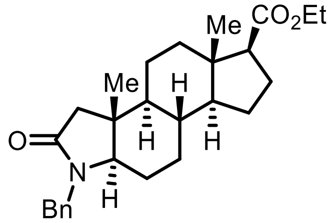

Prepared from **4d** (0.3 mmol, 1 eq) following General Procedure D to afford the product as a colourless oil (54.7 mg, 65%).

^{1}H NMR (400 MHz, $CDCl_3$) δ 7.32 – 7.26 (m, 2H), 7.26 – 7.19 (m, 3H), 4.59 (d, *J* = 15.1 Hz, 1H), 4.25 (d, *J* = 15.2 Hz, 1H), 4.23 – 4.10 (m, 1H), 4.15 – 4.01 (m, 1H), 3.05 (dd, *J* = 12.9, 3.3 Hz, 1H), 2.31 (t, *J* = 9.4 Hz, 1H), 2.21 (d, *J* = 15.2 Hz, 1H), 2.10 (d, *J* = 15.3 Hz, 1H), 2.17 – 2.03 (m, 1H), 1.99 (dt, *J* = 12.8, 3.3 Hz, 1H), 1.82 – 1.66 (m, 3H), 1.66 – 1.57 (m, 1H), 1.52 – 1.32 (m, 4H), 1.24 (t, *J* = 7.2 Hz, 3H), 1.32 – 1.14 (m, 2H), 1.13 – 1.04 (m, 2H), 0.94 (s, 3H), 0.96 – 0.84 (m, 1H), 0.64 (s, 3H).
^{13}C NMR (101 MHz, $CDCl_3$) δ 176.7, 174.0, 137.7, 128.6, 127.9, 127.4, 67.8, 60.1, 55.4, 55.2, 51.2, 46.3, 44.7, 44.4, 41.9, 38.2, 35.8, 29.7, 24.4, 23.5, 23.2, 22.8, 15.1, 14.6, 13.8.
IR (film) ν_{max}/cm^{-1} 2937, 1727, 1695, 1454.
HRMS (ESI+) exact mass calculated for $[M+H]^+$ ($C_{27}H_{38}NO_3$) requires m/z 424.2846, found m/z 424.2850.

ethyl 2-(4-((1-benzyl-5-oxopyrrolidin-2-yl)methyl)phenyl)propanoate (4g)

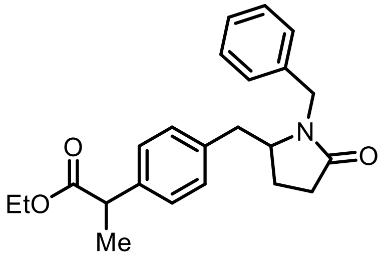

Prepared from **4f** (0.23 mmol, 1 eq) following General Procedure D to afford the product as a colourless oil (72.6 mg, 86%).

^{1}H NMR (400 MHz, $CDCl_3$) δ 7.38 – 7.25 (m, 3H), 7.25 – 7.19 (m ~ d, 4H), 7.02 (d, *J* = 8.2 Hz, 2H), 5.10 (d, *J* = 14.9 Hz, 1H), 4.19 – 4.06 (m, 2H), 4.01 (d, *J* = 15.0 Hz, 1H), 3.73 – 3.60 (m, 2H), 2.99 (dd, *J* = 14.4, 3.5 Hz, 1H), 2.56 (dd, *J* = 13.4, 10.6 Hz, 1H), 2.35 – 2.24 (m, 2H), 1.99 – 1.87 (m, 1H), 1.81 – 1.70 (m, 1H), 1.48 (d, *J* = 7.2 Hz, 3H), 1.19 (t, *J* = 7.1 Hz, 3H).
^{13}C NMR (101 MHz, $CDCl_3$) δ 175.3, 174.5, 139.3, 136.7, 135.9, 129.5, 128.8, 128.1, 127.8, 127.6, 60.8, 58.0, 45.2, 44.5, 38.8, 29.9, 23.8, 18.6, 14.2.
IR (film) ν_{max}/cm^{-1} 2980, 1729, 1686, 1445, 1420.
HRMS (ESI+) exact mass calculated for $[M+K]^+$ ($C_{23}H_{27}NO_3K$) requires m/z 404.1623, found m/z 404.1634.

1-benzyl-5-(3-(cyclopentyloxy)-4-methoxyphenyl)piperidin-2-one (4j)

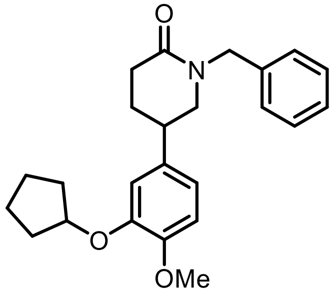

Prepared from 1-benzyl-6-(3-(cyclopentyloxy)-4-methoxyphenyl)azepan-2-one (**4i**) (0.49 mmol, 1 eq) according to General Procedure D to afford the desired product as an off-white solid (140 mg, 75%).

^{1}H NMR (400 MHz, $CDCl_3$) δ 7.35 – 7.25 (m, 5H), 6.78 (d, *J* = 8.8 Hz, 1H), 6.72 – 6.63 (m, 2H), 4.77 – 4.67 (m, 2H), 4.53 (d, *J* = 14.6 Hz, 1H), 3.80 (s, 3H), 3.35 (ddd, *J* = 12.2, 5.4, 1.7 Hz, 1H), 3.23 (dd, *J* = 12.1, 10.7 Hz, 1H), 2.97 (tdd, *J* = 11.0, 5.4, 3.5 Hz, 1H), 2.67 (ddd, *J* = 17.8, 5.7, 3.1 Hz, 1H), 2.57 (ddd, *J* = 17.8, 11.2, 6.4 Hz, 1H), 2.13 – 1.92 (m, 2H), 1.92 – 1.76 (m, 6H), 1.68 – 1.51 (m, 2H).
^{13}C NMR (101 MHz, $CDCl_3$) δ 169.5, 149.3, 147.8, 137.2, 134.4, 128.7, 128.4, 127.6, 119.0, 114.4, 112.2, 80.7, 56.2, 54.0, 50.4, 39.9, 32.9, 32.9, 32.2, 28.4, 24.1.
IR (film) vmax/cm^{-1} 2951, 1640, 1515, 1453, 1442, 1260.
HRMS (ESI+) exact mass calculated for $[M+H]^+$ ($C_{24}H_{30}NO_3$) requires m/z 380.2220, found m/z 380.2219.
mp 110 – 112 °C

1-benzyl-4-(3-(cyclopentyloxy)-4-methoxyphenyl)pyrrolidin-2-one (4k)

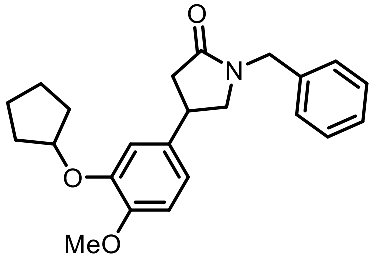

Prepared from 1-benzyl-5-(3-(cyclopentyloxy)-4-methoxyphenyl)piperidin-2-one (**4j**) (0.25 mmol, 1 eq) according to General Procedure D to afford the desired compound as a yellow oil (80.0 mg, 88%).

^{1}H NMR (400 MHz, $CDCl_3$) δ 7.36 – 7.30 (m, 2H), 7.30 – 7.25 (m, 3H), 6.78 (d, *J* = 8.1 Hz, 1H), 6.68 (dd, *J* = 8.2, 2.2 Hz, 1H), 6.66 (d, *J* = 2.1 Hz, 1H), 4.73 – 4.65 (m, 1H), 4.56 (d, *J* = 14.6 Hz, 1H), 4.46 (d, *J* = 14.6 Hz, 1H), 3.80 (s, 3H), 3.61 (dd, *J* = 9.6, 8.3 Hz, 1H), 3.53 – 3.42 (m ~ p, 1H), 3.25 (dd, *J* = 9.6, 7.0 Hz, 1H), 2.87 (dd, *J* = 16.9, 9.0 Hz, 1H), 2.60 (dd, *J* = 16.9, 8.3 Hz, 1H), 1.95 – 1.74 (m, 6H), 1.67 – 1.53 (m, 2H).
^{13}C NMR (101 MHz, $CDCl_3$) δ 173.9, 149.2, 147.9, 136.4, 134.9, 128.8, 128.3, 127.7, 118.8, 113.7, 112.2, 80.6, 56.2, 54.2, 46.7, 39.2, 36.7, 32.9, 32.8, 24.1.
HRMS (ESI+) exact mass calculated for $[M+Na]^+$ ($C_{23}H_{27}NO_3Na$) requires m/z 388.1883, found m/z 388.1891.

Spectral data is consistent with the literature. [45]

1-benzyl-3-(3-(cyclopentyloxy)-4-methoxyphenyl)piperidin-2-one (4m)

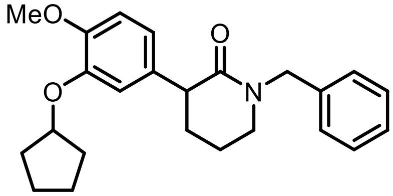

Prepared from 1-benzyl-4-(3-(cyclopentyloxy)-4-methoxyphenyl)azepan-2-one (**4l**) (0.3 mmol, 1 eq) according to General Procedure D to give the desired product as a pale, yellow oil (61.8 mg, 54%).

^{1}H NMR (400 MHz, $CDCl_3$) δ 7.38 – 7.26 (m, 5H), 6.81 (d, *J* = 8.1 Hz, 1H), 6.78 – 6.70 (m, 2H), 4.92 (d, *J* = 14.4 Hz, 1H), 4.76 – 4.65 (m, 1H), 4.42 (d, *J* = 14.4 Hz, 1H), 3.81 (s, 3H), 3.71 (dd, *J* = 7.4, 6.0 Hz, 1H), 3.37 (ddd, *J* = 12.4, 7.2, 5.4 Hz, 1H), 3.32 – 3.25 (m, 1H), 2.21 – 2.09 (m, 1H), 1.98 – 1.69 (m, 9H), 1.66 – 1.50 (m, 2H).
^{13}C NMR (101 MHz, $CDCl_3$) δ 170.9, 148.8, 147.6, 137.6, 134.3, 128.6, 128.5, 127.5, 120.3, 115.5, 112.2, 80.4, 56.2, 50.7, 48.1, 47.6, 32.9, 32.8, 30.5, 24.1, 24.1, 20.7.
IR (film) $\nu max/cm^{-1}$ 2947, 1708, 1513, 1493, 1442, 1257.
HRMS (ESI+) exact mass calculated for $[M+H]^+$ ($C_{24}H_{29}NO_3Na$) requires m/z 402.2040, found m/z 402.2052.

Linear amide products

***N*-benzyl-3-(phenylamino)propenamide (5e)**

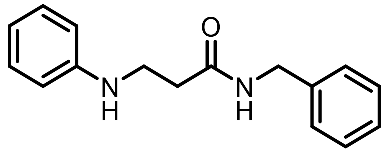

Prepared following General Procedure F from 1-benzyl-3-((2,2,6,6-tetramethylpiperidin-1-yl)oxy)pyrrolidin-2-one (**5d**) (0.1 mmol, 1 eq) and benzylamine (0.5 mmol, 5 eq) to afford the titled compound as a brown oil (18.7 mg, 74%).

^{1}H NMR (500 MHz, $CDCl_3$) δ 7.34 (t, *J* = 7.1 Hz, 2H), 7.31 – 7.24 (m, 3H), 7.20 (dd, *J* = 8.6, 7.4 Hz, 2H), 6.76 (t, *J* = 7.3 Hz, 1H), 6.66 (d, *J* = 7.5 Hz, 2H), 6.19 (brs, 1H), 4.43 (d, *J* = 5.7 Hz, 2H), 3.85 (brs, 1H), 3.53 – 3.46 (m, 2H), 2.52 (t, *J* = 6.2 Hz, 2H).
^{13}C NMR (126 MHz, $CDCl_3$) δ 171.57, 147.46, 138.23, 129.45, 128.82, 127.82, 127.63, 118.23, 113.58, 43.65, 40.48, 35.64.
IR (film) ν_{max}/cm^{-1} 3294, 2928, 1644, 1603, 1506.
HRMS (ESI+) exact mass calculated for $[M+H]^+$ ($C_{16}H_{19}N_2O$) requires m/z 255.1492, found m/z 255.1486.

***N*-(((1R,4aS,10a*R*)-7-isopropyl-1,4a-dimethyl-1,2,3,4,4a,9,10,10a-octahydrophenanthren-1-yl)methyl)-3-(phenylamino)propenamide (5f)**

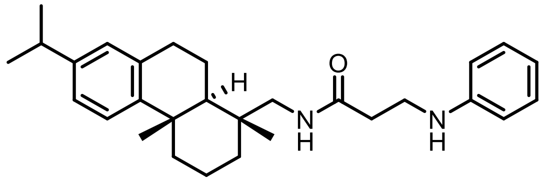

Prepared following General Procedure F from 1-benzyl-3-((2,2,6,6-tetramethylpiperidin-1-yl)oxy)pyrrolidin-2-one (**5d**) (0.1 mmol, 1 eq) and dehydroabietylamine (0.5 mmol, 5 eq) to afford the titled compound as a yellow oil (26.0 mg, 60%).

^{1}H NMR (400 MHz, $CDCl_3$) δ 7.17 (d, *J* = 8.3 Hz, 1H), 7.12 (dd, *J* = 8.7, 7.3 Hz, 2H), 7.01 (dd, *J* = 8.2, 2.4 Hz, 1H), 6.88 (d, *J* = 2.4 Hz, 1H), 6.70 (t, *J* = 7.3 Hz, 1H), 6.53 (dd, *J* = 8.7, 1.1 Hz, 2H), 5.80 (s, 1H), 4.05 (brs, 1H), 3.44 (t, *J* = 5.9 Hz, 2H), 3.26 (dd, *J* = 13.7, 6.4 Hz, 1H), 3.07 (dd, *J* = 13.7, 6.4 Hz, 1H), 2.95 – 2.70 (m, 3H), 2.50 – 2.42 (m, 2H), 2.27 (d, *J* = 13.0 Hz, 1H), 1.92 – 1.83 (m, 1H), 1.79 – 1.67 (m, 2H), 1.67 – 1.58 (m, 1H), 1.42 – 1.33 (m, 2H), 1.33 – 1.26 (m, 1H), 1.24 – 1.19 (m, 1H), 1.23 (d, *J* = 7.0 Hz, 6H), 1.21 (s, 3H), 0.92 (s, 3H).

^{13}C NMR (101 MHz, $CDCl_3$) δ 171.8, 147.6, 147.3, 145.8, 134.8, 129.4, 127.1, 124.3, 124.0, 118.0, 113.4, 49.8, 45.3, 40.4, 38.4, 37.5, 37.4, 36.3, 36.0, 33.6, 30.3, 25.4, 24.1, 19.1, 18.9, 18.7.
IR (film) ν_{max}/cm^{-1} 3317, 2927, 1643, 1603, 1504.
HRMS (ESI+) exact mass calculated for $[M+H]^+$ ($C_{29}H_{41}N_2O$) requires m/z 433.3213, found m/z 433.3218.

1-morpholino-3-(phenylamino)propan-1-one (5g)

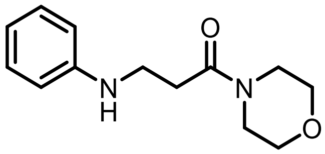

Prepared following General Procedure F from 1-benzyl-3-((2,2,6,6-tetramethylpiperidin-1-yl)oxy)pyrrolidin-2-one (**5d**) (0.1 mmol, 1 eq) and morpholine (0.5 mmol, 5 eq) to afford the titled compound as a yellow solid (19.1 mg, 82%).

^{1}H NMR (400 MHz, $CDCl_3$) δ 7.18 (dd, *J* = 8.6, 7.3 Hz, 2H), 6.77 – 6.69 (t, *J* = 7.3 Hz, 1H), 6.66 (dd, *J* = 8.6, 1.1 Hz, 2H), 4.14 (brs, 1H), 3.71 – 3.55 (m, 6H), 3.50 (t, *J* = 6.0 Hz, 2H), 3.44 – 3.34 (m, 2H), 2.60 (t, *J* = 6.0 Hz, 2H).
^{13}C NMR (101 MHz, $CDCl_3$) δ 170.4, 147.4, 129.5, 118.0, 113.5, 66.9, 66.6, 45.9, 41.9, 39.8, 31.9.
IR (film) ν_{max}/cm^{-1} 3367, 2858, 1632, 1602, 1504, 1435, 1230, 1114.
HRMS (ESI+) exact mass calculated for $[M+H]^+$ ($C_{13}H_{19}N_2O_2$) requires m/z 234.1441, found m/z 235.1436.
mp 76 – 78 °C

***tert*-butyl 3-(3-(phenylamino)propanamido)azetidine-1-carboxylate (5h)**

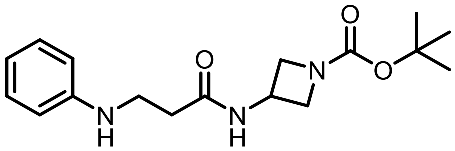

Prepared following General Procedure F from 1-benzyl-3-((2,2,6,6-tetramethylpiperidin-1-yl)oxy)pyrrolidin-2-one (**5d**) (0.1 mmol, 1 eq) and *tert*-butyl 3-aminoazetidine-1-carboxylate (0.5 mmol, 5 eq) to afford the titled compound as a yellow oil (29.9 mg, 94%).

^{1}H NMR (400 MHz, $CDCl_3$) δ 7.21 – 7.12 (m ~ t, 2H), 6.72 (t, *J* = 7.3 Hz, 1H), 6.62 (d, *J* = 7.6 Hz, 2H), 4.57 (qt, *J* = 7.5, 5.1 Hz, 1H), 4.18 (dd, *J* = 9.3, 7.7 Hz, 2H), 3.67 (dd, *J* = 9.3, 5.2 Hz, 2H), 3.43 (t, *J* = 6.1 Hz, 2H), 2.47 (t, *J* = 6.1 Hz, 2H), 1.41 (s, 9H).
^{13}C NMR (101 MHz, $CDCl_3$) δ 171.8, 156.2, 147.7, 129.4, 118.0, 113.3, 80.0, 56.3, 40.1, 39.3, 35.5, 28.4.
IR (film) ν_{max}/cm^{-1} 3305, 2977, 1679, 1657, 1603, 1545, 1506, 1415, 1151.
HRMS (ESI+) exact mass calculated for $[M+H]^+$ ($C_{17}H_{26}N_3O_3$) requires m/z 320.1969, found m/z 320.1968.

N-(3,4-dimethoxyphenethyl)-3-(phenylamino)propanamide (5i)

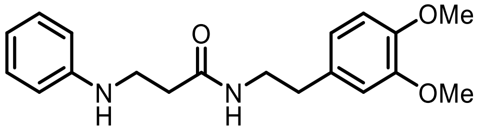

Prepared following General Procedure F from 1-benzyl-3-((2,2,6,6-tetramethylpiperidin-1-yl)oxy)pyrrolidin-2-one (**5d**) (0.1 mmol, 1 eq) and 2-(3,4-dimethoxyphenyl)ethan-1-amine (0.5 mmol, 5 eq) to afford the titled compound as a brown oil (28.7 mg, 88%).

^{1}H NMR (400 MHz, $CDCl_3$) δ 7.17 (dd, *J* = 8.5, 7.3 Hz, 2H), 6.76 – 6.70 (m, 2H), 6.69 – 6.63 (m, 2H), 6.60 (d, *J* = 8.7, 1.1 Hz, 2H), 5.87 (brs, 1H), 3.83 (s, 3H), 3.82 (s, 3H), 3.48 (q, *J* = 6.5, 2H), 3.40 (t, *J* = 6.0, 1H), 2.73 (t, *J* = 7.0 Hz, 2H), 2.42 (t, *J* = 6.1 Hz, 2H).
^{13}C NMR (101 MHz, $CDCl_3$) δ 171.6, 149.1, 147.8, 147.5, 131.3, 129.4, 120.7, 118.1, 113.5, 111.9, 111.4, 56.0, 55.9, 40.7, 40.4, 35.7, 35.2.
IR (film) ν_{max}/cm^{-1} 3363, 2936, 1645, 1603, 1514, 1261, 1236.
HRMS (ESI+) exact mass calculated for $[M+H]^+$ ($C_{19}H_{25}N_2O_3$) requires m/z 329.1860, found m/z 329.1849.

methyl 3-(benzylamino)propanoate (S1-10)

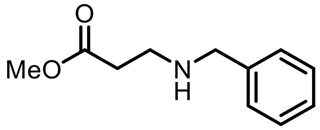

To an oven-dried 25 mL vial, was added 1-benzyl-3-((2,2,6,6-tetramethylpiperidin-1-yl)oxy)pyrrolidin-2-one (**5a**) (0.3 mmol, 1 eq), a magnetic stir bar, and THF (0.3 M) to afford a colourless solution. Purified *m*-CPBA (0.9 mmol, 3 eq) was added to the stirred reaction mixture, which rapidly turned red. The vessel was sealed and heated to 40 °C for 1 h. Dry, MeOH (3 mL) was added to the reaction vessel, which was resealed and heated to 60 °C for 18 h. Following cooling to ambient conditions, the volatiles were removed using a rotary evaporator before dilution with CH_2Cl_2 and addition to saturated aqueous $NaHCO_3$. The mixture was extracted with CH_2Cl_2 (3 × 30 mL) and the combined organic layers were dried over Na_2SO_4 before concentration *in vacuo*. The crude material was purified using FCC (eluting with 0-5% MeOH in EtOAc) to afford the titled compound as a colourless oil (30.6 mg, 53%).

^{1}H NMR (400 MHz, $CDCl_3$) δ 7.28 – 7.20 (m, 4H), 7.21 – 7.13 (m, 1H), 3.75 (s, 2H), 3.59 (s, 3H), 3.50 (br s, 1H), 2.85 (t, *J* = 6.6 Hz, 2H), 2.51 (t, *J* = 6.6 Hz, 2H).
^{13}C NMR (101 MHz, $CDCl_3$) δ 173.0, 138.9, 128.6, 128.4, 127.3, 53.4, 51.7, 44.1, 34.1.
Spectral data are consistent with the literature [46].

methyl 3-(benzylamino)-2,2-dimethylpropanoate (5l)

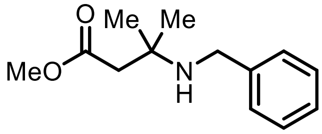

To an oven-dried 25 mL vial, was added 1-benzyl-5,5-dimethyl-3-((2,2,6,6-tetramethylpiperidin-1-yl)oxy)pyrrolidin-2-one (**5k**) (0.56 mmol, 1 eq), a magnetic stir bar, and THF (0.3 M) to afford a colourless solution. Purified *m*-CPBA (1.7 mmol, 3 eq) was added to the stirred reaction mixture, which rapidly turned red. The vessel was sealed and heated to 40 °C for 1 h. Dry, MeOH (5.6 mL)

was added to the reaction vessel, which was resealed and heated to 60 °C for 18 h. Following cooling to ambient conditions, the volatiles were removed using a rotary evaporator before dilution with CH_2Cl_2 and addition to saturated aqueous $NaHCO_3$. The mixture was extracted with CH_2Cl_2 (3 × 30 mL) and the combined organic layers were dried over Na_2SO_4 before concentration *in vacuo*. The crude material was purified using FCC (eluting with 0-5% MeOH in EtOAc) to afford the titled compound as a red oil (56 mg, 45%).

^{1}H NMR (400 MHz, $CDCl_3$) δ 7.38 – 7.33 (m, 2H), 7.33 – 7.28 (m, 2H), 7.25 – 7.20 (m, 1H), 3.72 (s, 2H), 3.68 (s, 3H), 2.53 (s, 2H), 1.24 (s, 6H).
^{13}C NMR (101 MHz, $CDCl_3$) δ 172.5, 141.1, 128.5, 128.4, 126.9, 52.7, 51.5, 47.0, 44.2, 27.7.
IR (film) ν_{max}/cm^{-1} 2969, 1732, 1453, 1438, 1230.
HRMS (ESI+) exact mass calculated for $[M+Na]^+$ ($C_{13}H_{19}NO_2Na$) requires m/z 244.1308, found m/z 244.1306.

1-benzyl-4,4-dimethylazetidin-2-one (5m)

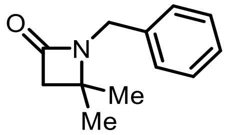

Following a modified literature procedure[47], to an oven-dried 10 mL vial containing a stir bar was added methyl 3-(benzylamino)-3-methylbutanoate (**5l**) (52 mg, 0.23 mmol, 1 eq). The vessel was sealed subjected to three cycles of evacuation and backfilling with N_2 gas, before Et_2O (0.2 M) was added. The contents were cooled to 0 °C and with stirring, MeMgBr (3 M in THF) (80 µL, 0.25 mmol, 1.1 eq) was added dropwise. The mixture was stirred at 0 °C for 45 min. Saturated aqueous NH_4Cl was added to quench the reaction, which was subsequently extracted with Et_2O (3 × 15 mL). The combined organic layers were dried over Na_2SO_4, filtered and concentrated under reduced pressure. The crude material was purified using FCC (eluting with 40% EtOAc in pentane) to afford the desired product as a yellow oil (12.6 mg, 29%).

^{1}H NMR (400 MHz, $CDCl_3$) δ 7.38 – 7.23 (m, 5H), 4.30 (s, 2H), 2.76 (s, 2H), 1.22 (s, 6H).
^{13}C NMR (101 MHz, $CDCl_3$) δ 166.5, 137.1, 128.7, 128.4, 127.7, 56.3, 50.8, 43.4, 25.3.
Spectral data are consistent with the literature[48].

NMR Spectra

Compound 2d (^{1}H)

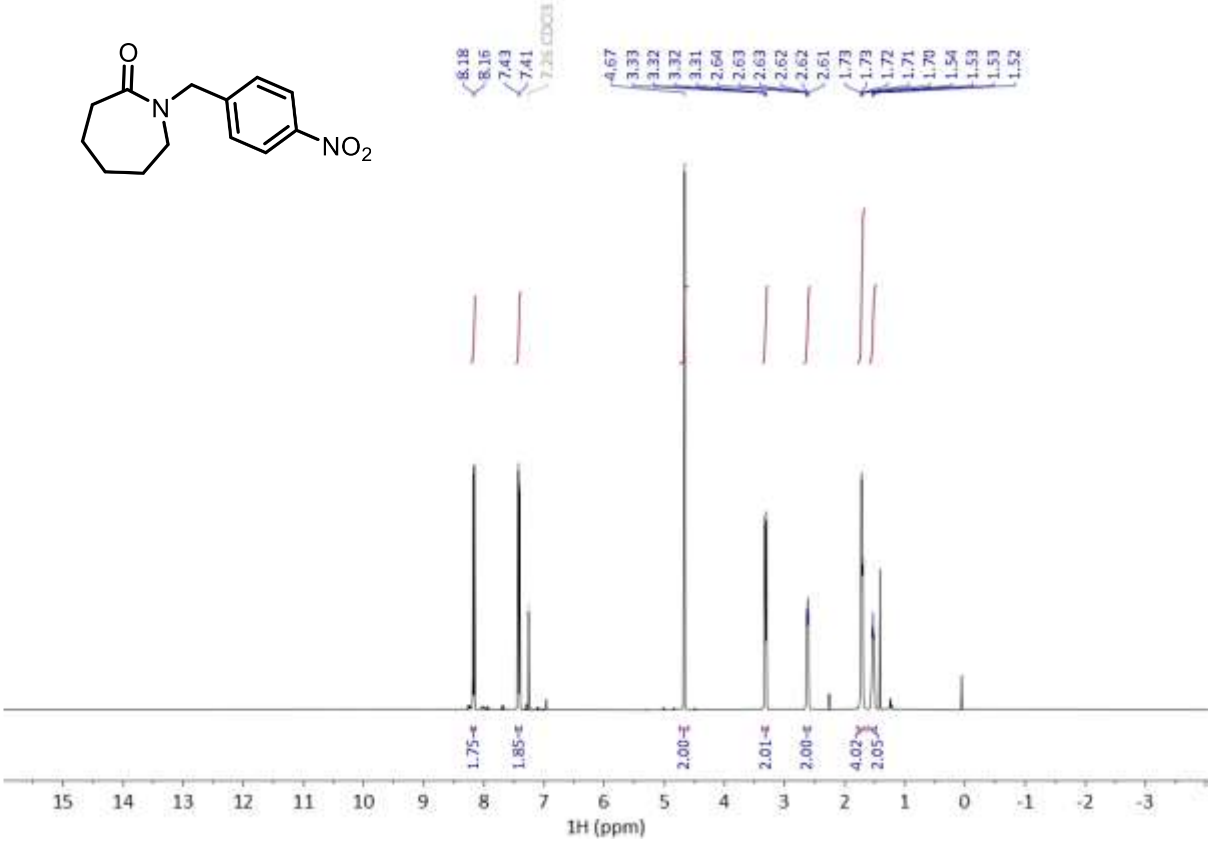

Compound 2d (^{13}C)

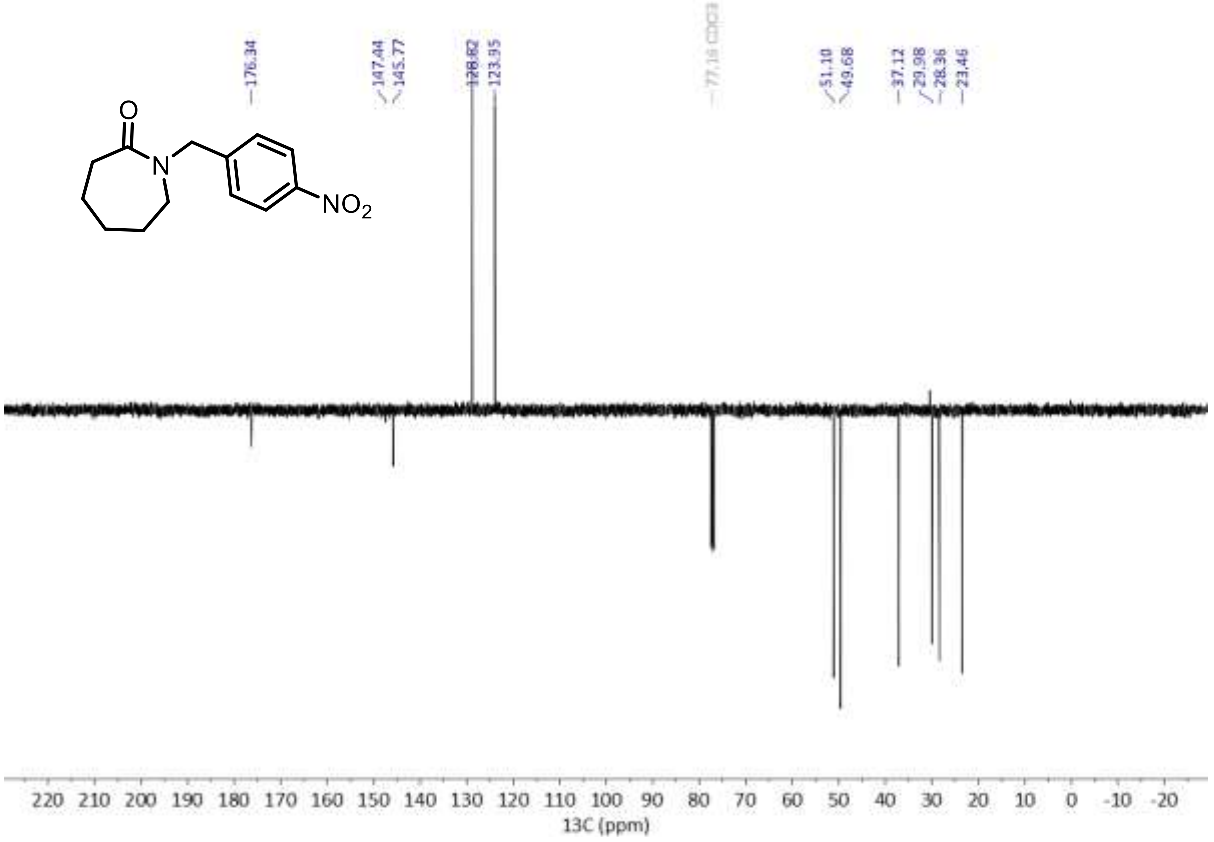

Compound 2i (^{1}H)

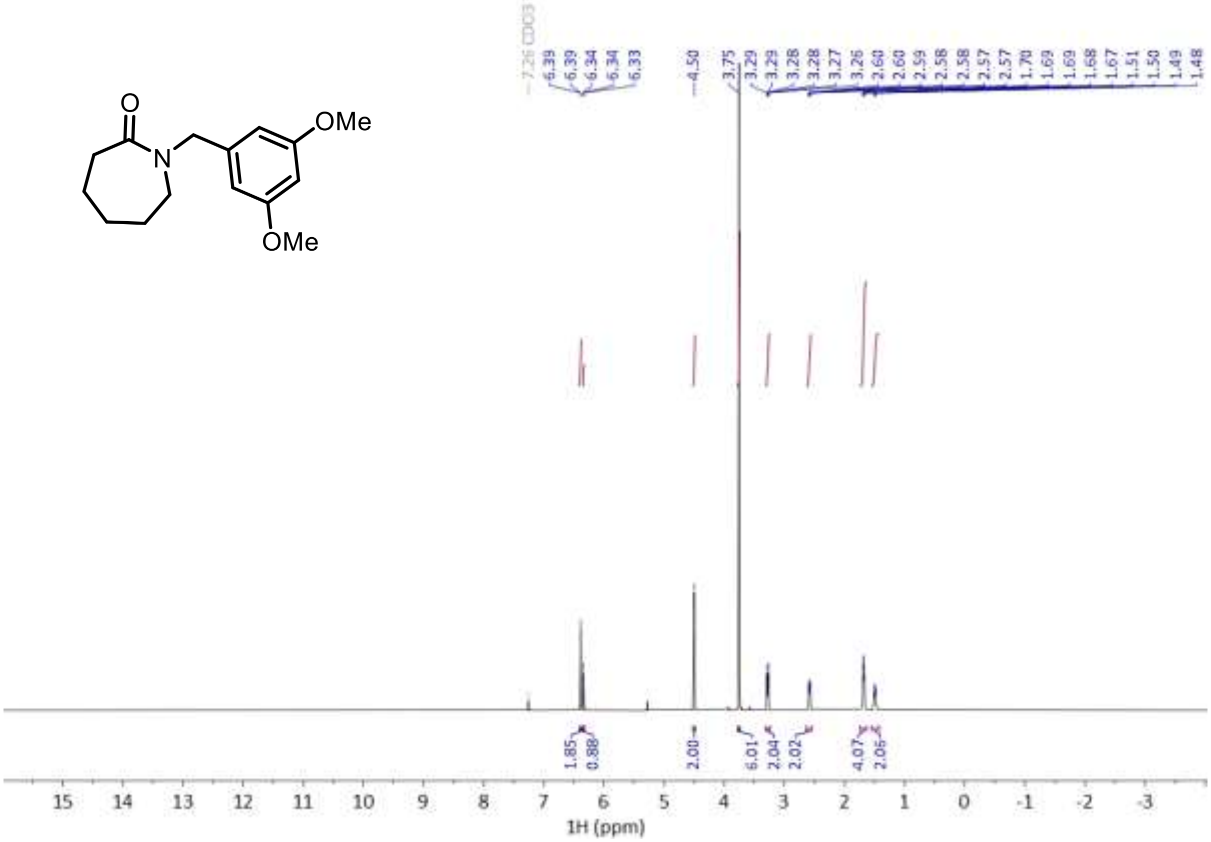

Compound 2i (^{13}C)

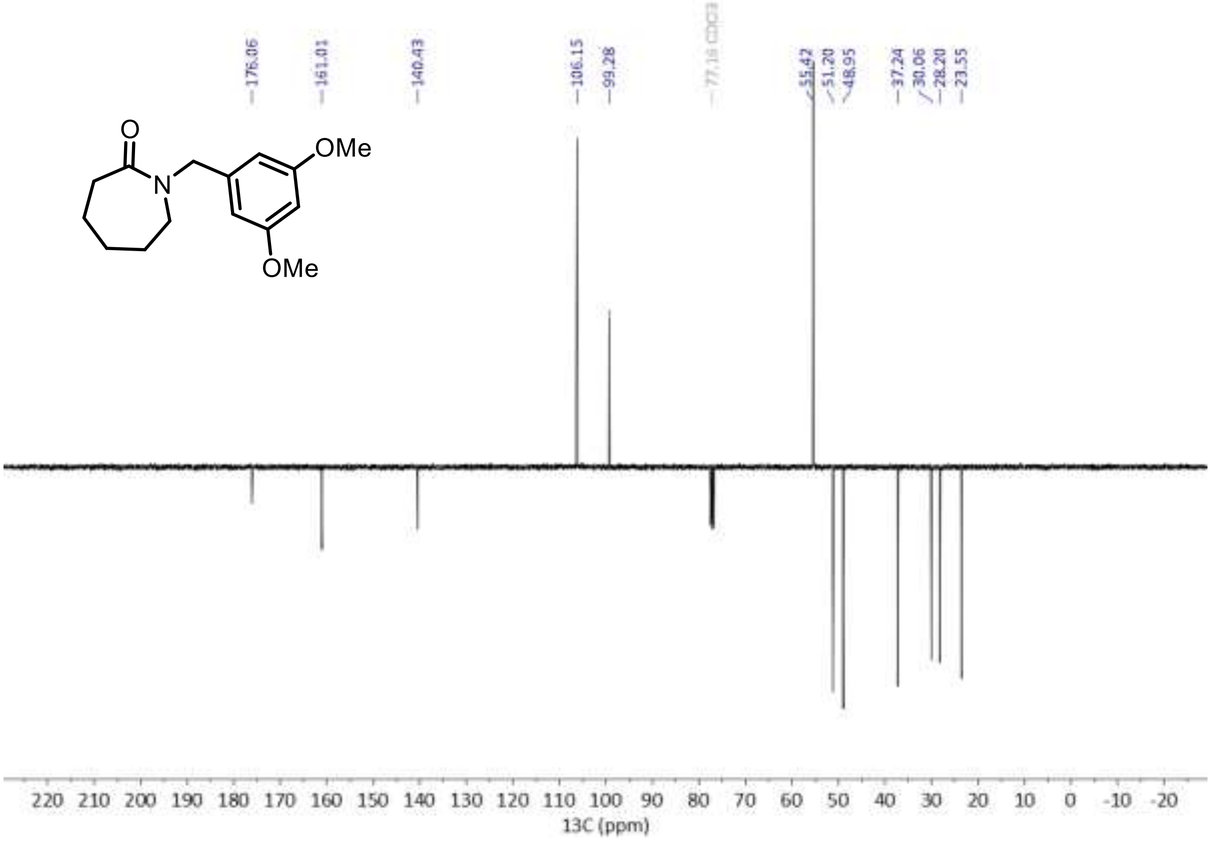

Compound 2j (^{1}H)

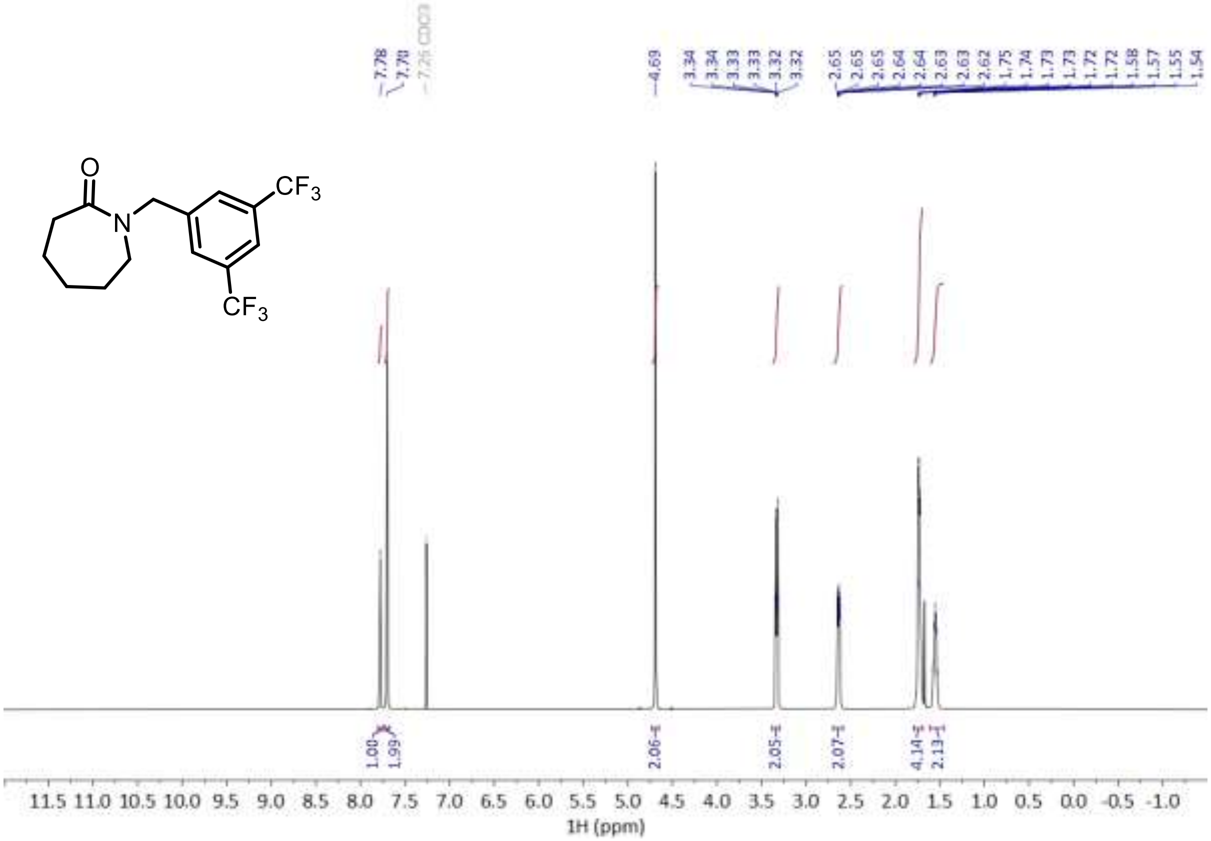

Compound 2j (^{13}C)

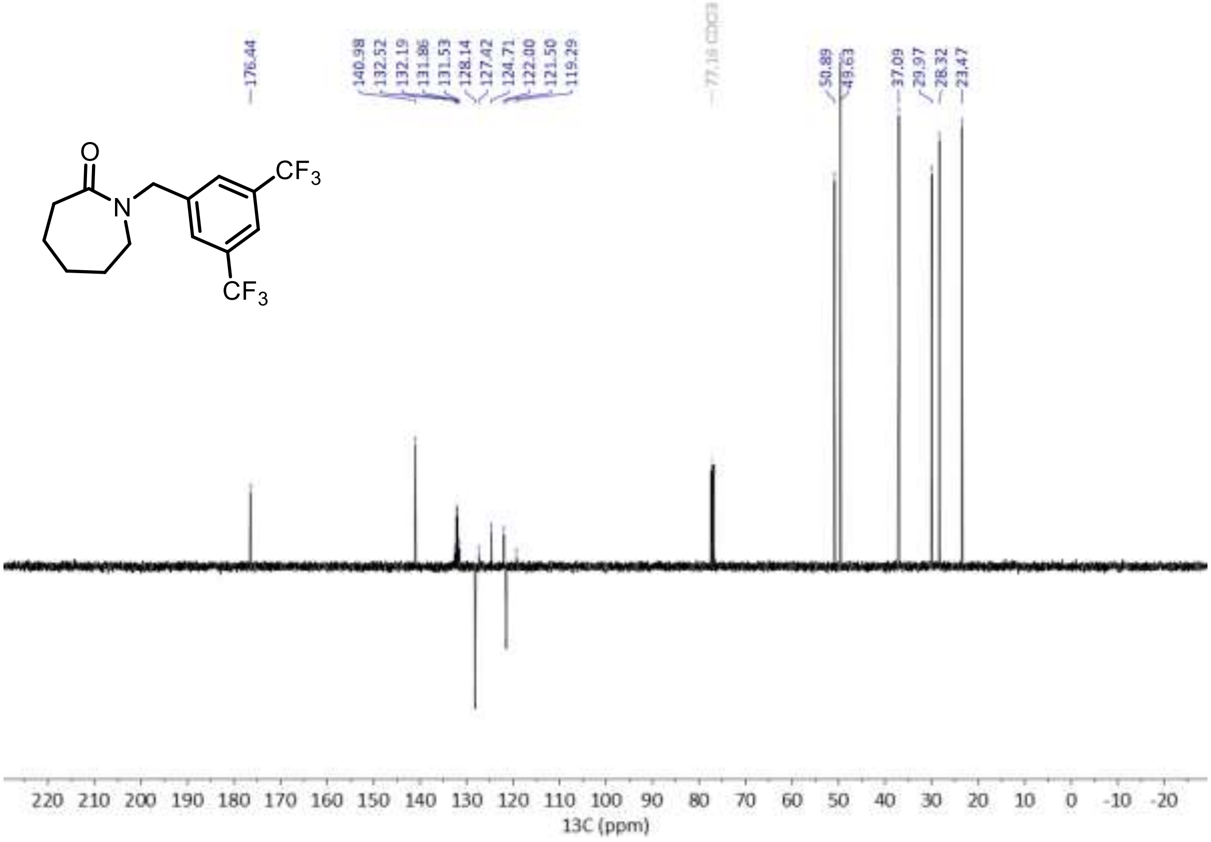

Compound 2j (^{19}F)

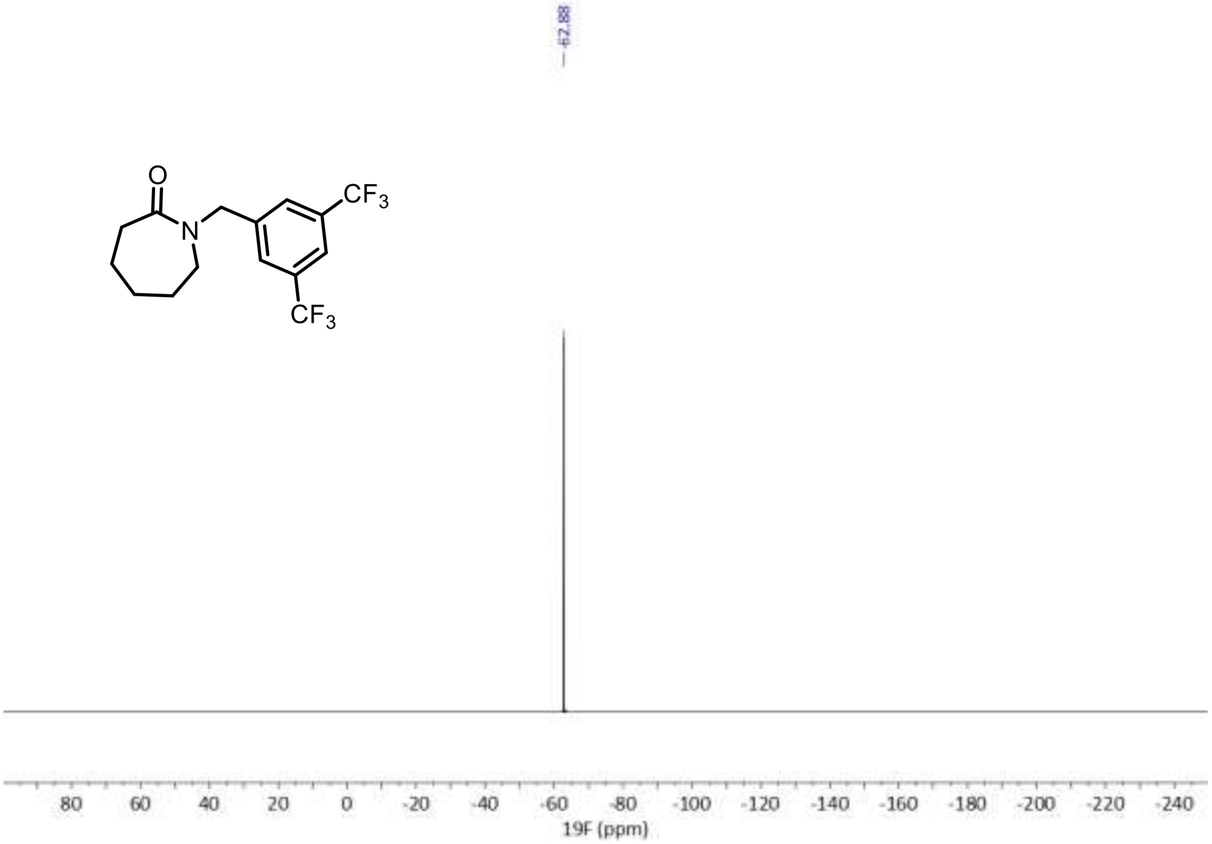

Compound 1k (^{1}H)

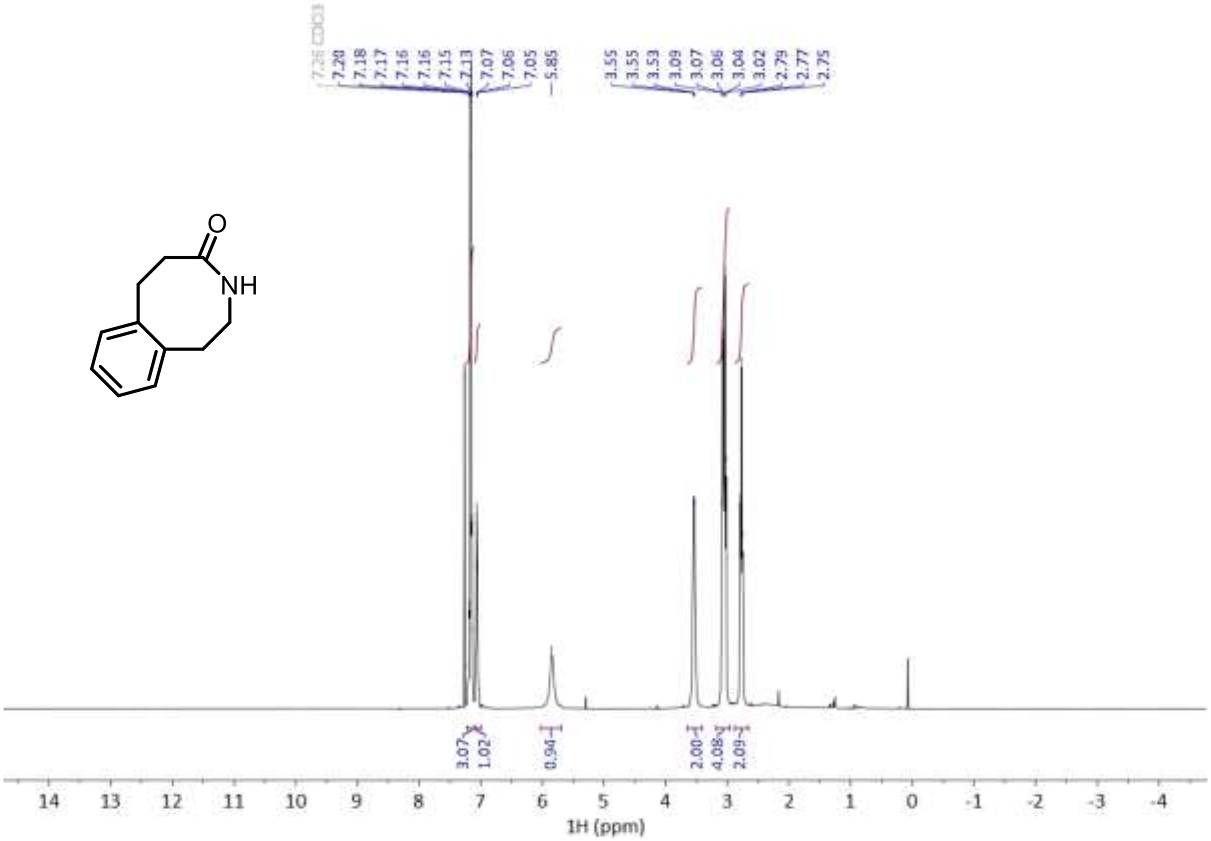

Compound 1k (^{13}C)

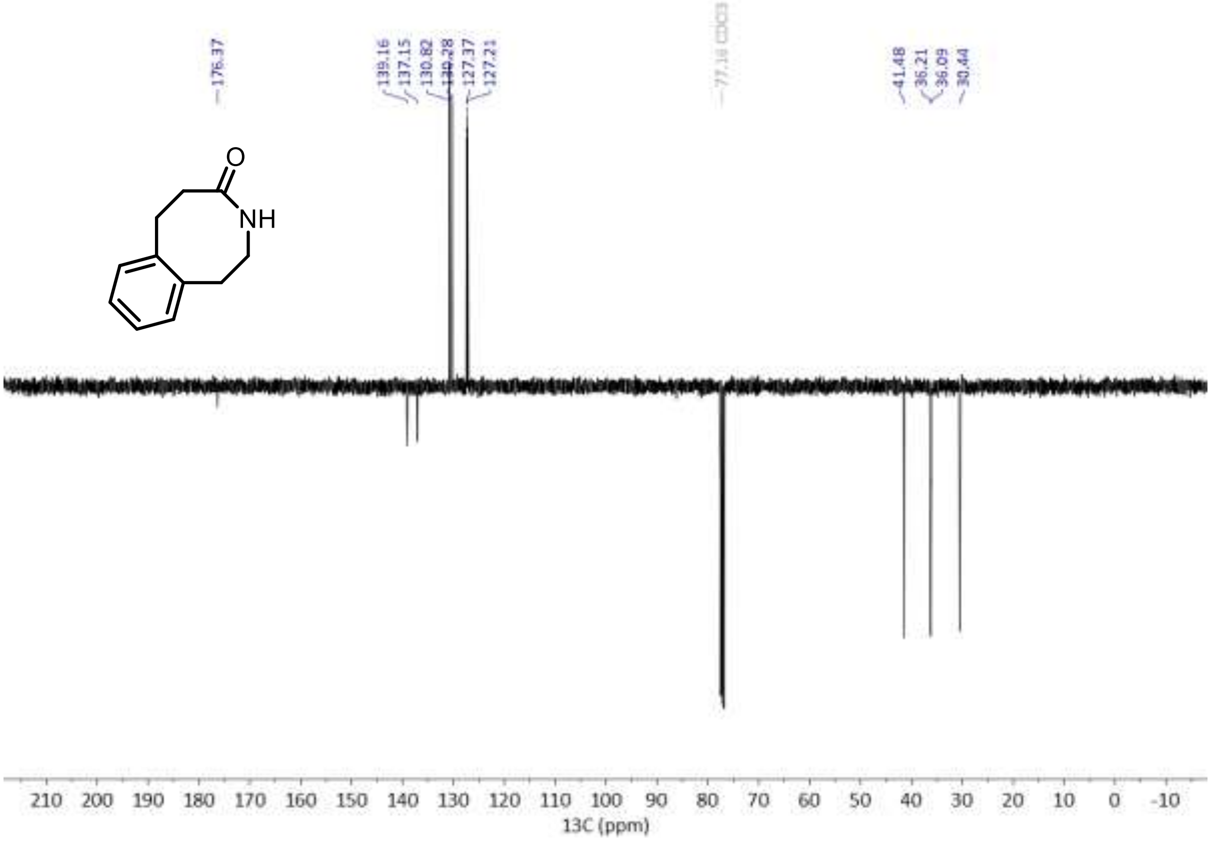

Compound 2k (^{1}H)

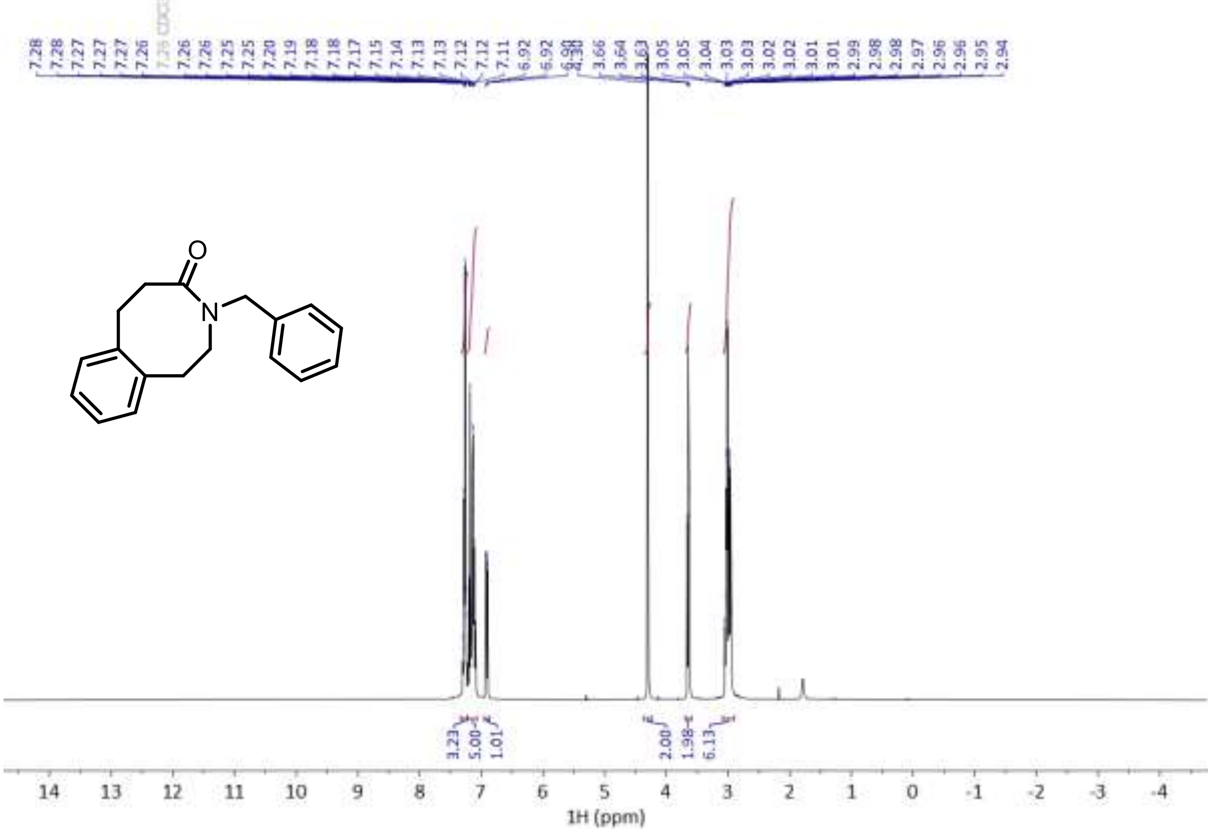

Compound 2k (^{13}C)

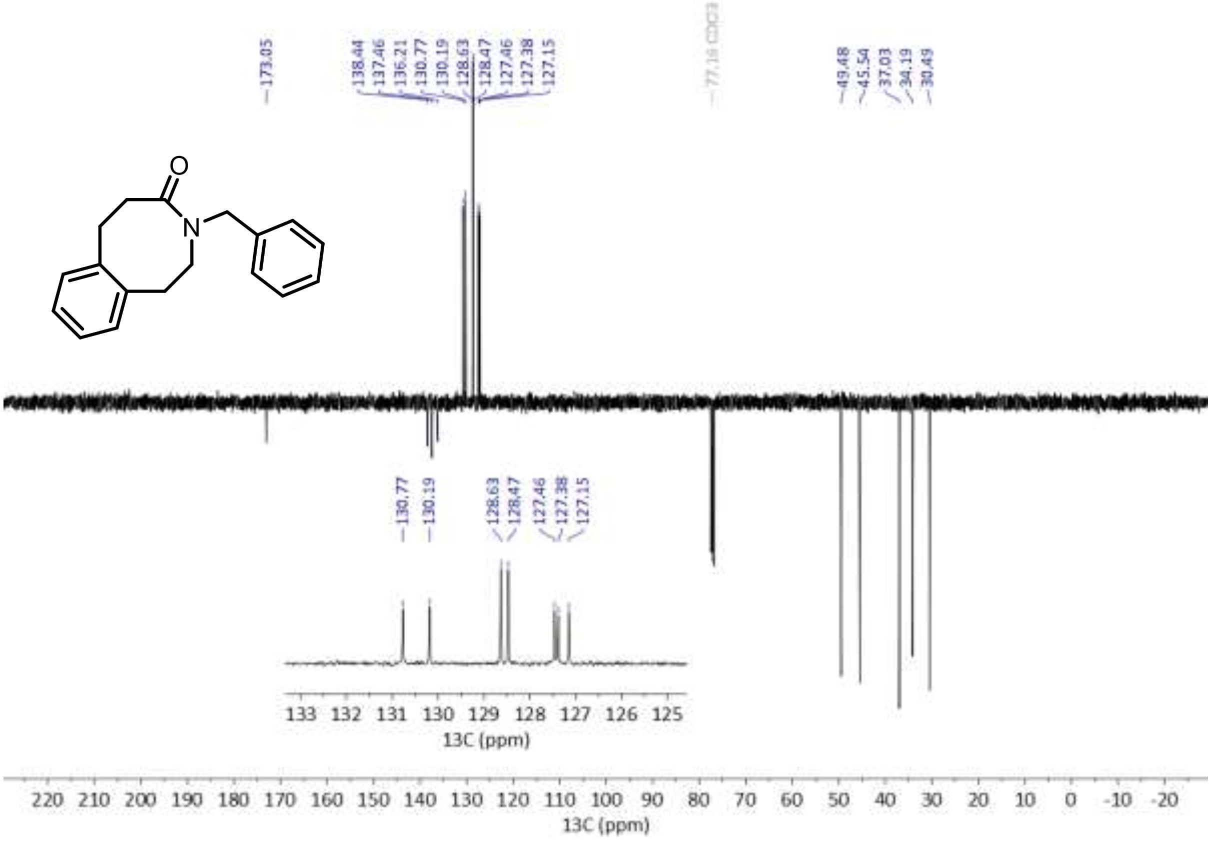

Compound 2n (^{1}H)

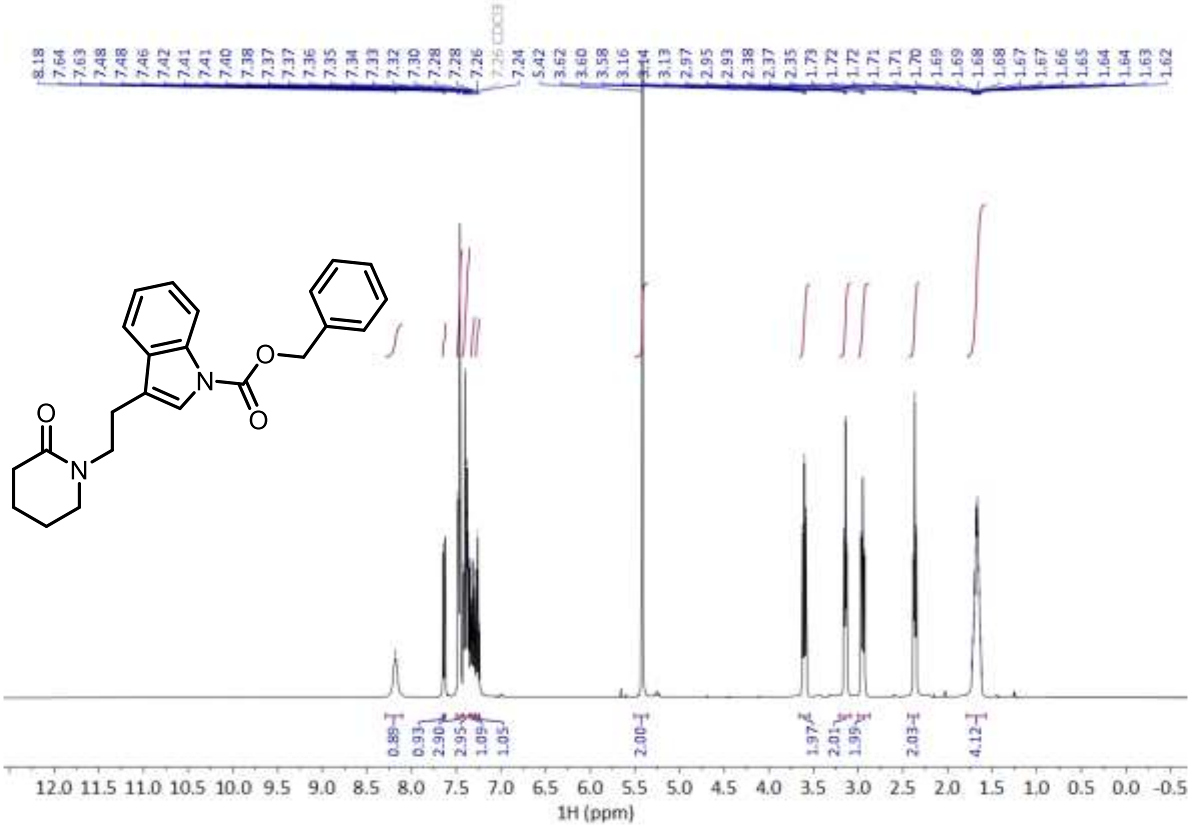

Compound 2n (^{13}C)

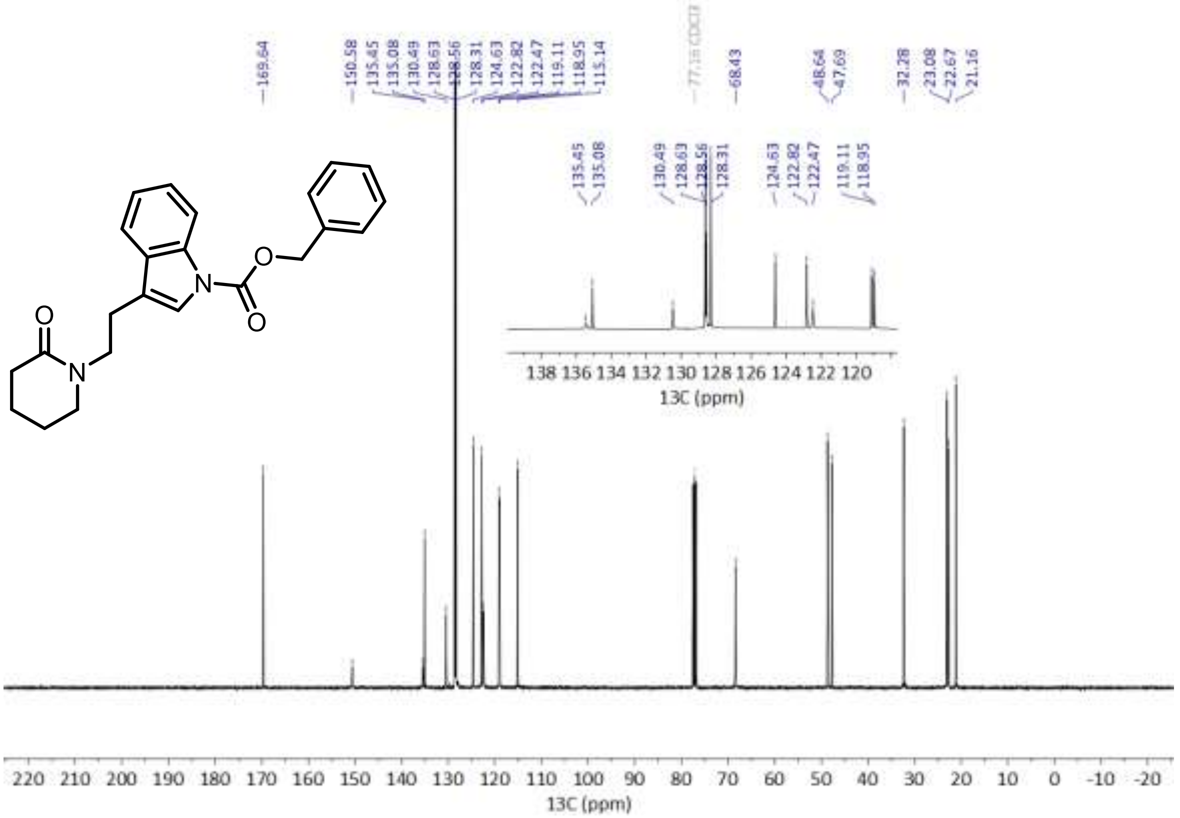

Compound 2p (¹H)

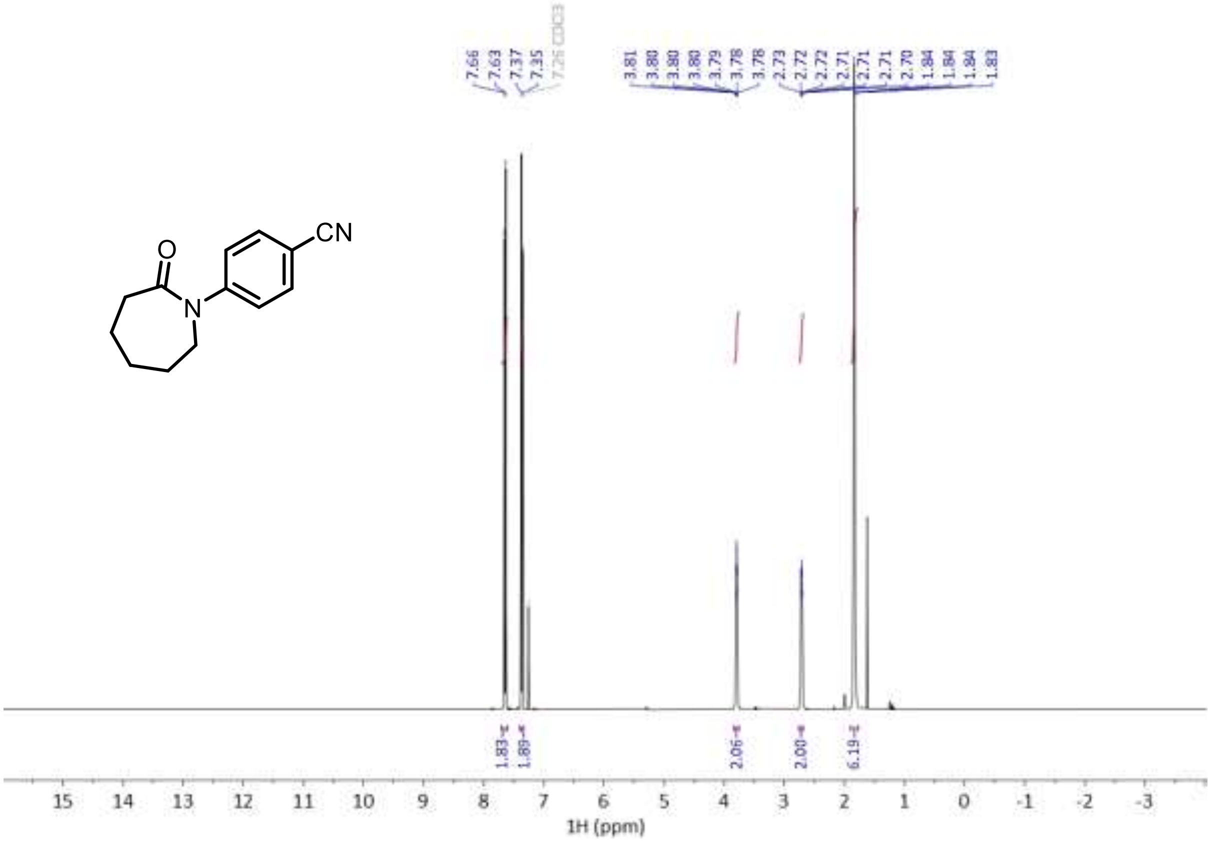

Compound 2p (¹³C)

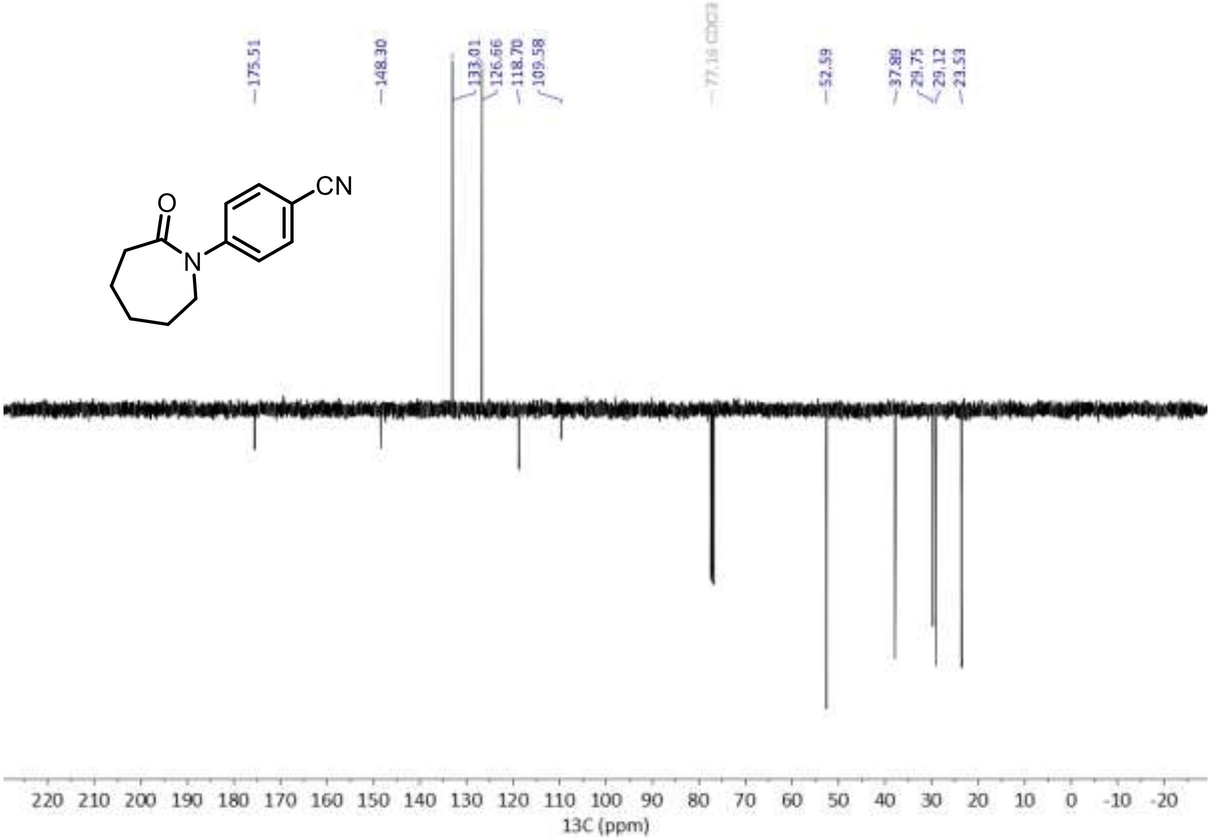

Compound 2q (^{1}H)

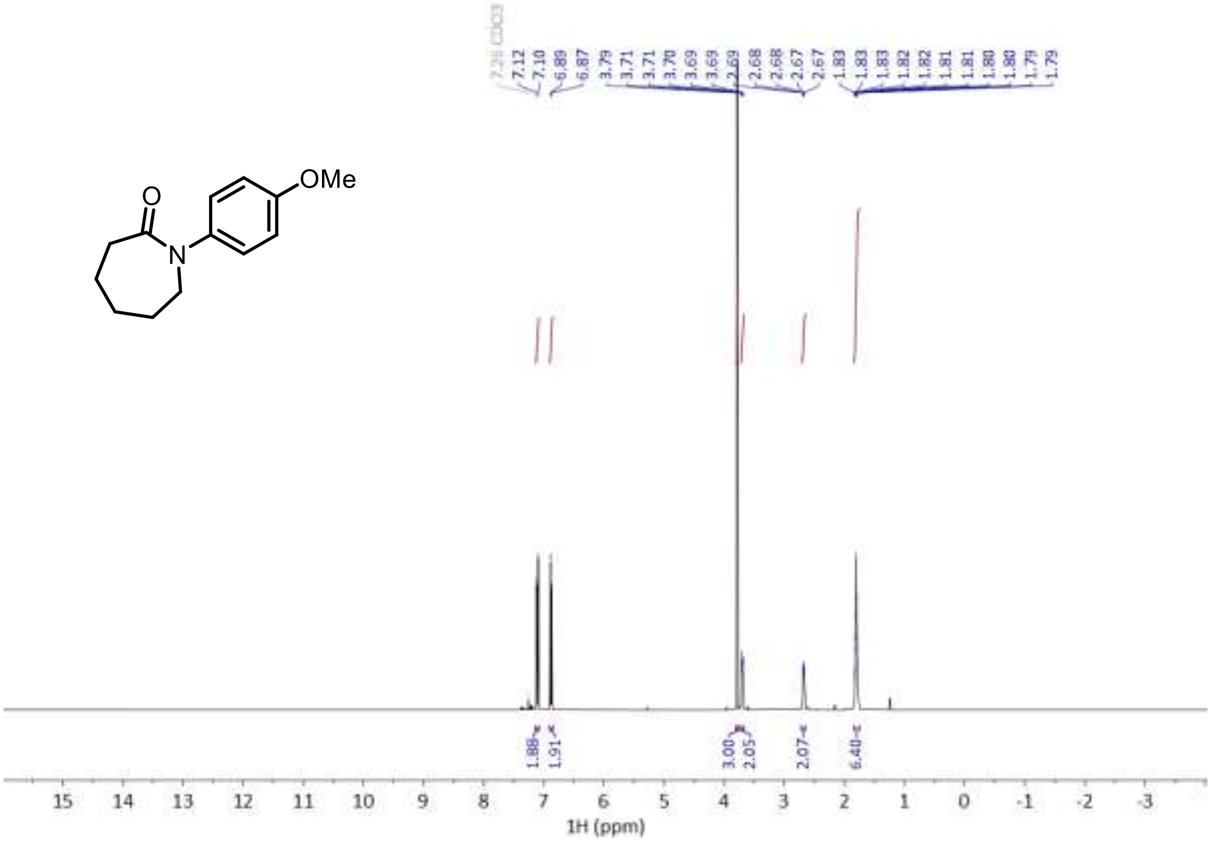

Compound 2q (^{13}C)

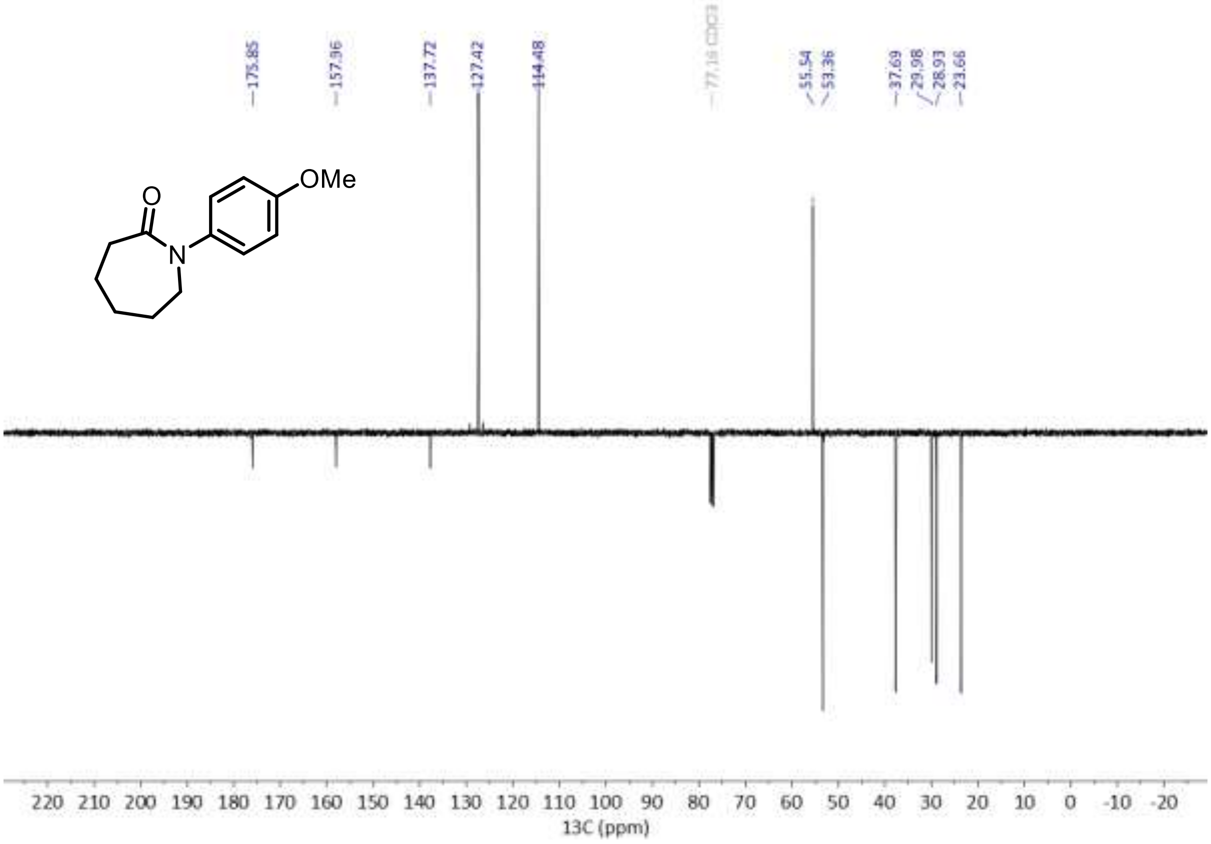

Compound 2r (^{1}H)

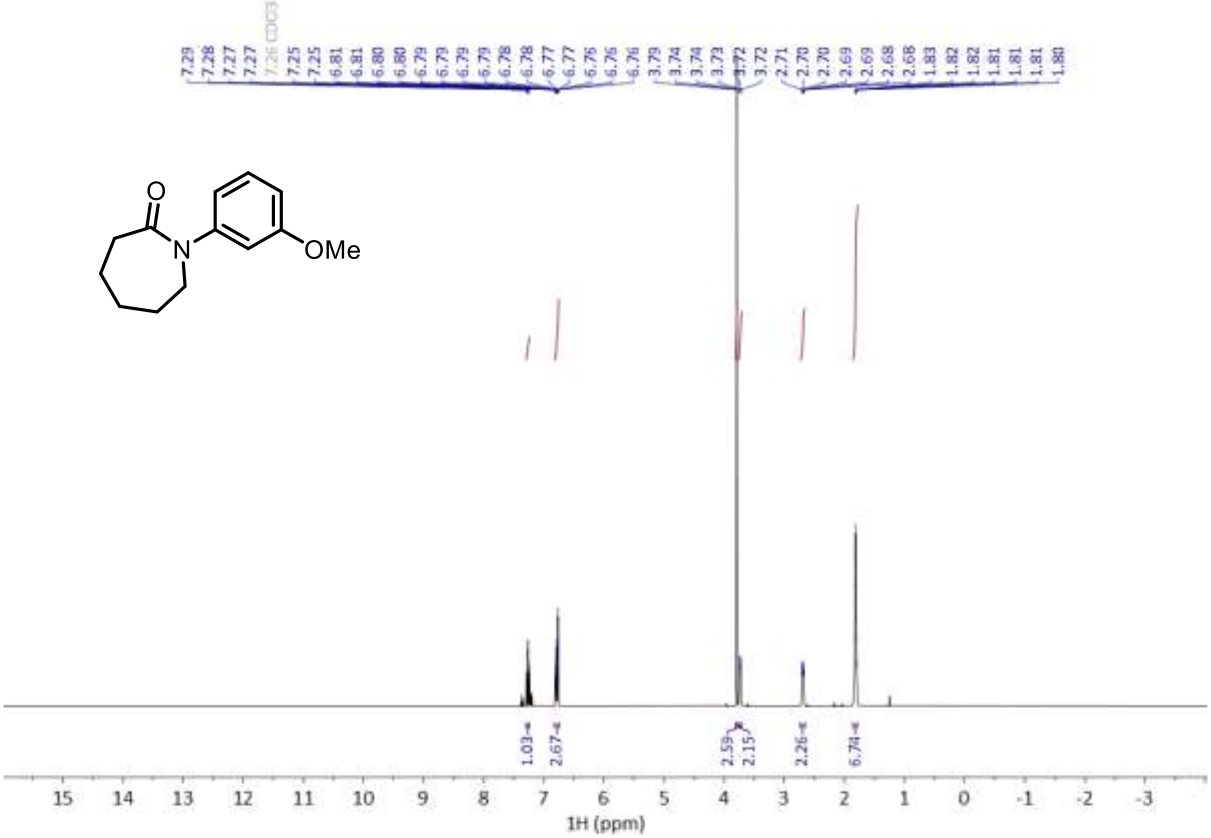

Compound 2r (^{13}C)

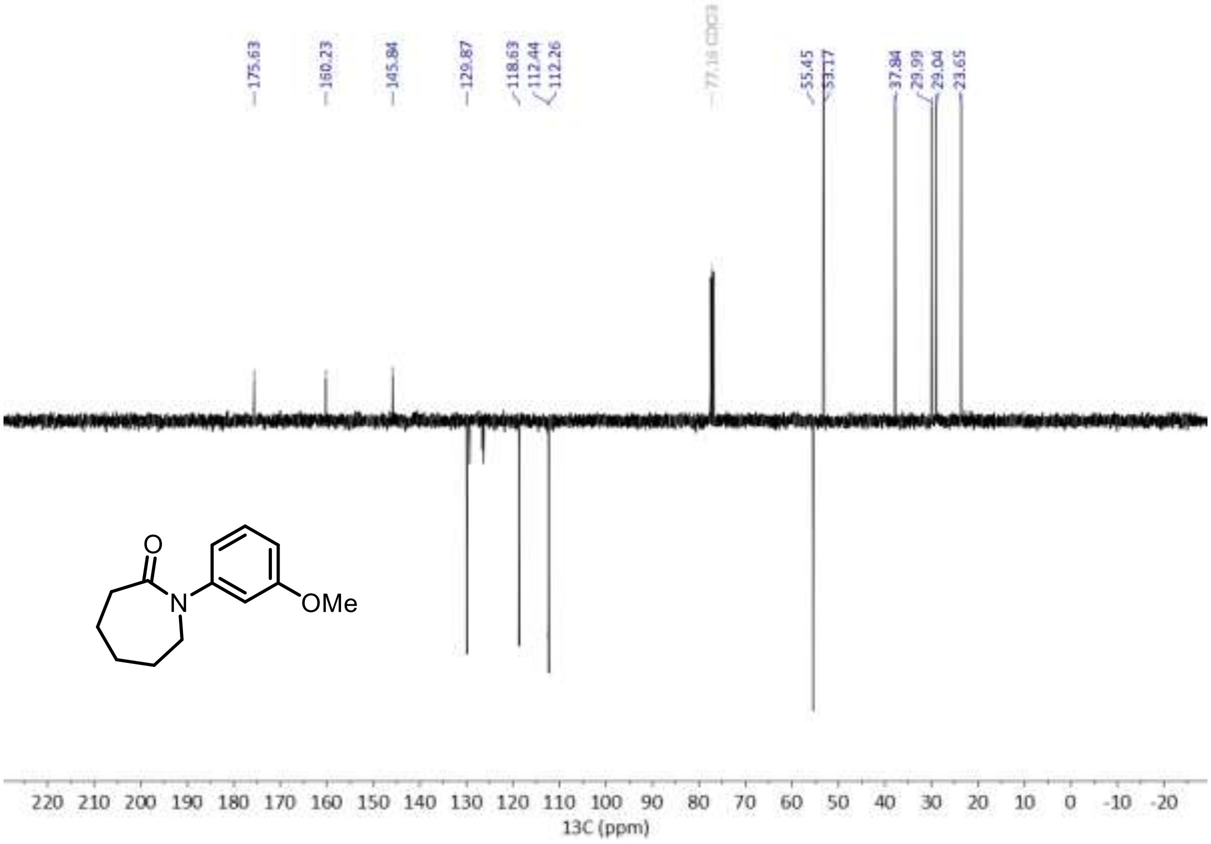

Compound 2t (^{1}H)

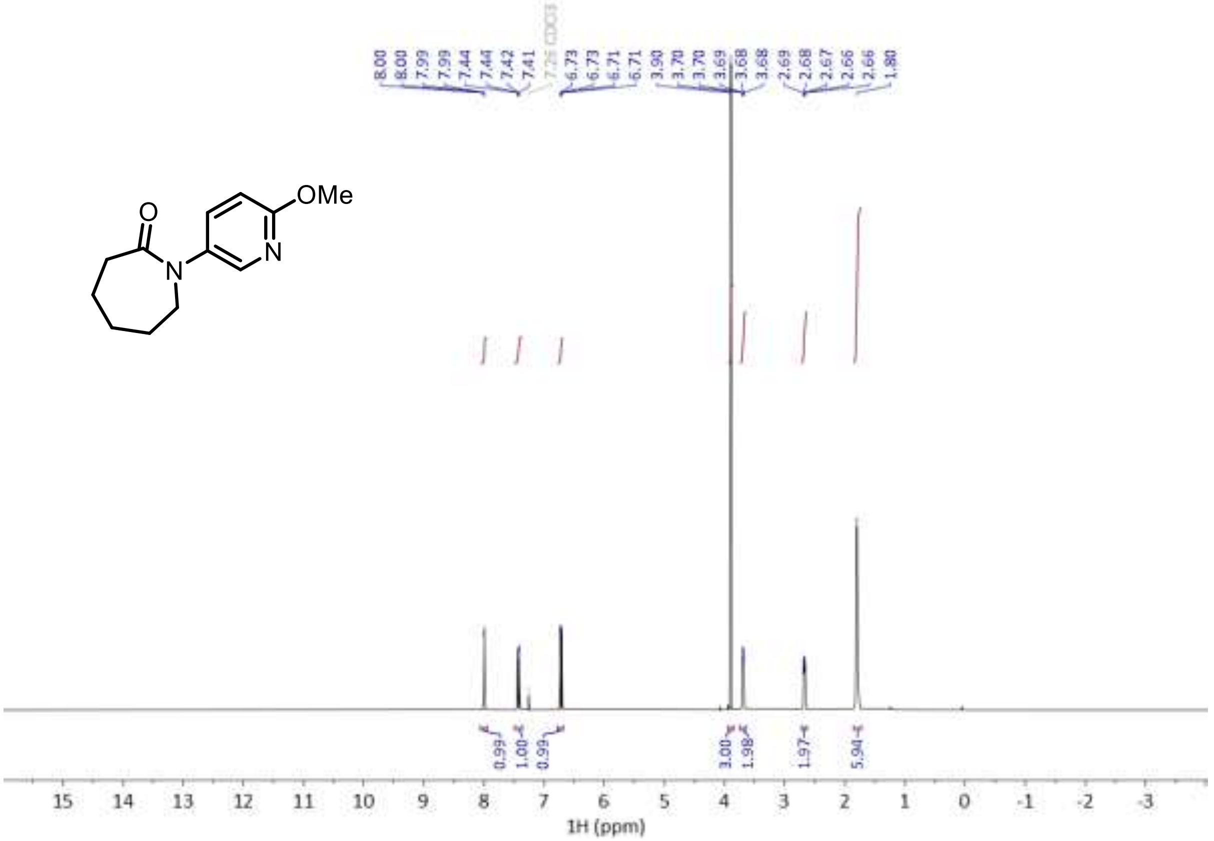

Compound 2t (^{13}C)

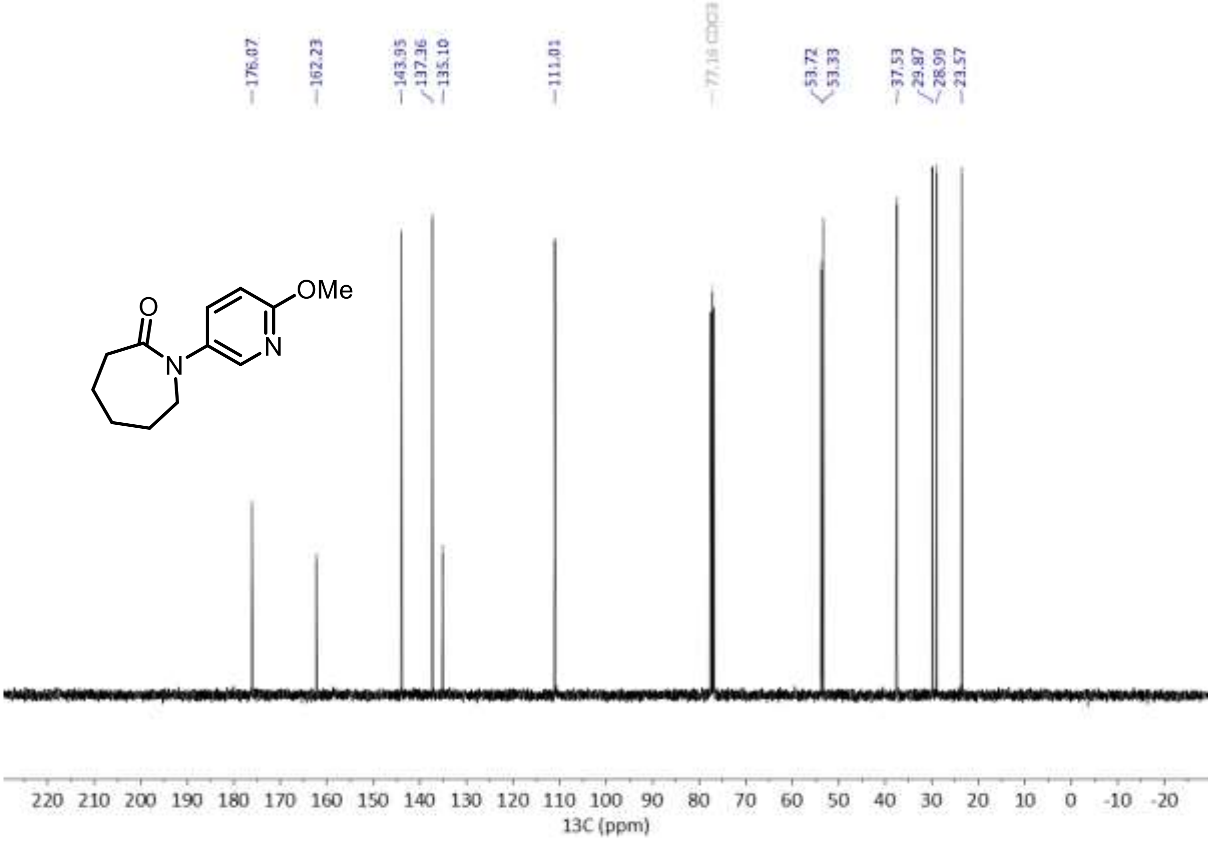

Compound 2u (^{1}H)

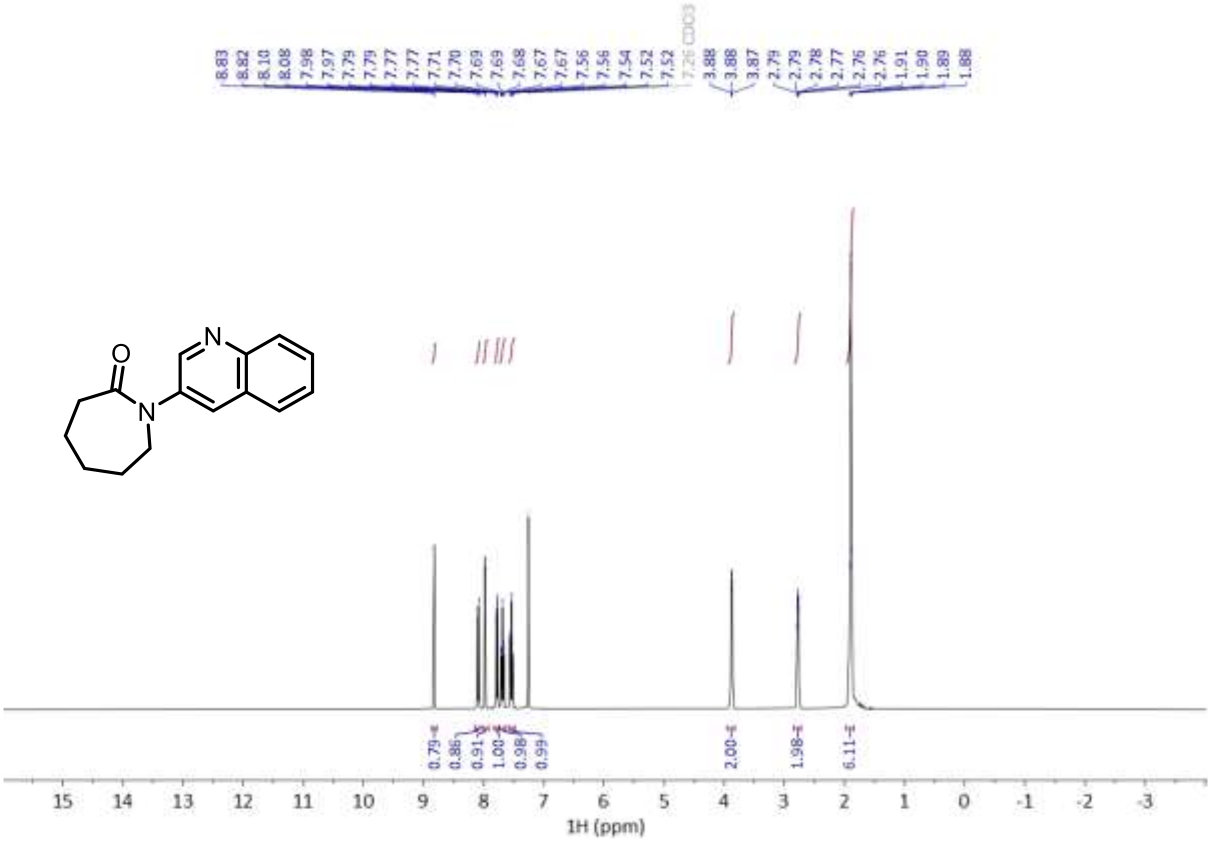

Compound 2u (^{13}C)

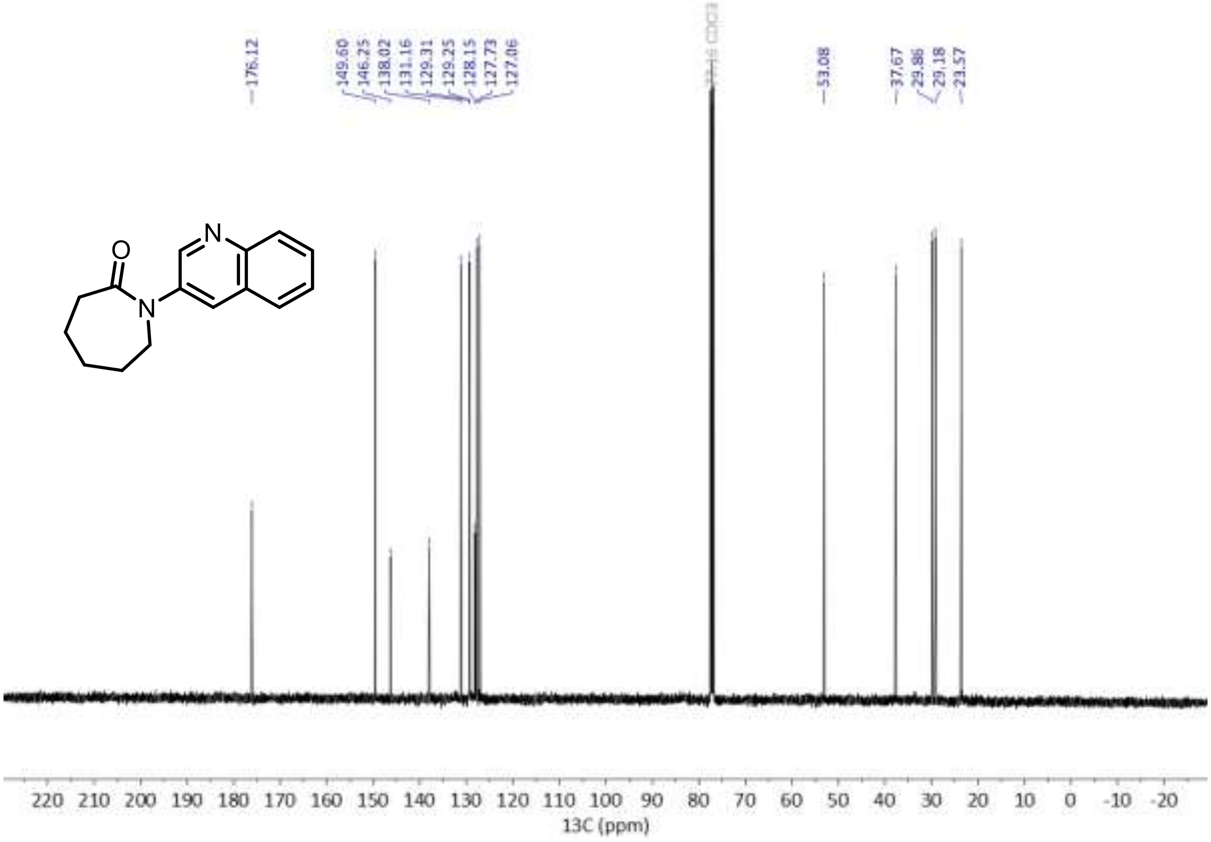

Compound 2v (^{1}H)

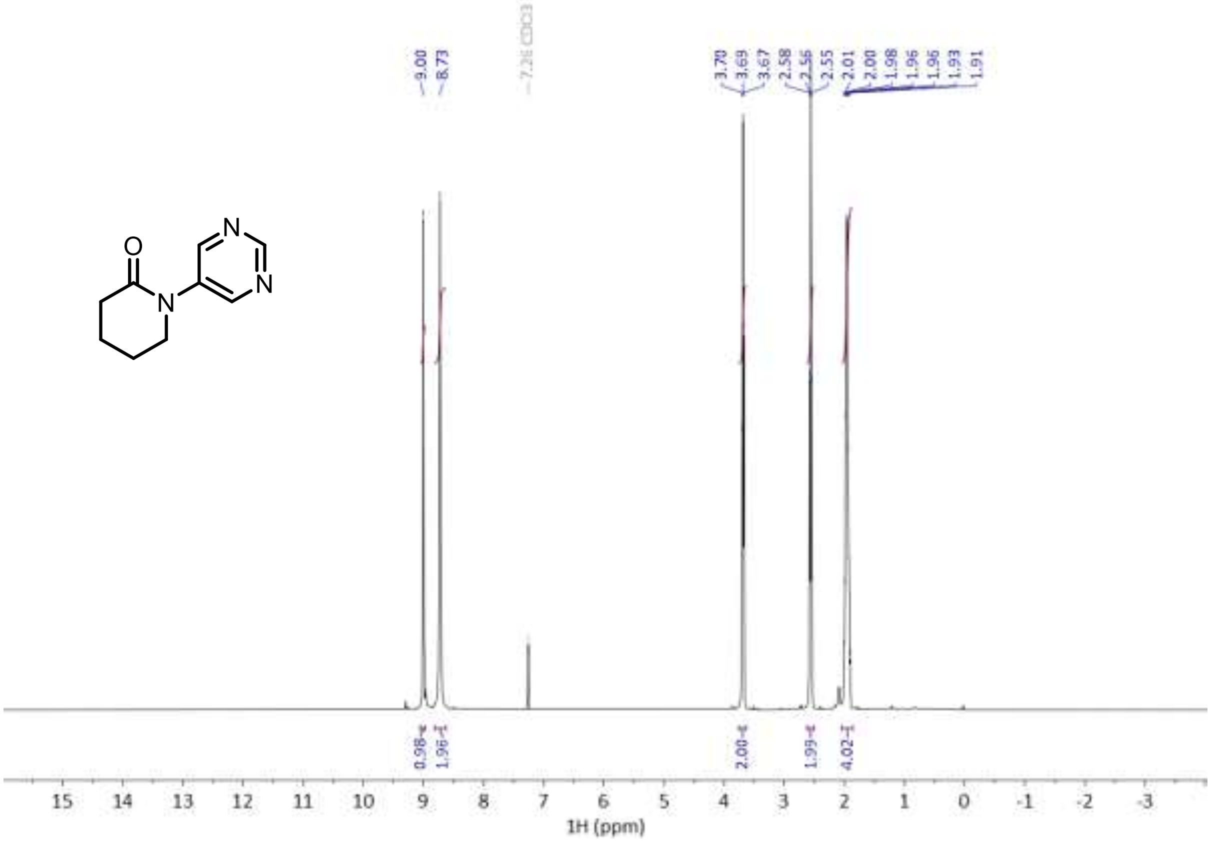

Compound 2v (^{13}C)

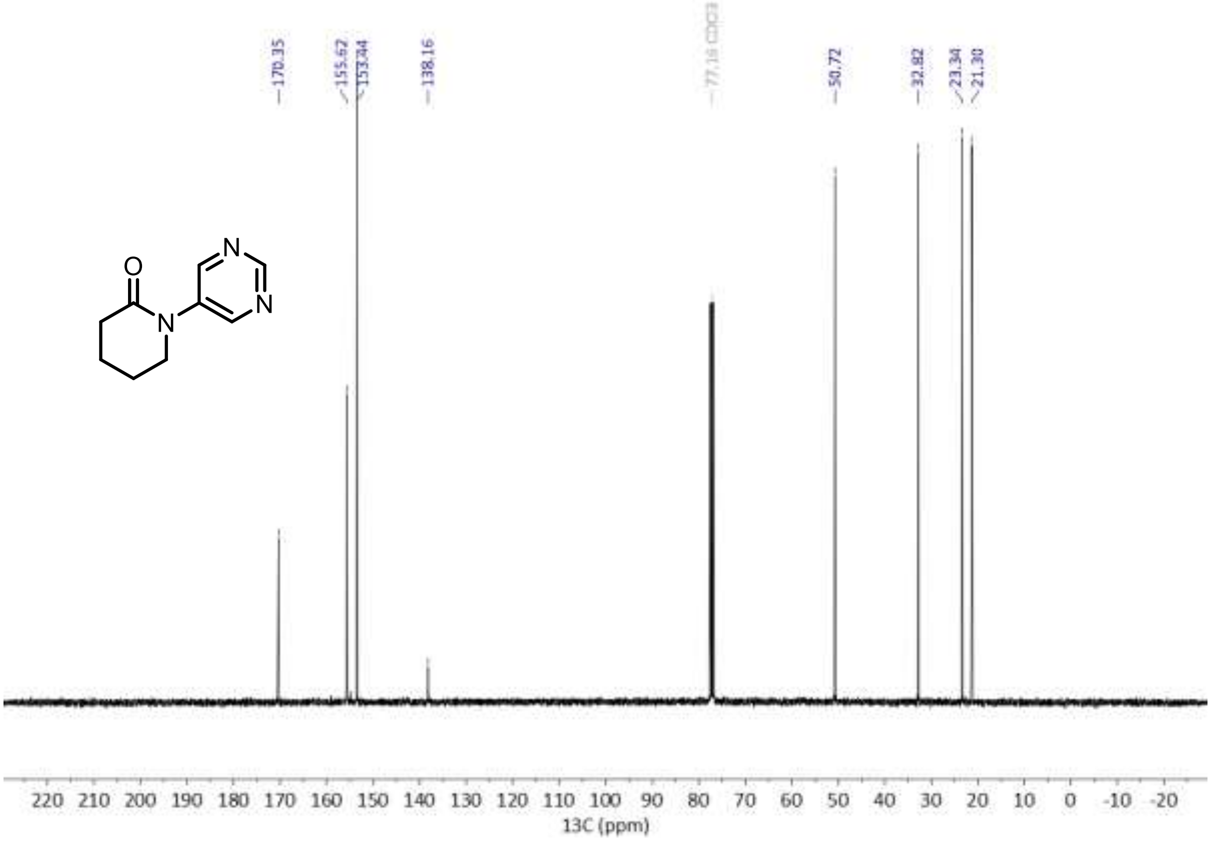

Compound 2w (^{1}H)

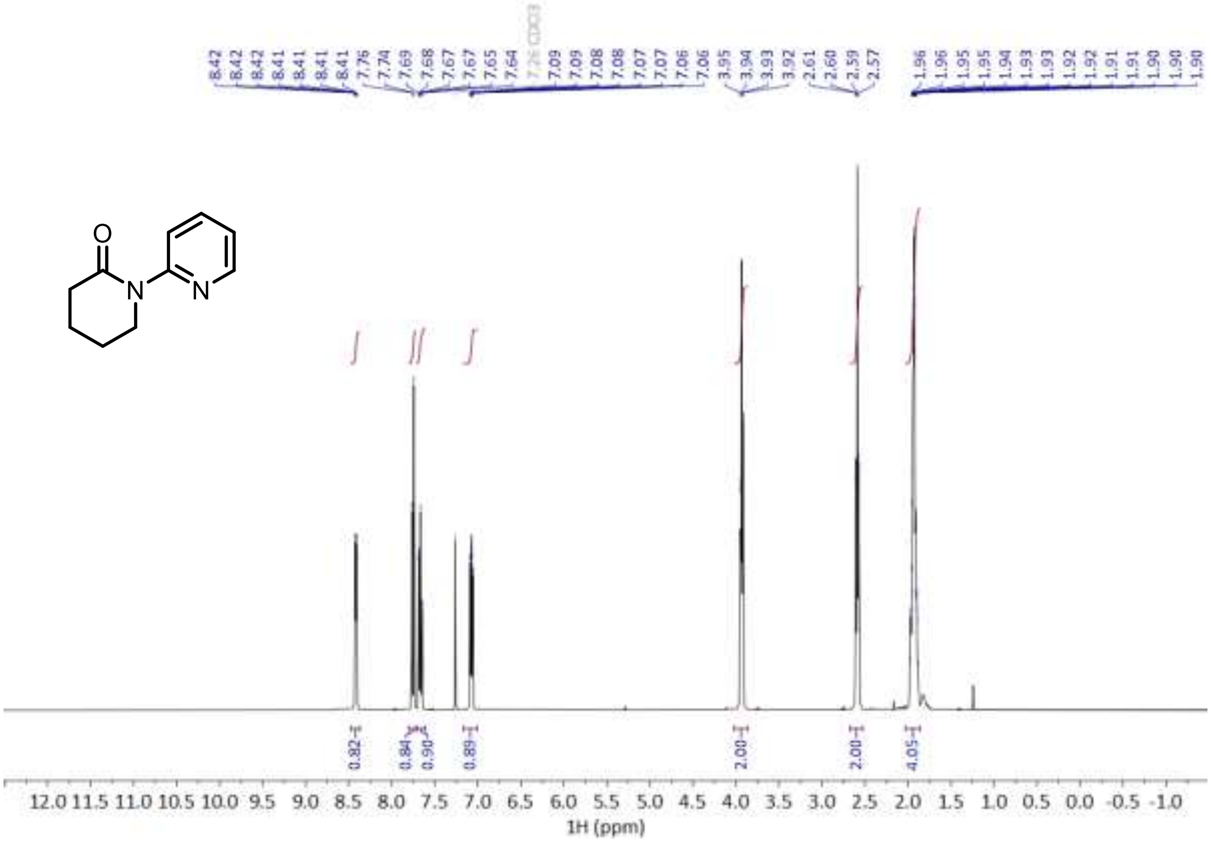

Compound 2w (^{13}C)

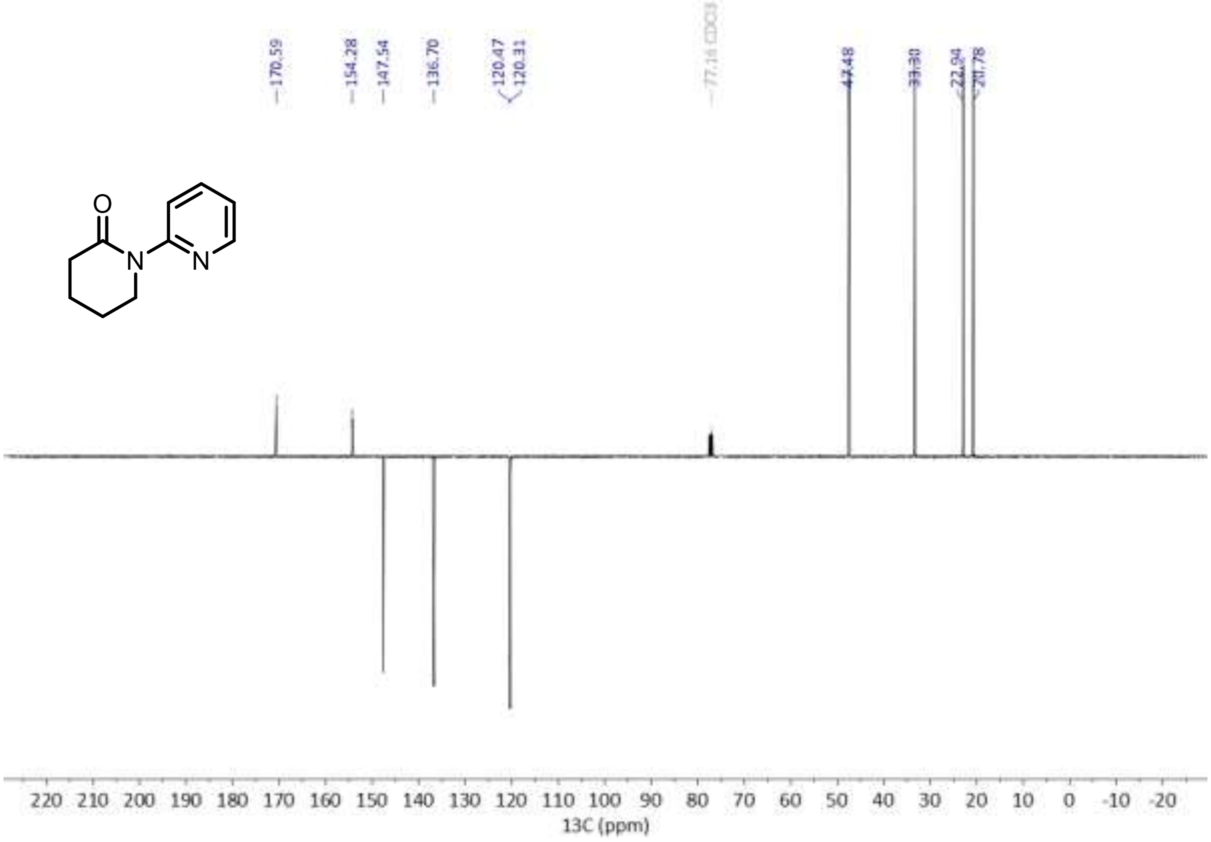

Compound 1y (^{1}H)

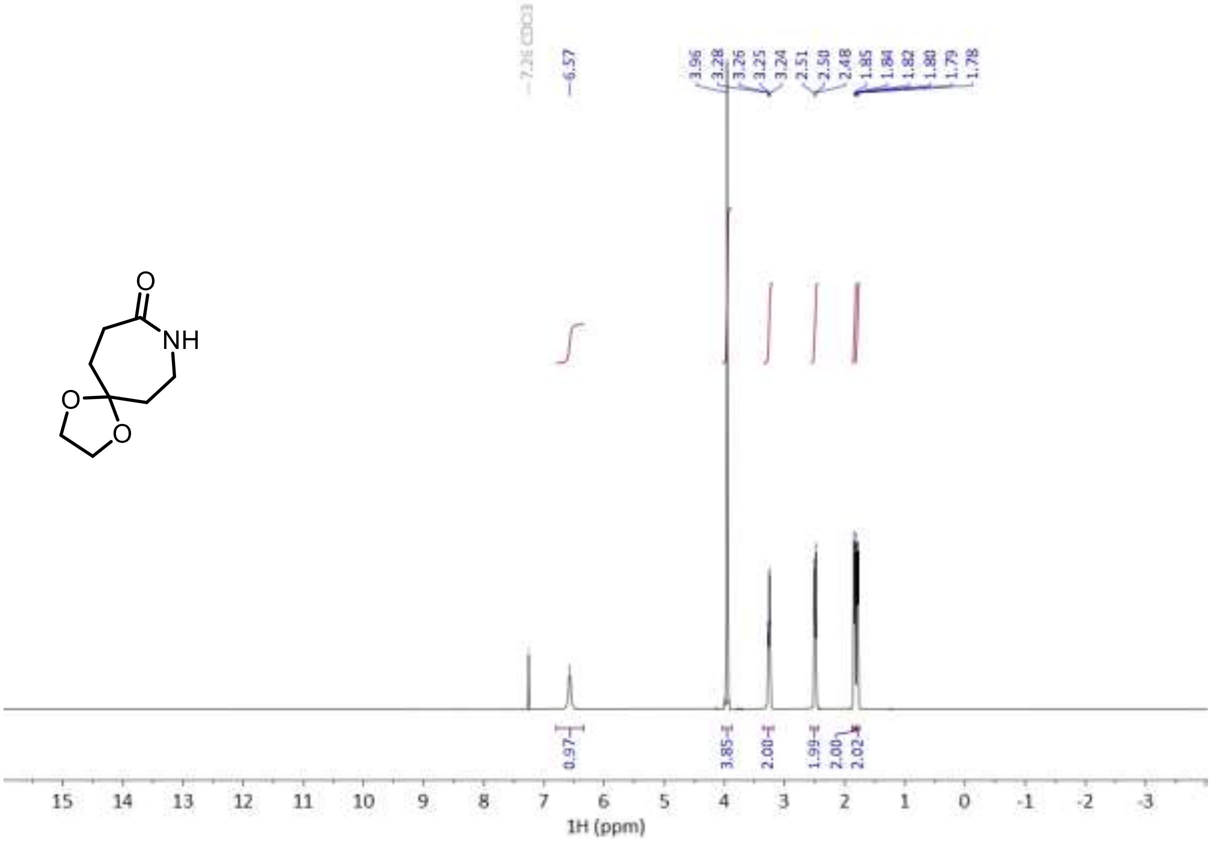

Compound 1y (^{13}C)

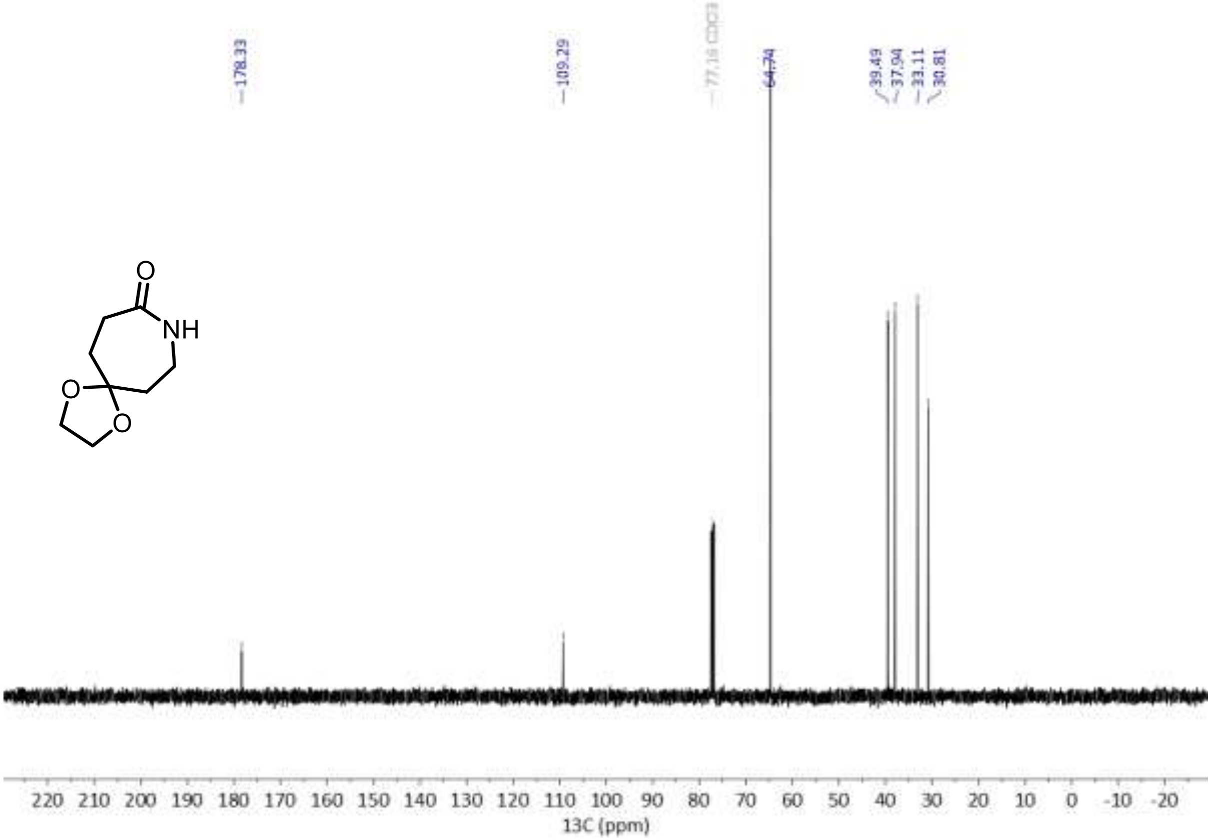

Compound 2y (^{1}H)

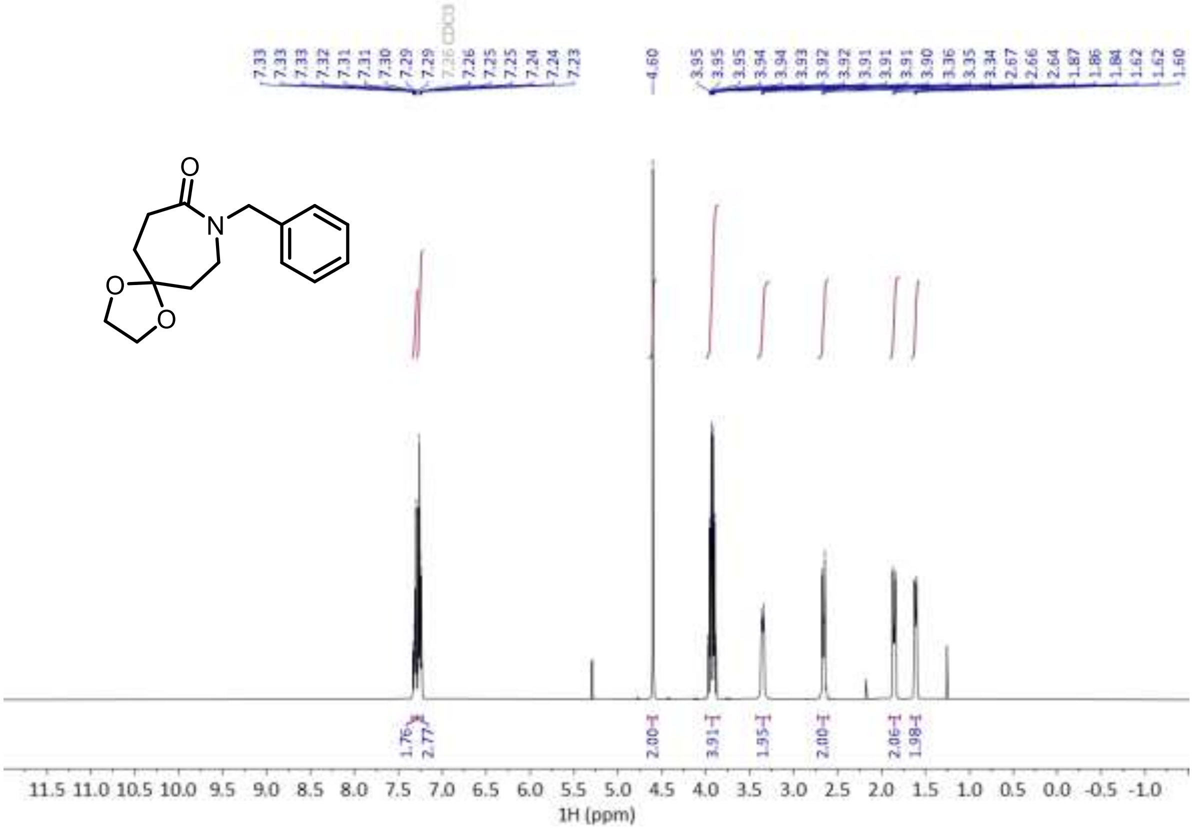

Compound 2y (^{13}C)

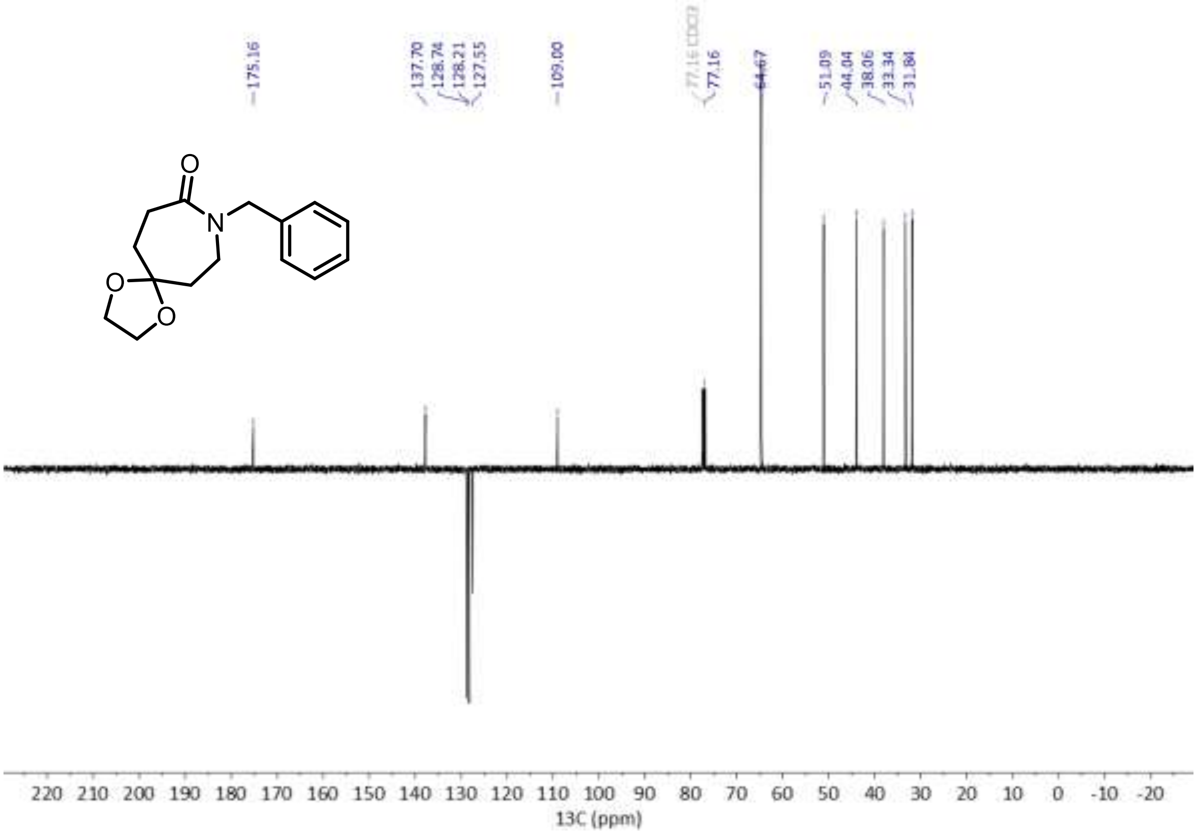

Compound 1z (¹H)

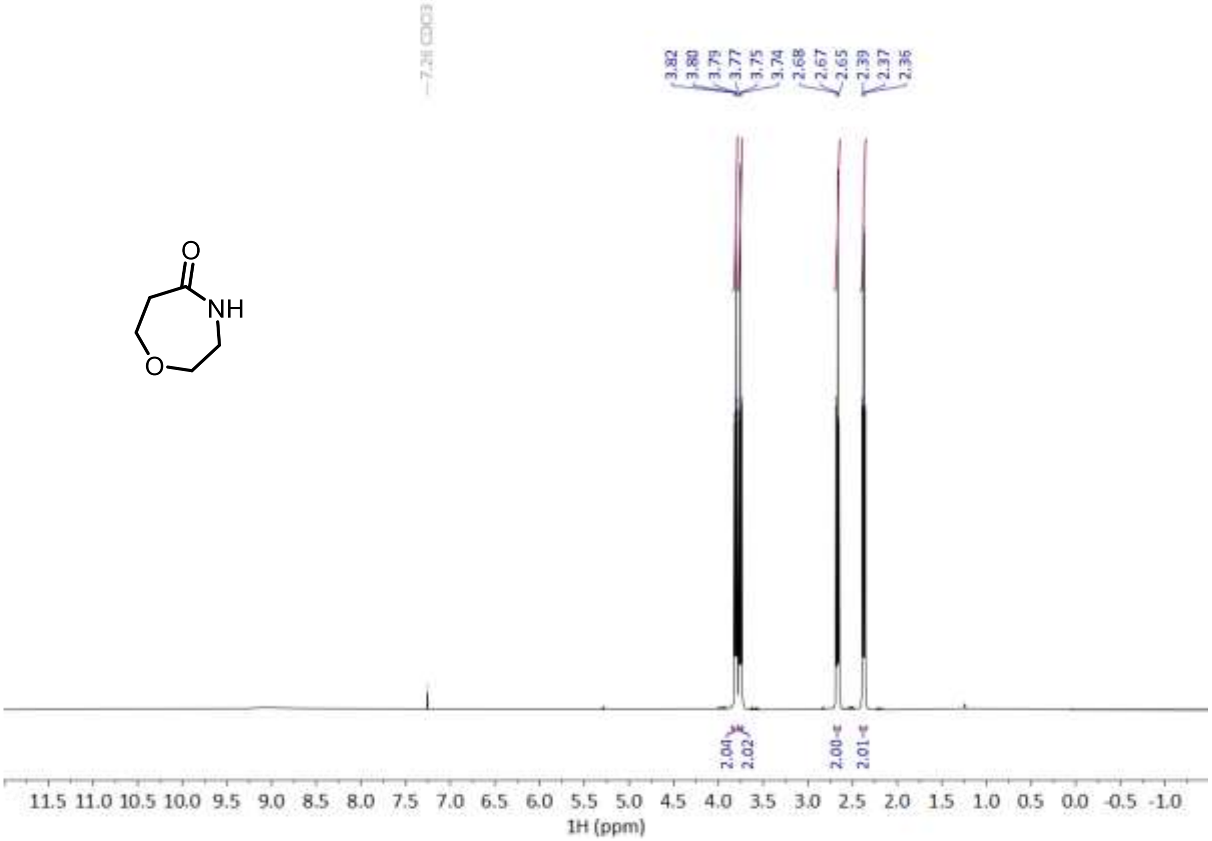

Compound 1z (¹³C)

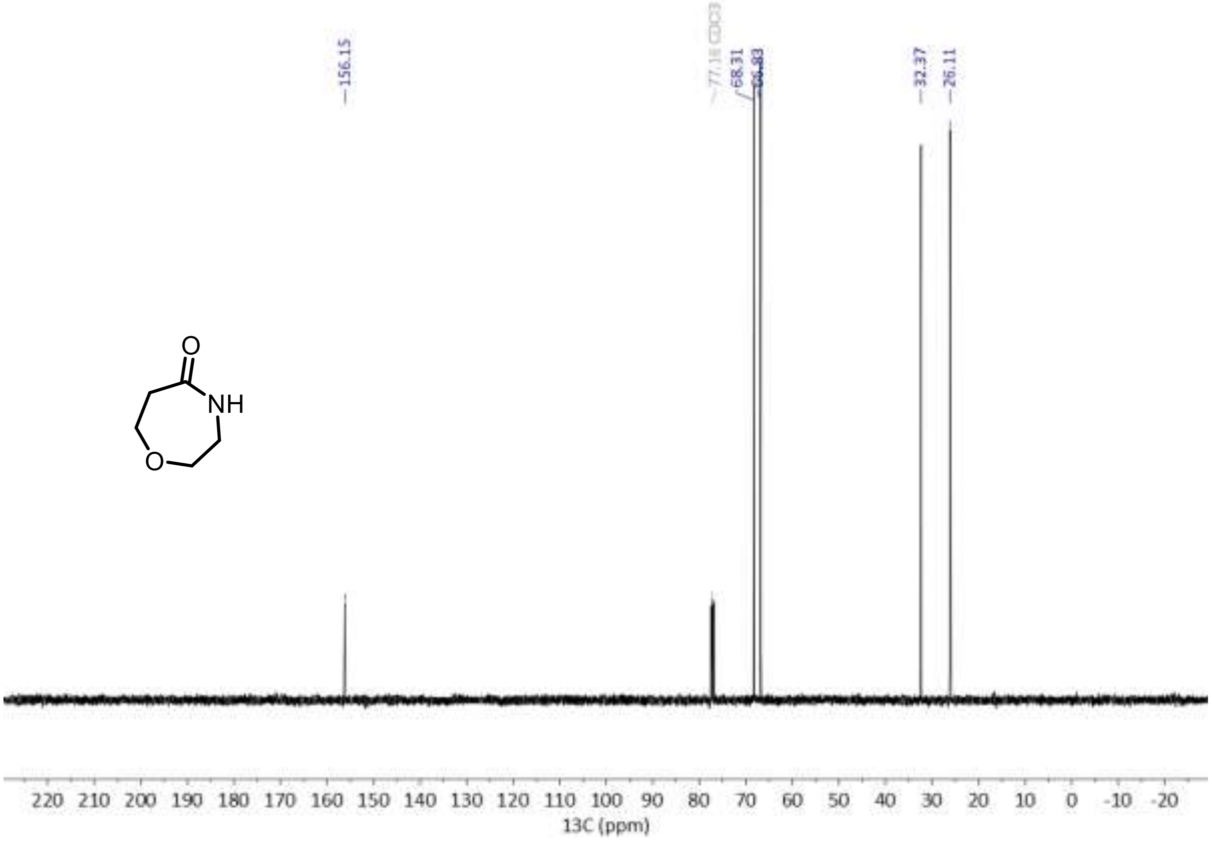

Compound 1aa (^{1}H)

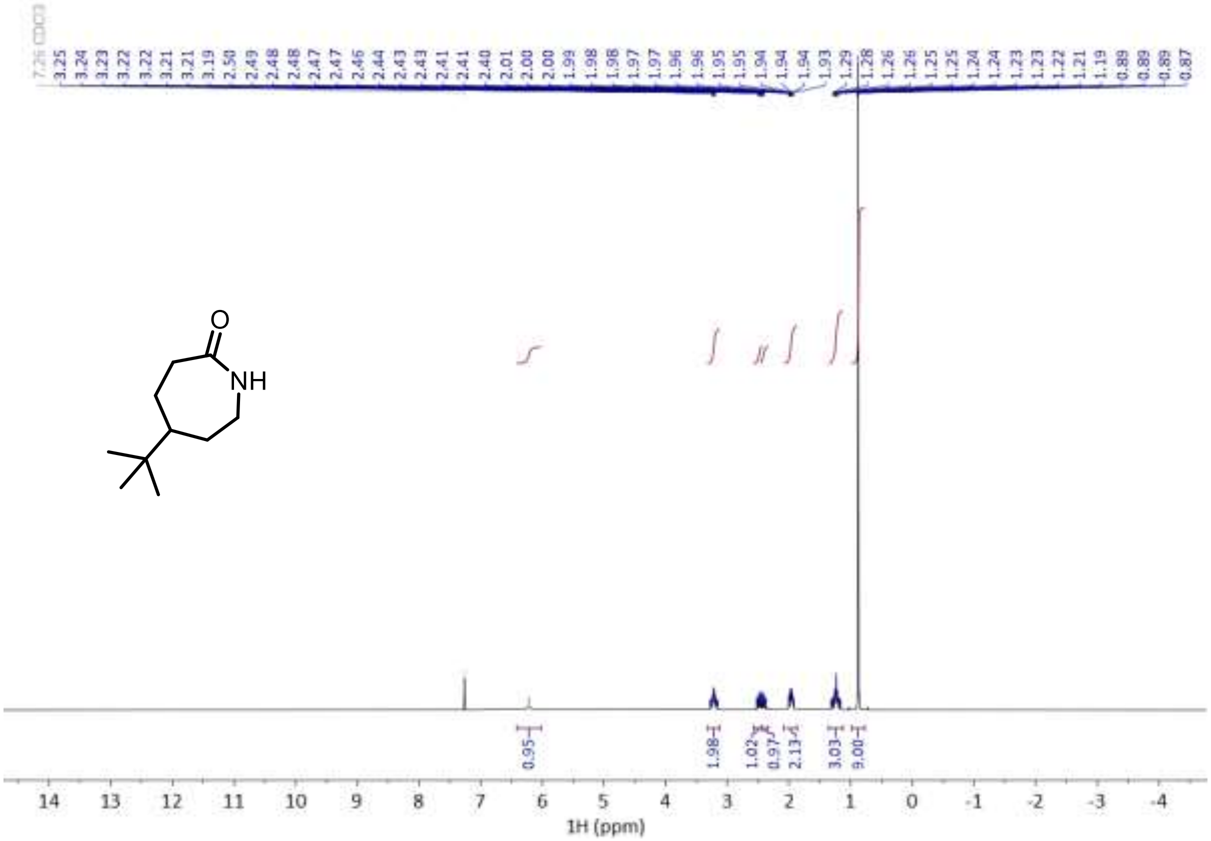

Compound 1aa (^{13}C)

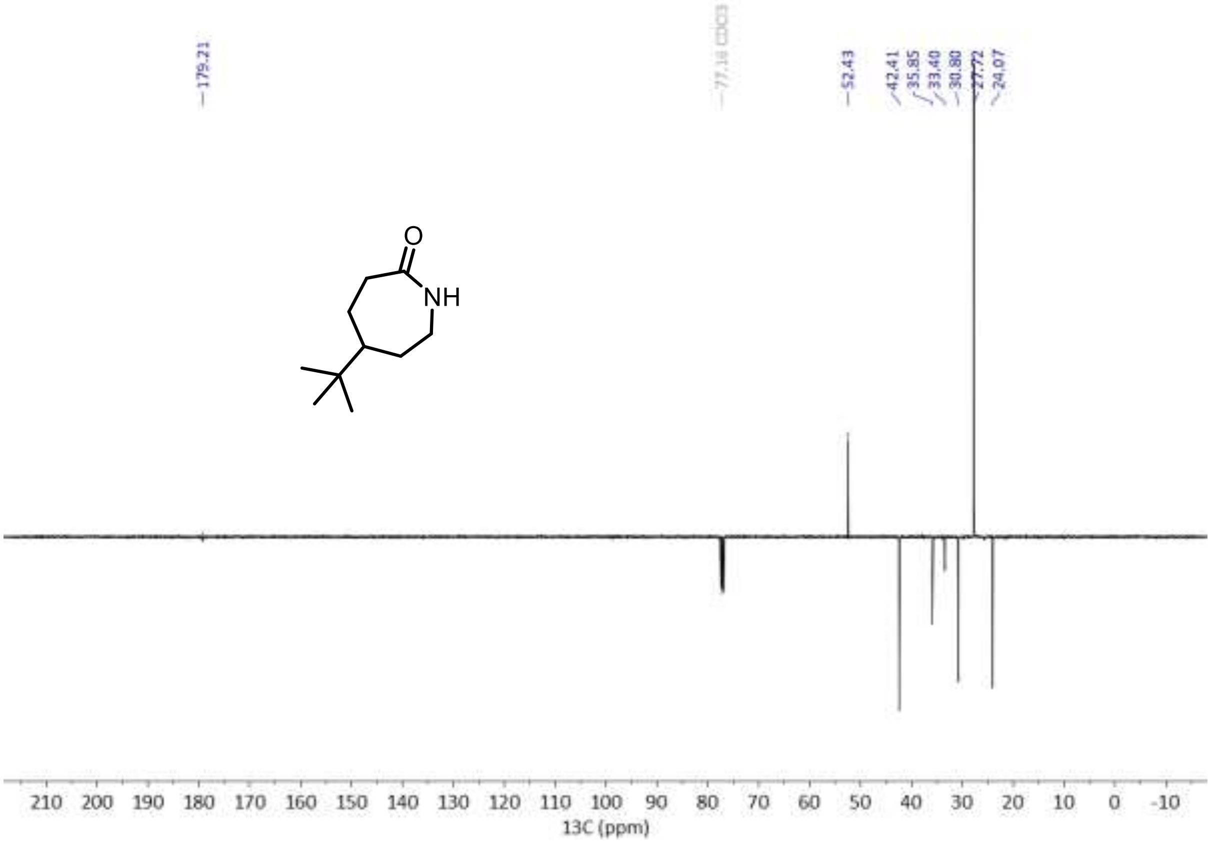

Compound 2aa (¹H)

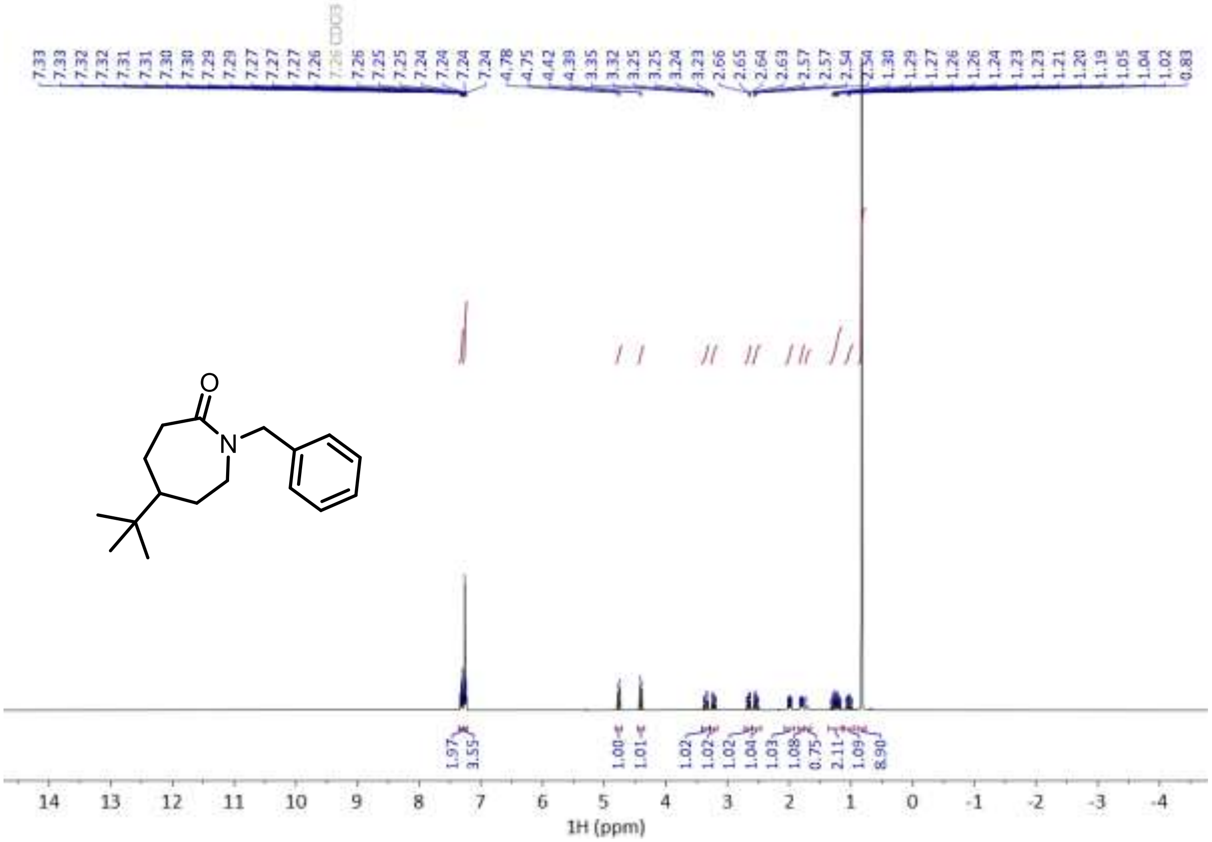

Compound 2aa (¹³C)

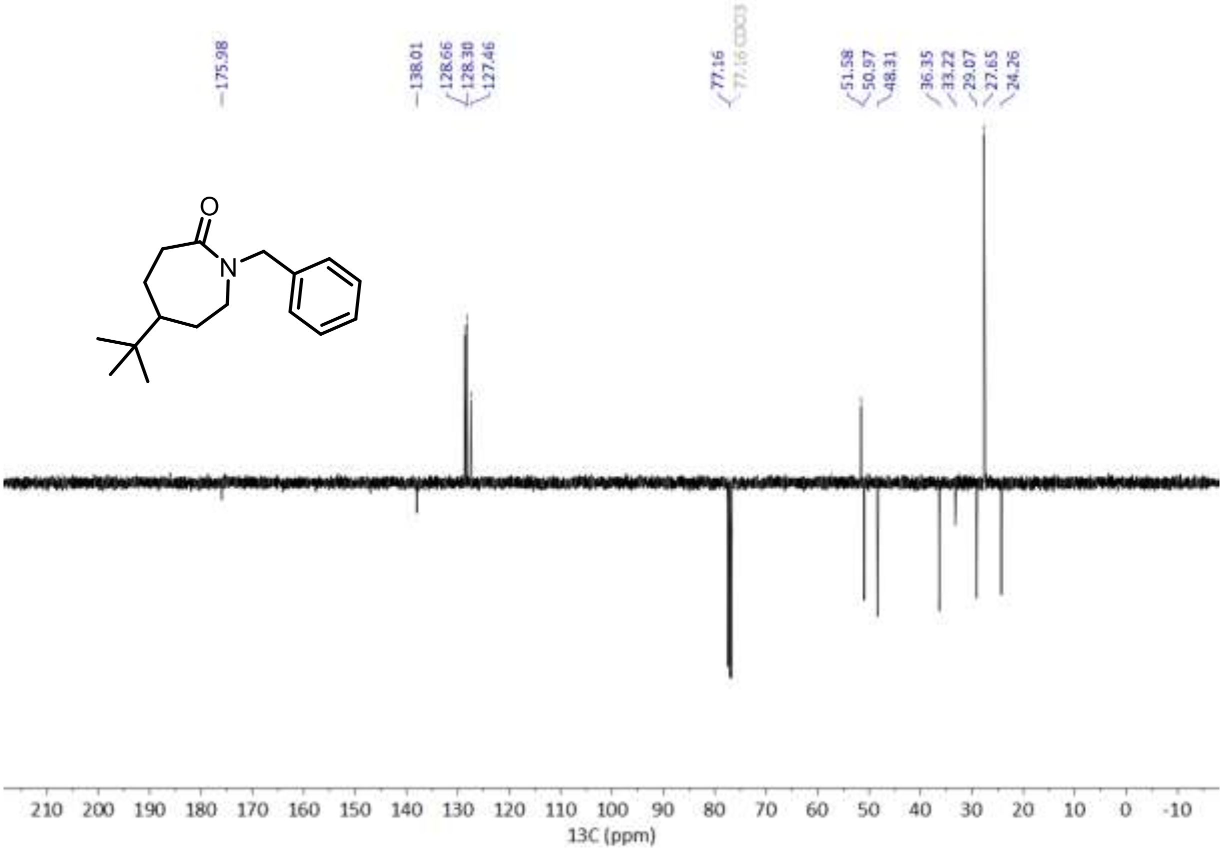

Compound 2ad (^{1}H)

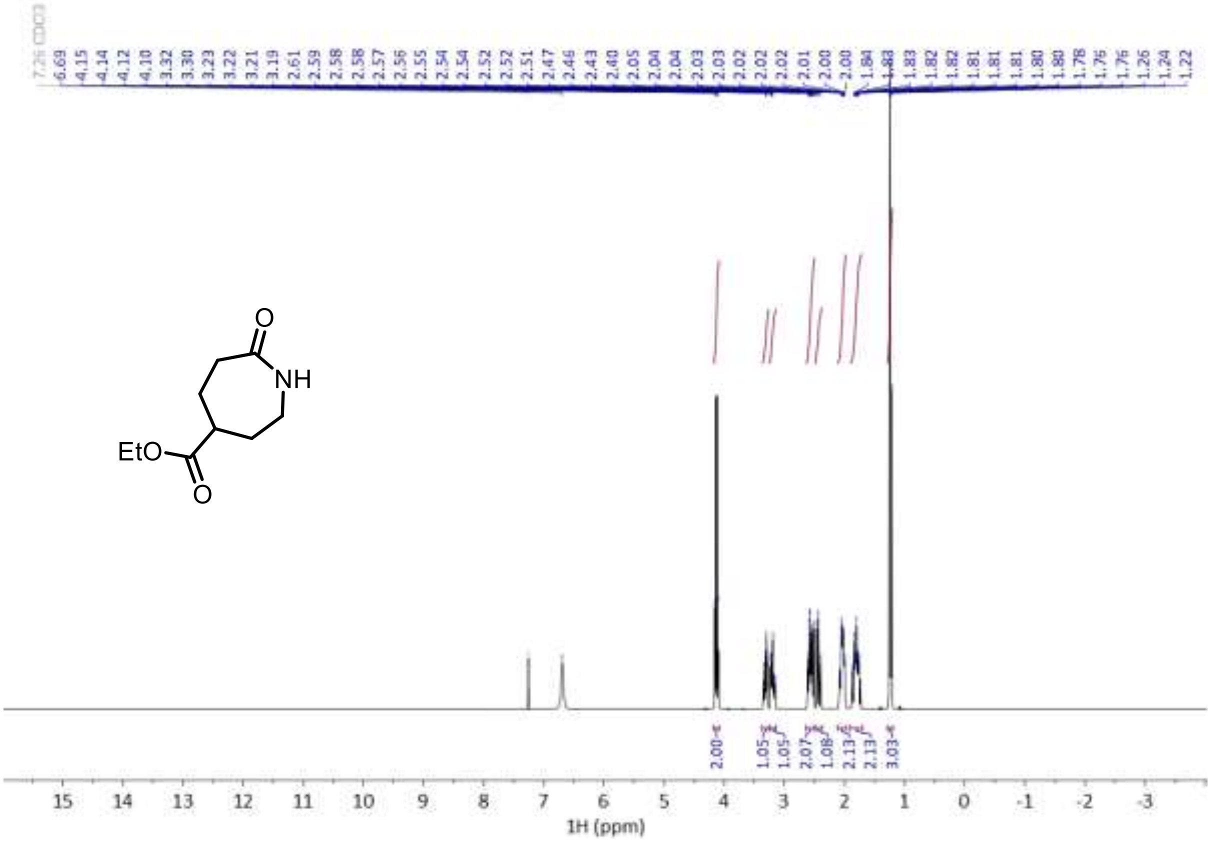

Compound 2ad (^{13}C)

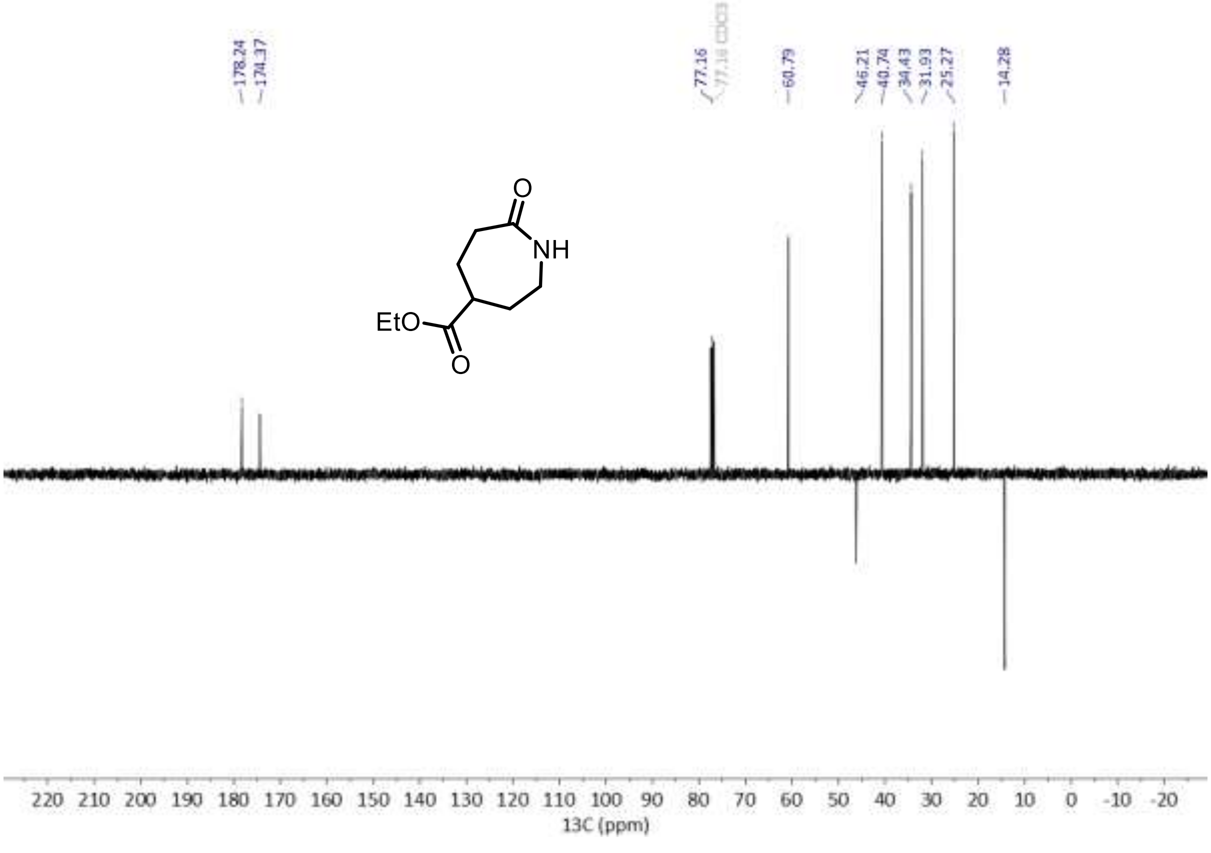

Compound 2ae (^{1}H)

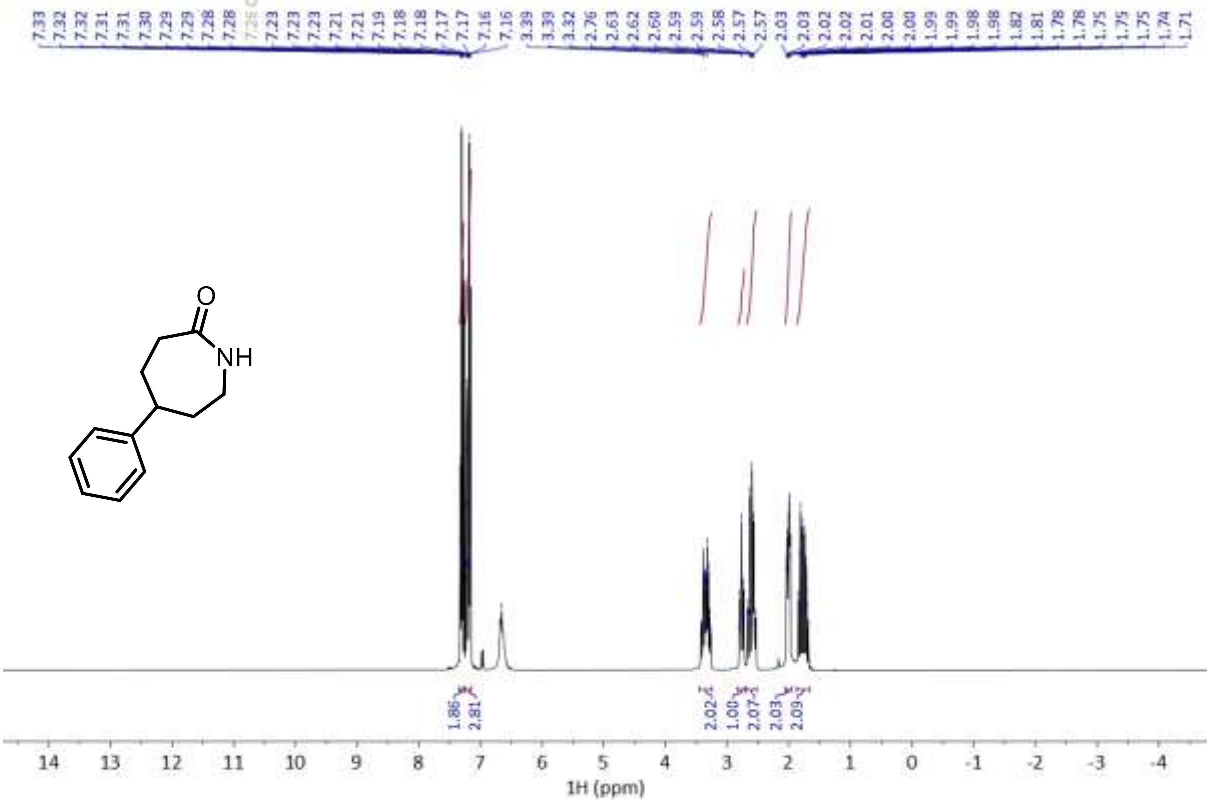

Compound 2ae (^{13}C)

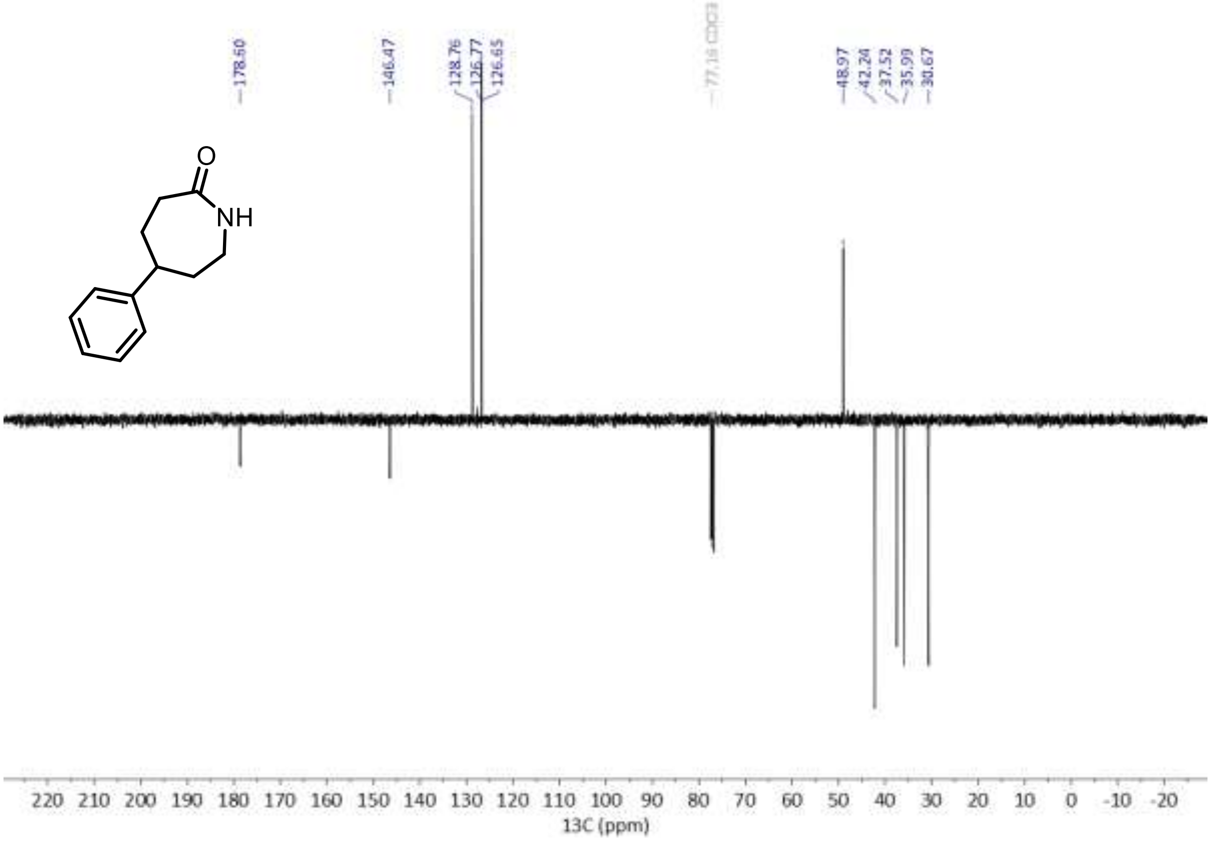

Compound 4a (^{1}H)

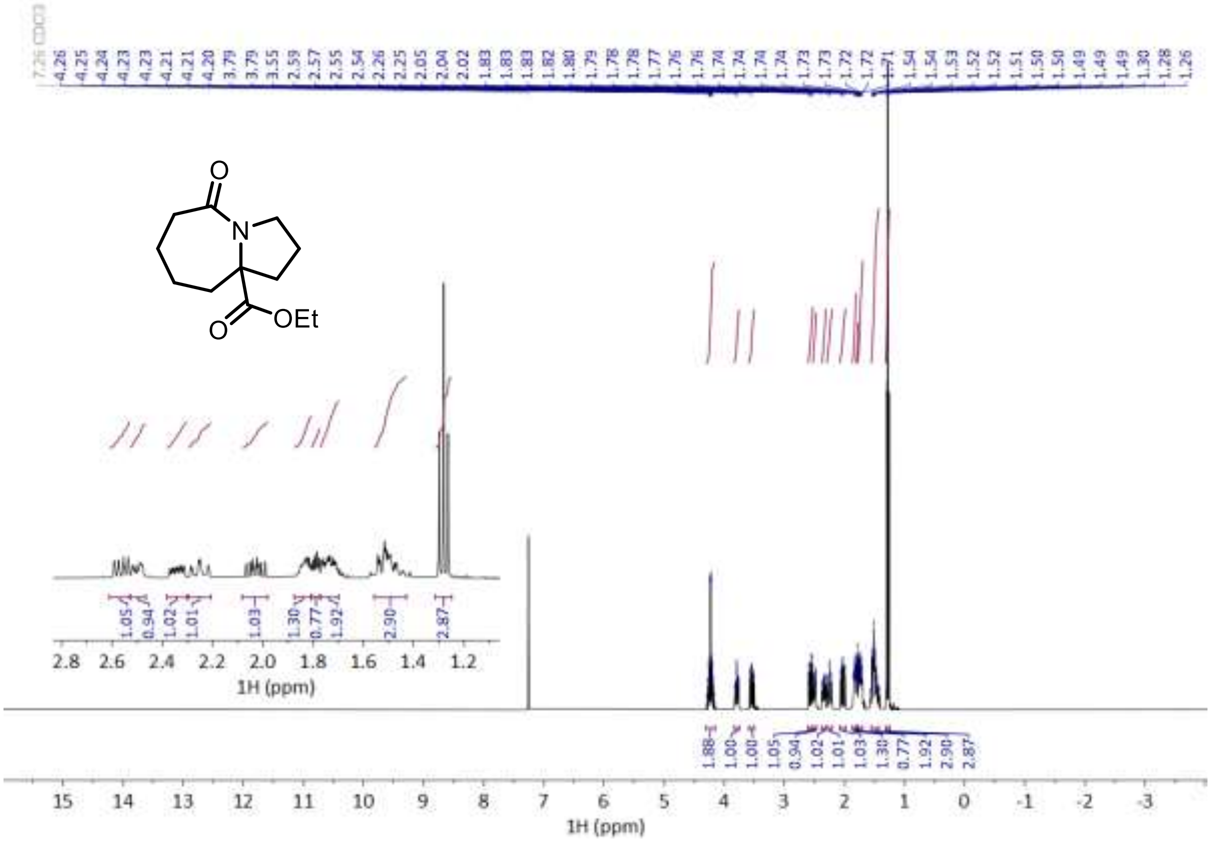

Compound 4a (^{13}C)

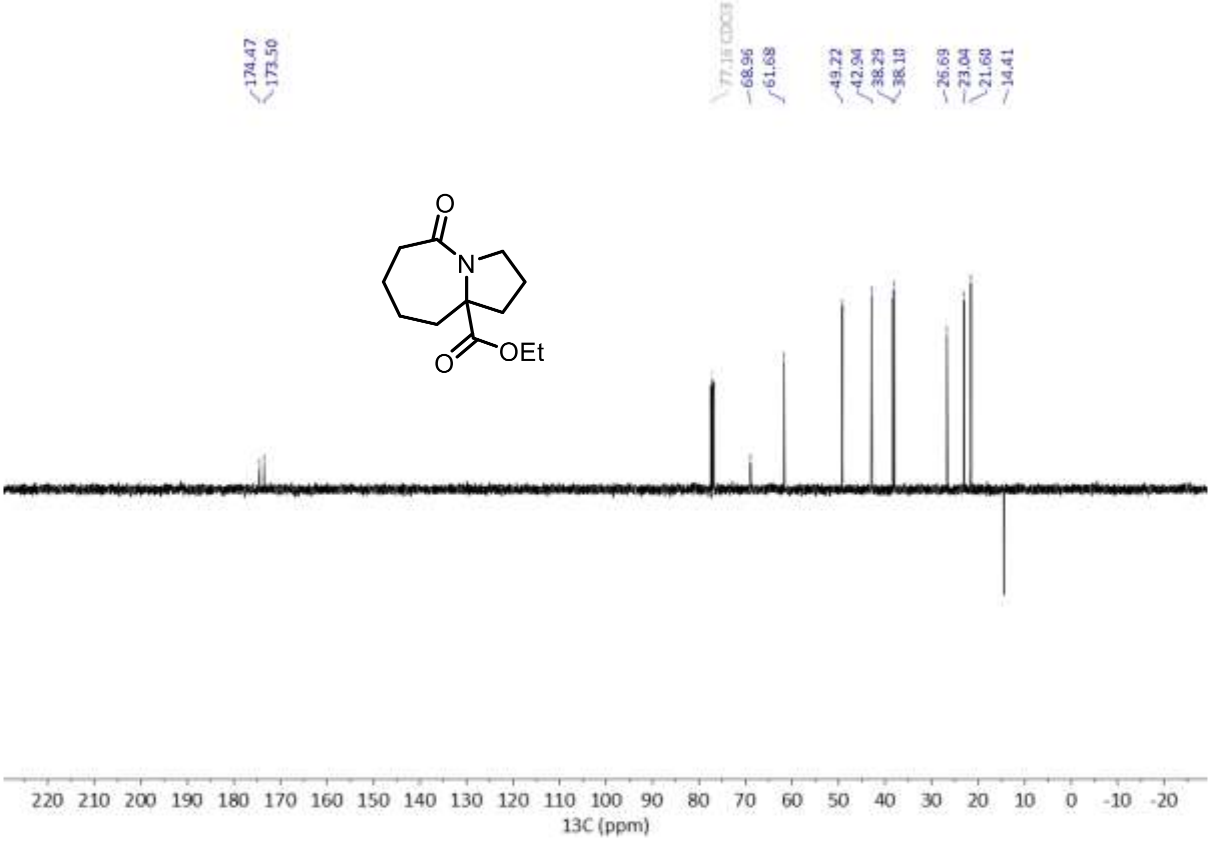

Compound S1-1 (^{1}H)

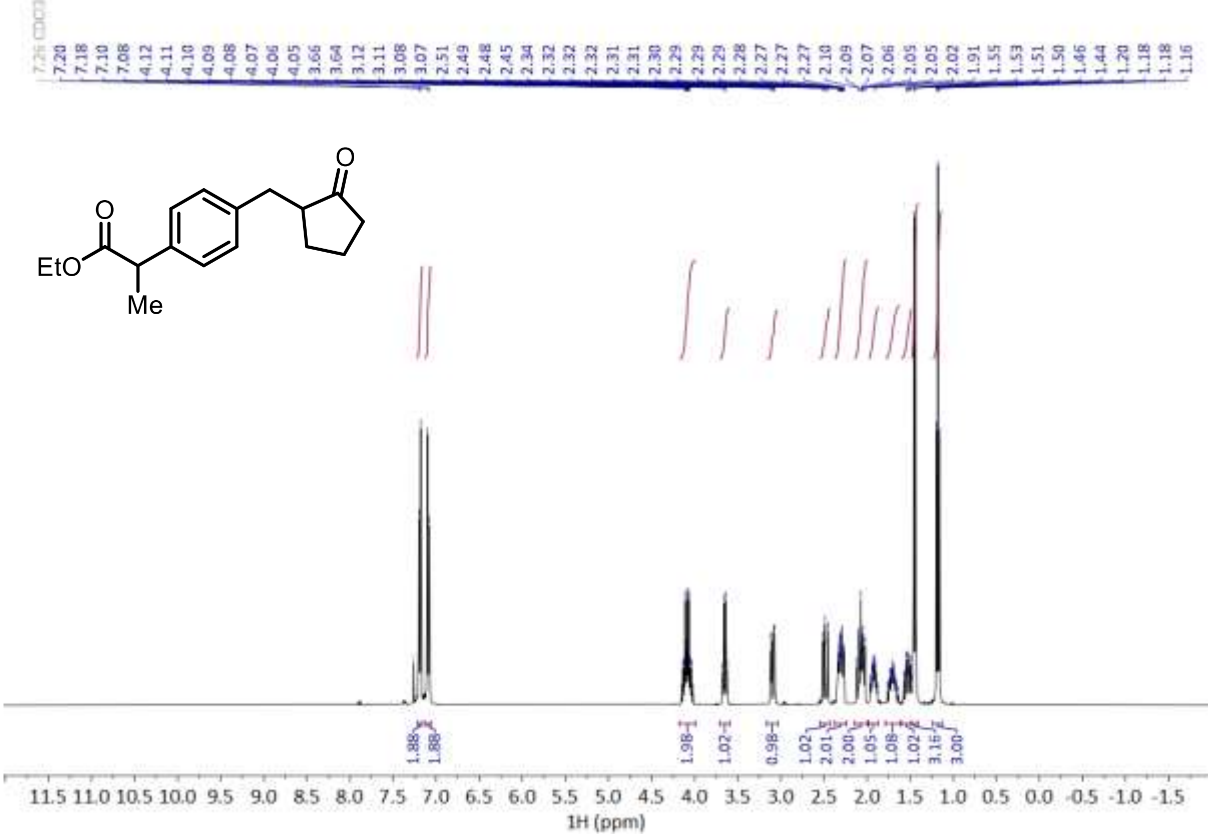

Compound S1-1 (^{13}C)

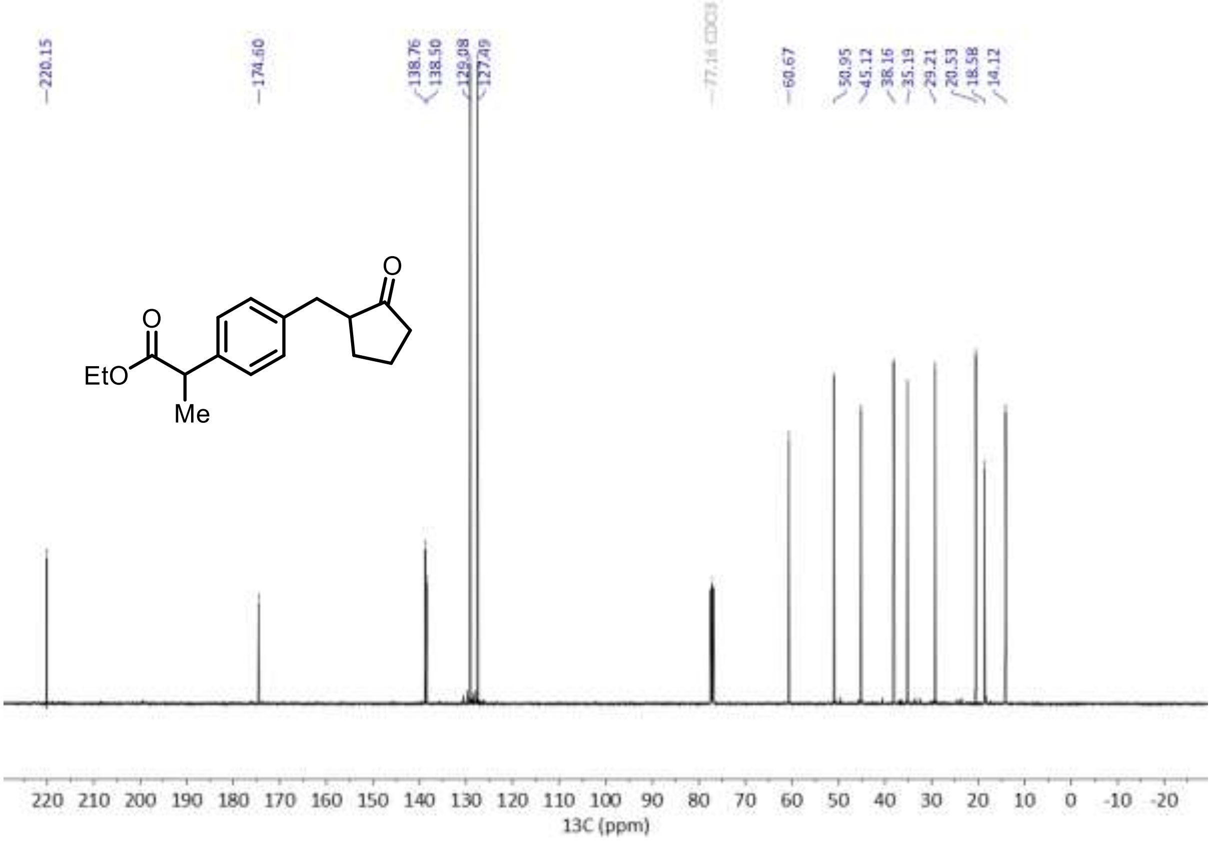

Compound 4f (1H)

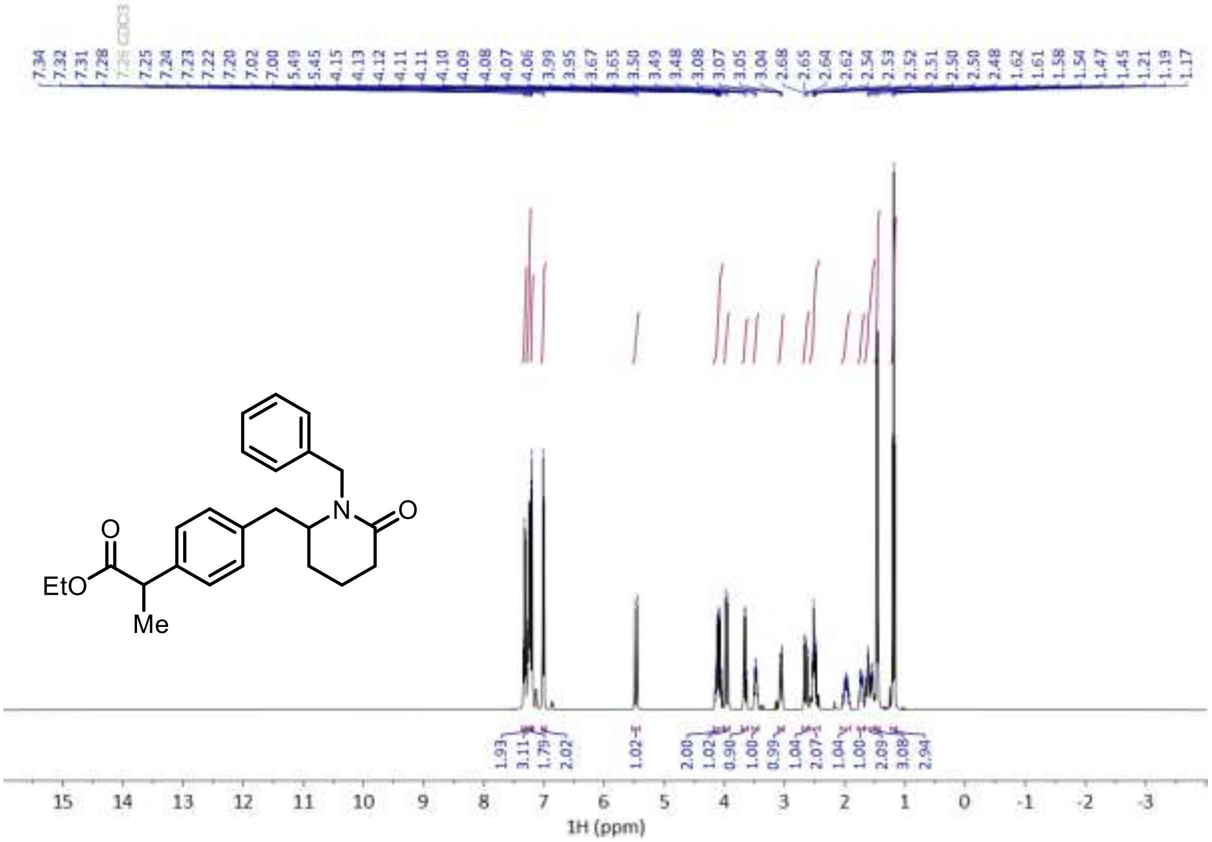

Compound 4f (13C)

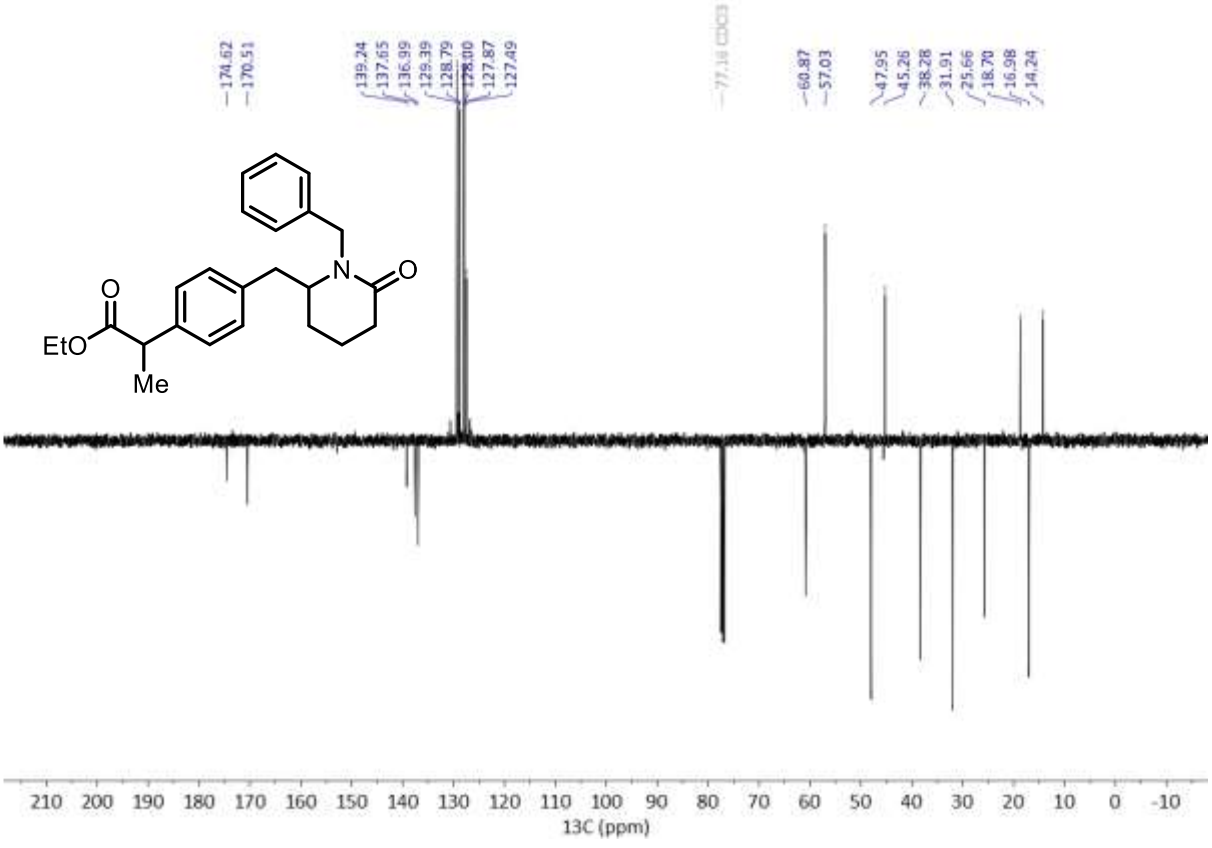

Compound S1-2 (^{1}H)

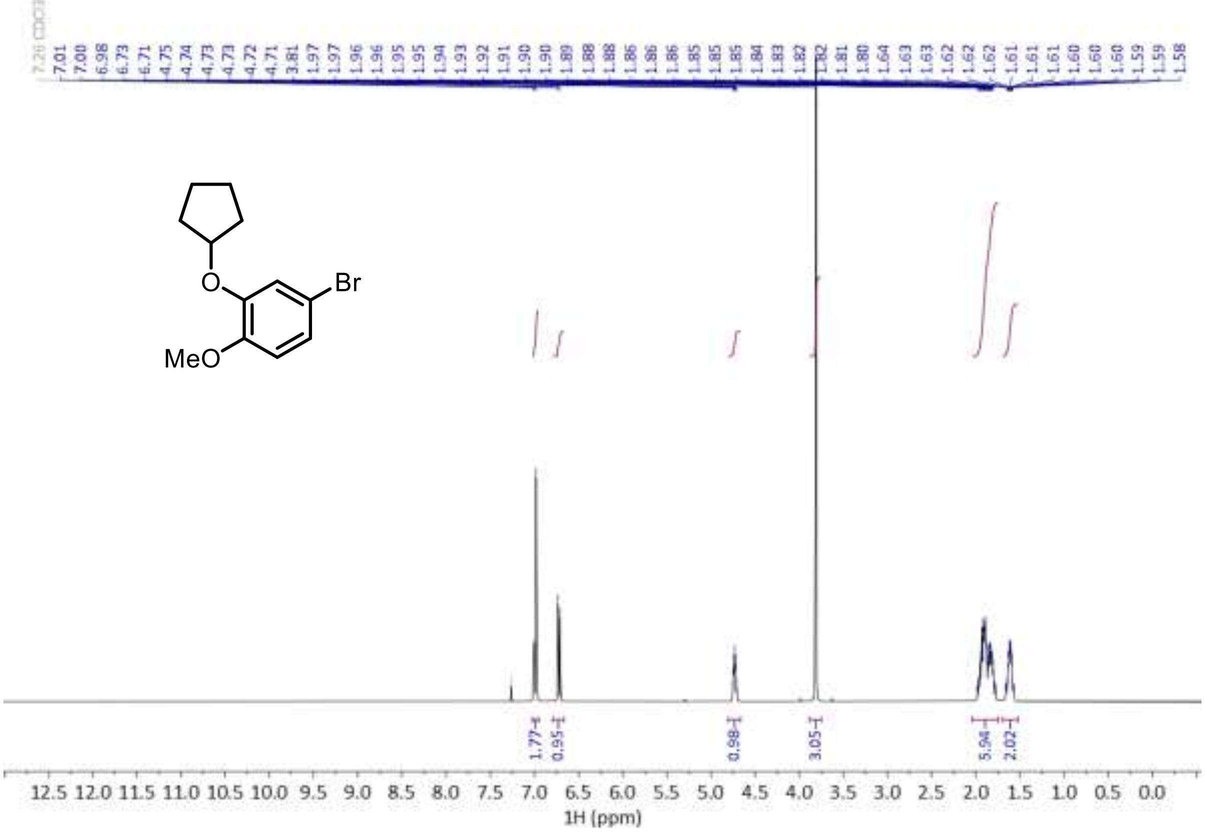

Compound S1-2 (^{13}C)

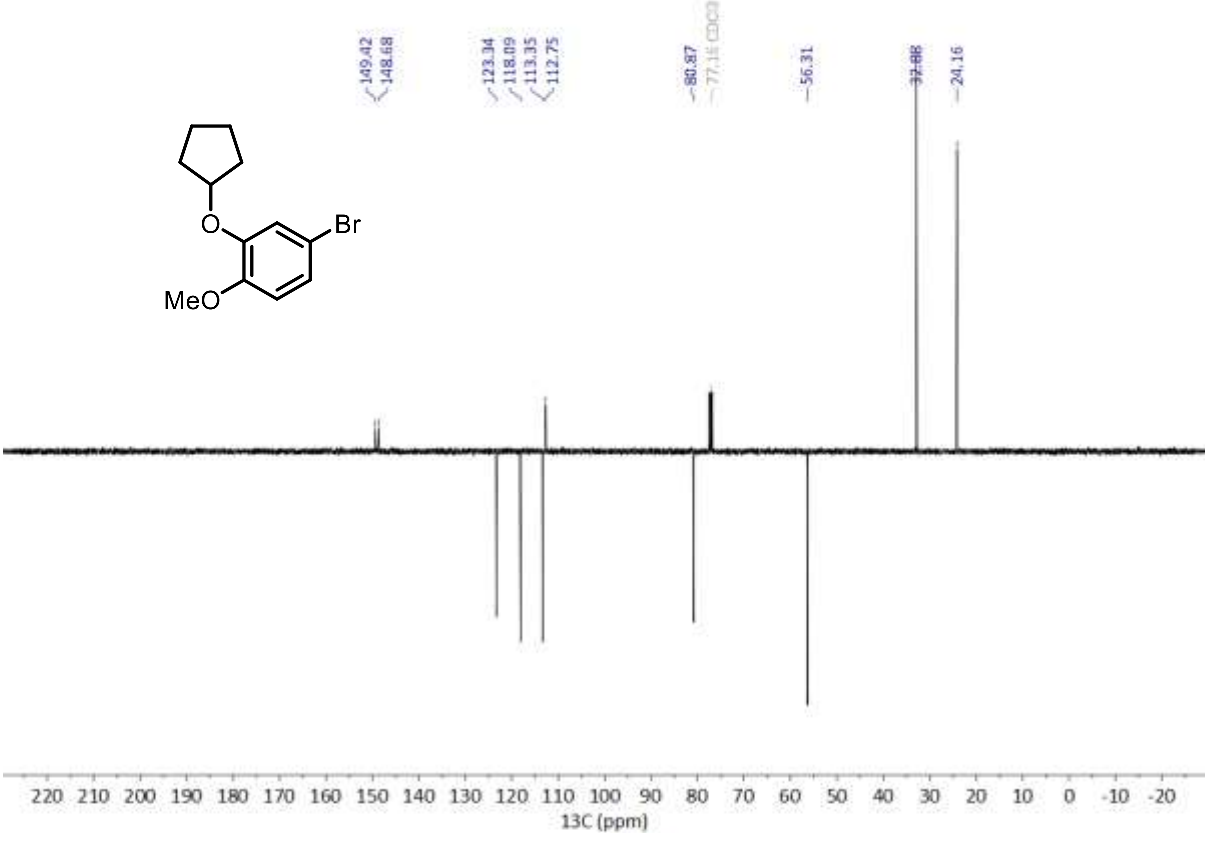

Compound S1-3 (^{1}H)

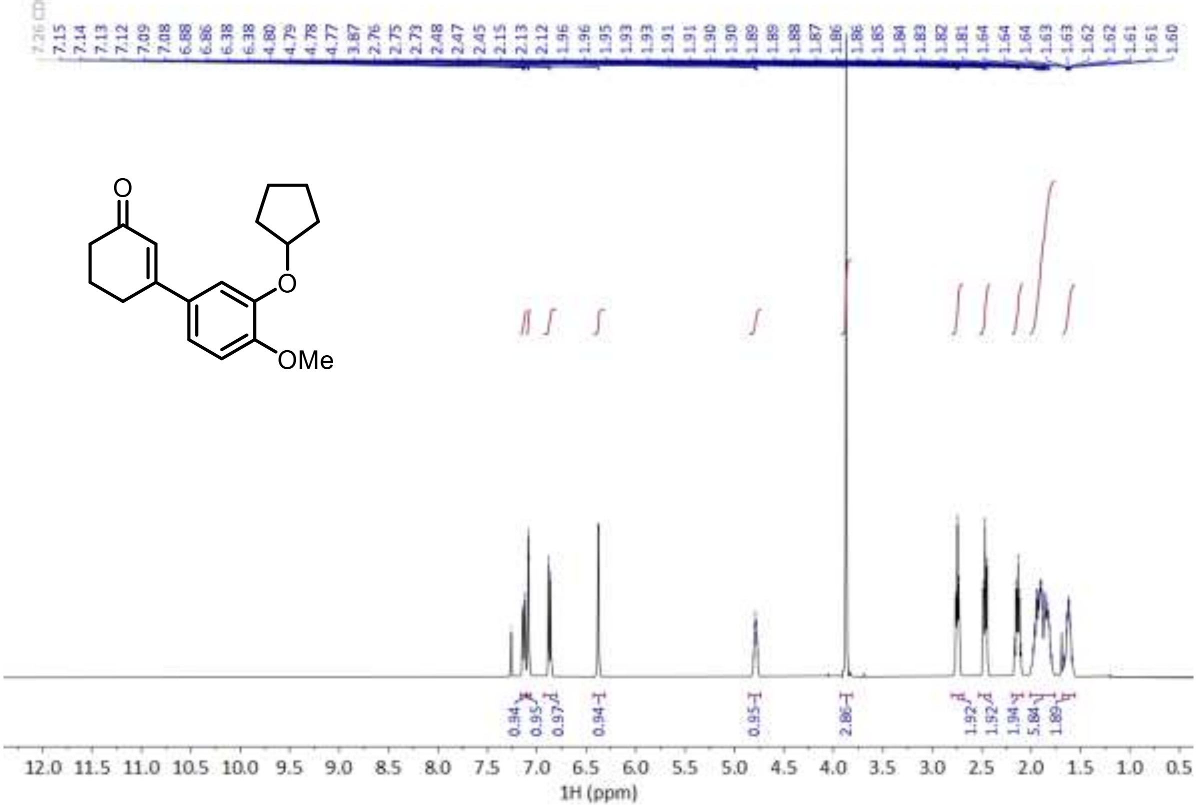

Compound S1-3 (^{13}C)

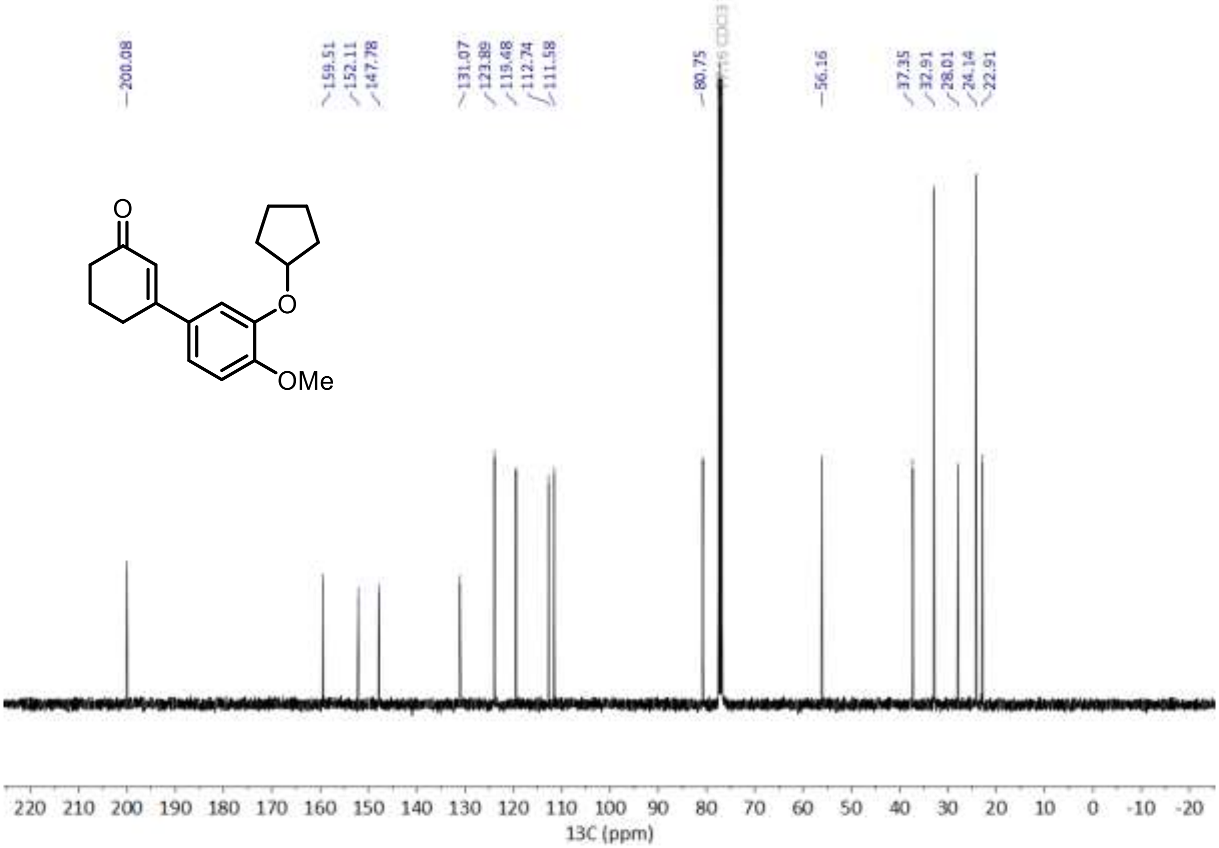

Compound 4h (^{1}H)

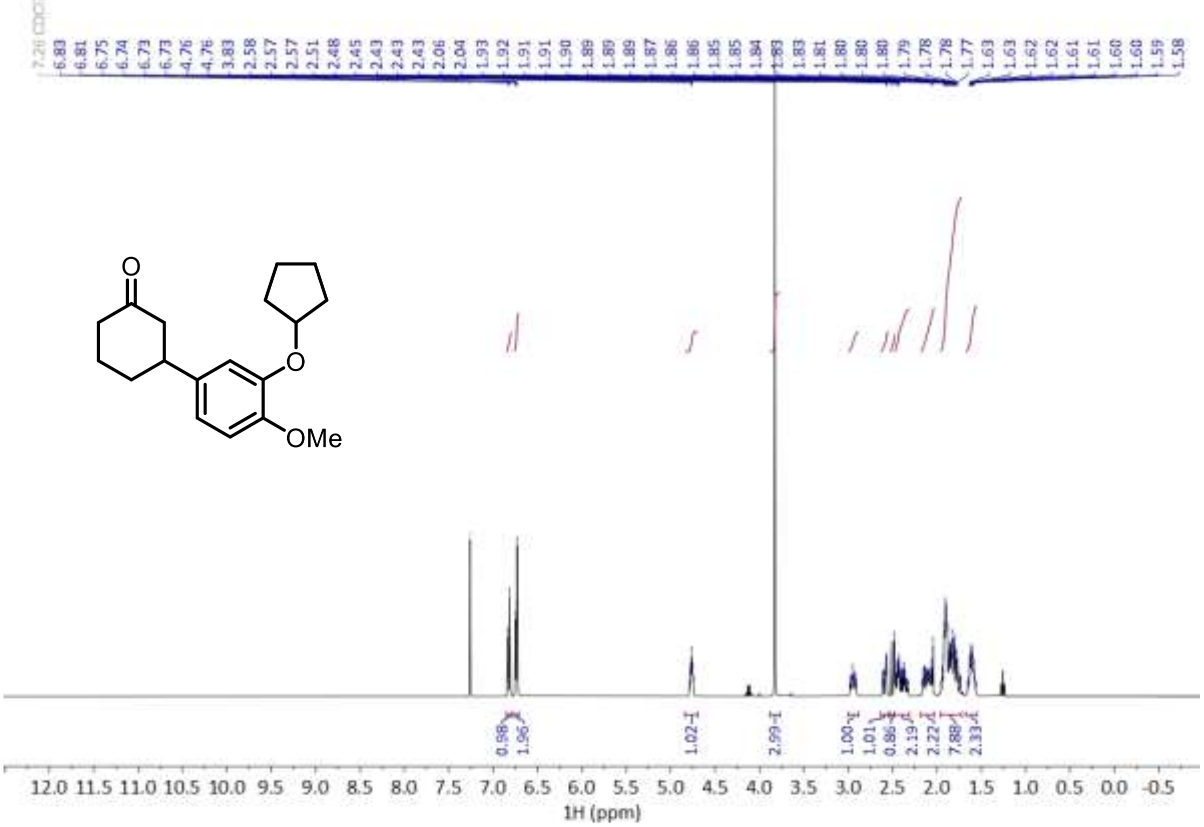

Compound 4h (^{13}C)

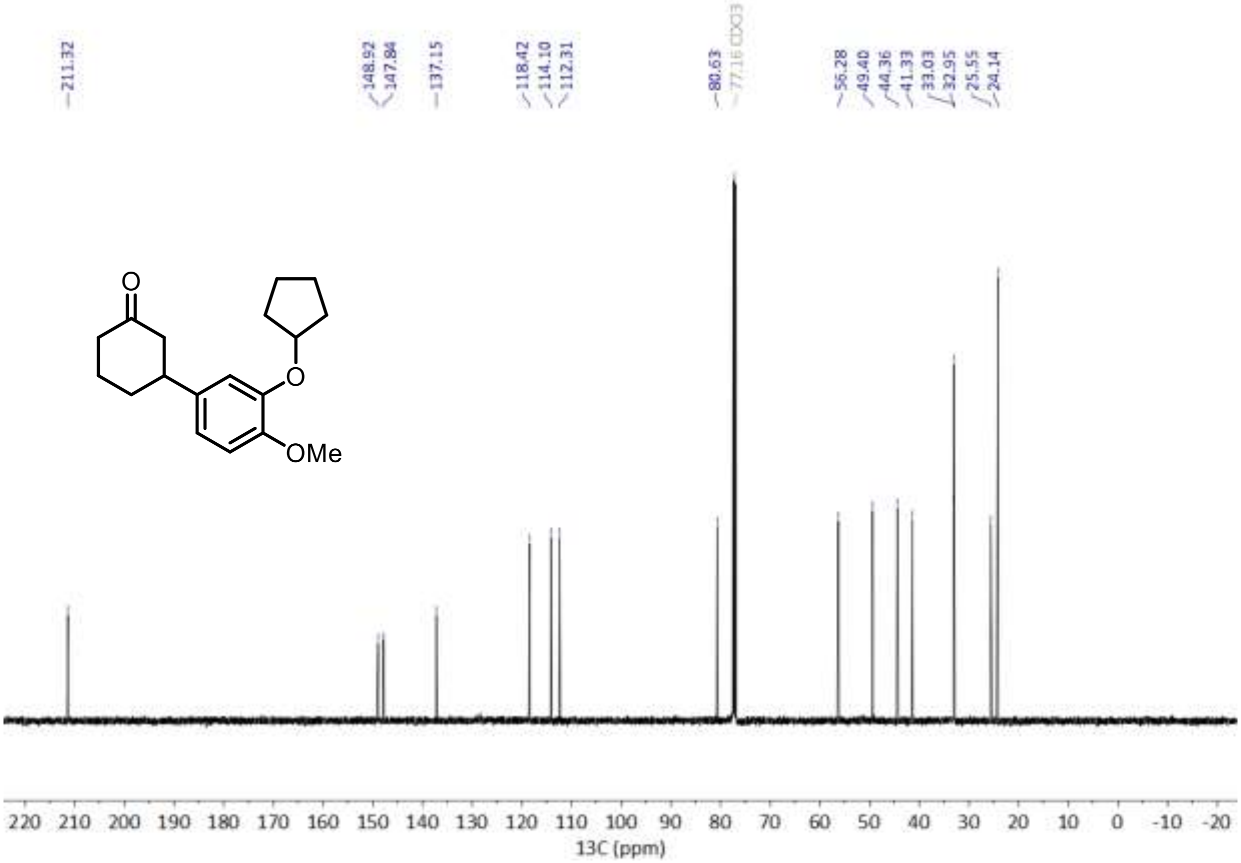

Compound S1-4 (^{1}H)

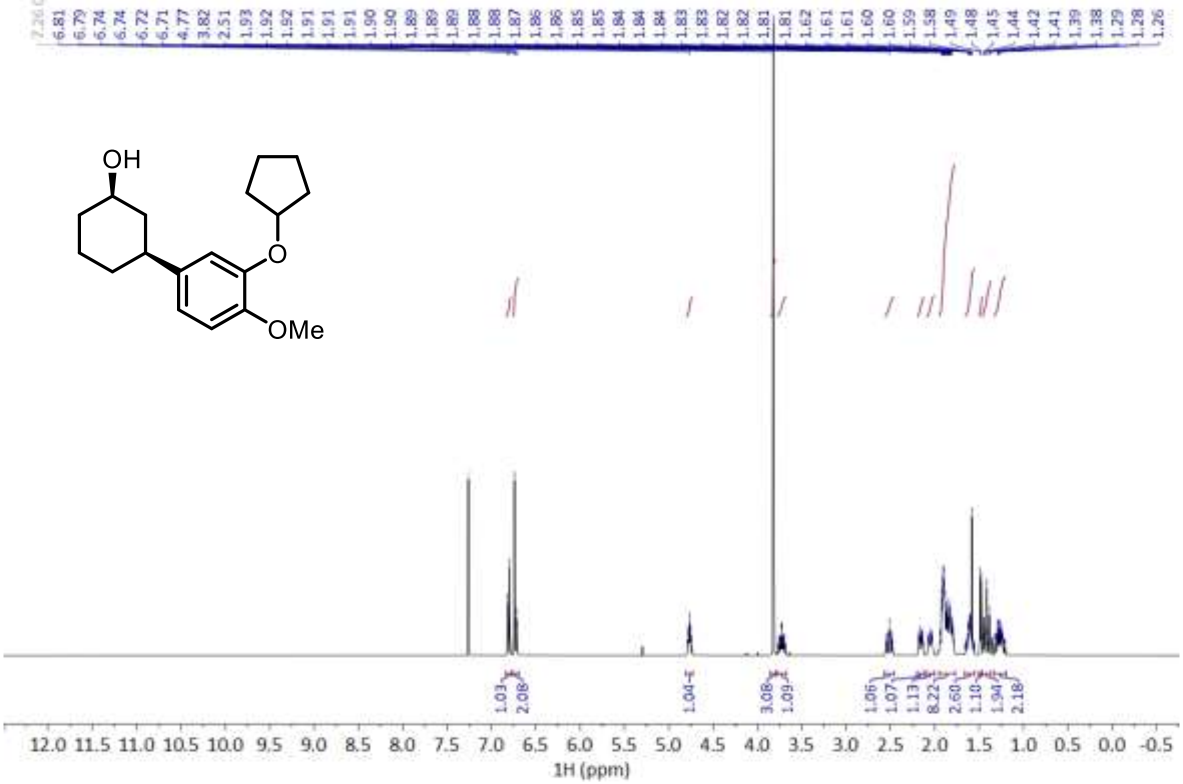

Compound S1-4 (^{13}C)

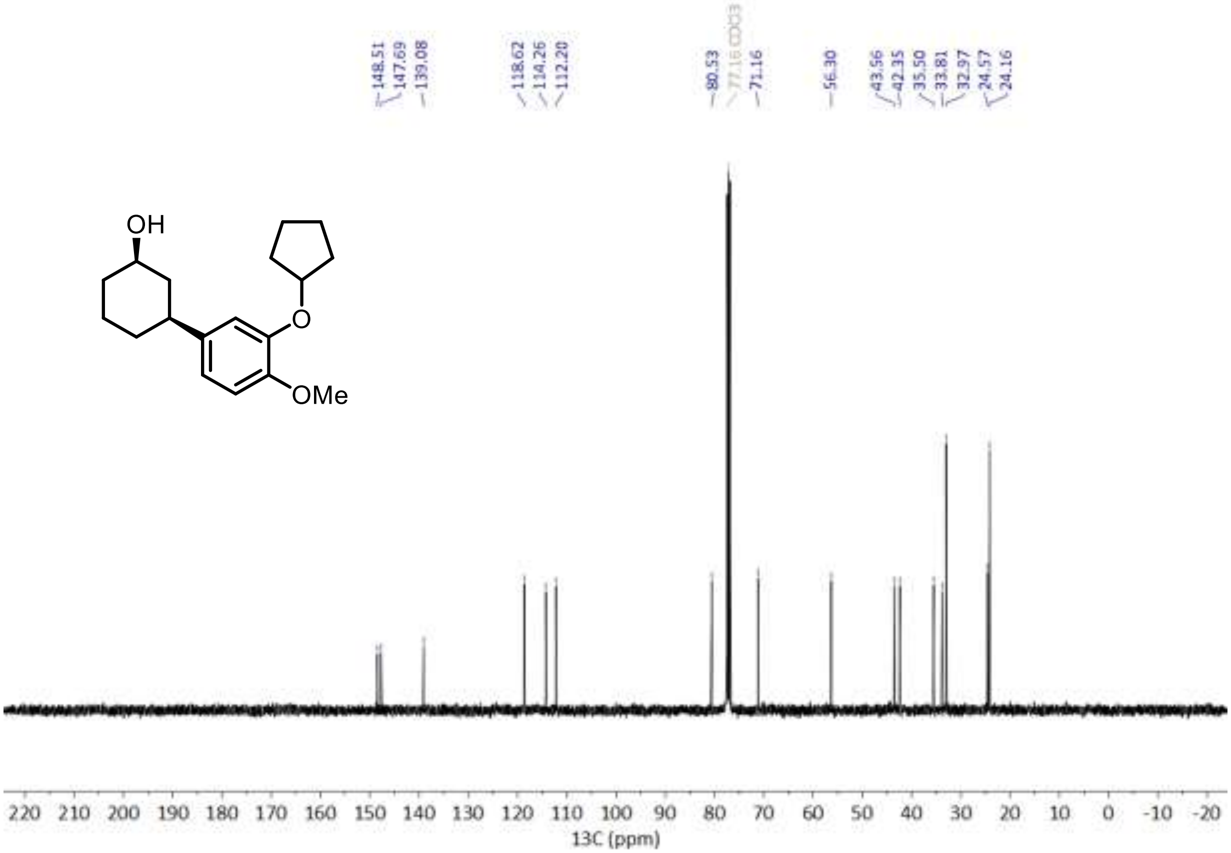

Compound S1-5 (^{1}H)

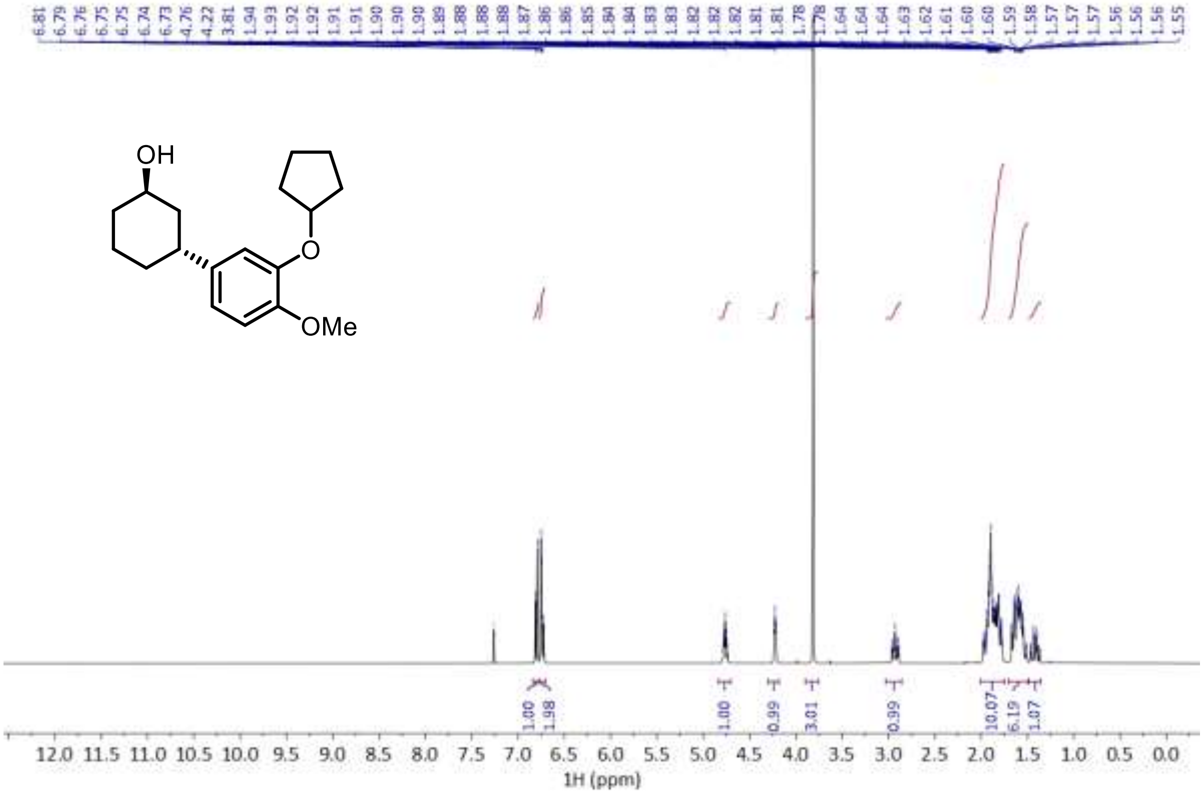

Compound S1-5 (^{13}C)

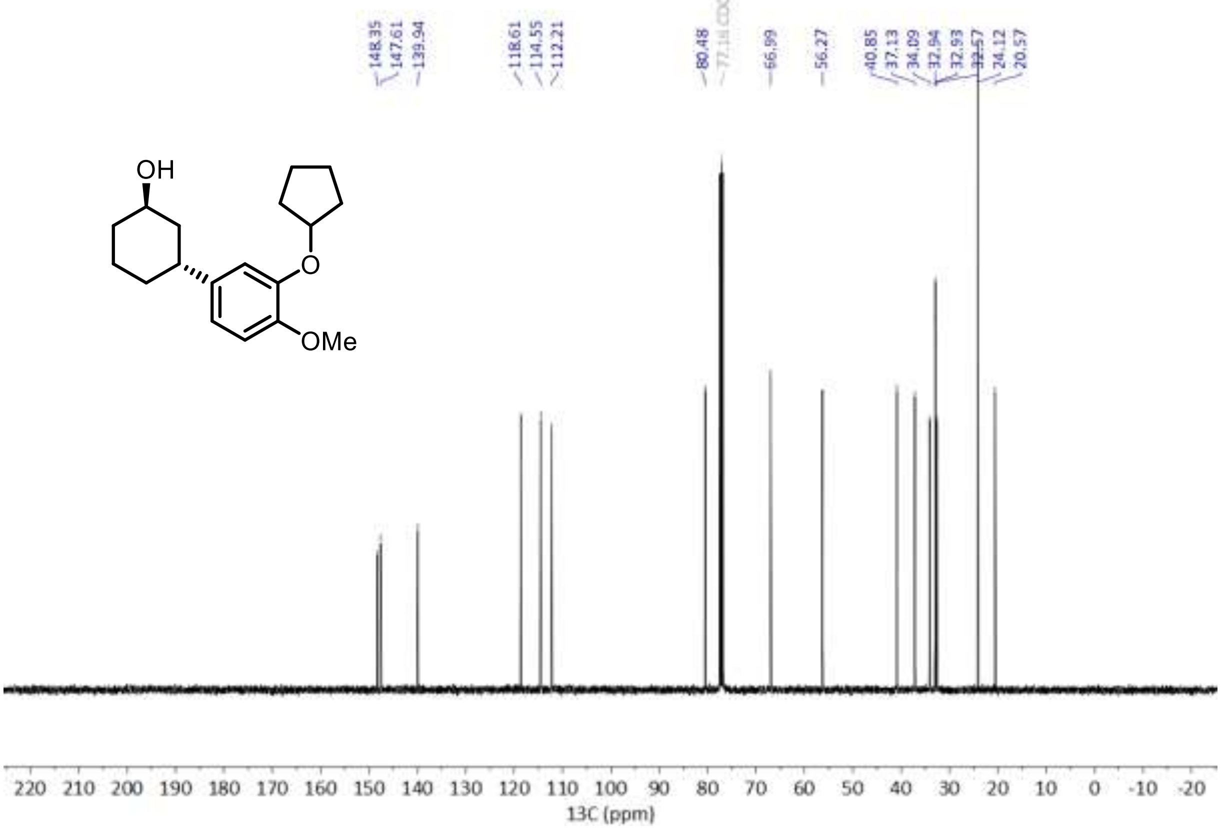

Compound 4i (^{1}H)

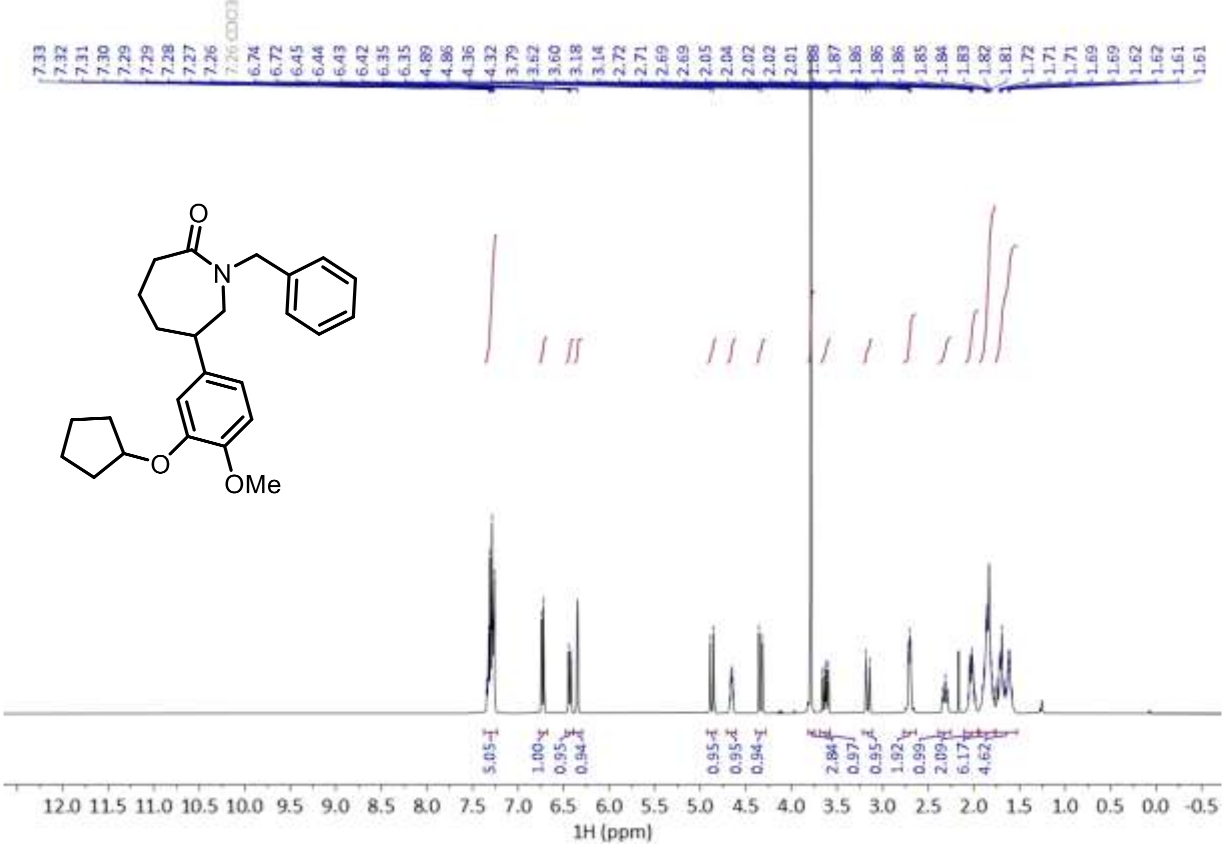

Compound 4i (^{13}C)

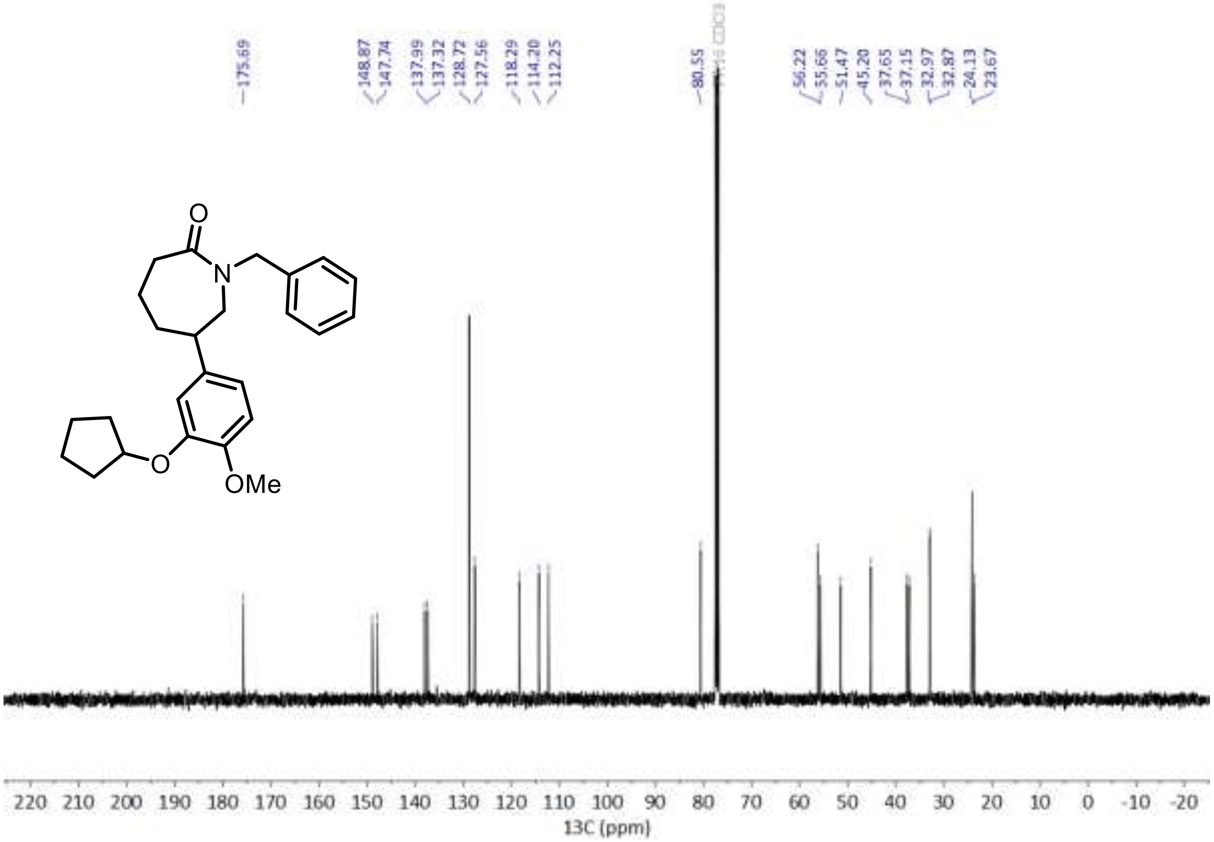

Compound 4l (^{1}H)

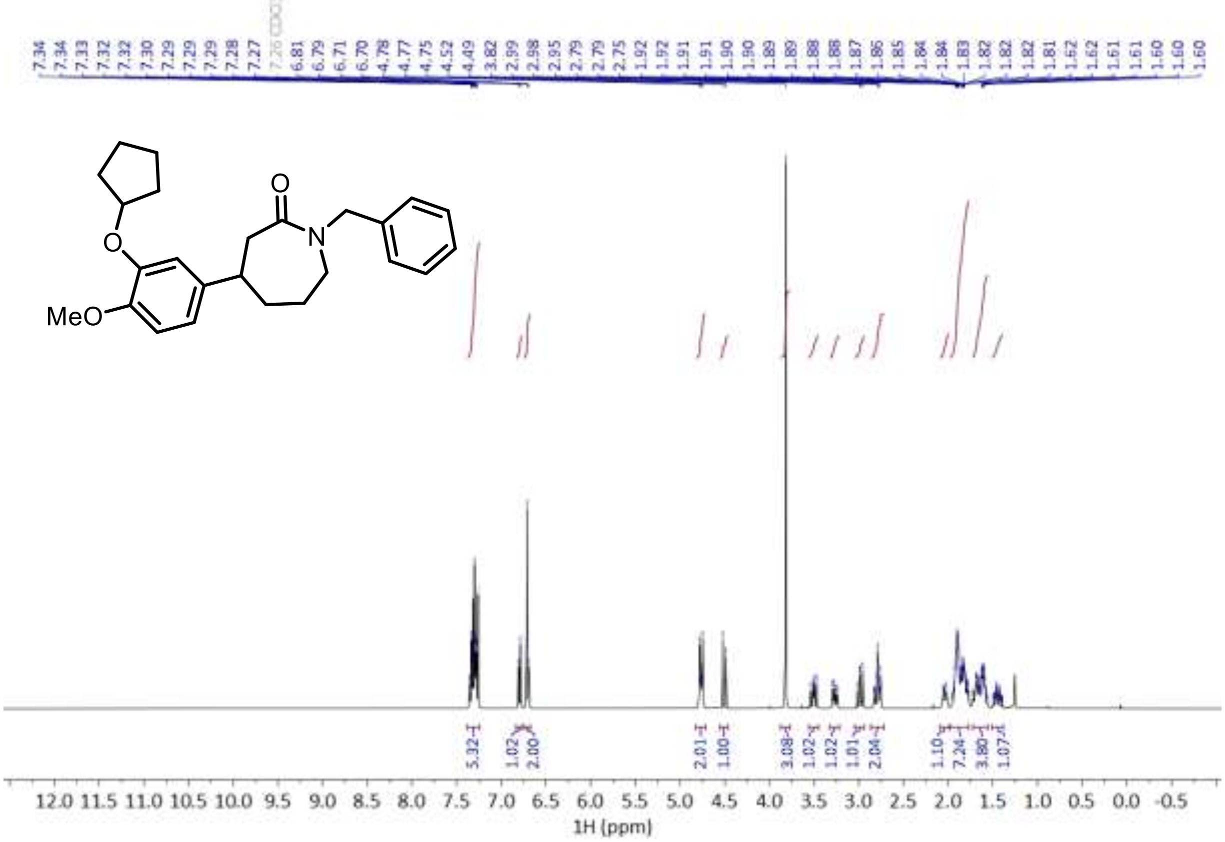

Compound 4l (^{13}C)

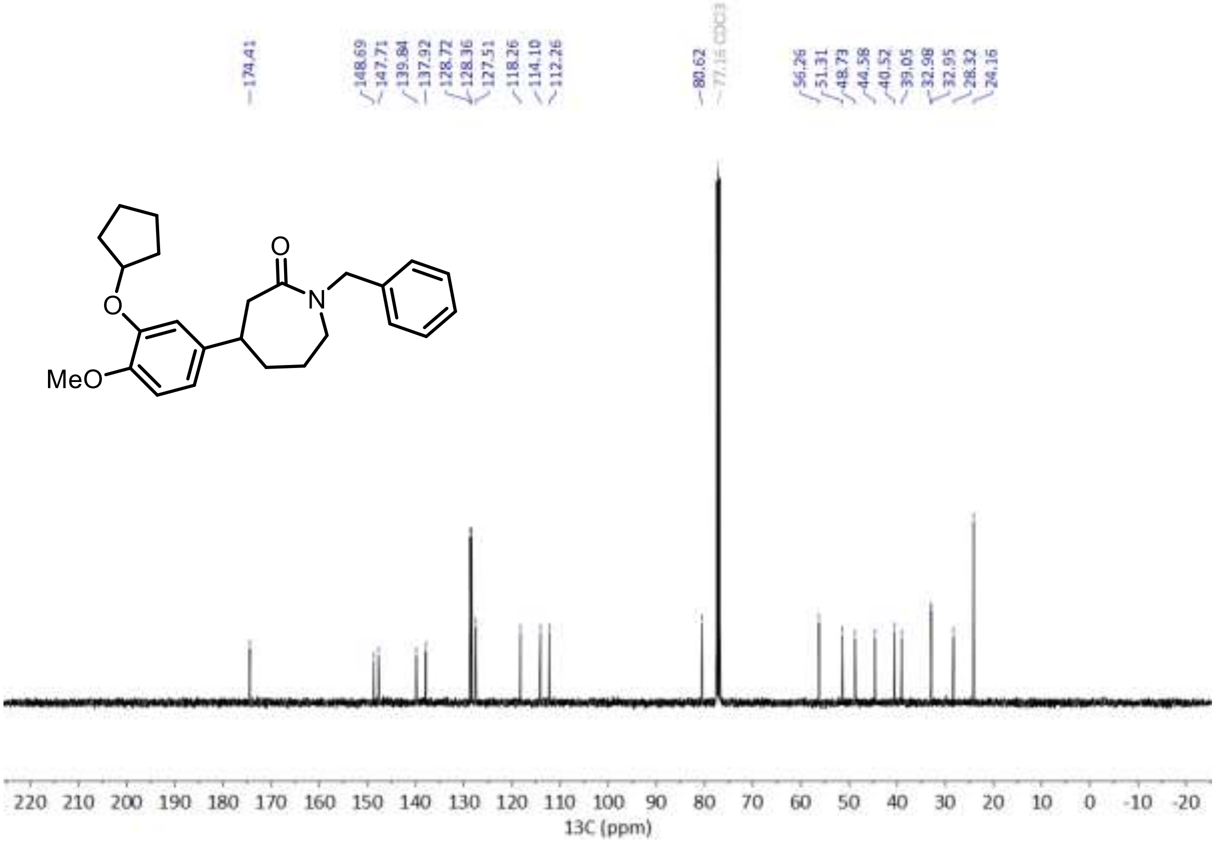

Compound S1-6 (^{1}H)

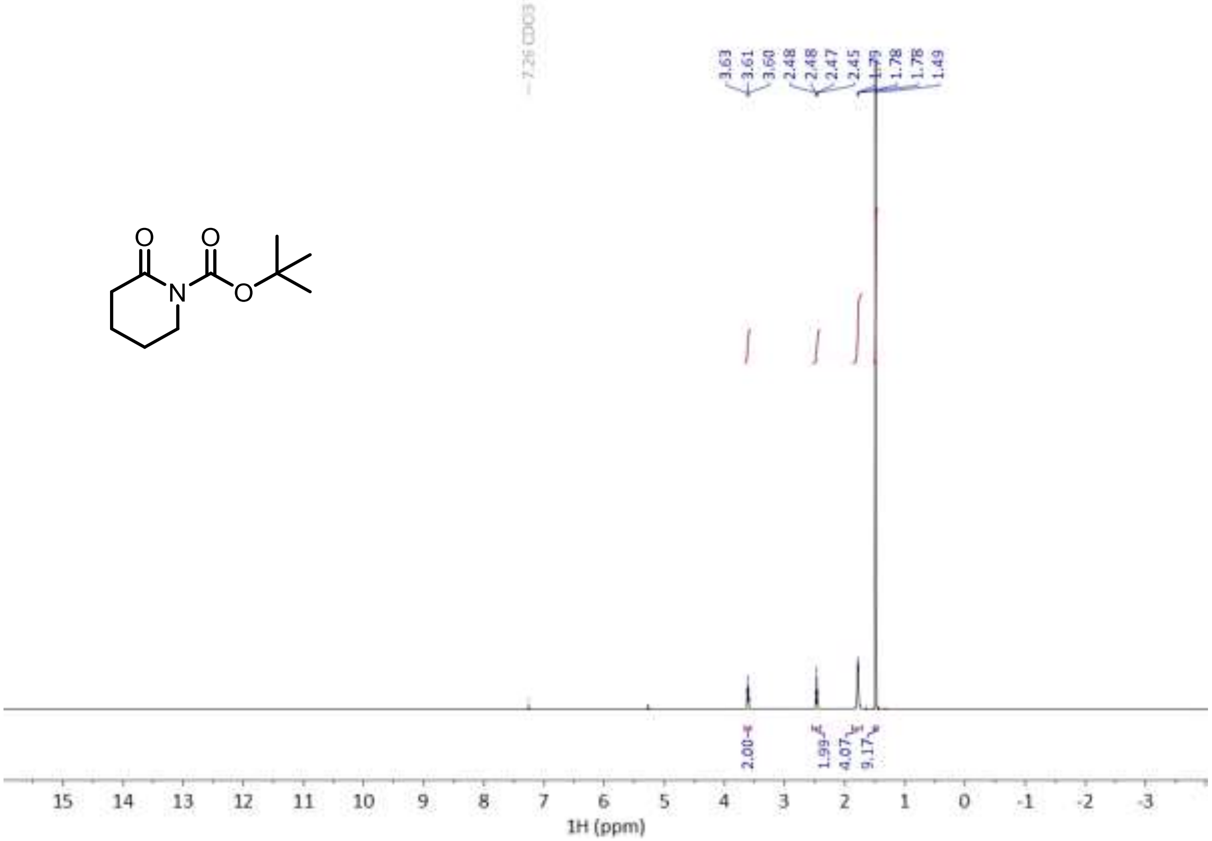

Compound S1-6 (^{13}C)

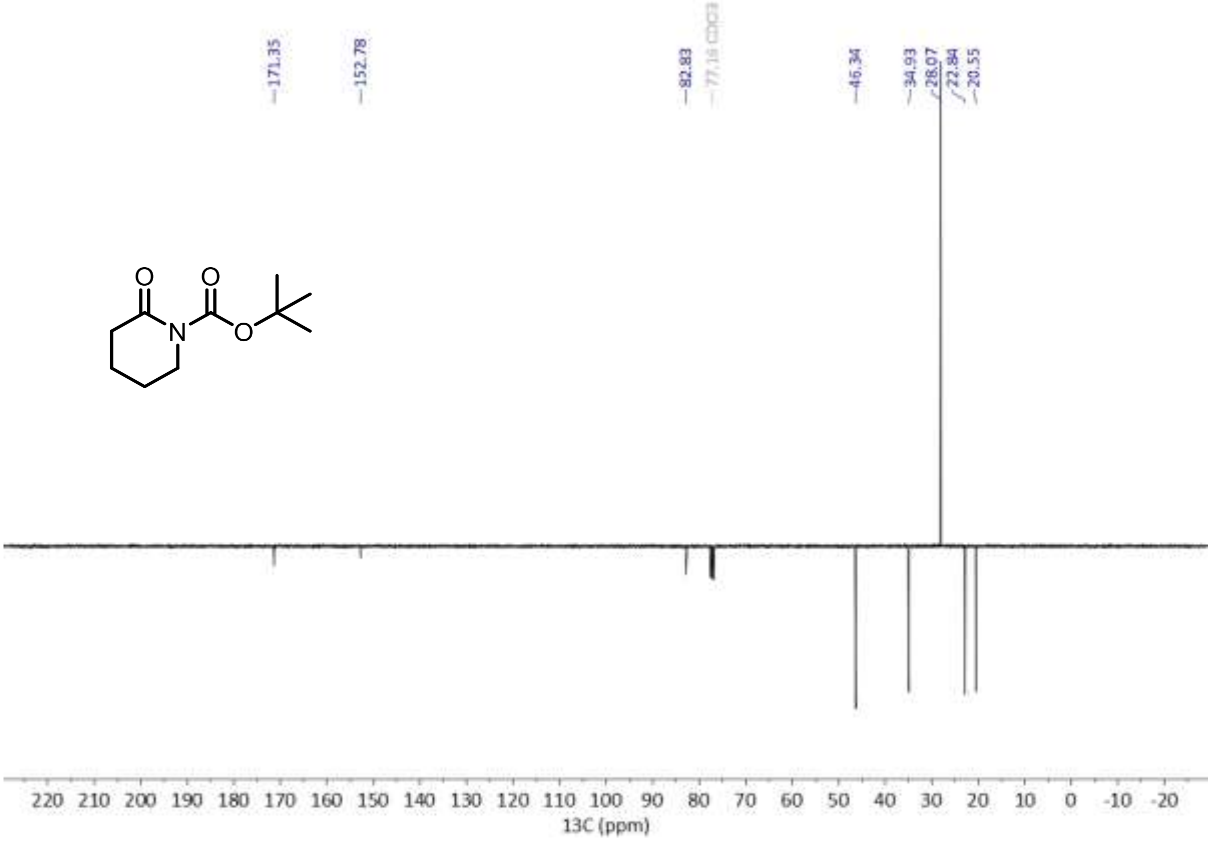

Compound S1-7 (^{1}H)

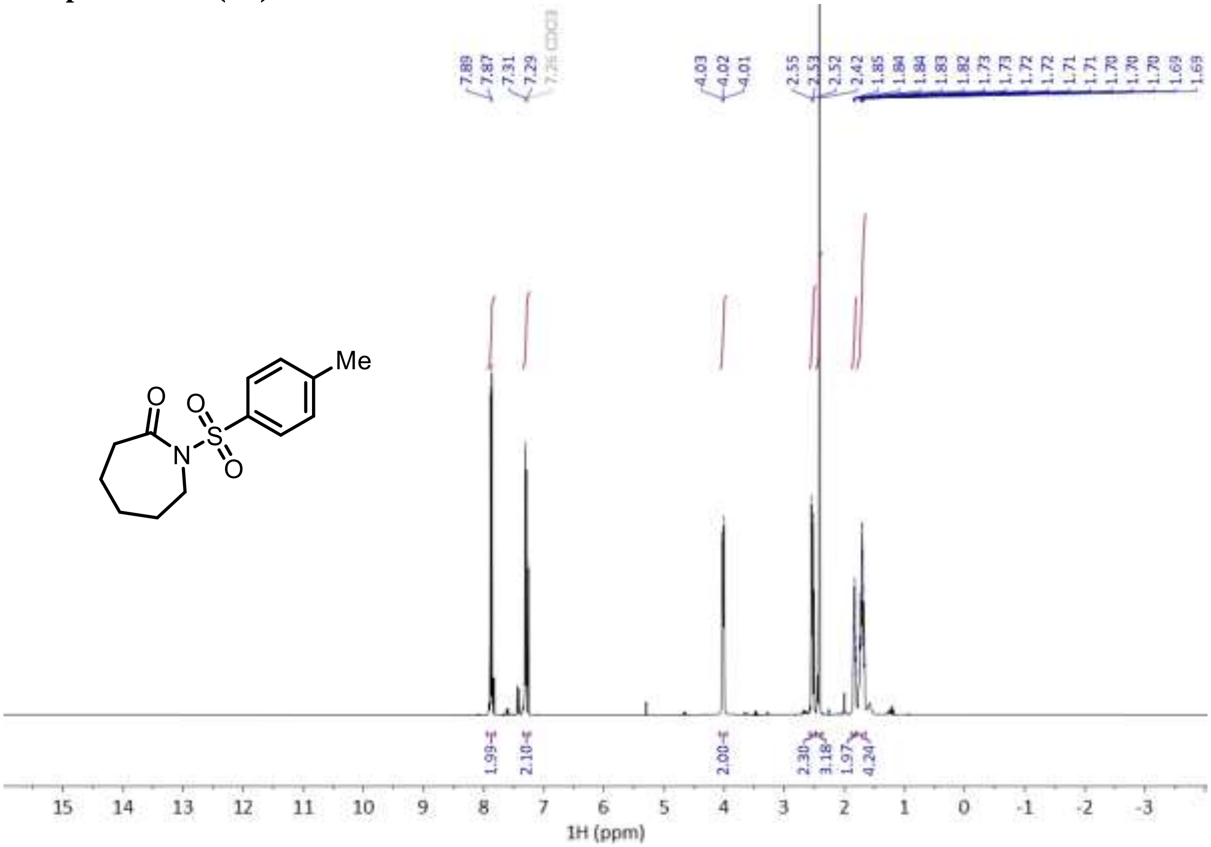

Compound S1-7 (^{13}C)

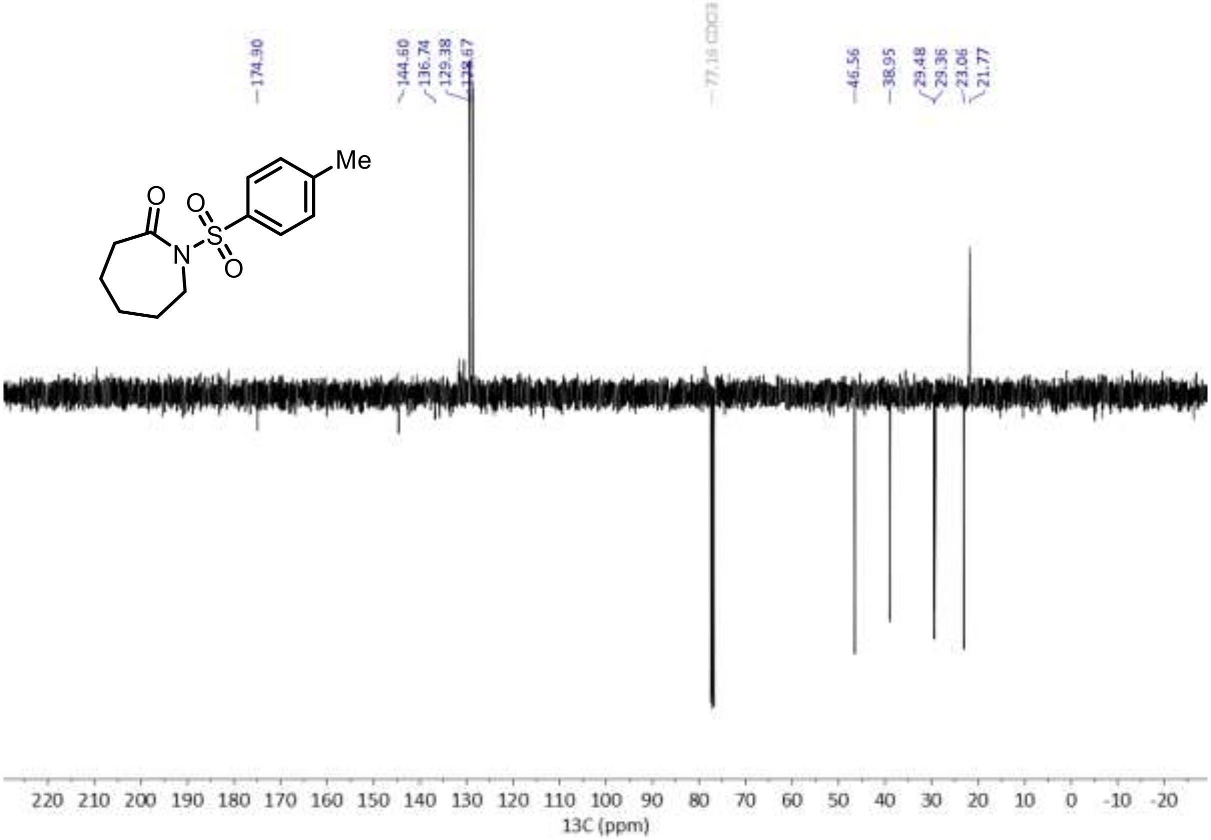

Compound 5j (^{1}H)

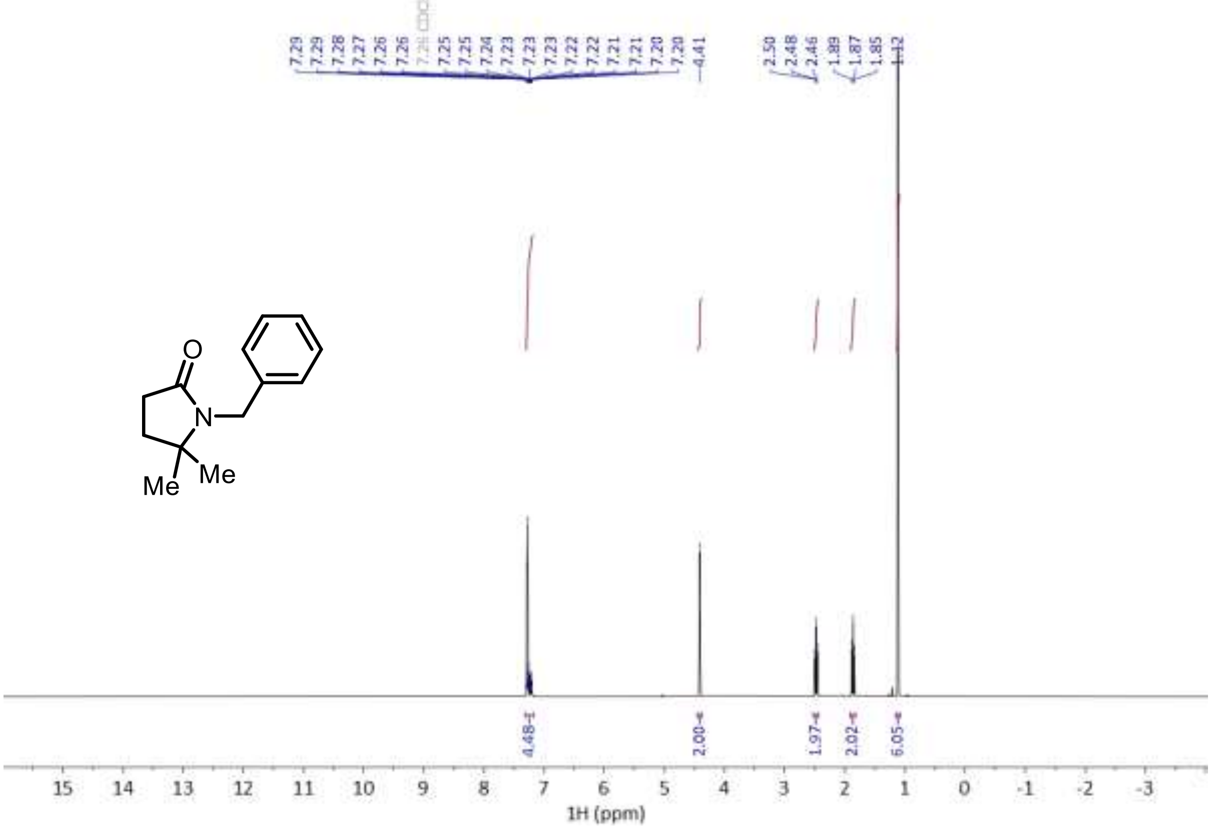

Compound 5j (^{13}C)

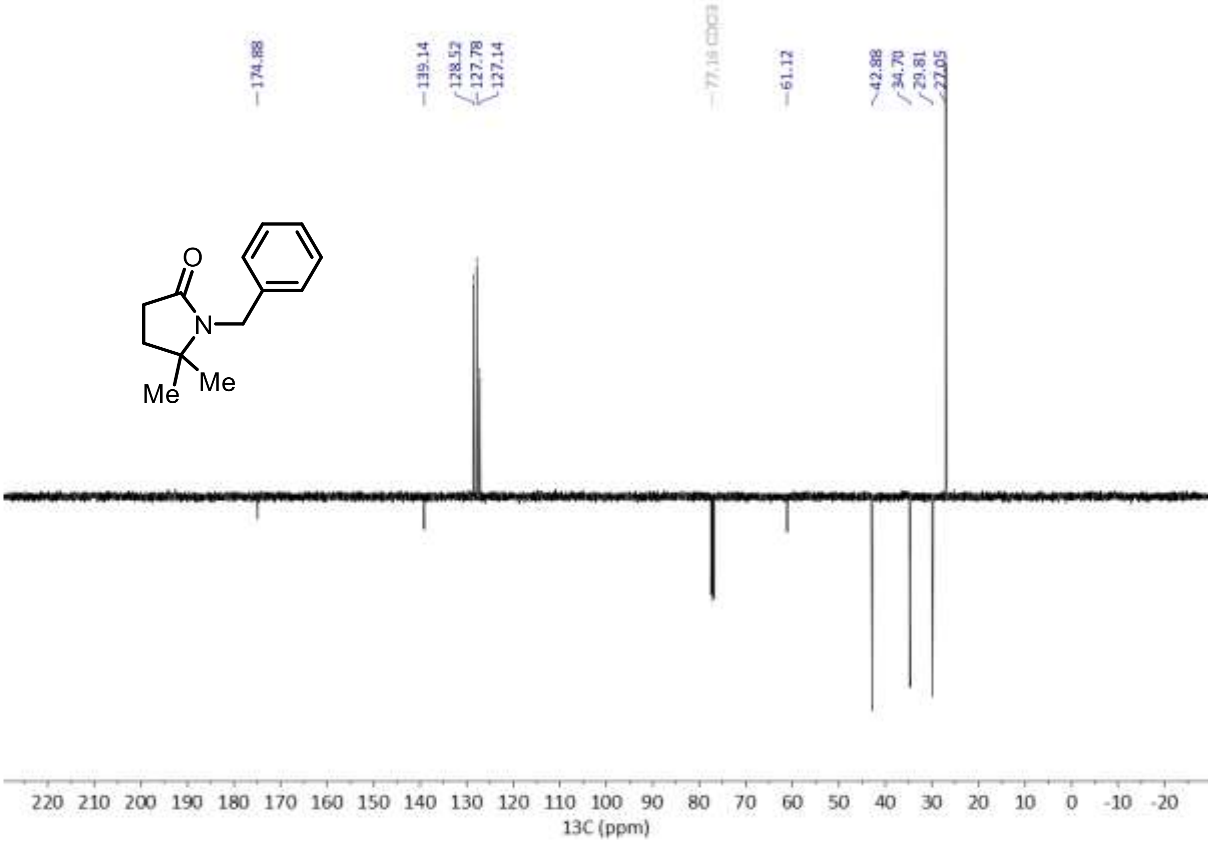

Compound 5a (^{1}H)

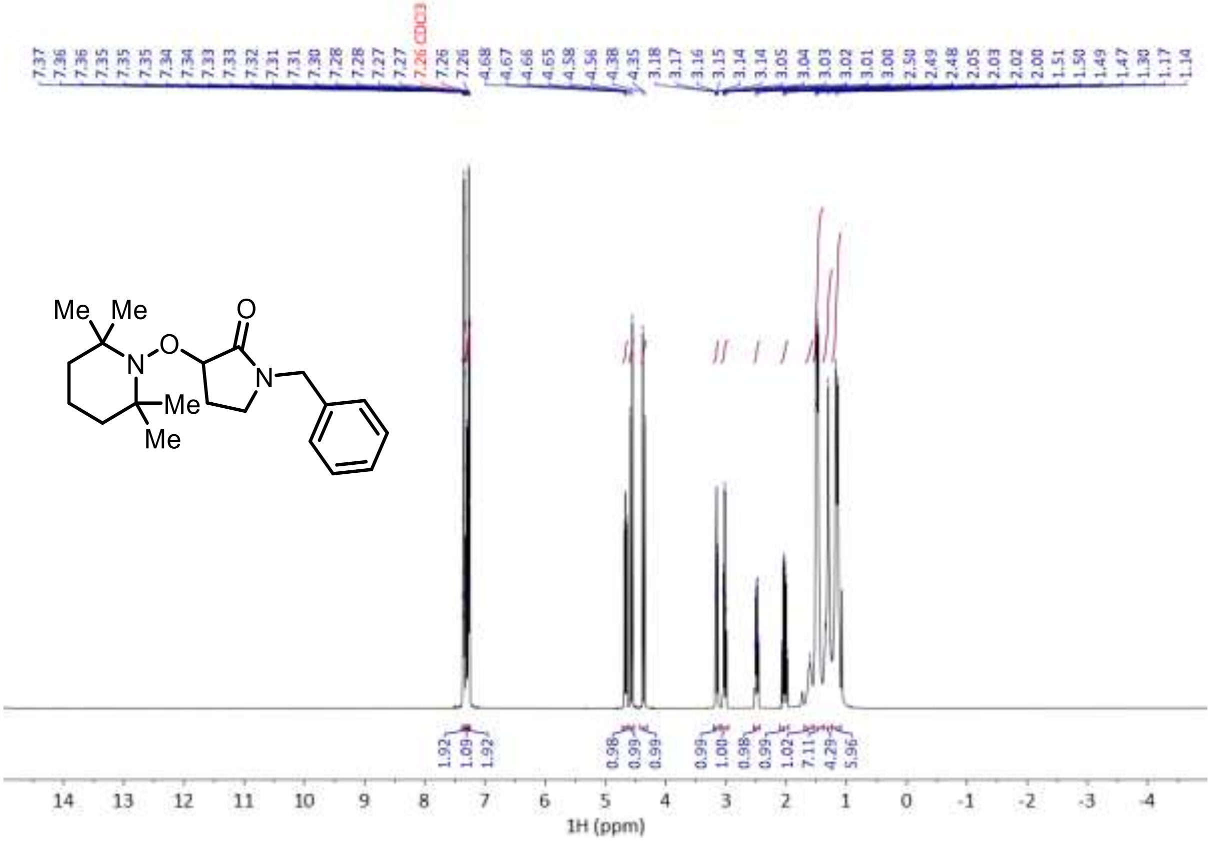

Compound 5a (^{13}C)

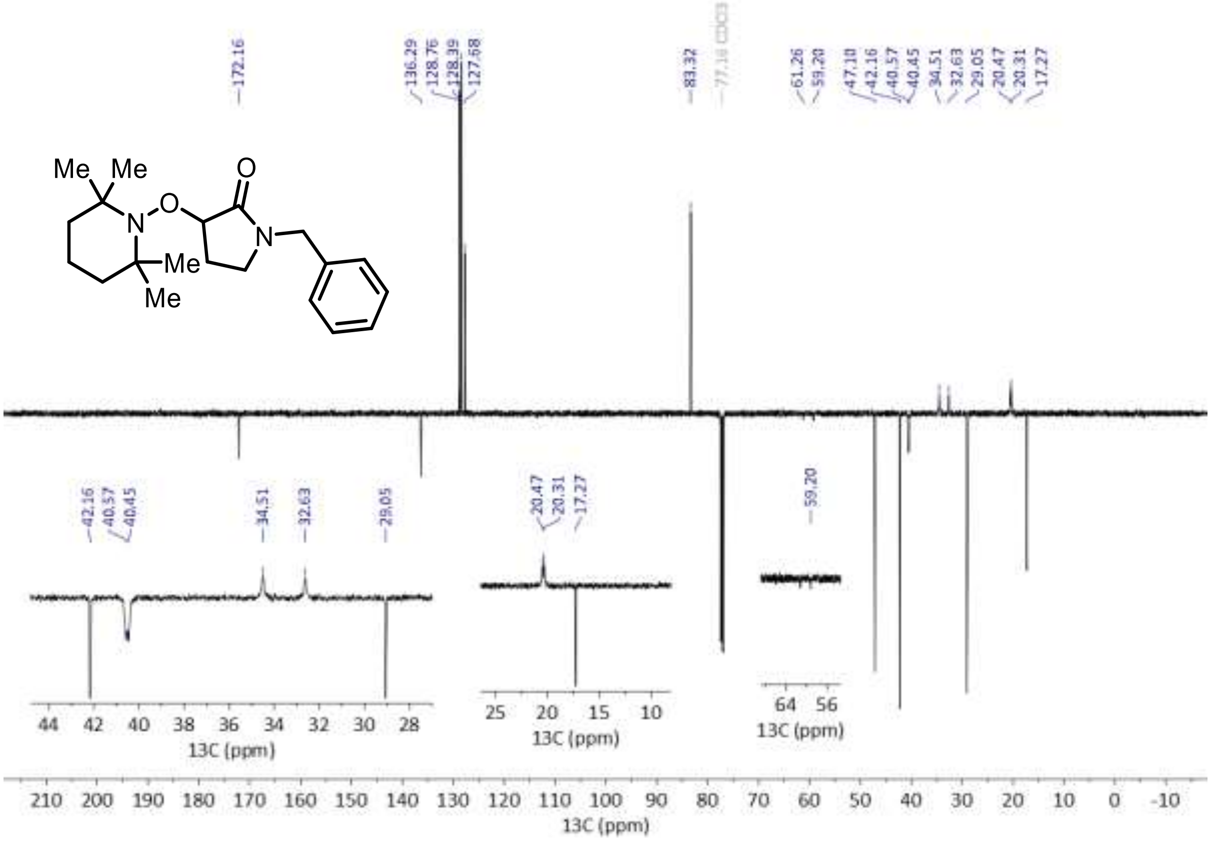

Compound 5k (^{1}H)

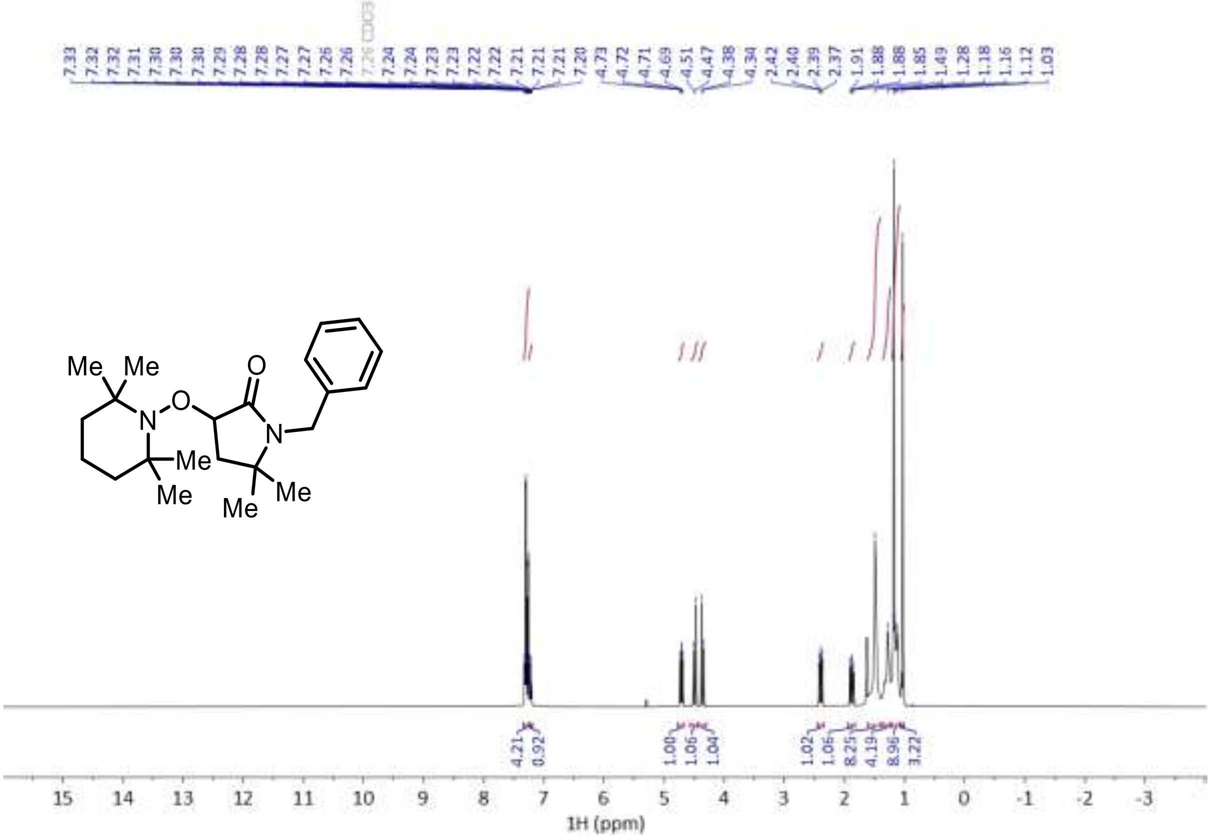

Compound 5k (^{13}C)

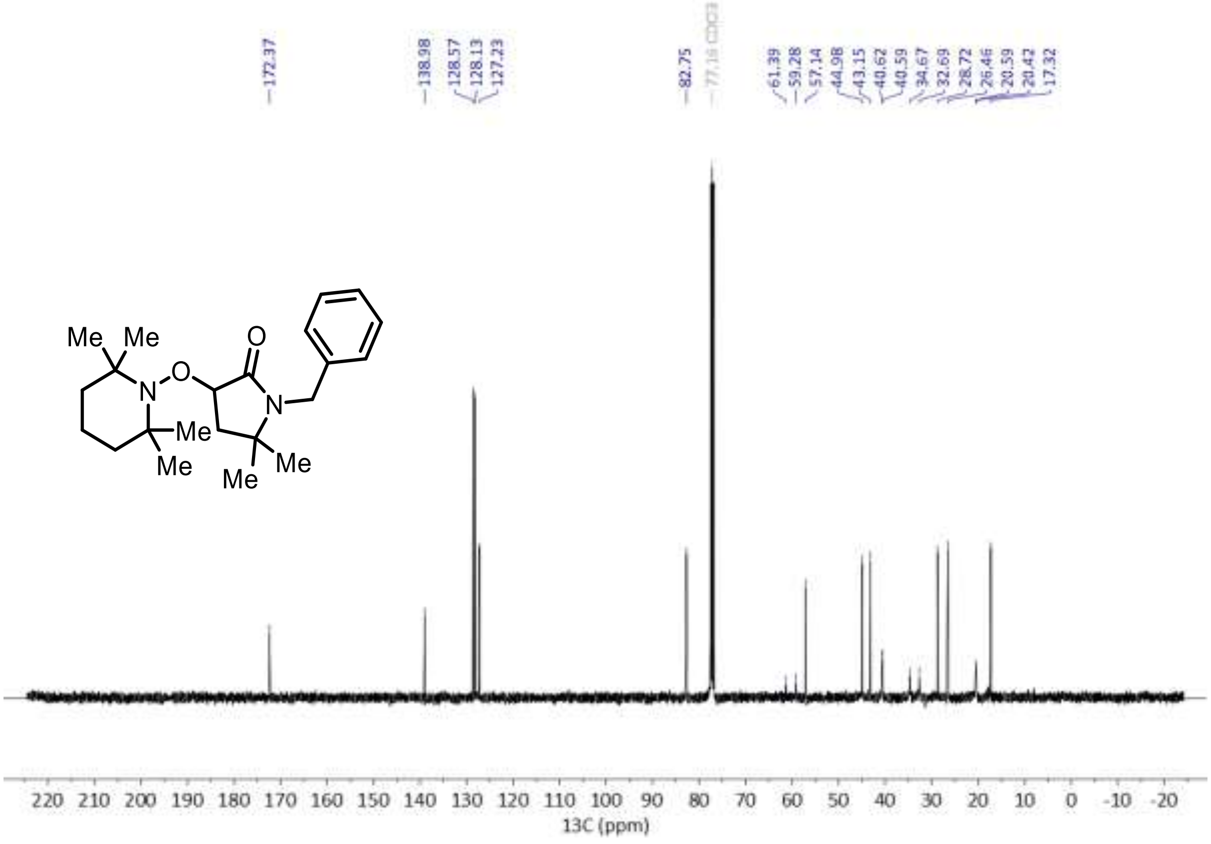

Compound S1-8 (¹H)

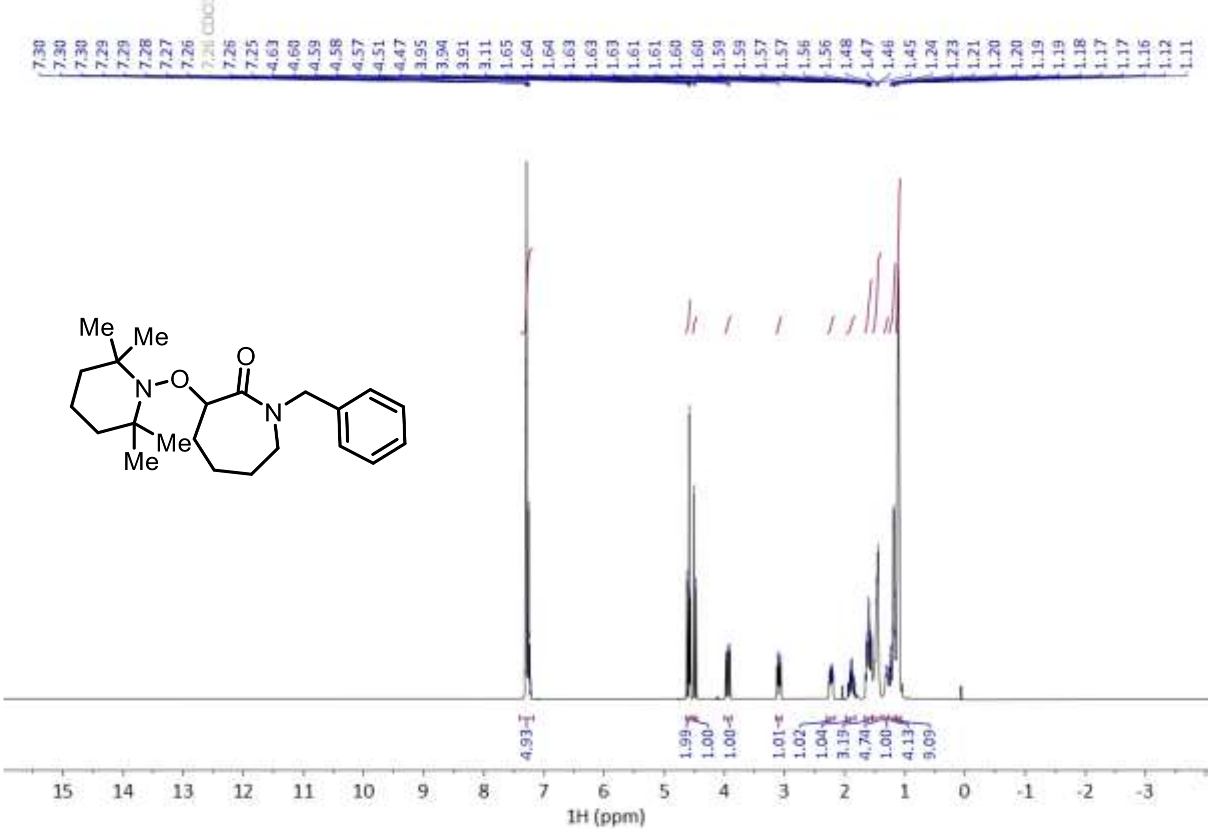

Compound S1-8 (¹³C)

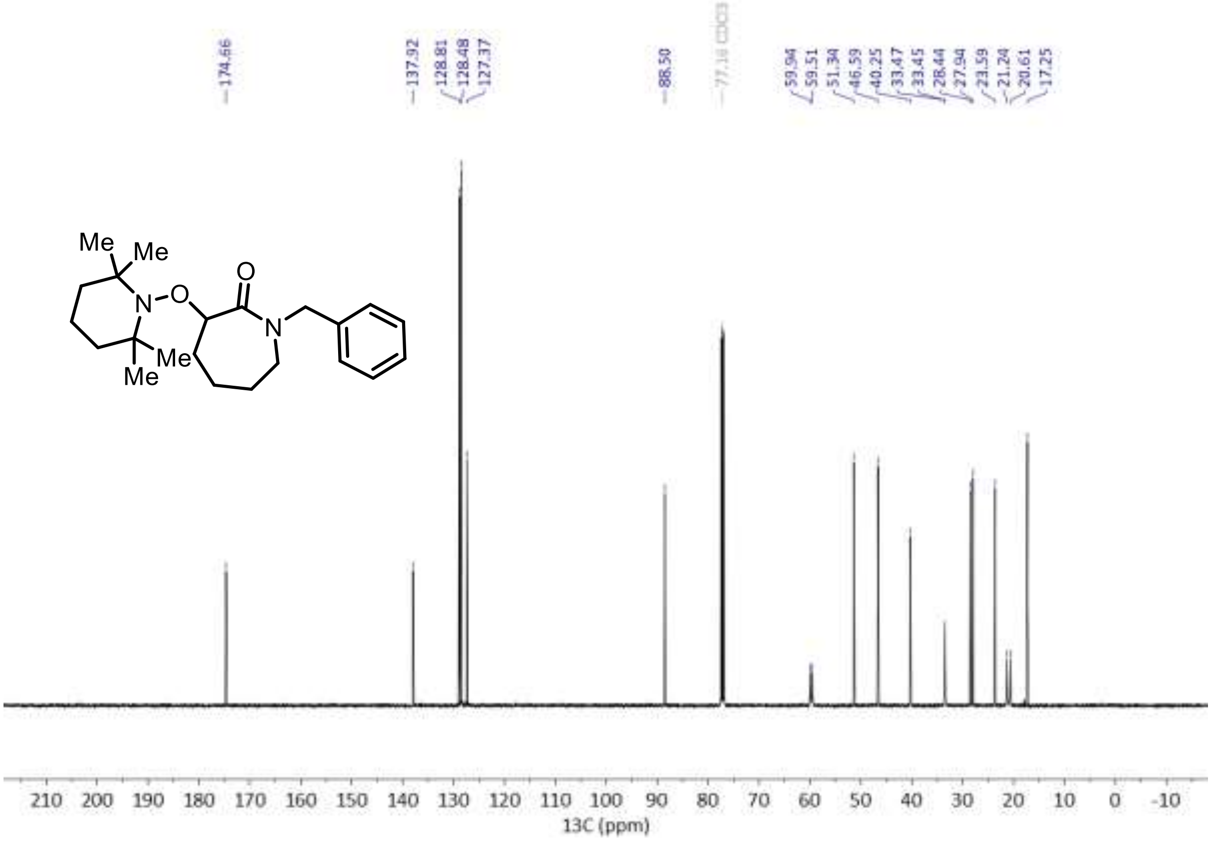

Compound 5d (¹H)

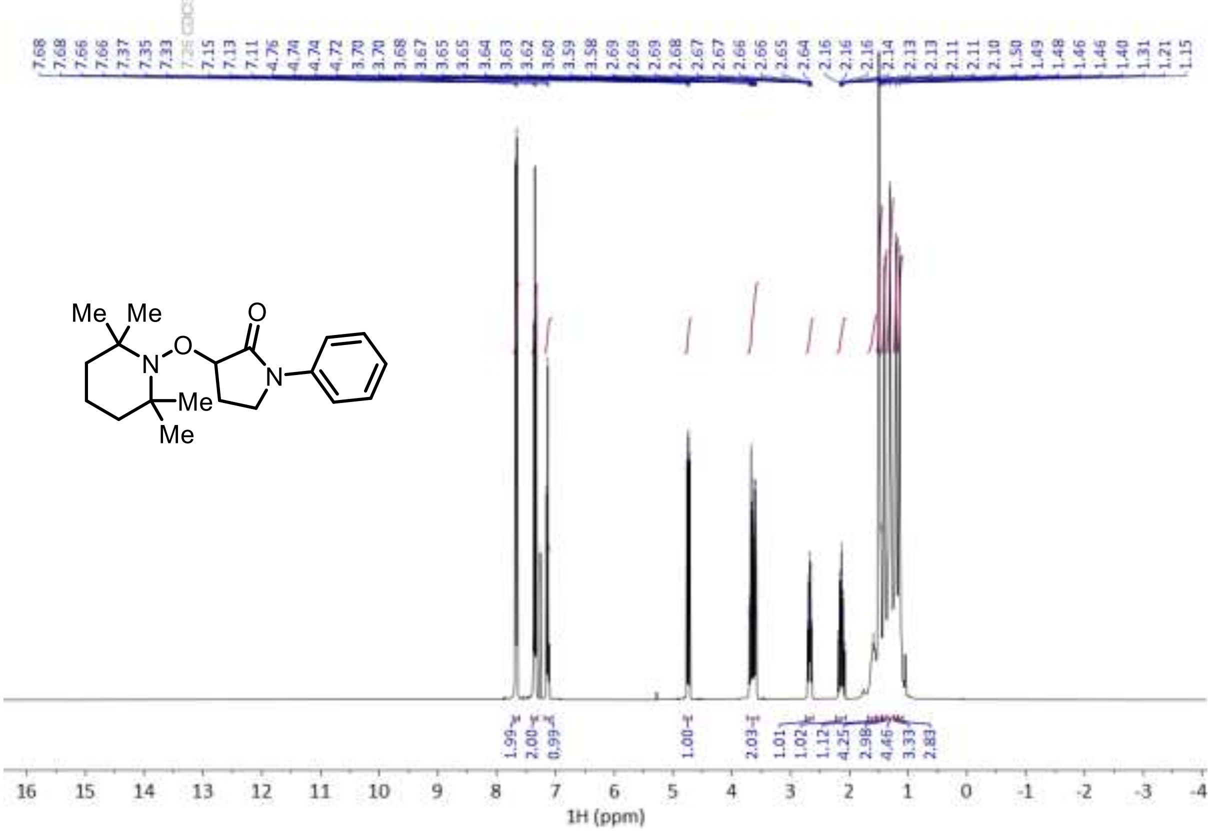

Compound 5d (¹³C)

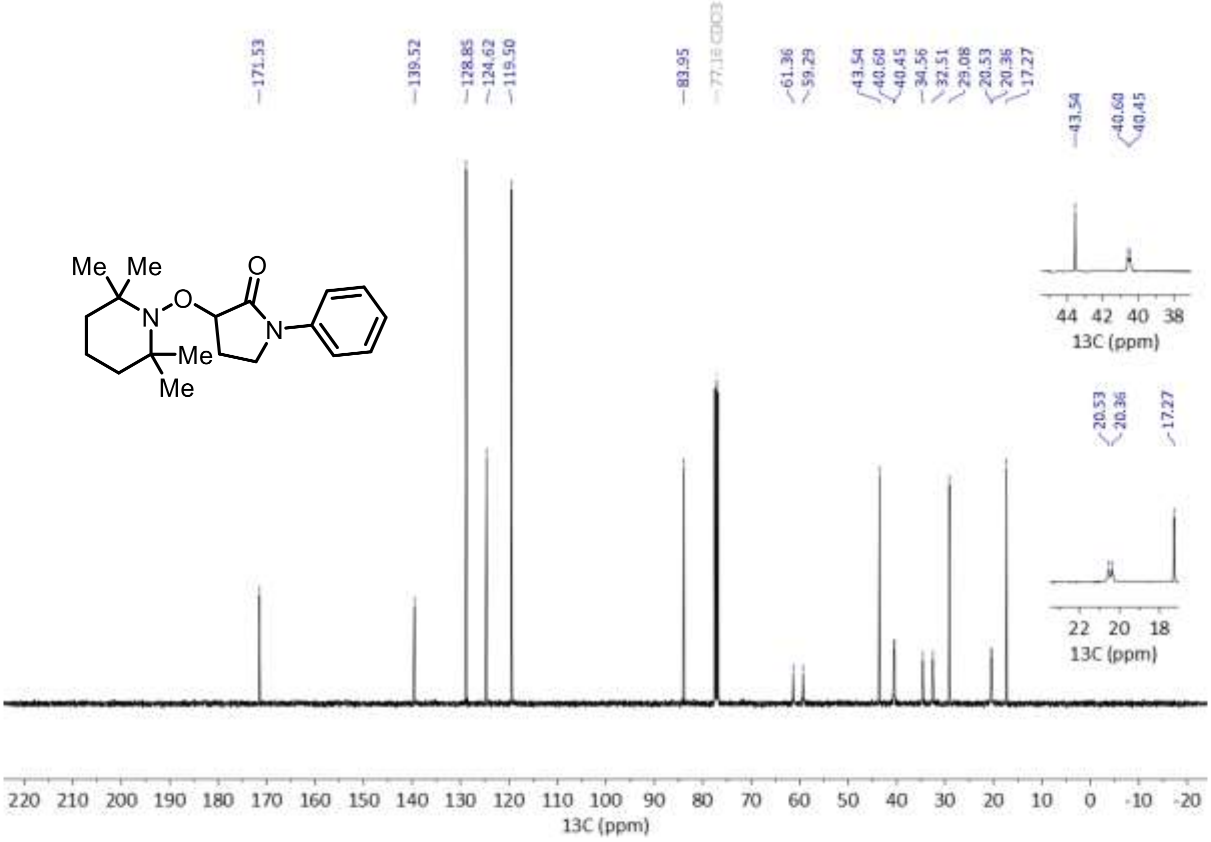

Compound S1-9 (^{1}H)

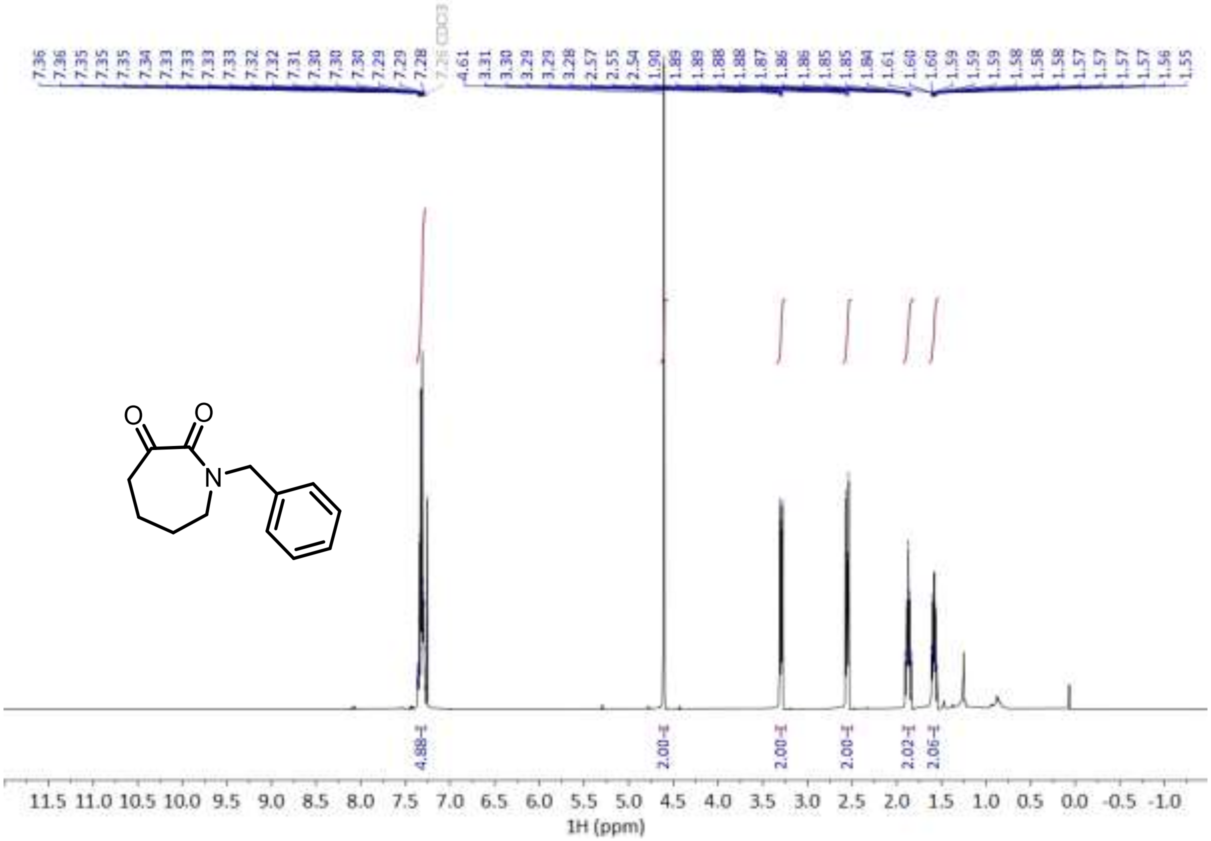

Compound S1-9 (^{13}C)

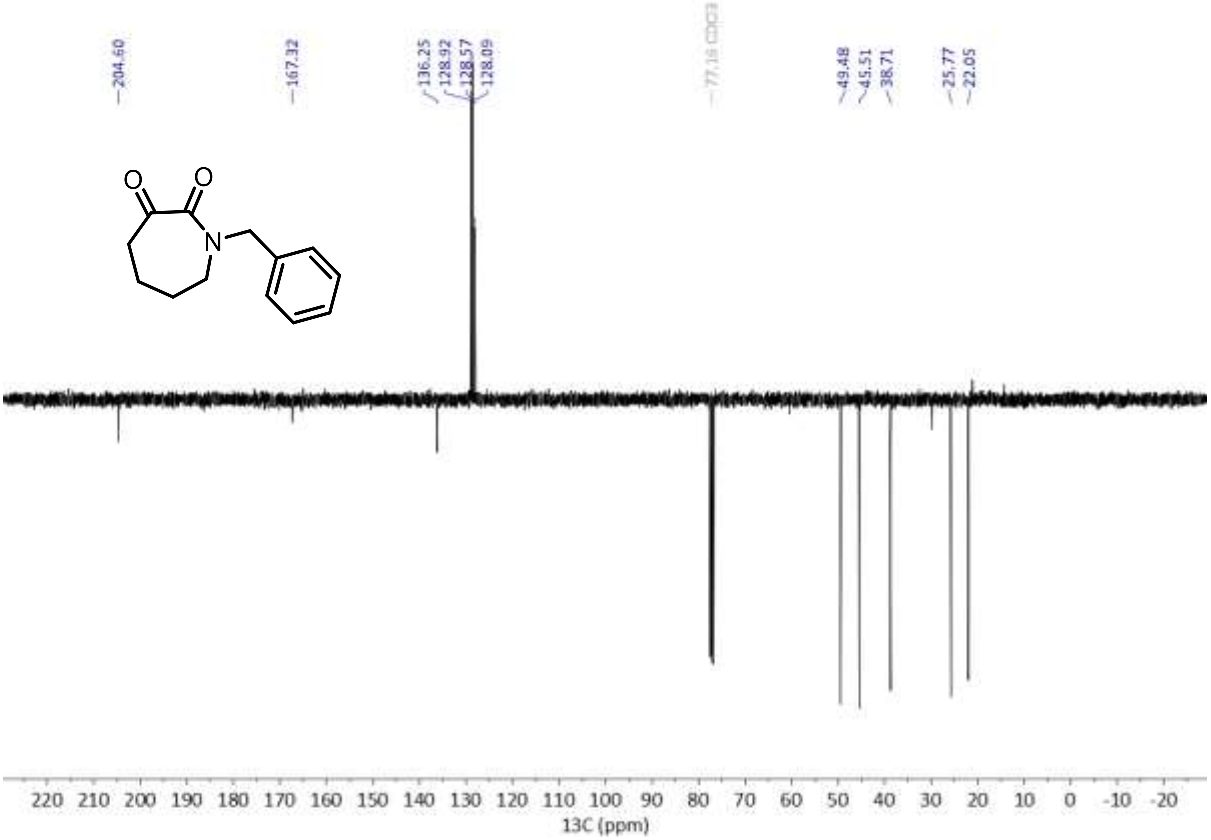

Compound 3a (¹H)

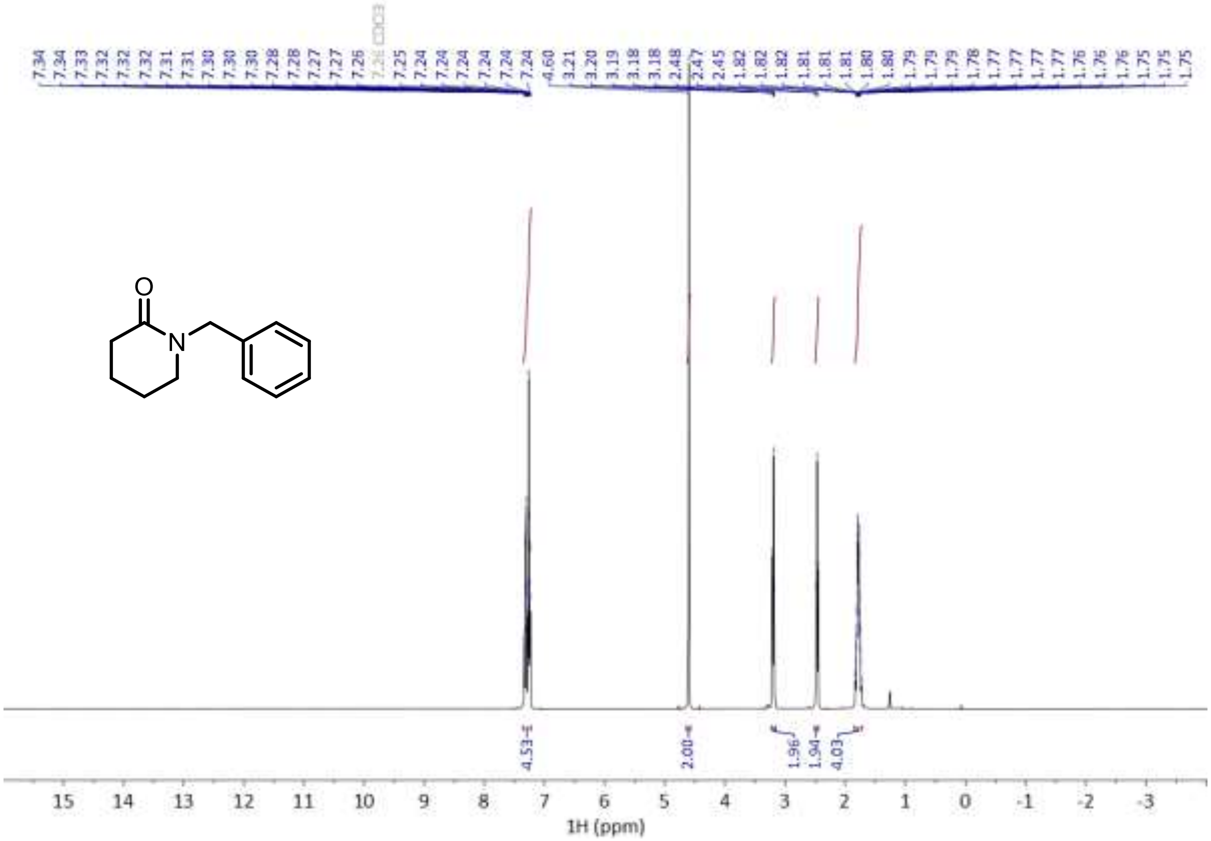

Compound 3a (¹³C)

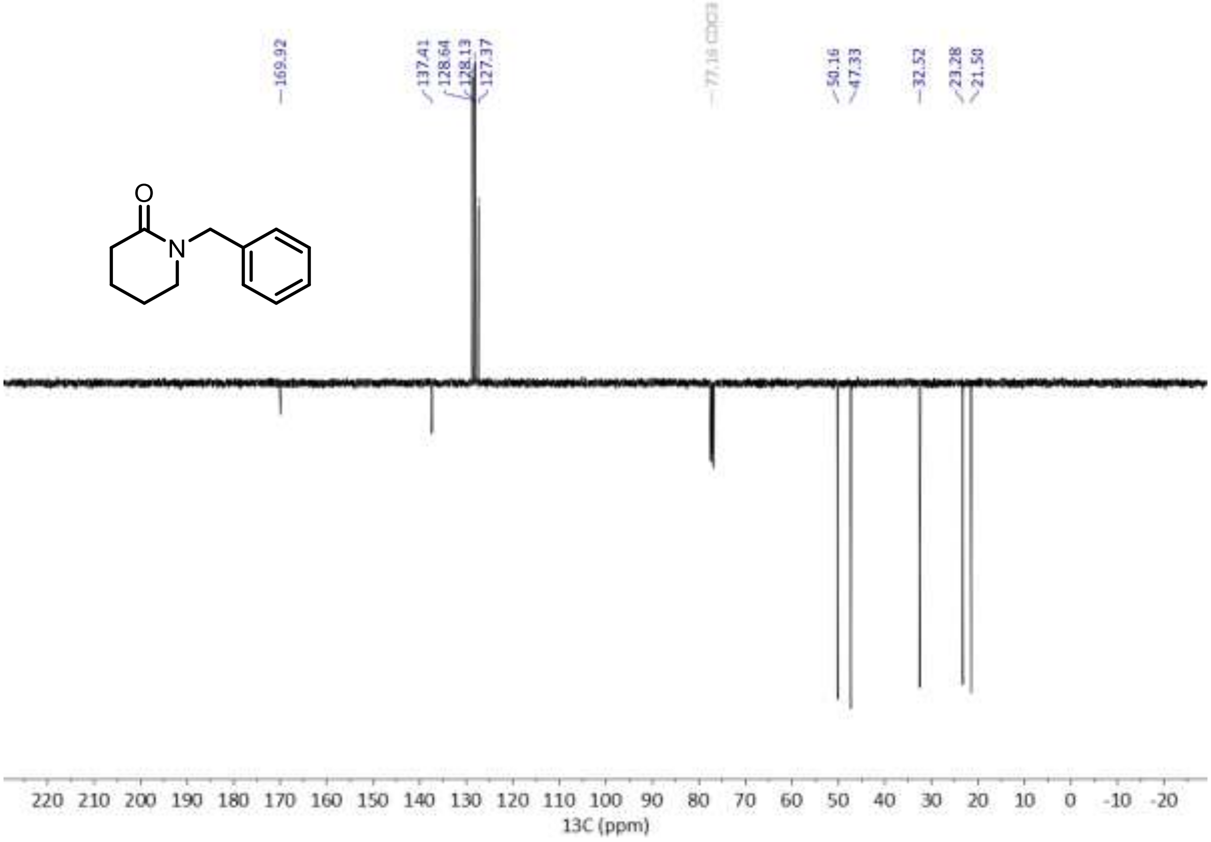

Compound 3b (¹H)

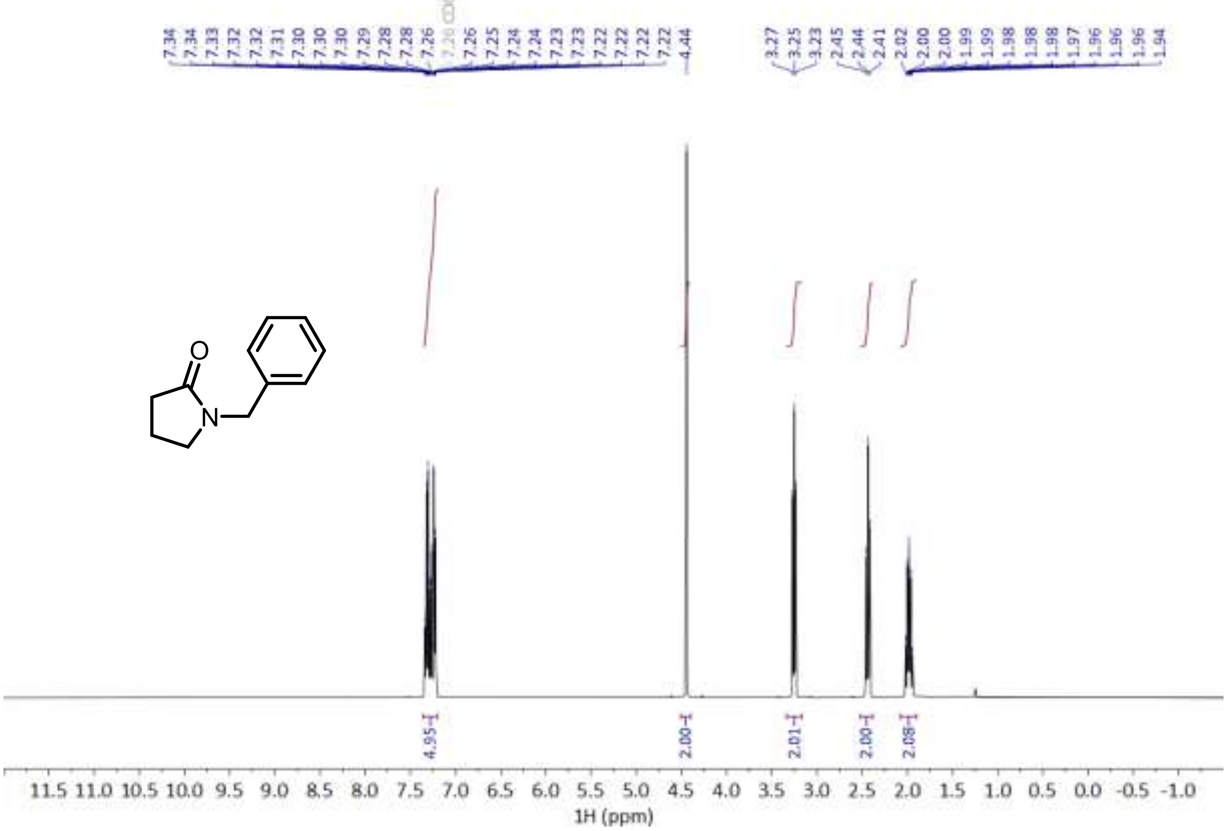

Compound 3b (¹³C)

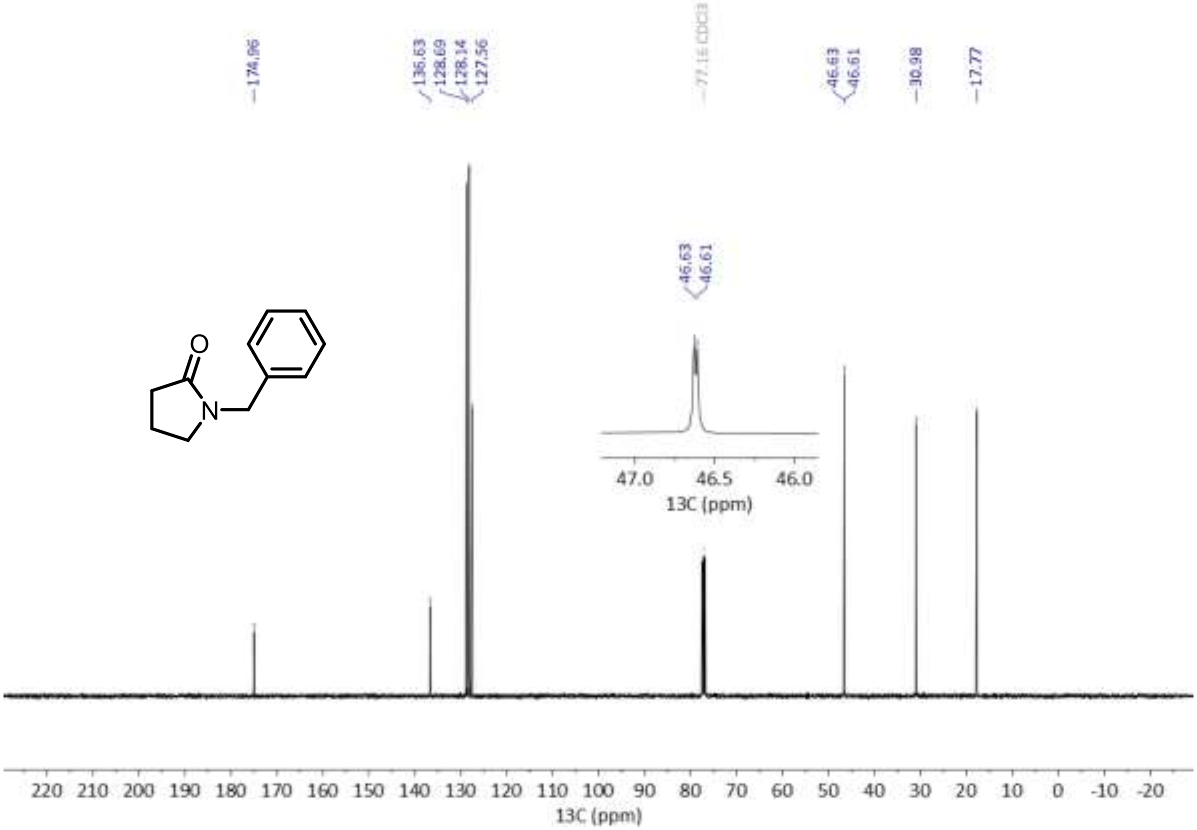

Compound 3c (^{1}H)

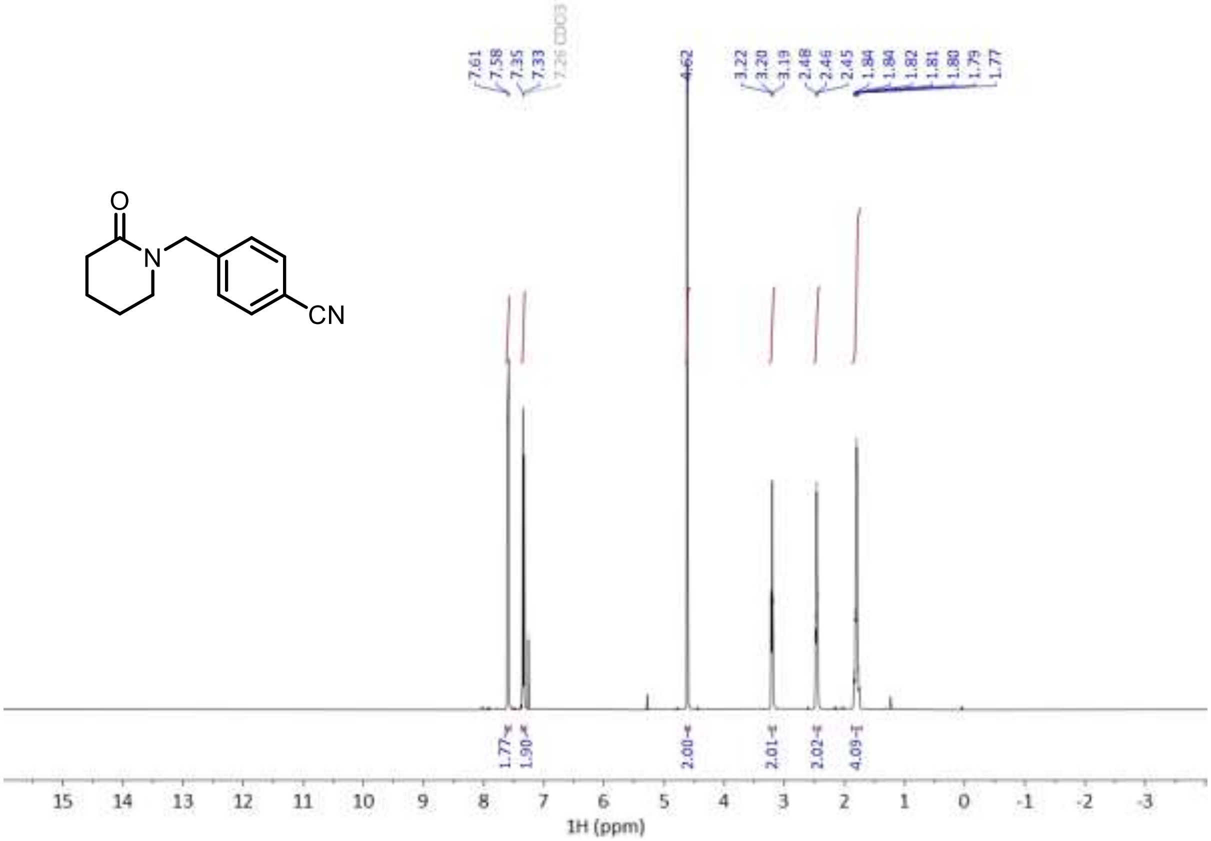

Compound 3c (^{13}C)

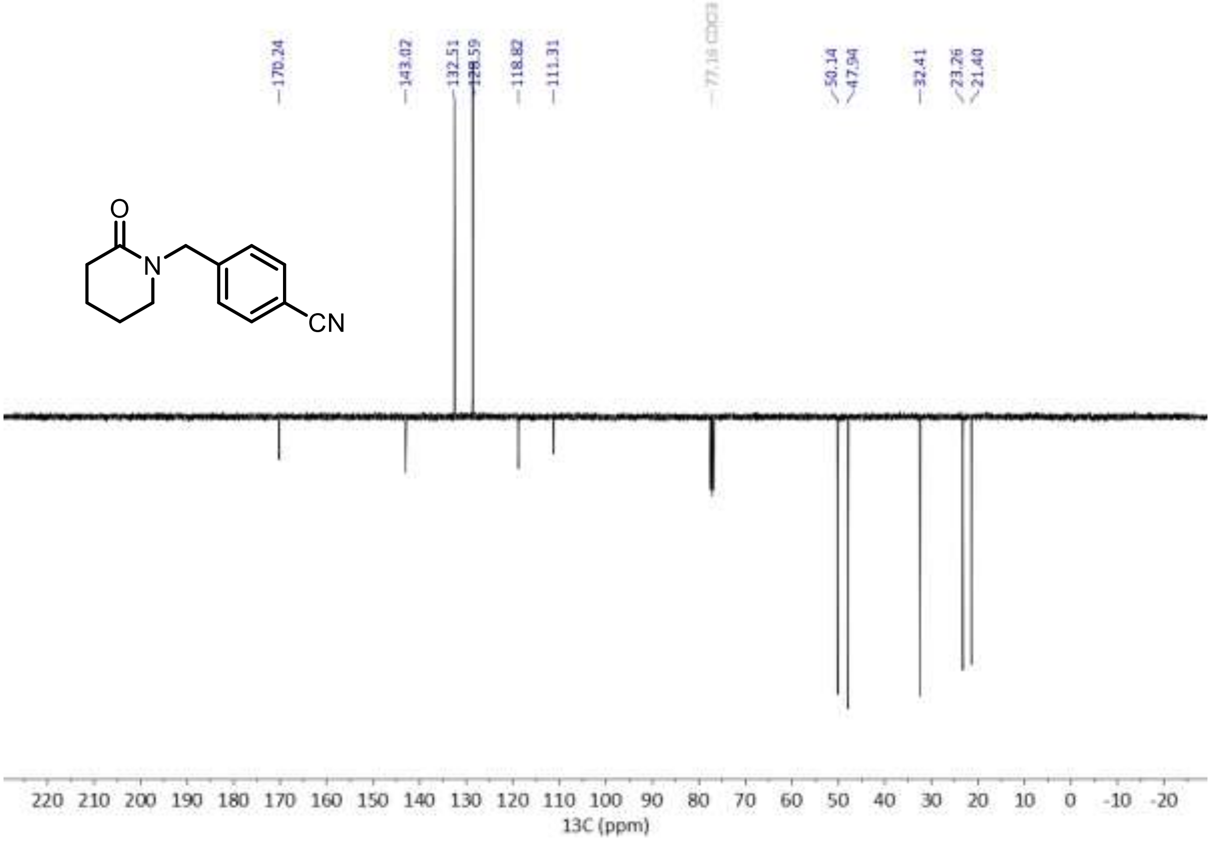

Compound 3d (^{1}H)

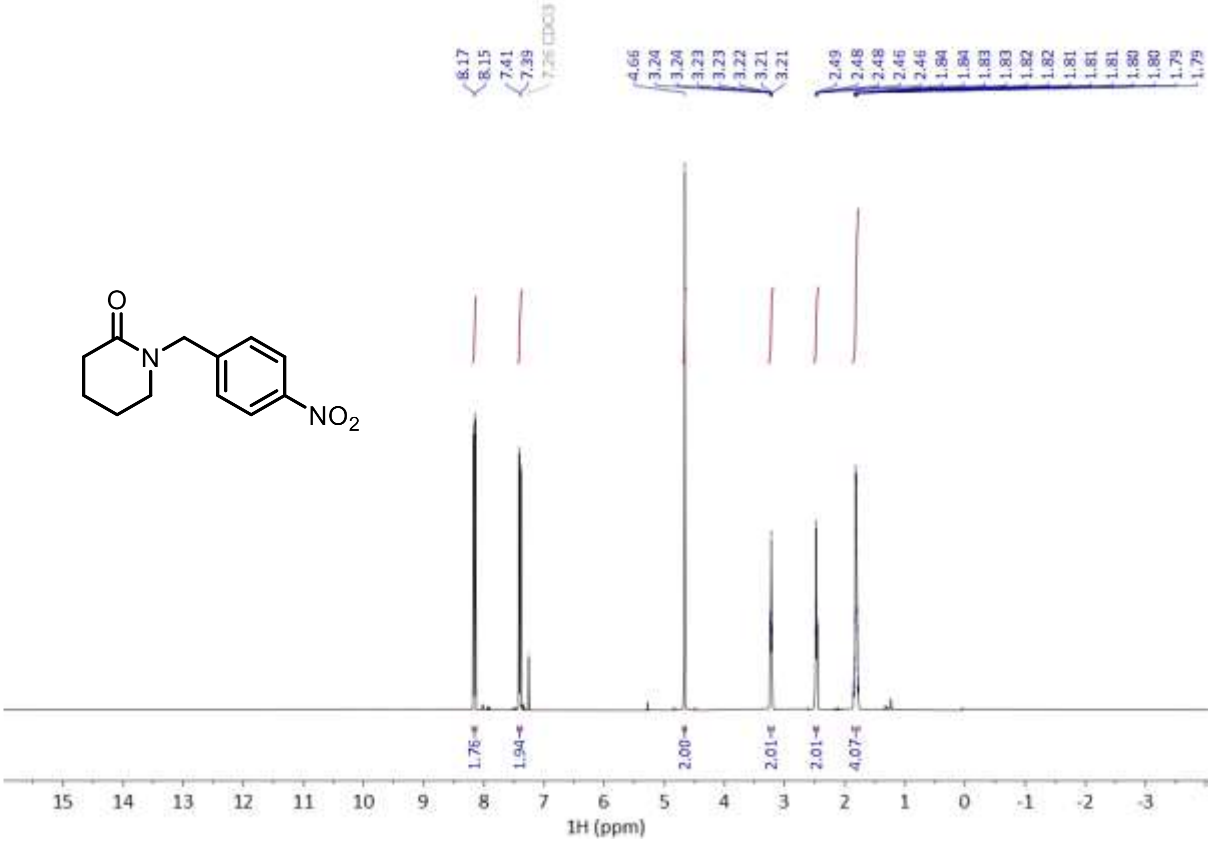

Compound 3d (^{13}C)

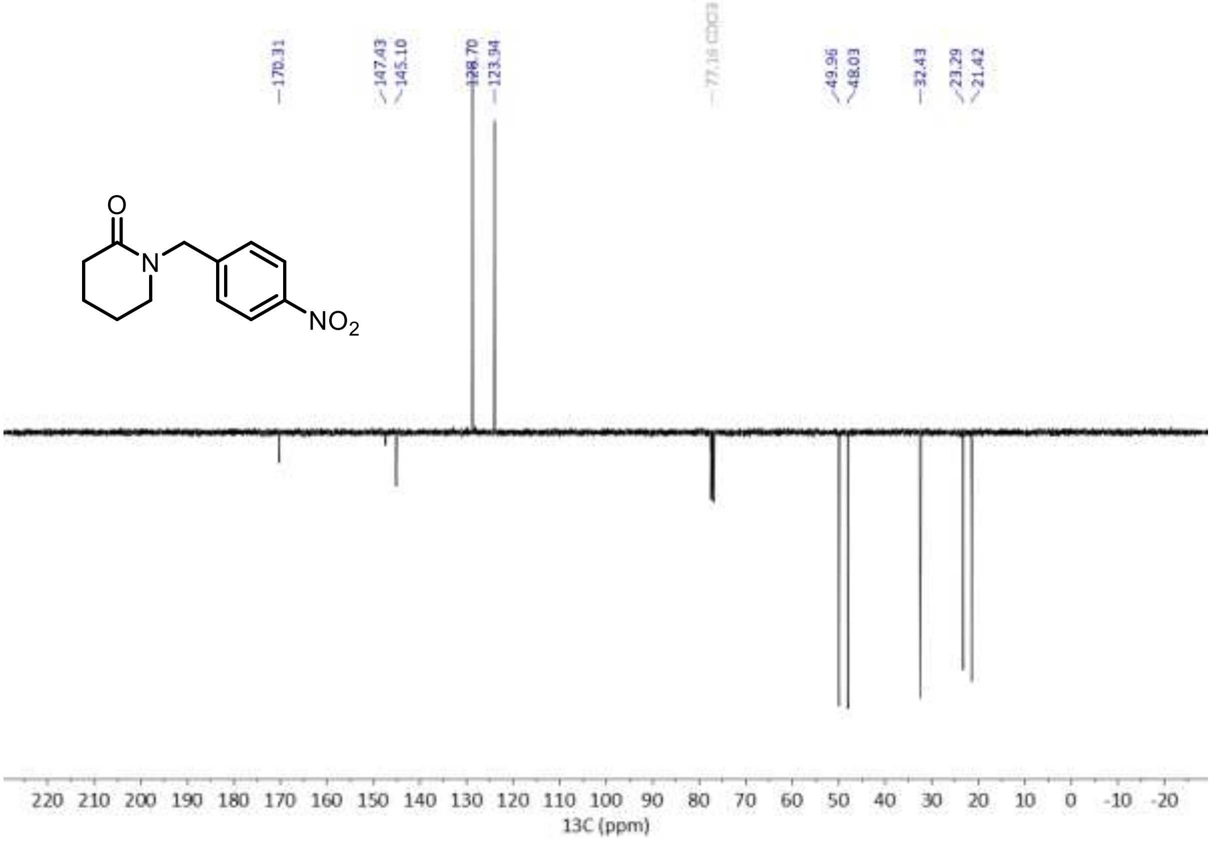

Compound 3e (^{1}H)

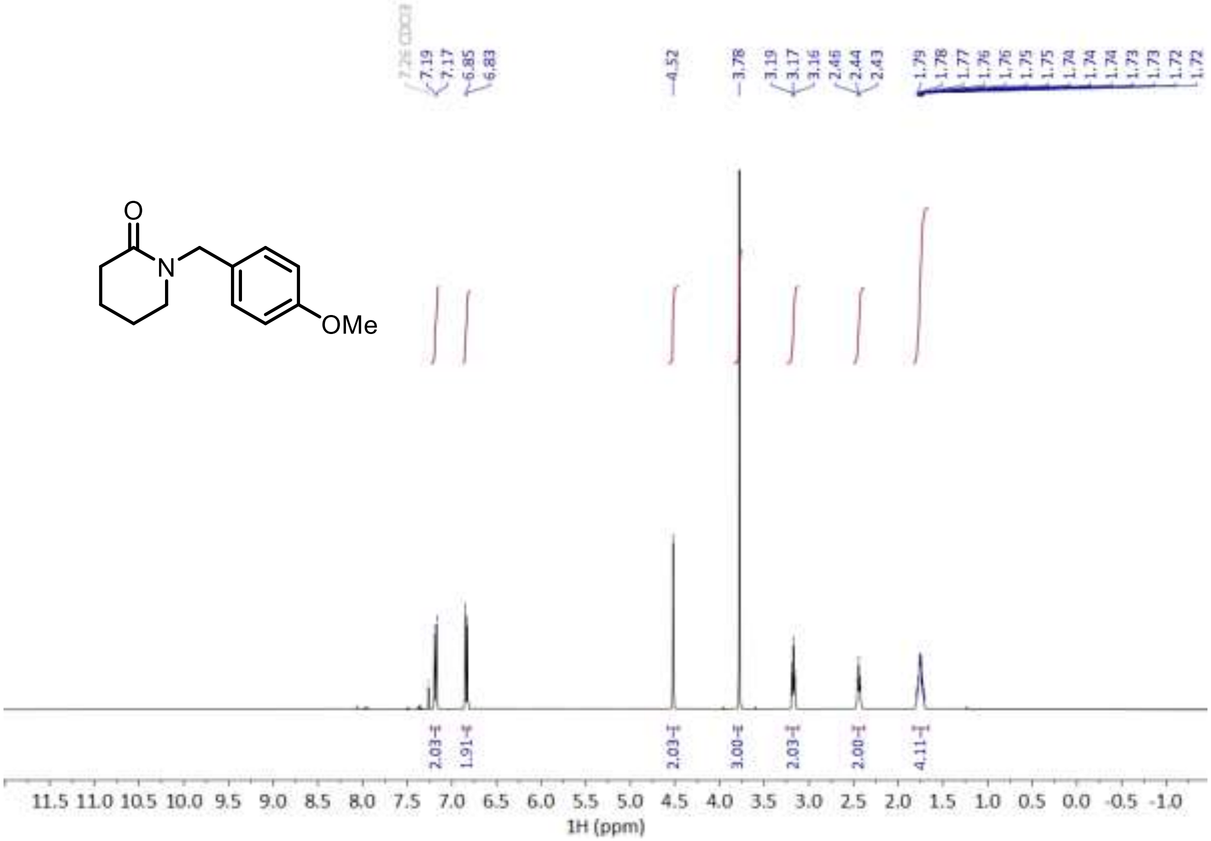

Compound 3e (^{13}C)

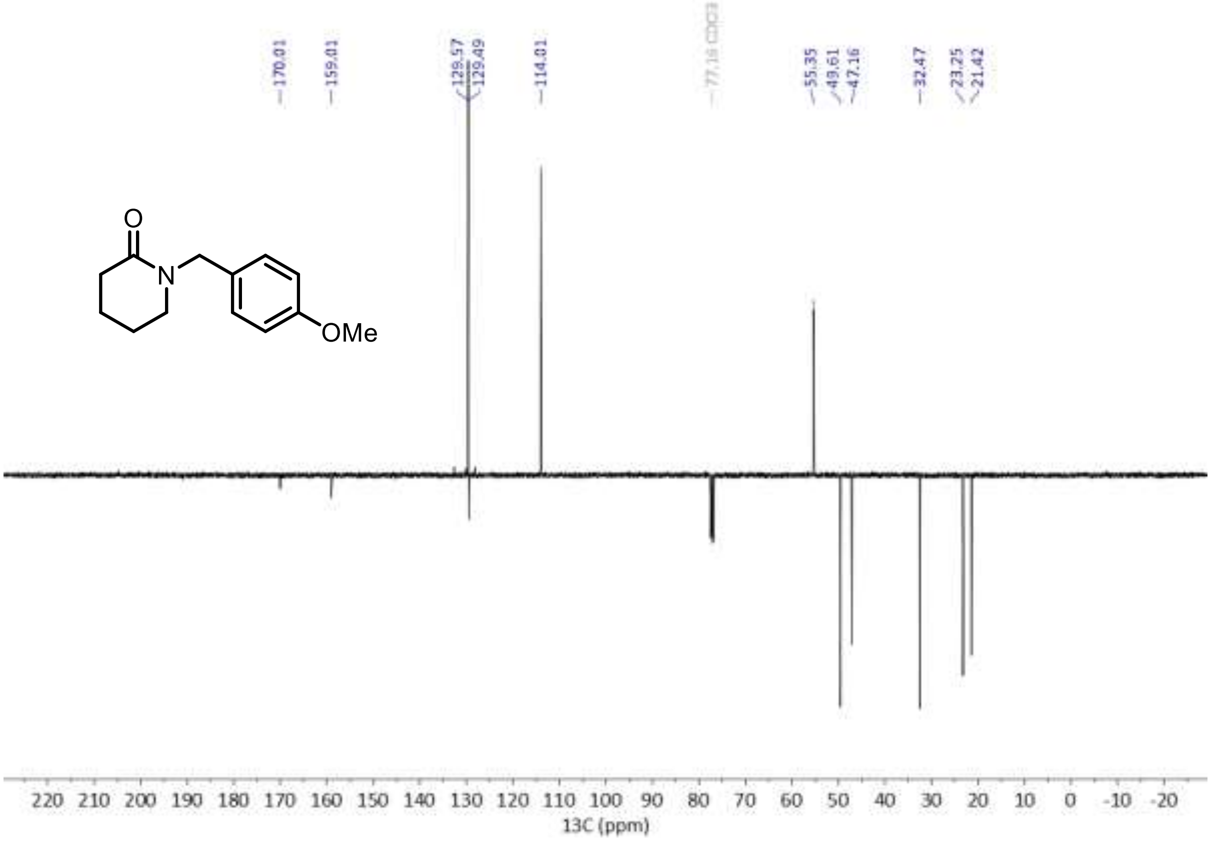

Compound 3f (^{1}H)

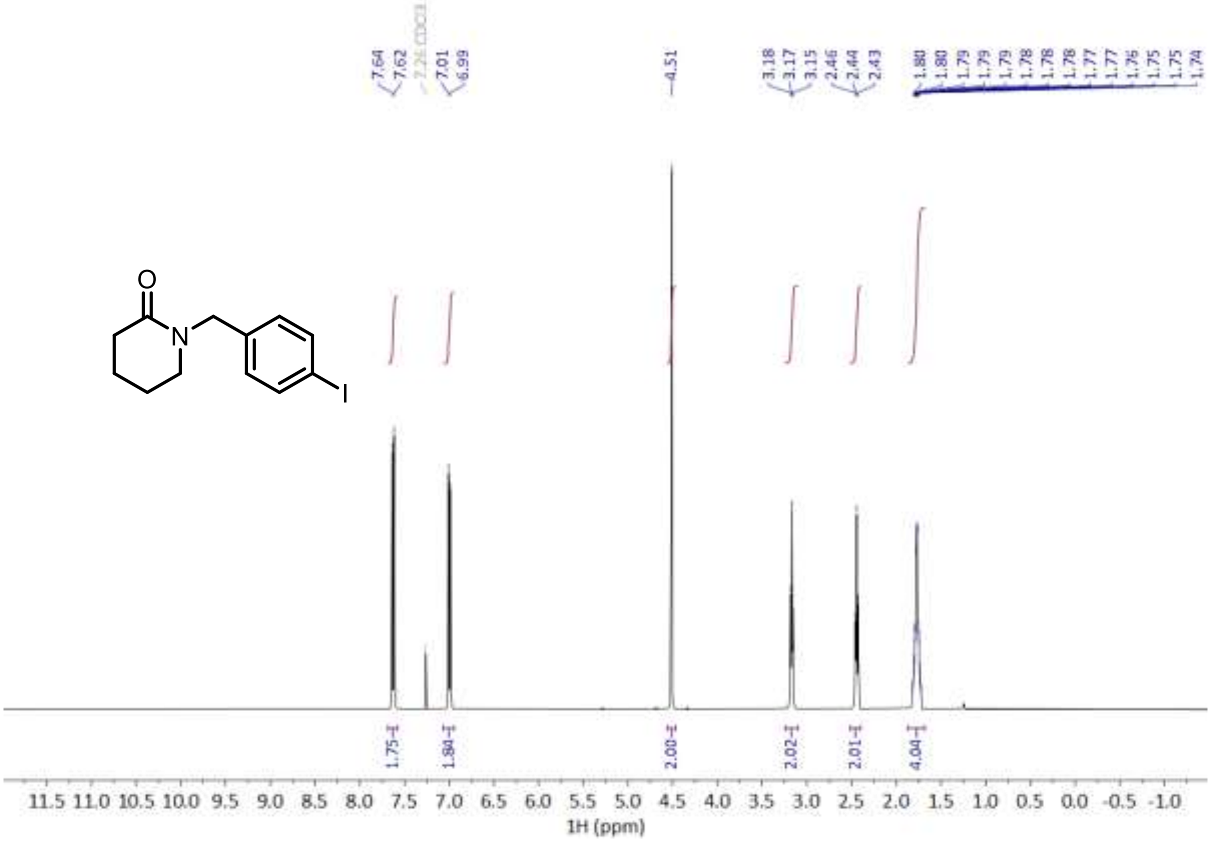

Compound 3f (^{13}C)

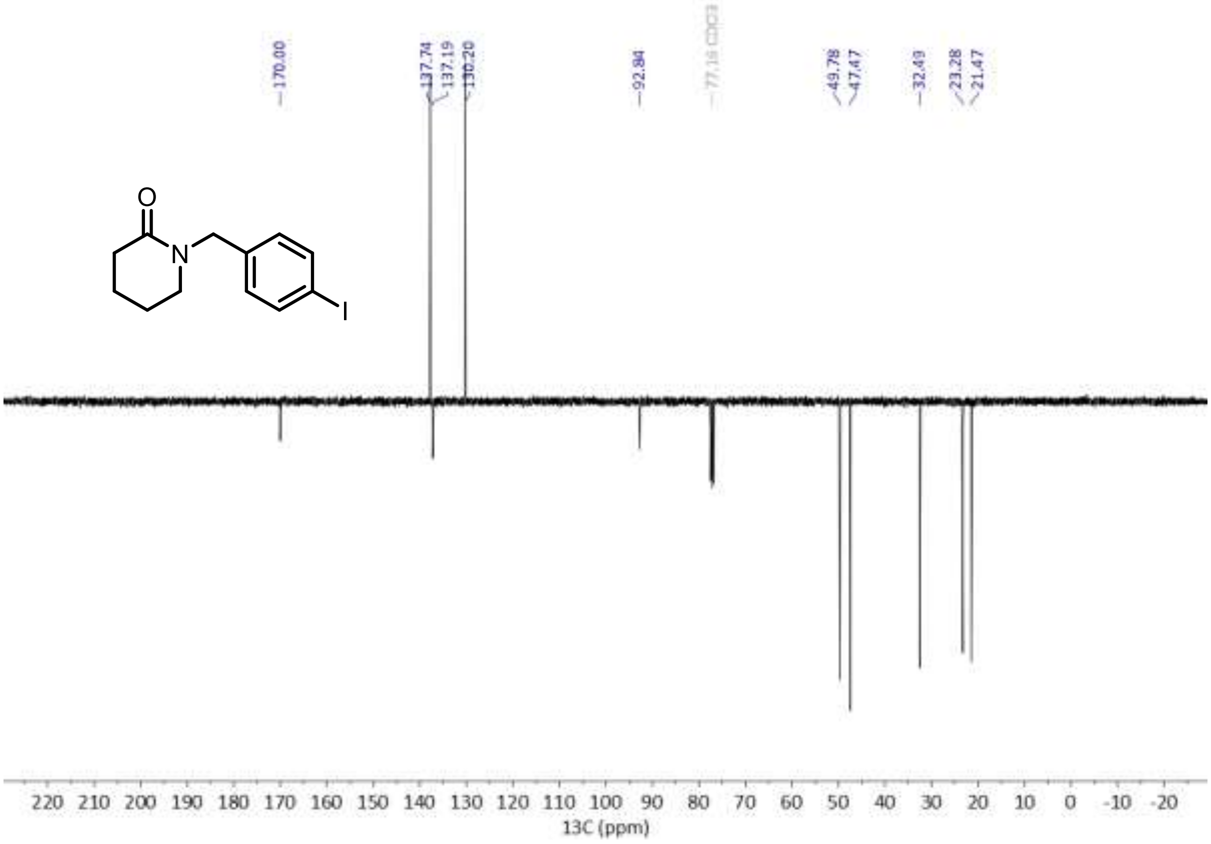

Compound 3g (^{1}H)

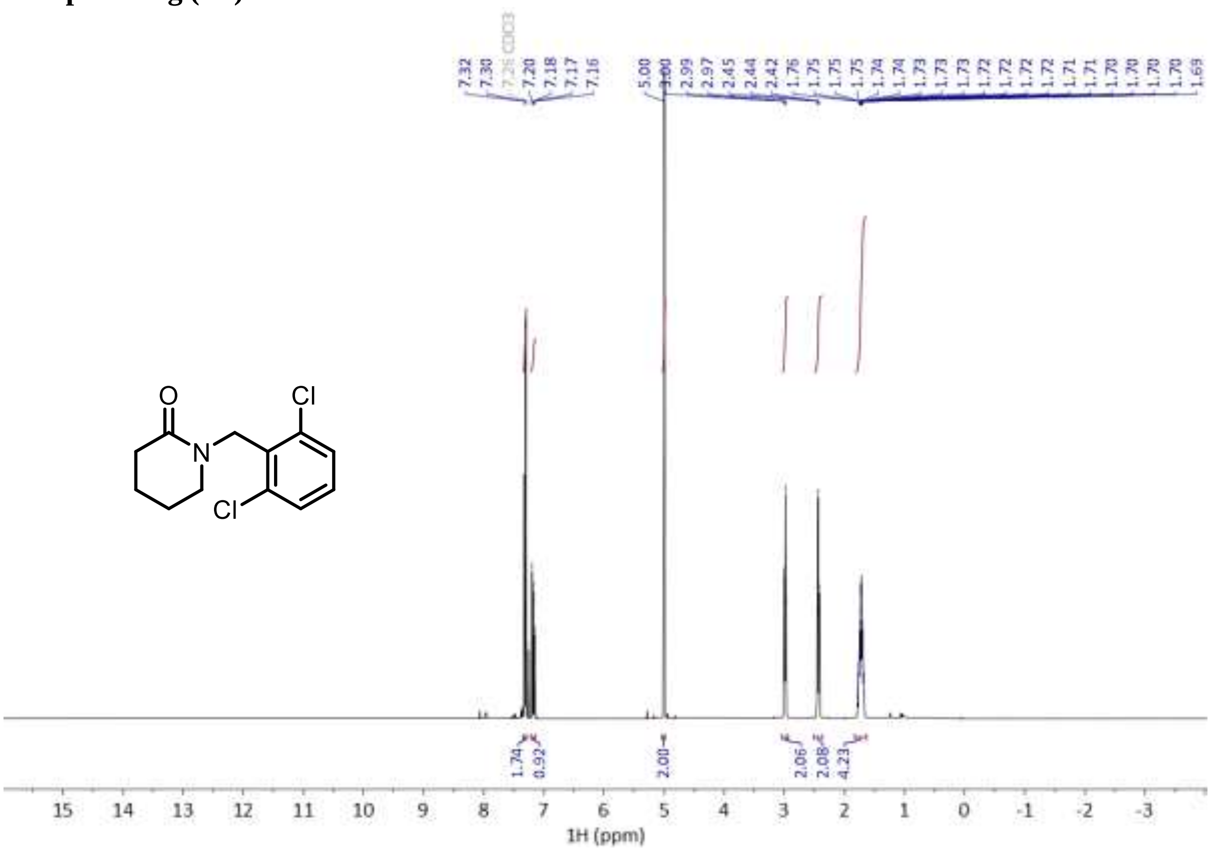

Compound 3g (^{13}C)

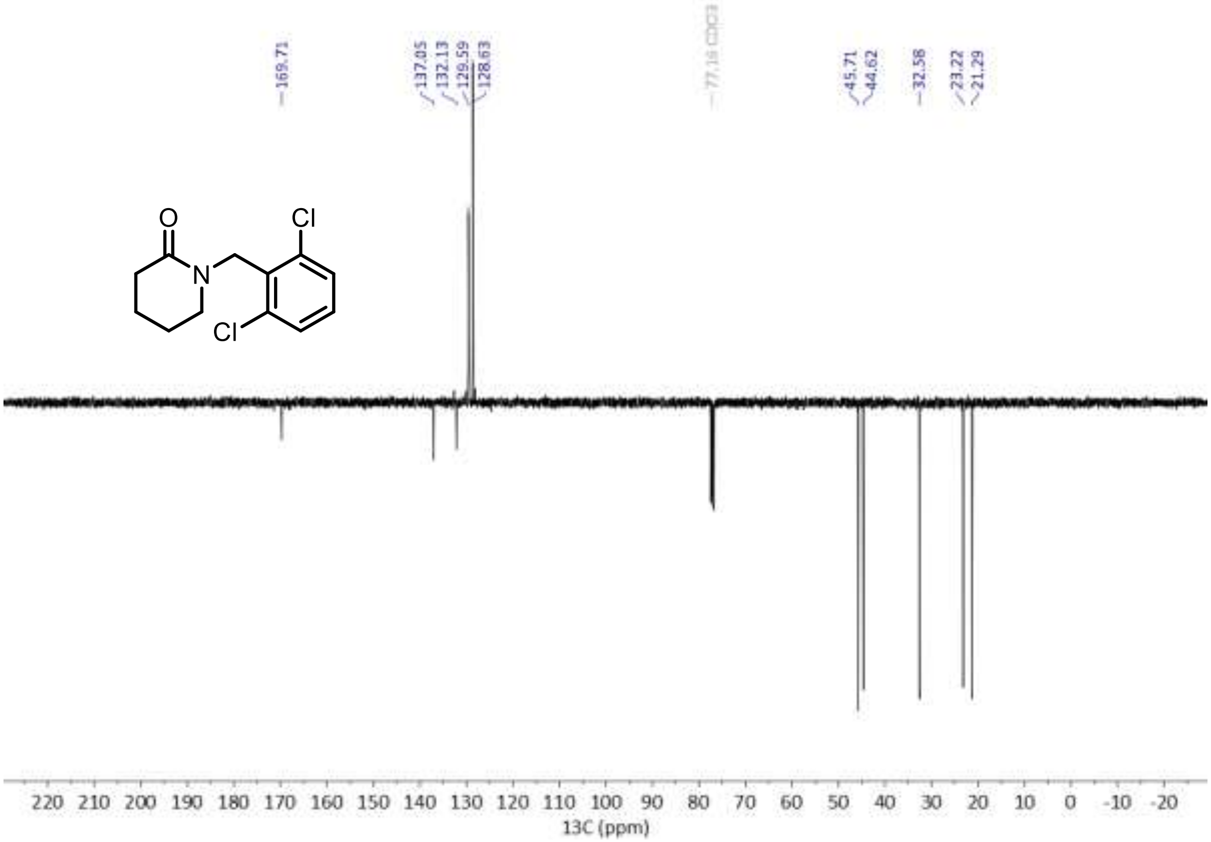

Compound 3h (^{1}H)

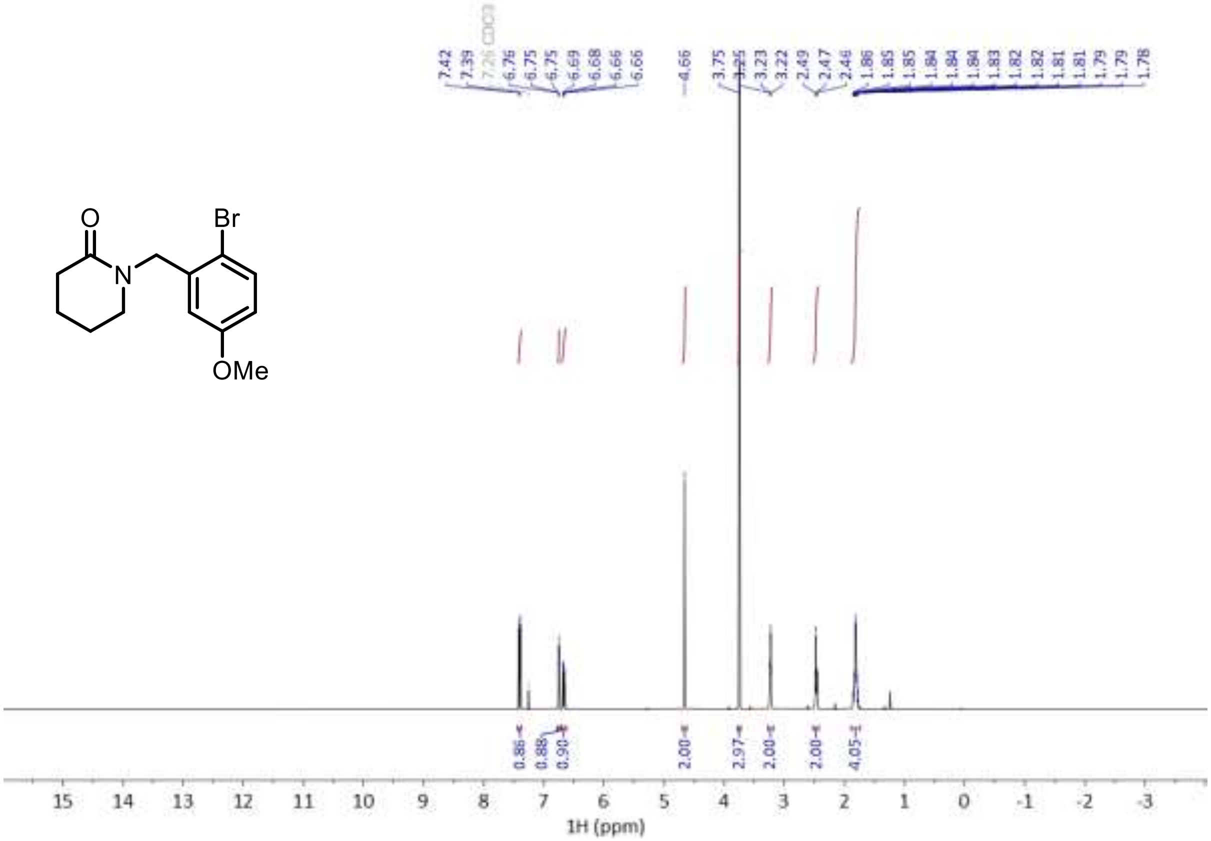

Compound 3h (^{13}C)

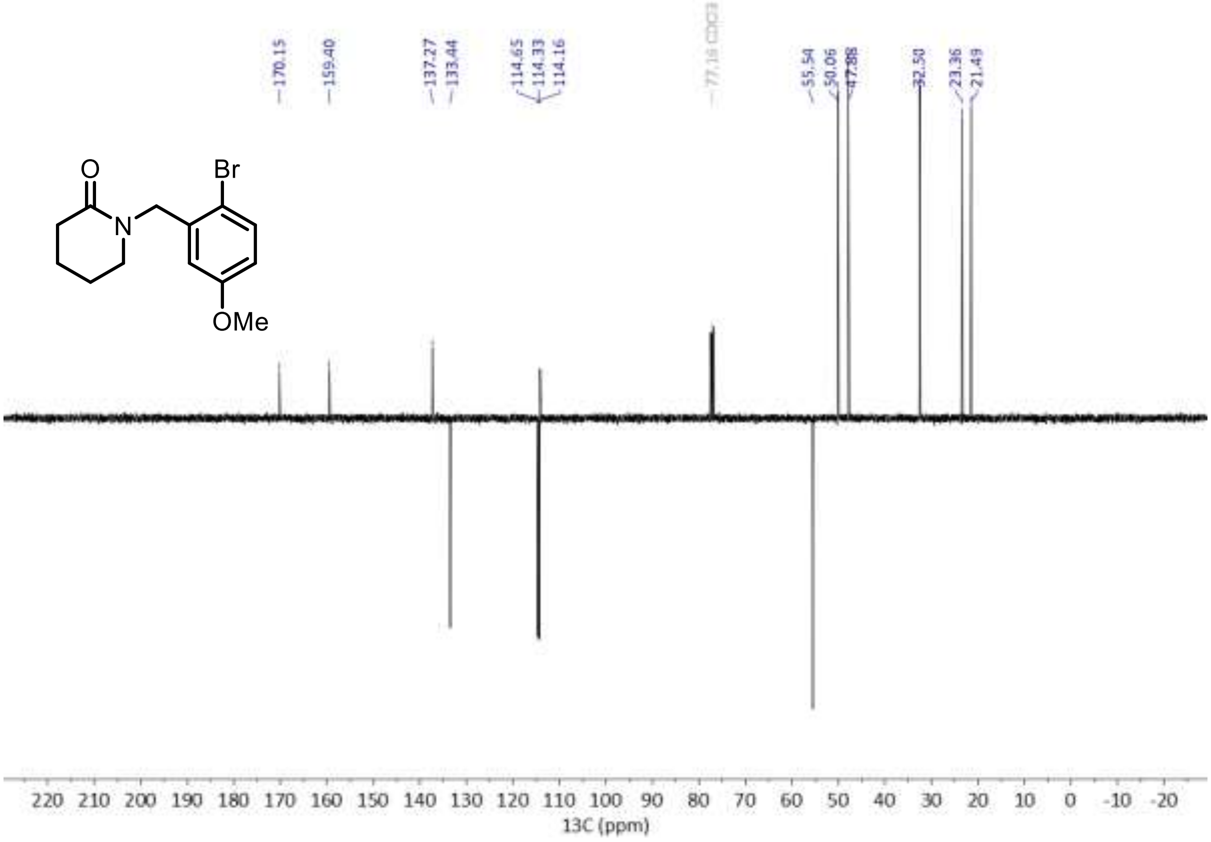

Compound 3i (^{1}H)

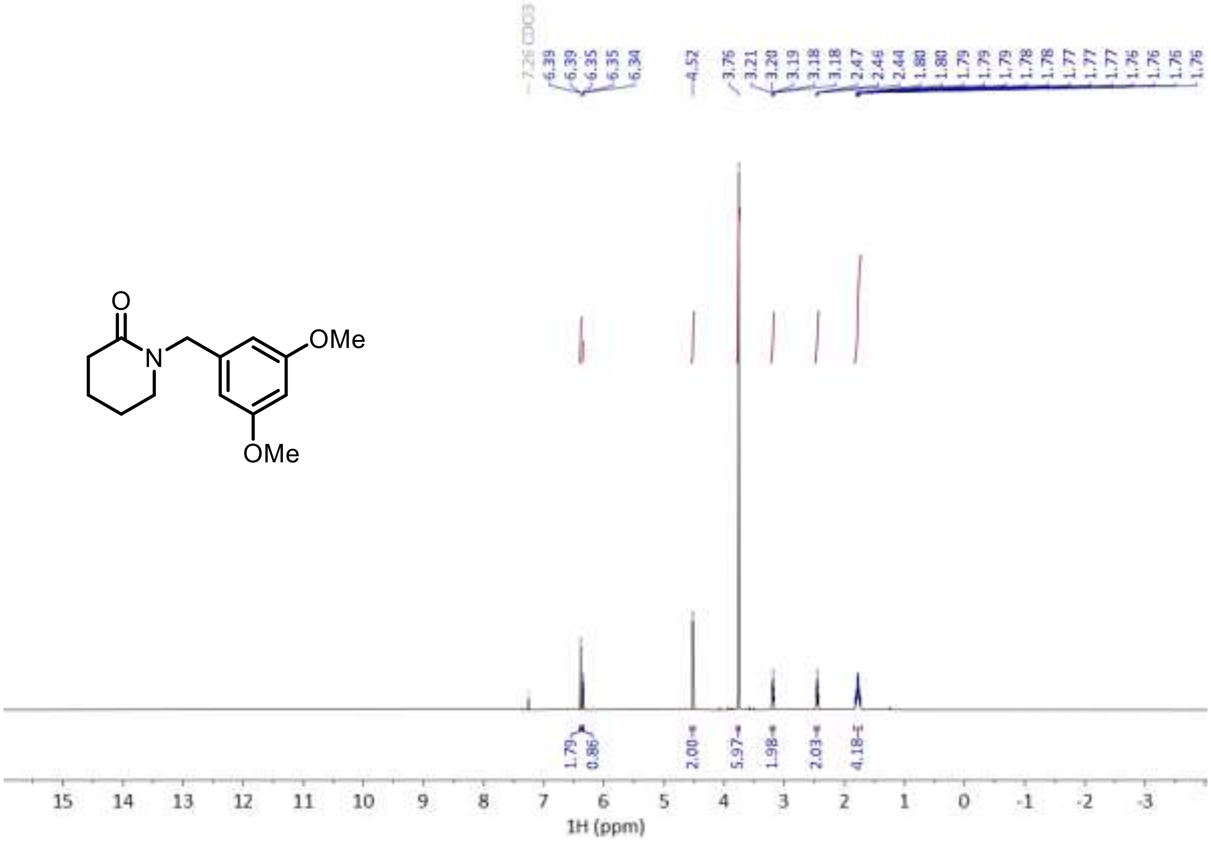

Compound 3i (^{13}C)

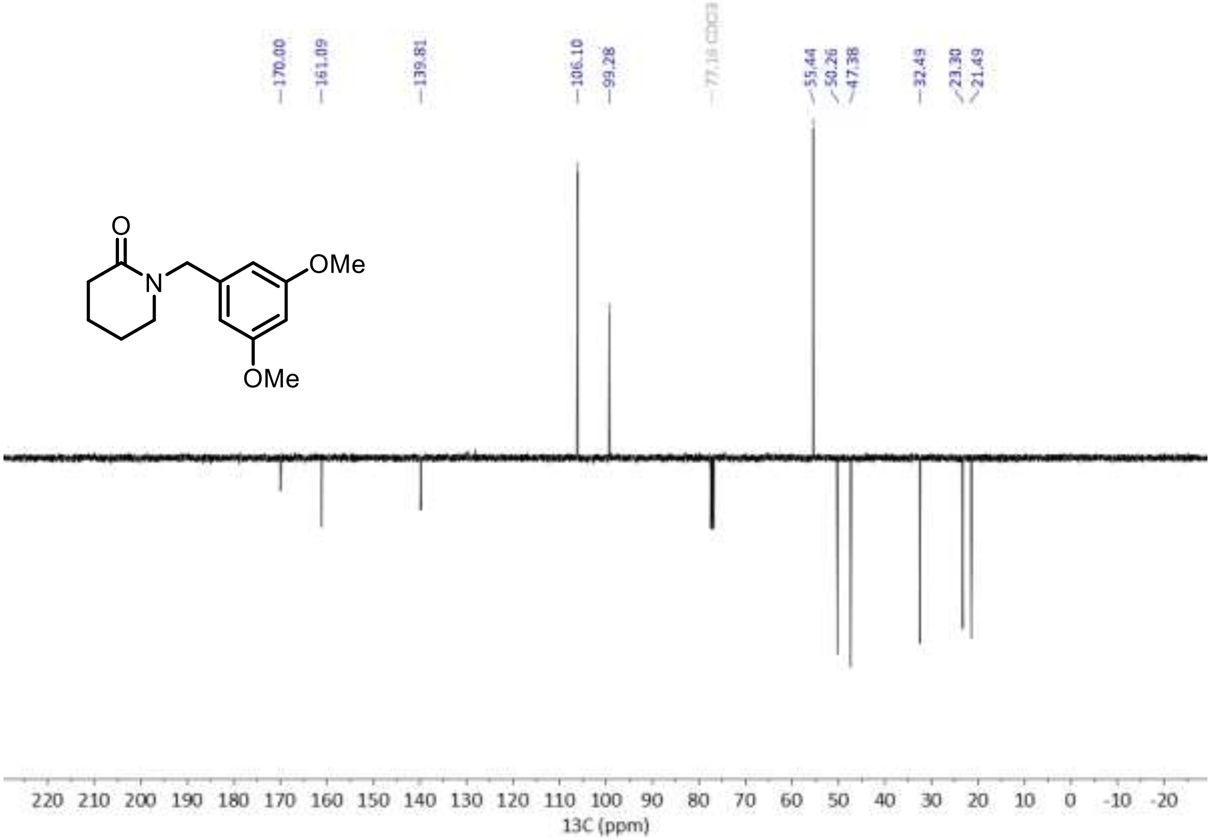

Compound 3j (^{1}H)

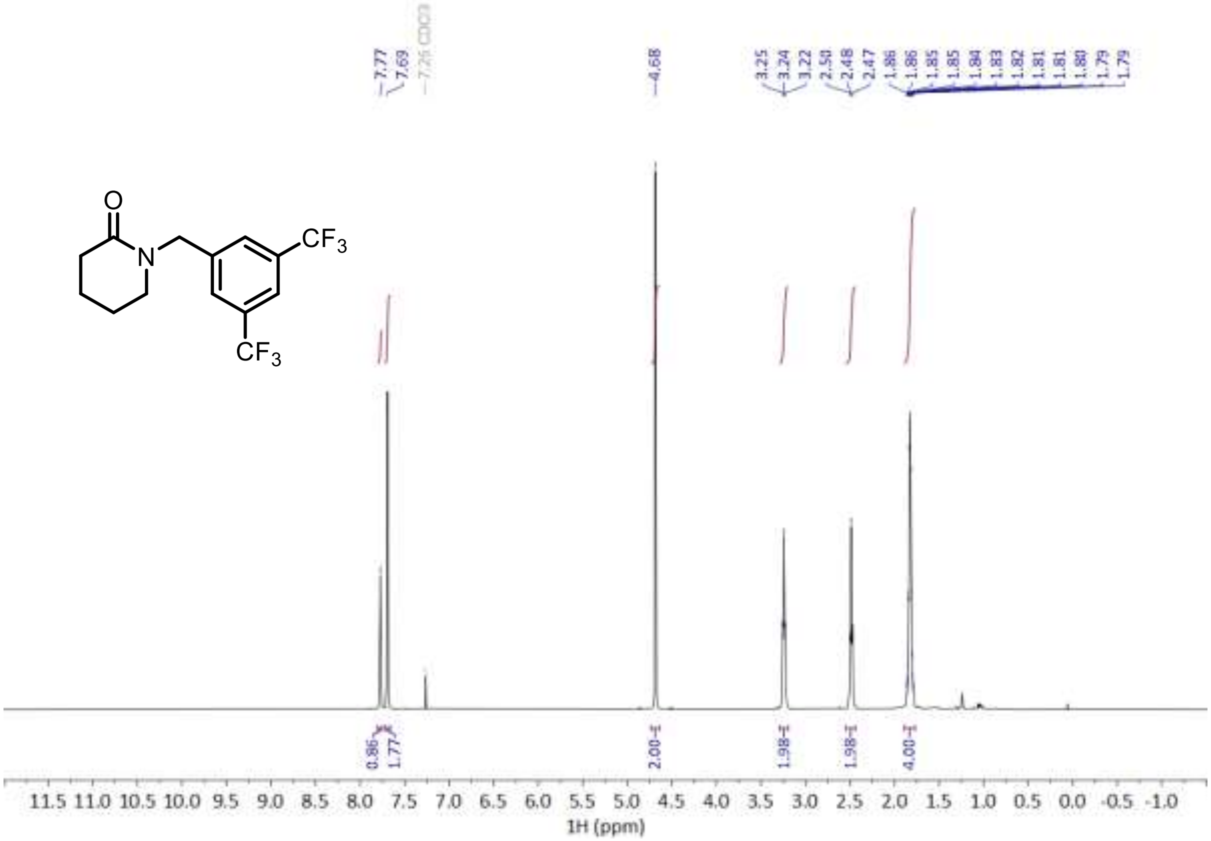

Compound 3j (^{13}C)

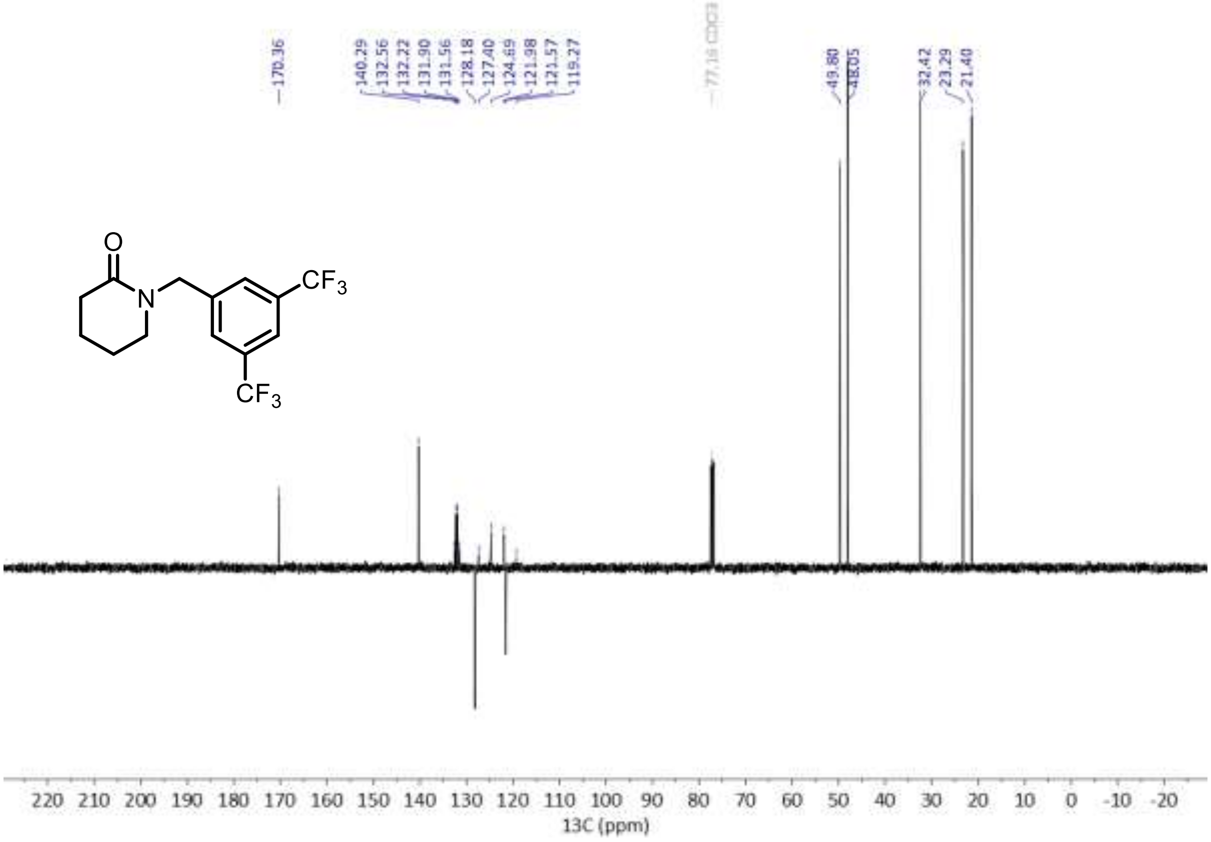

Compound 3j (^{19}F)

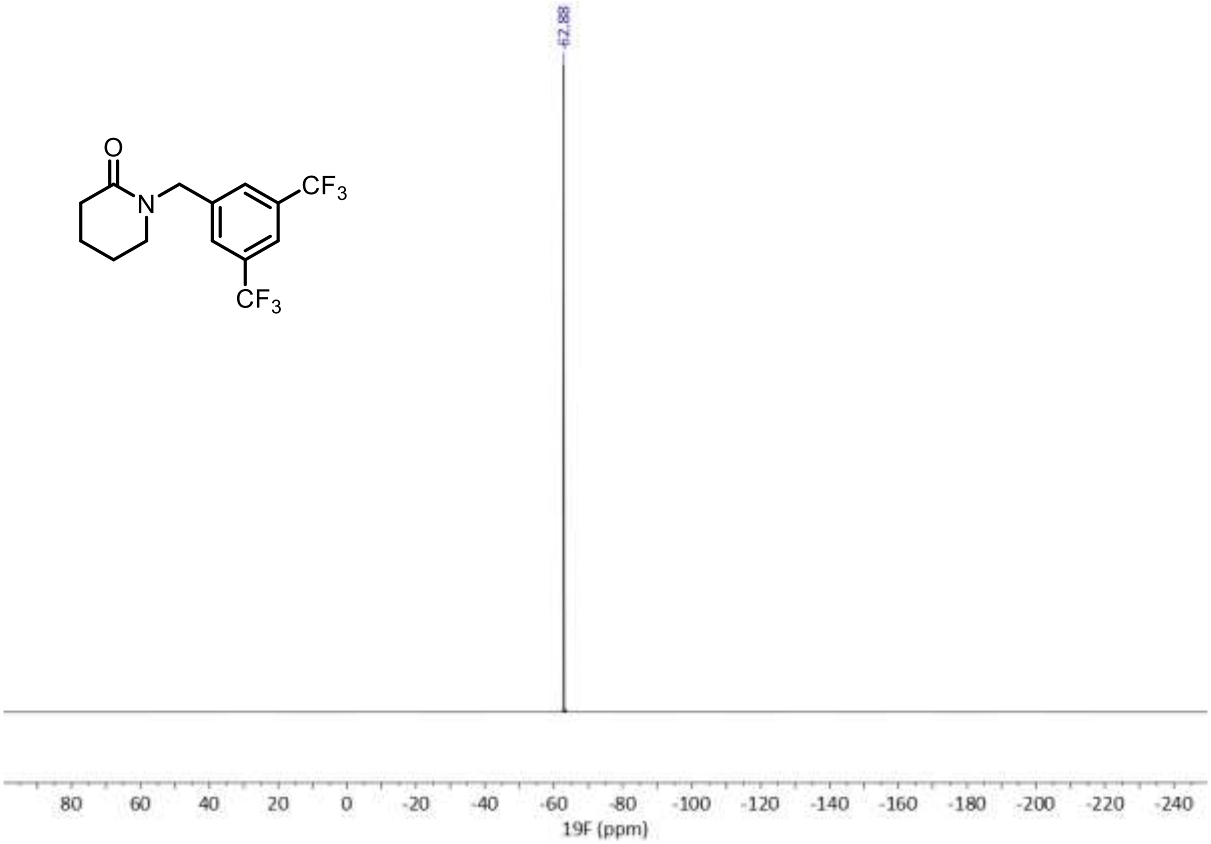

Compound 3k (¹H)

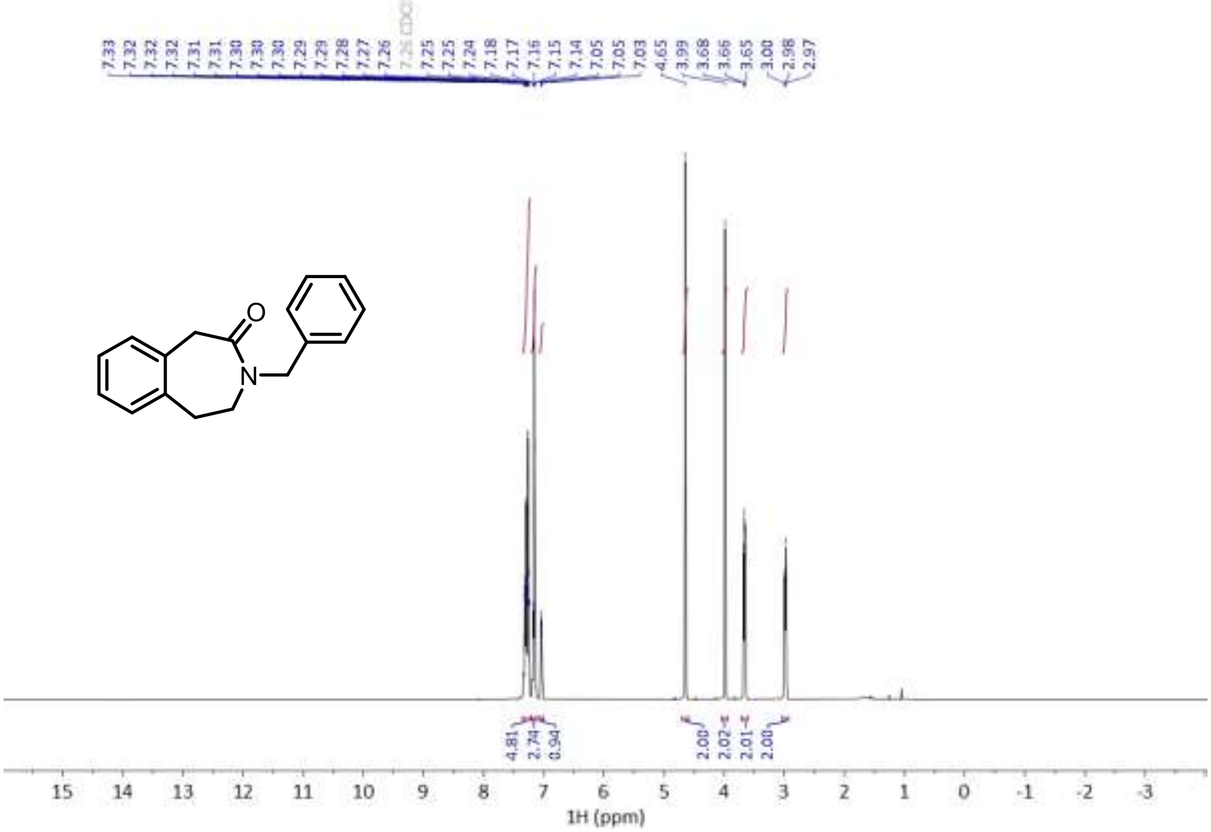

Compound 3k (¹³C)

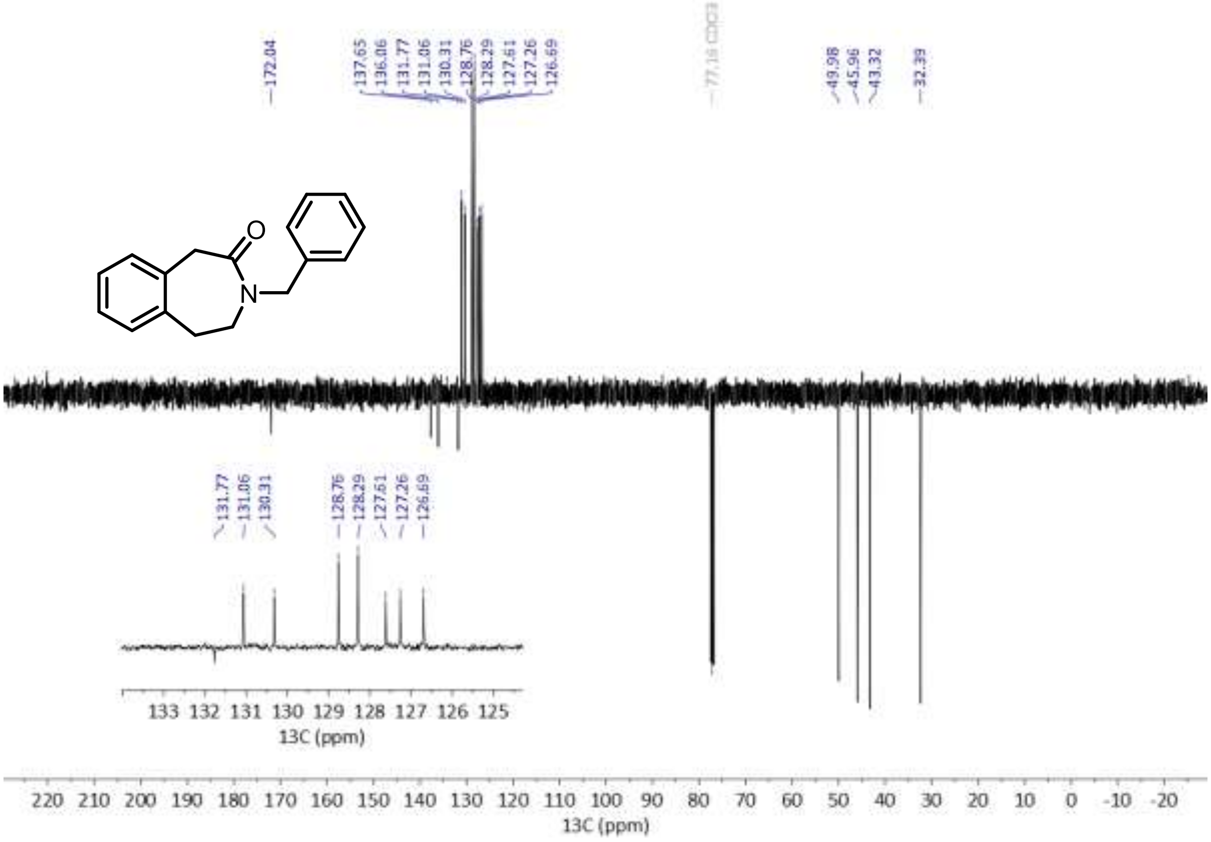

Compound 3l (¹H)

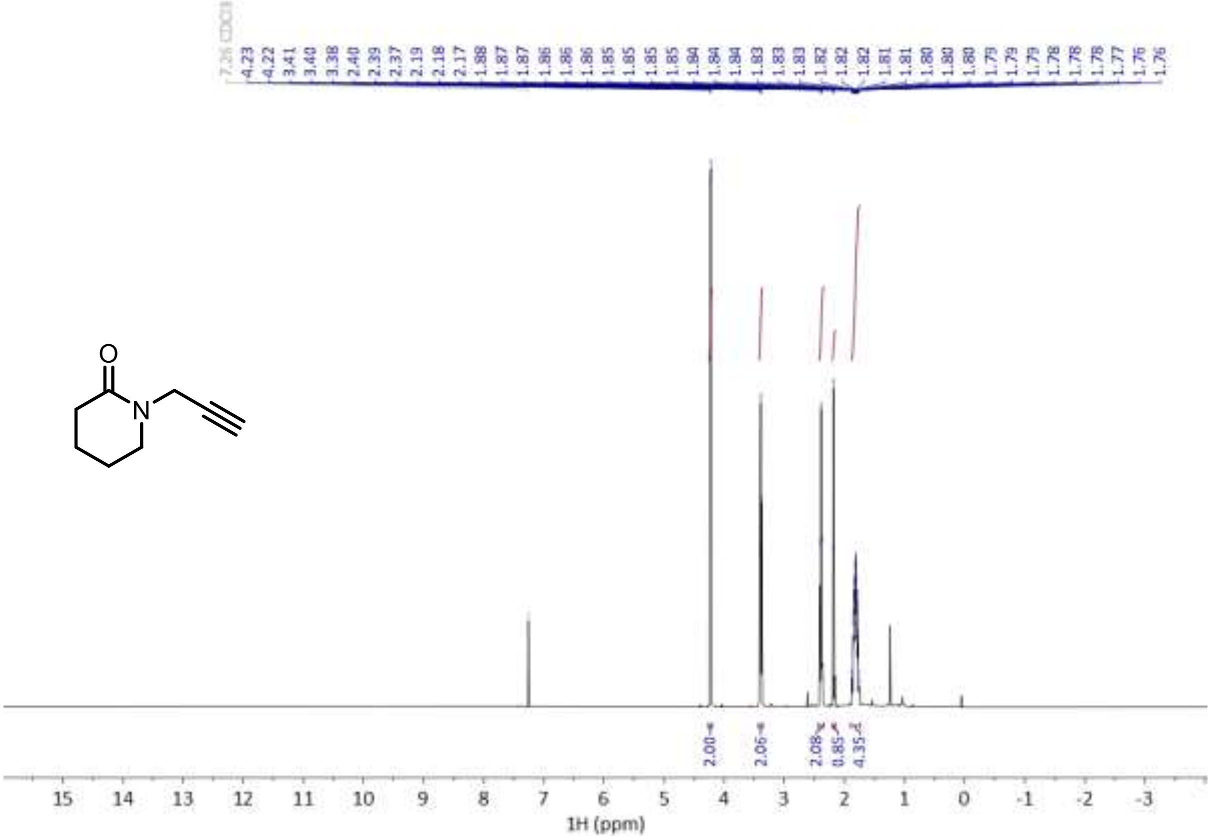

Compound 3l (¹³C)

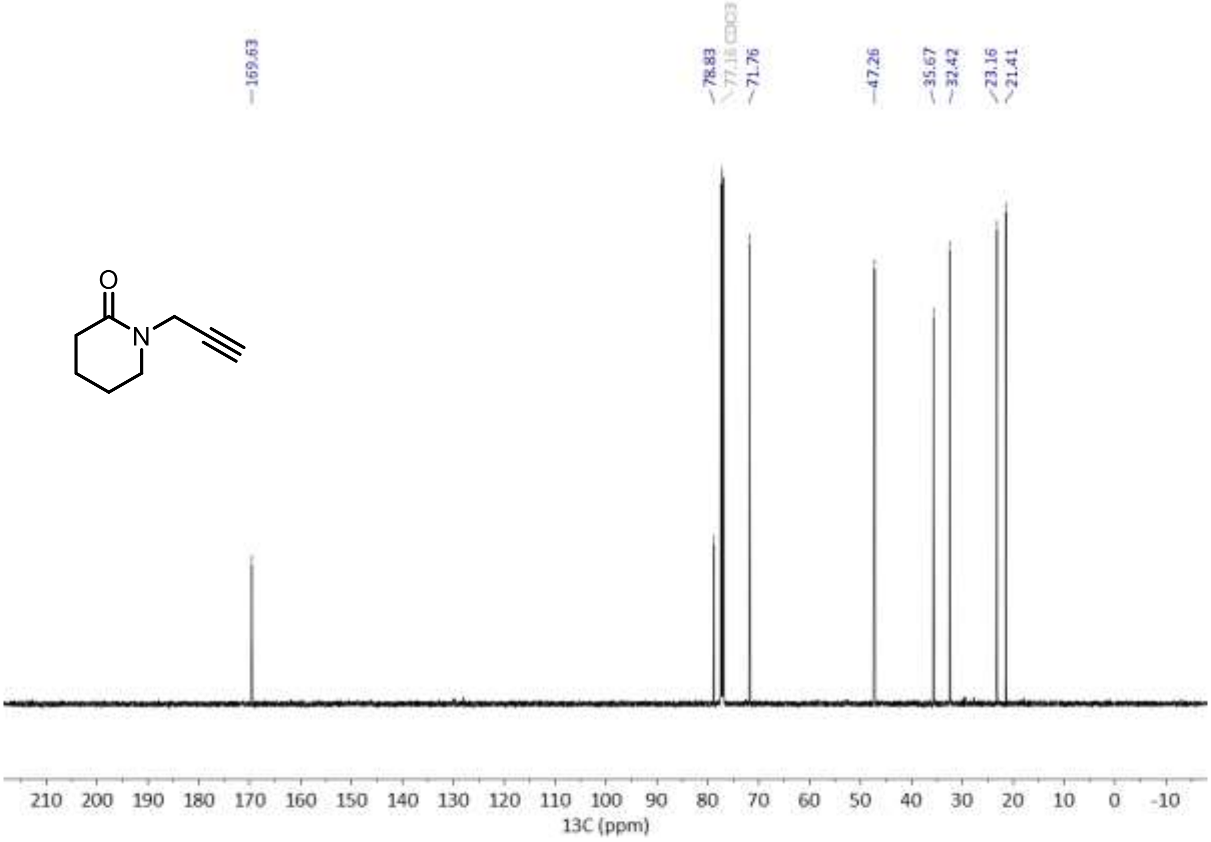

Compound 3m (^{1}H)

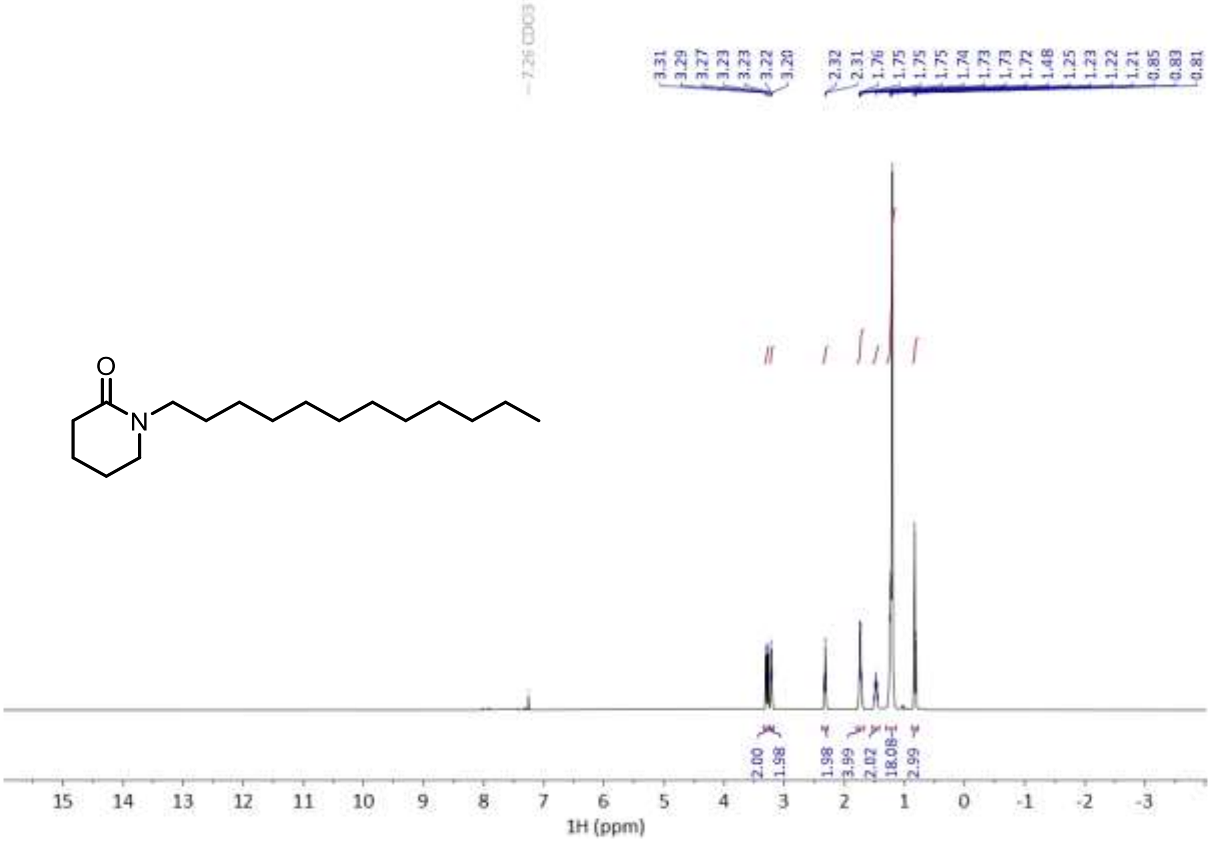

Compound 3m (^{13}C)

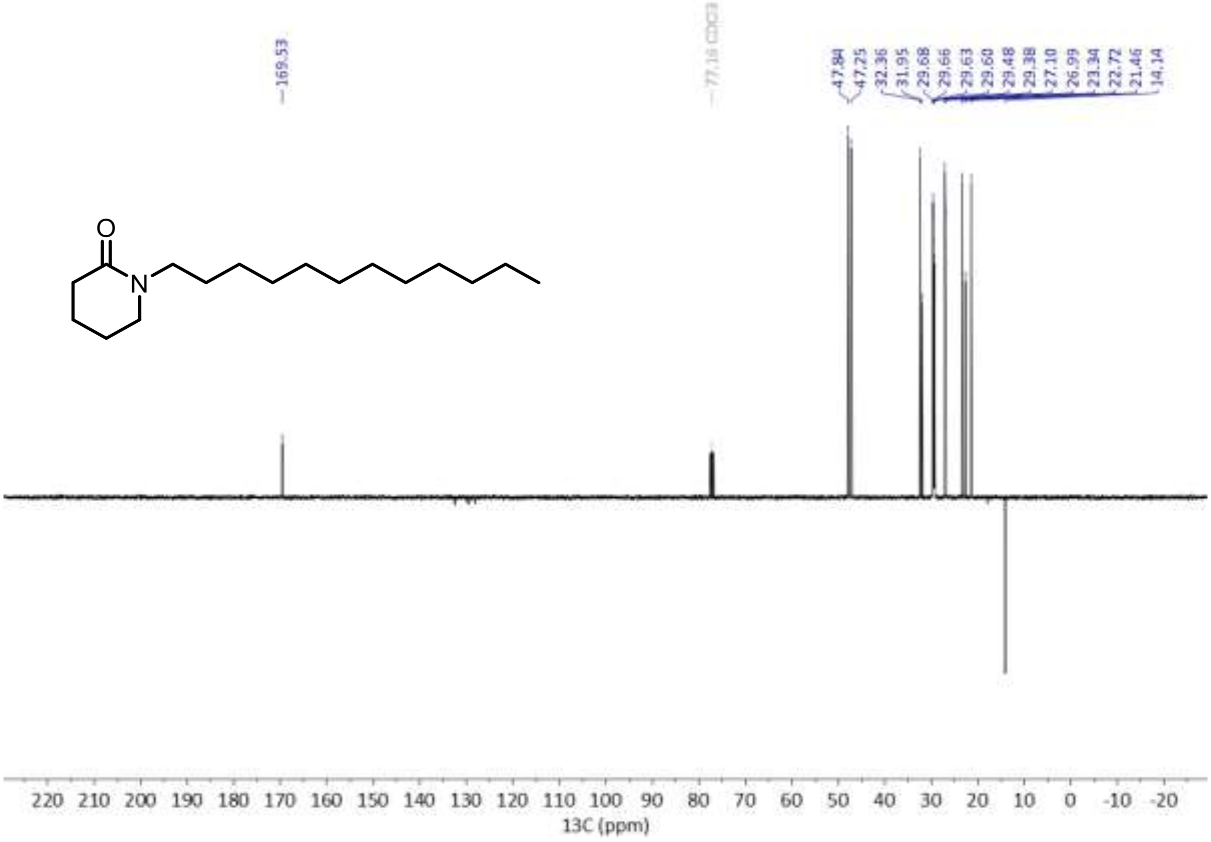

Compound 3n (^{1}H)

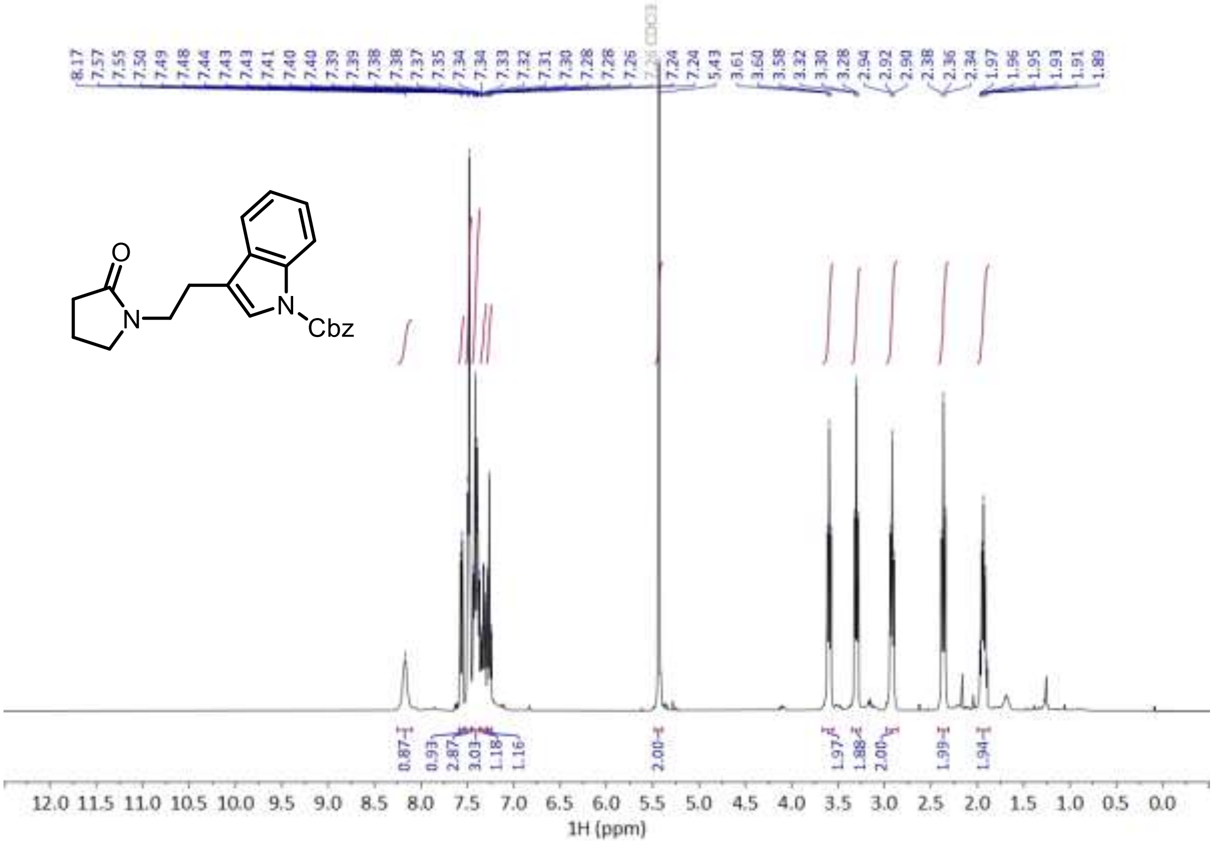

Compound 3n (^{13}C)

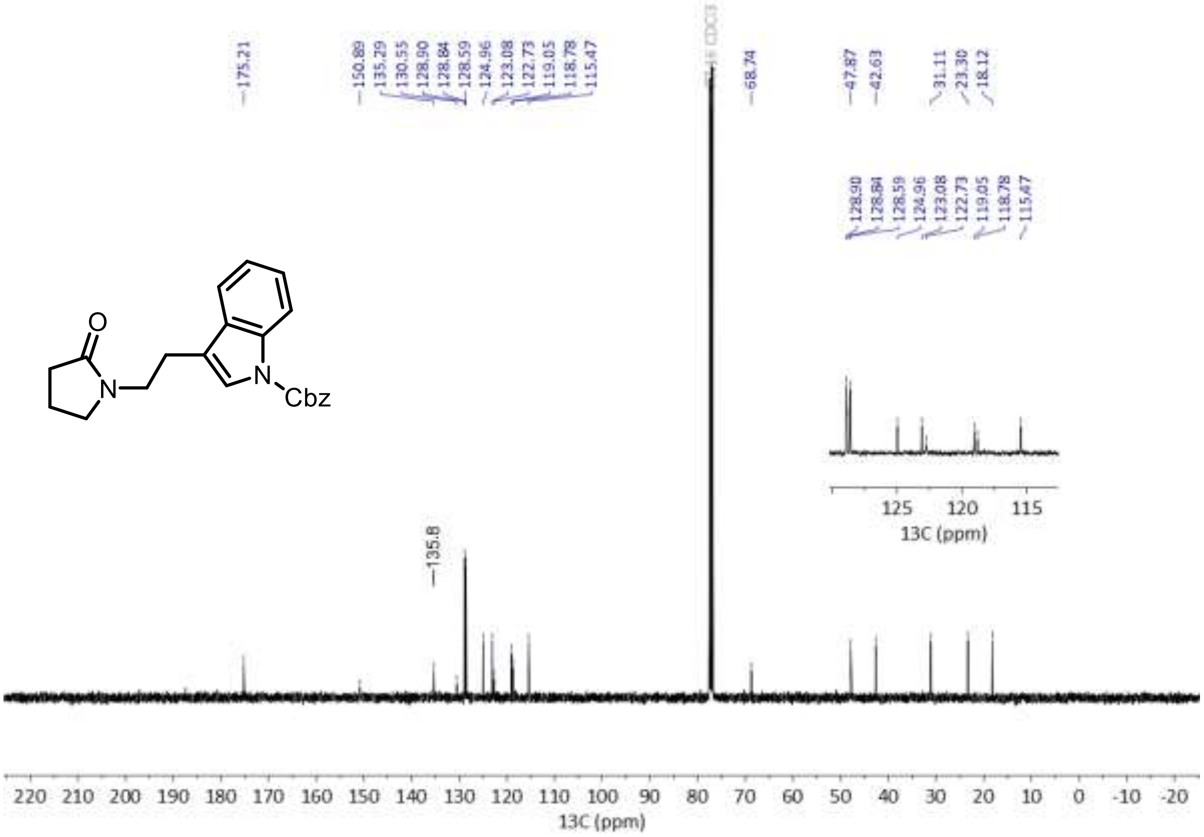

Compound 3o (^1H)

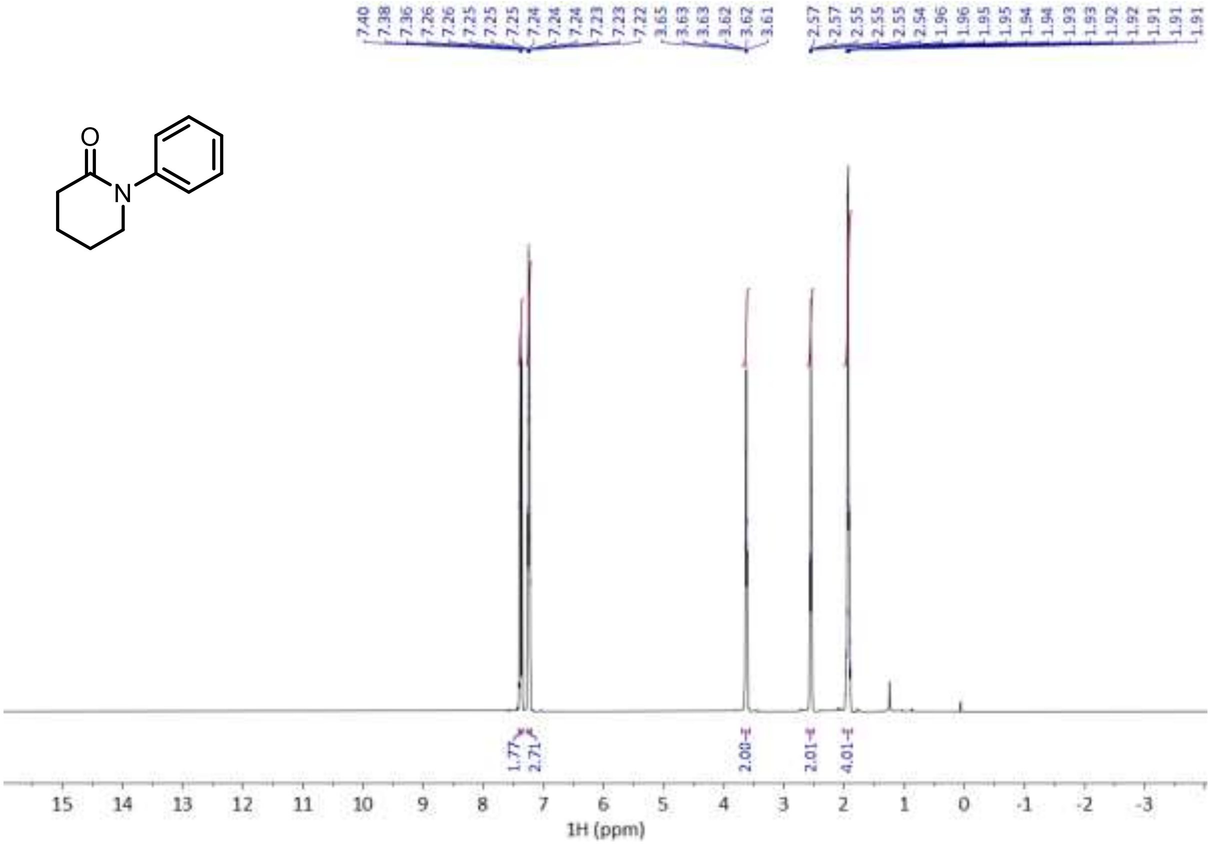

Compound 3o (^{13}C)

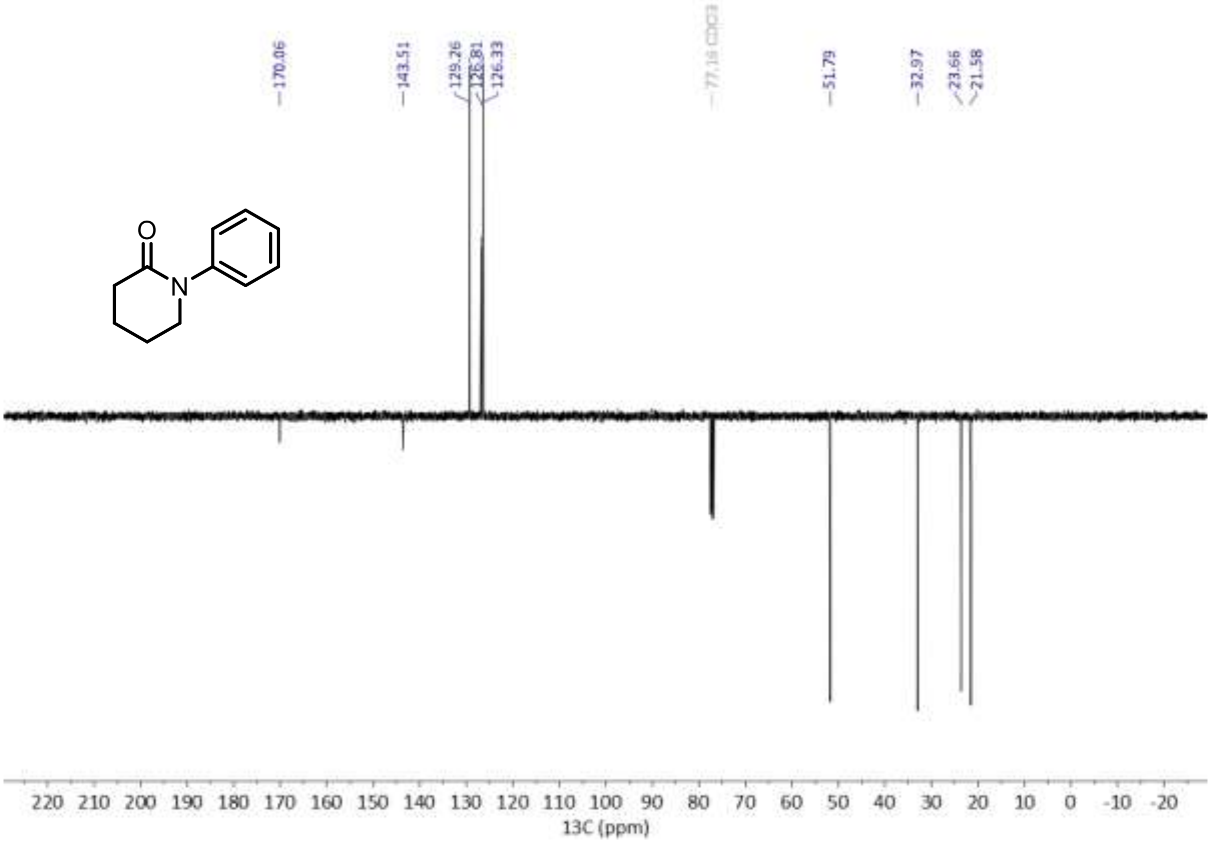

Compound 3p (^{1}H)

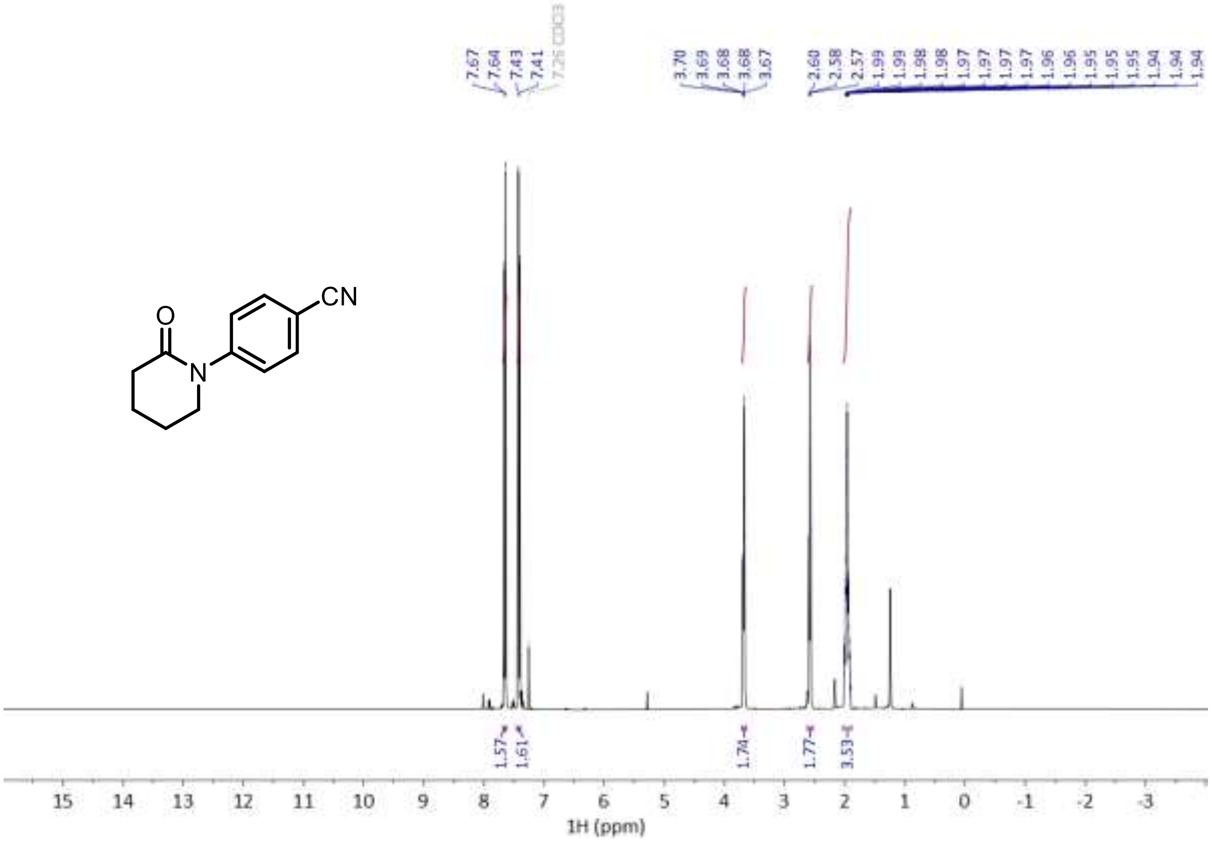

Compound 3p (^{13}C)

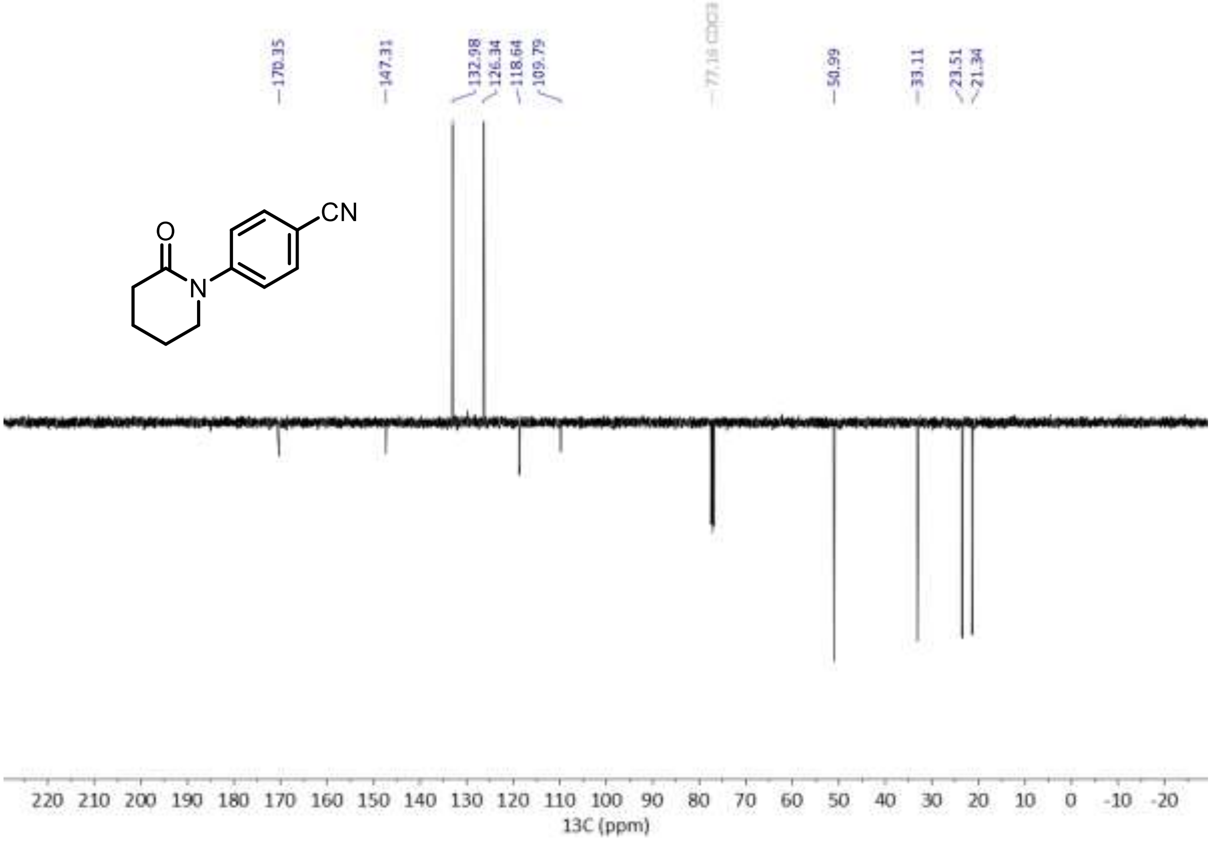

Compound 3q (^{1}H)

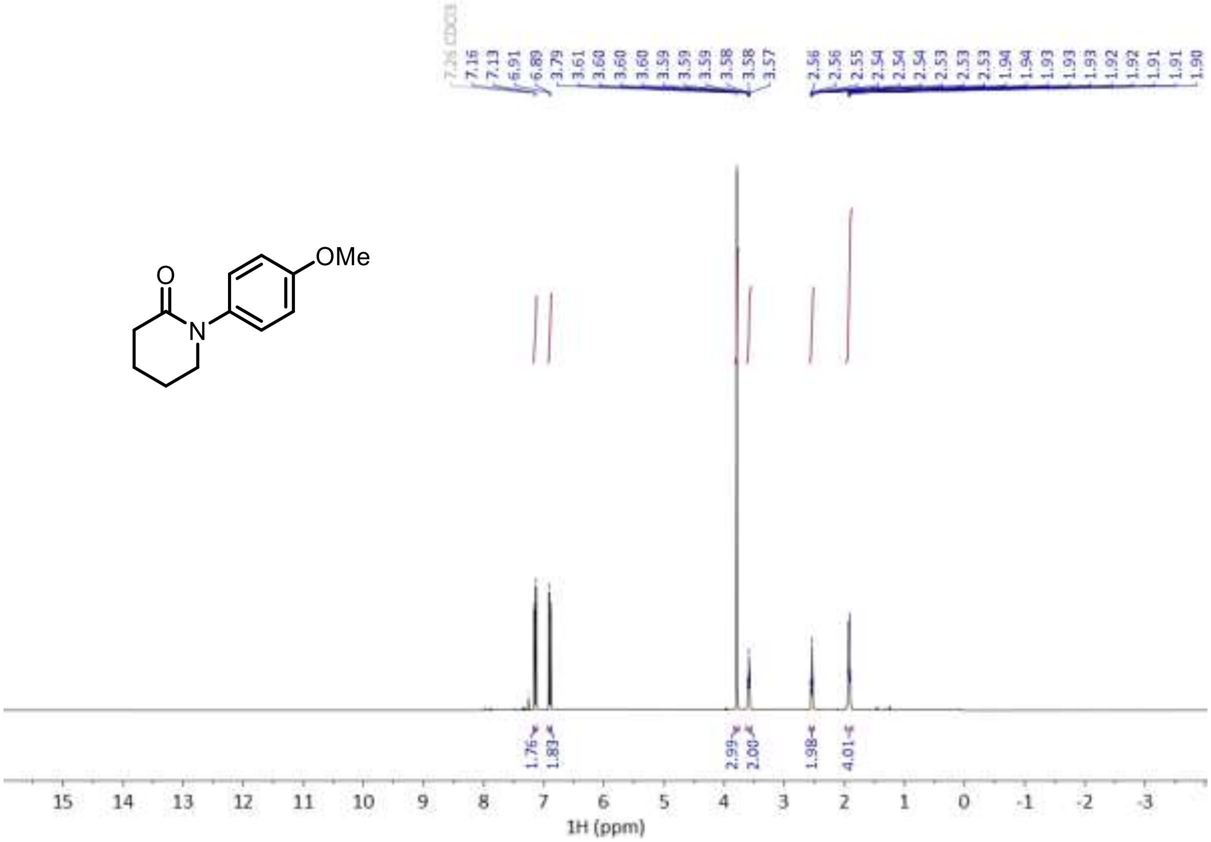

Compound 3q (^{13}C)

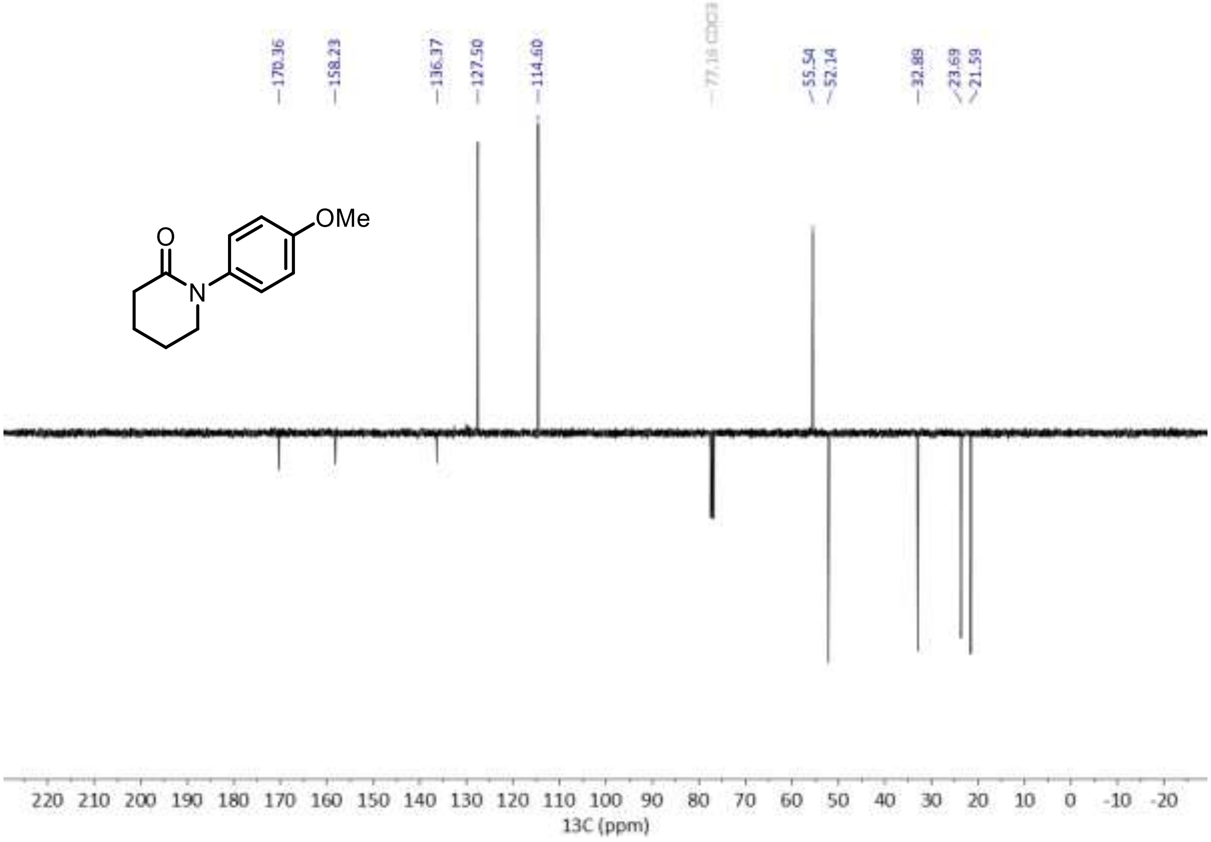

Compound 3r (^1H)

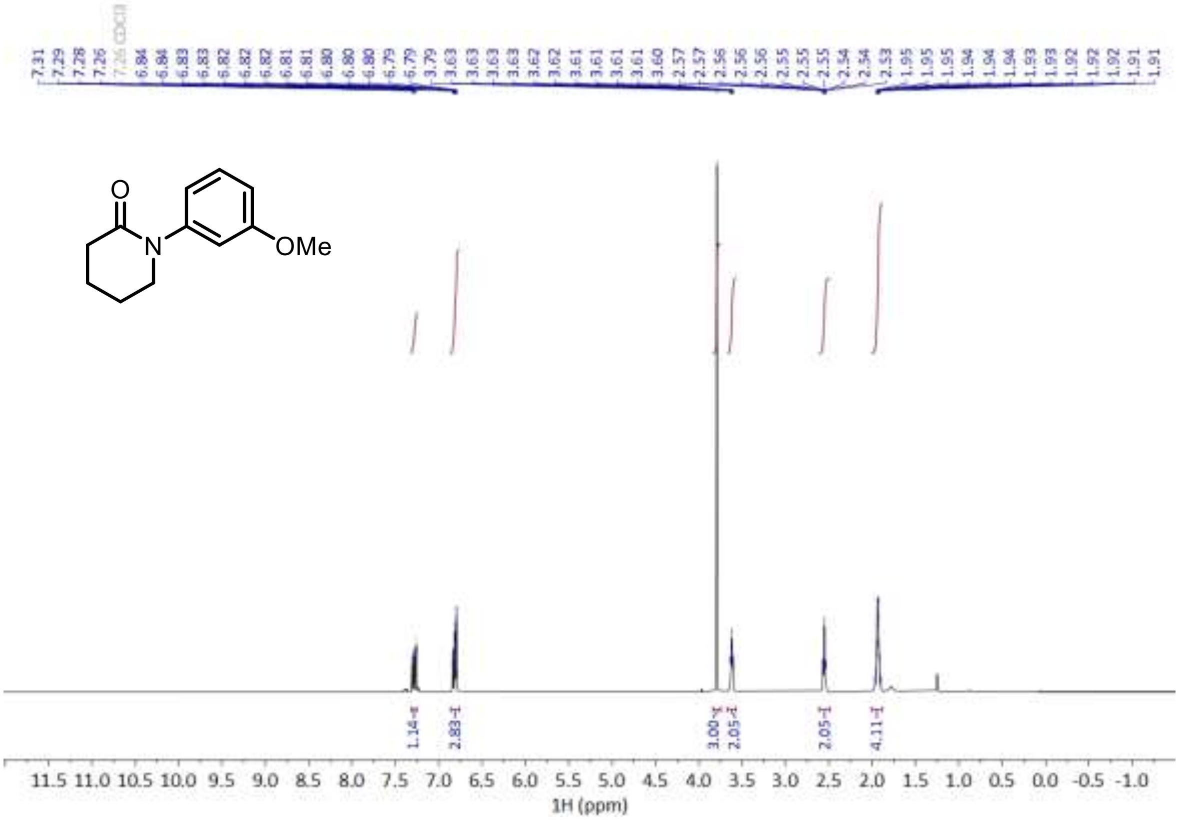

Compound 3r (^{13}C)

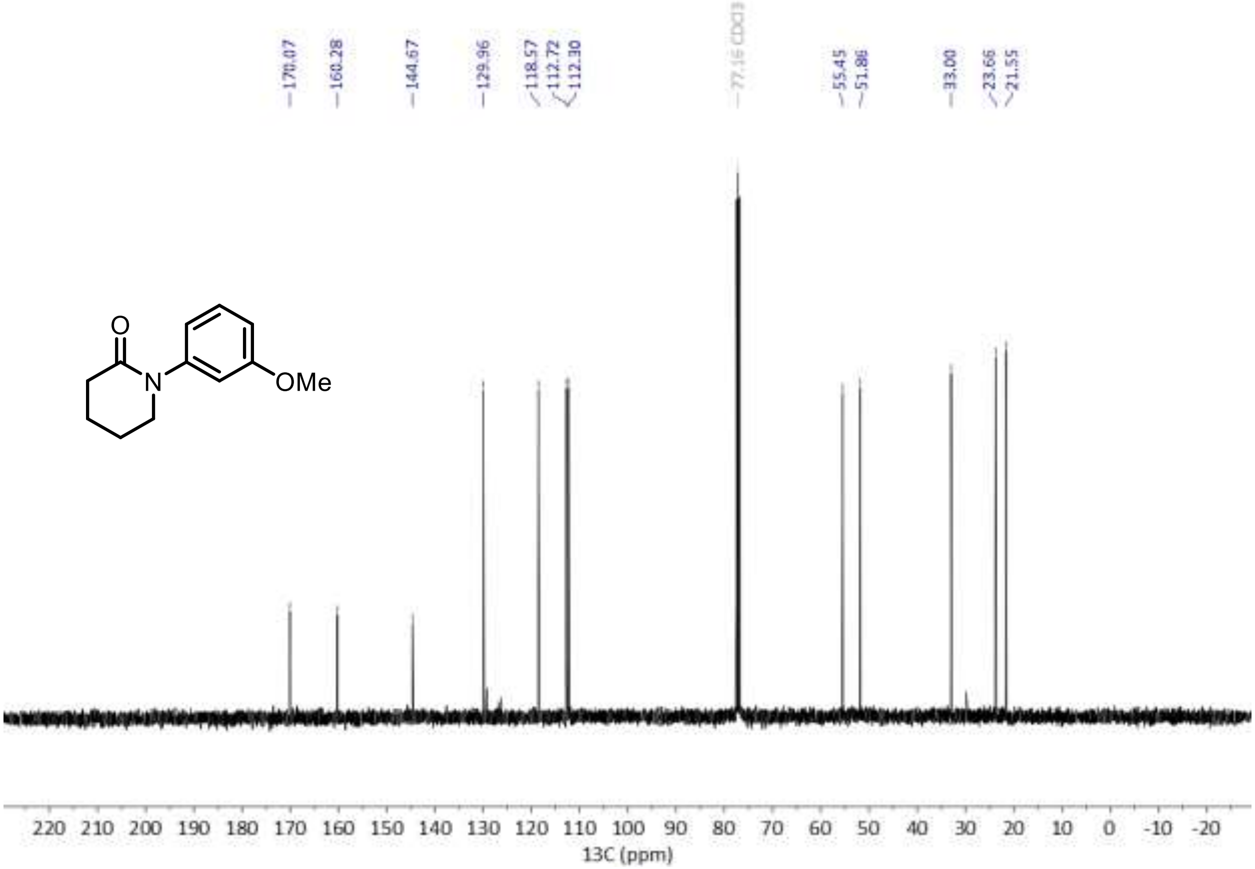

Compound 3s (^{1}H)

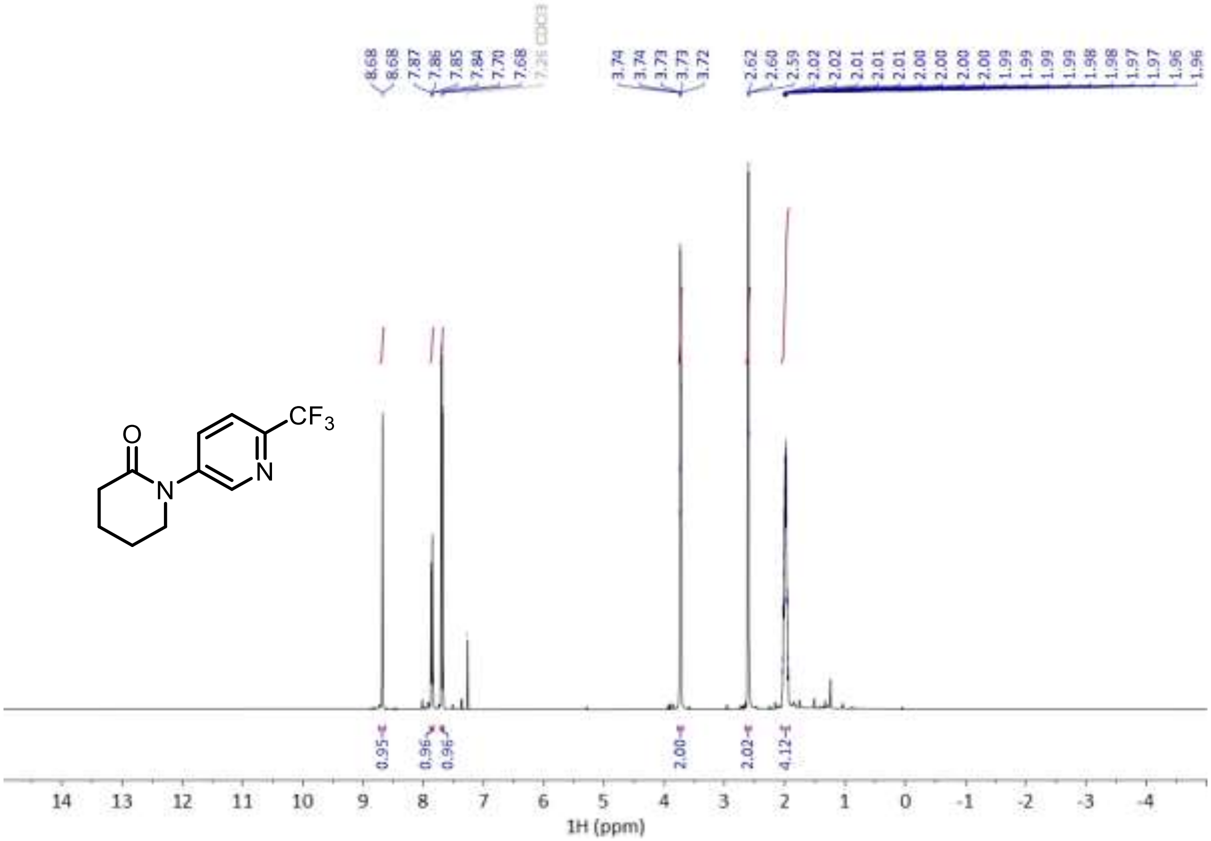

Compound 3s (^{13}C)

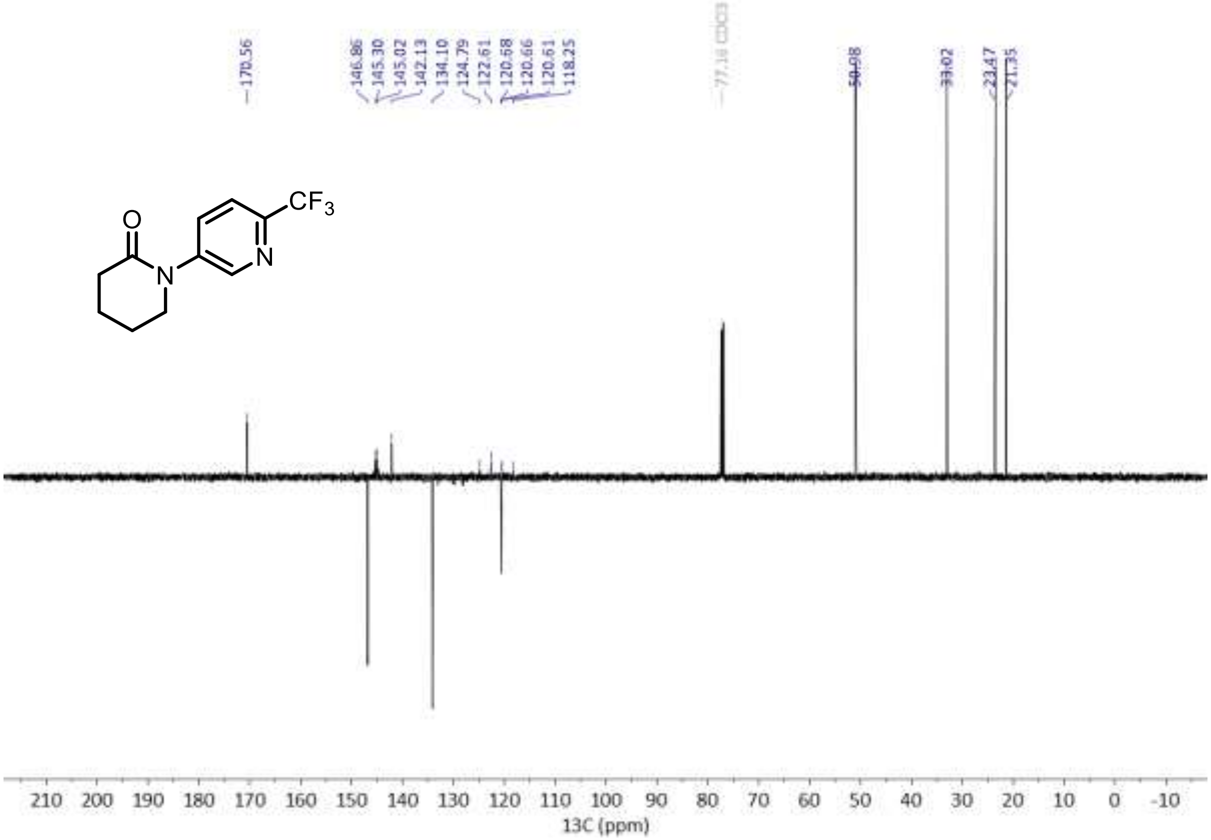

Compound 3f (^{19}F)

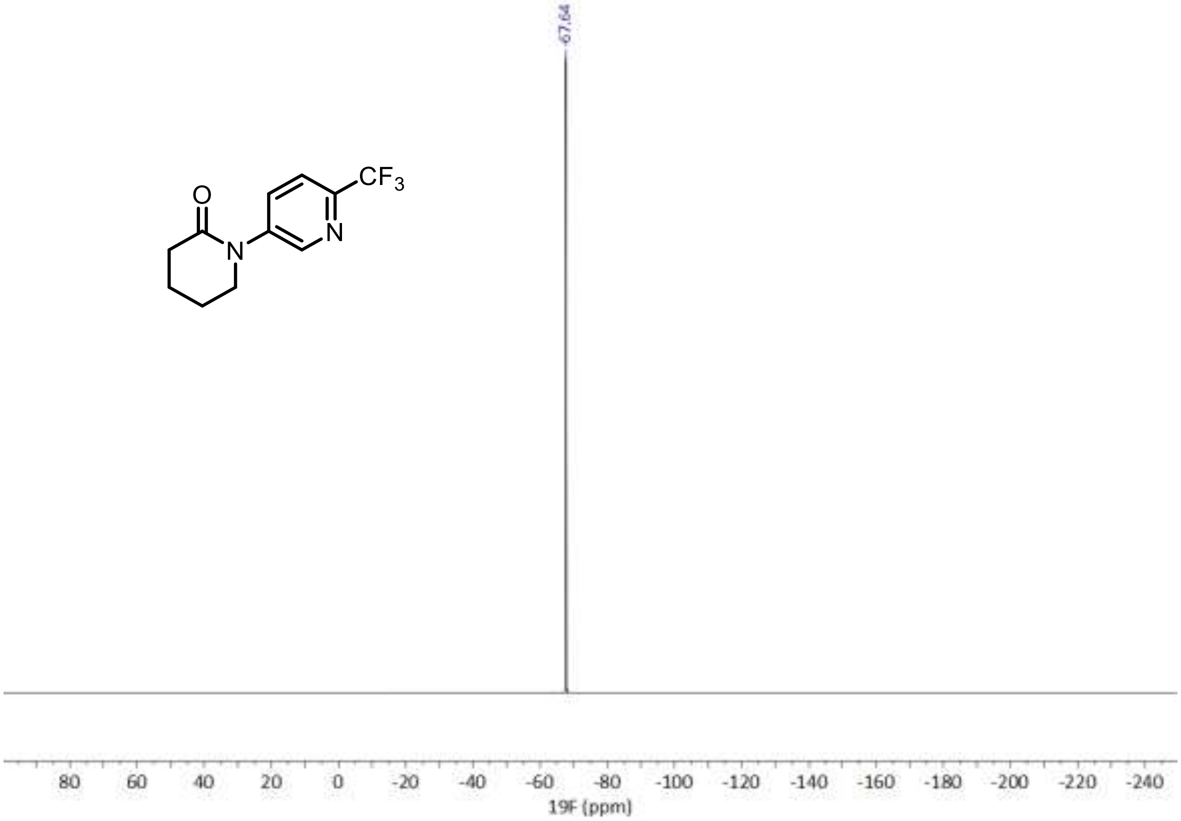

Compound 3t (¹H)

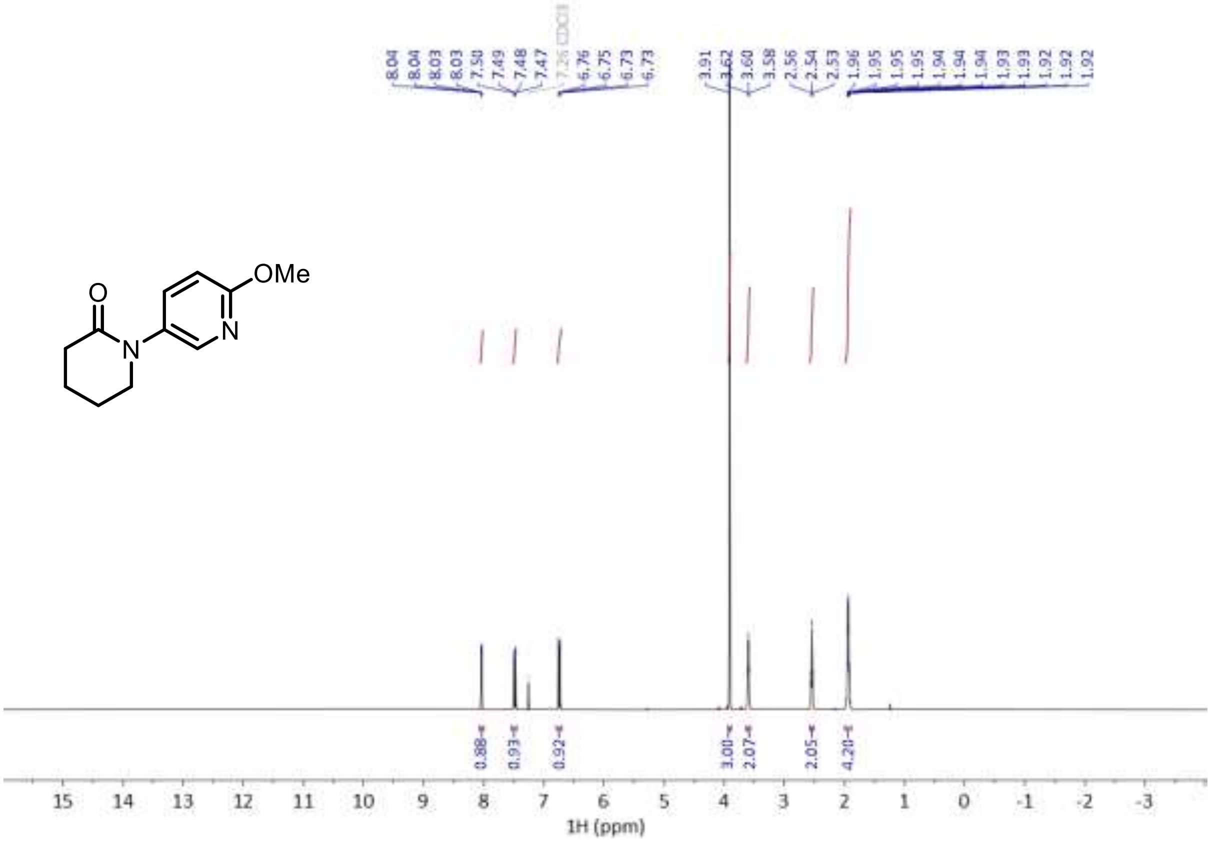

Compound 3t (¹³C)

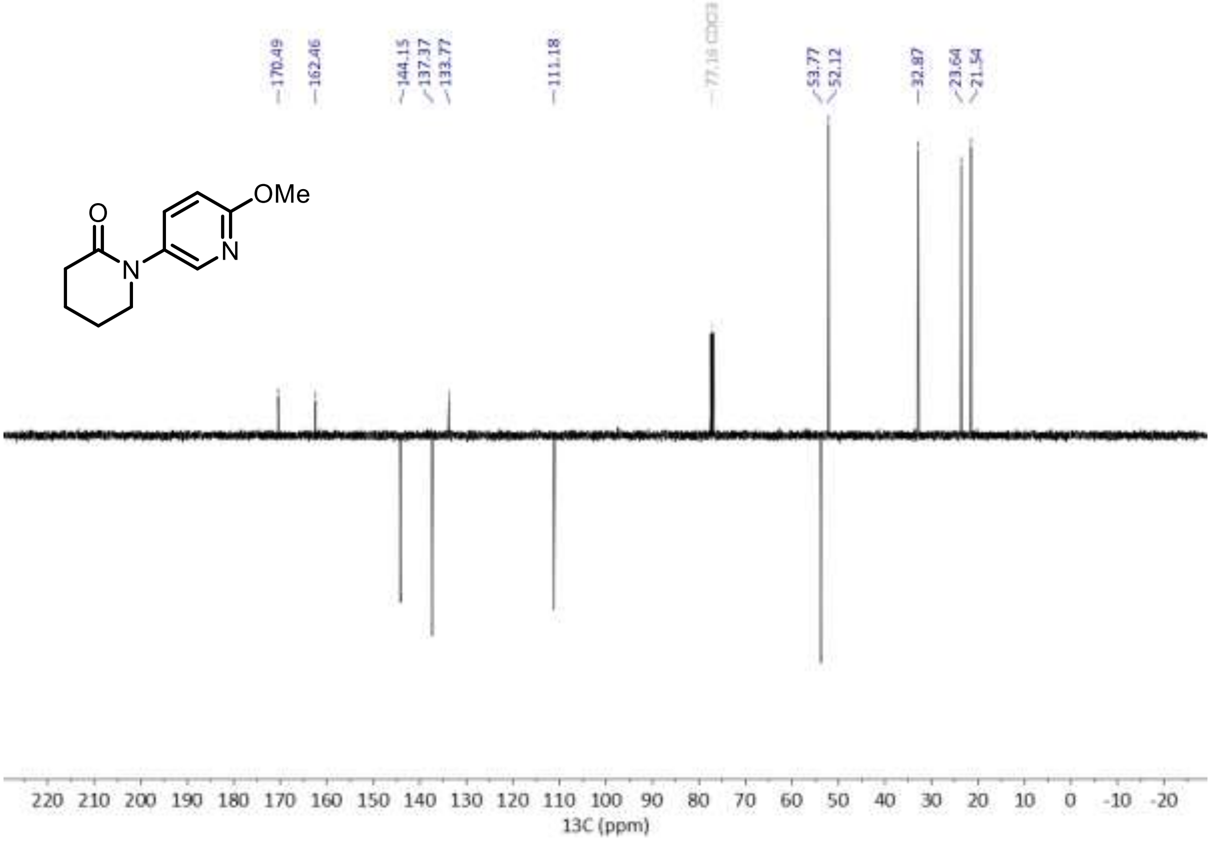

Compound 3u (^{1}H)

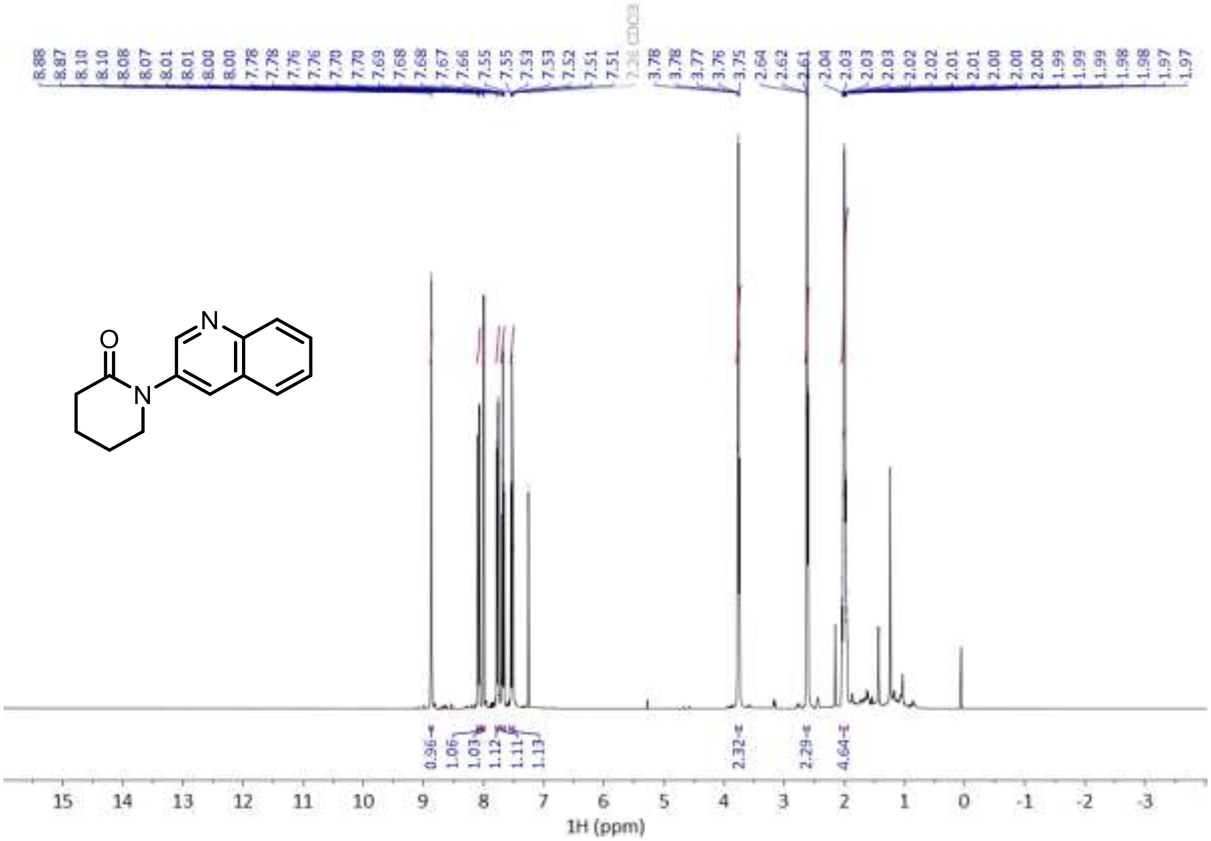

Compound 3u (^{13}C)

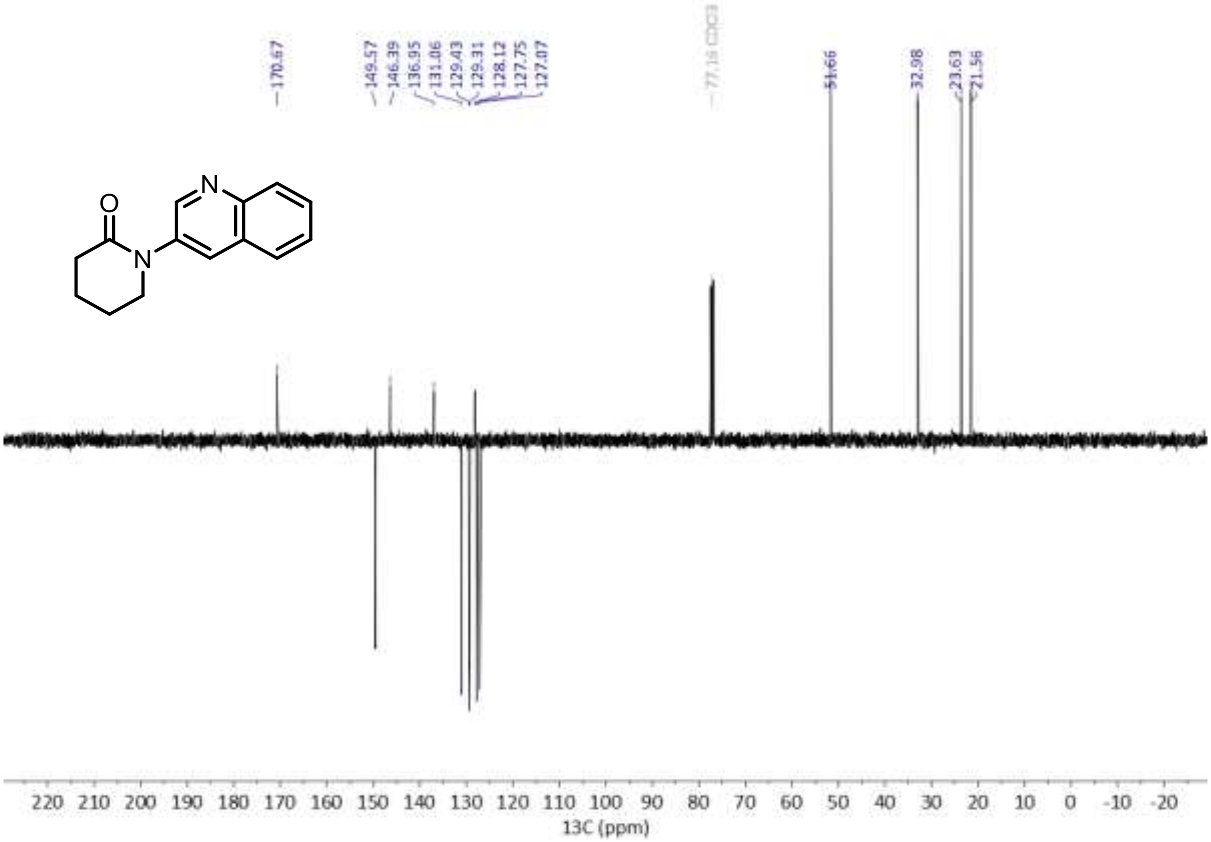

Compound 3v (^{1}H)

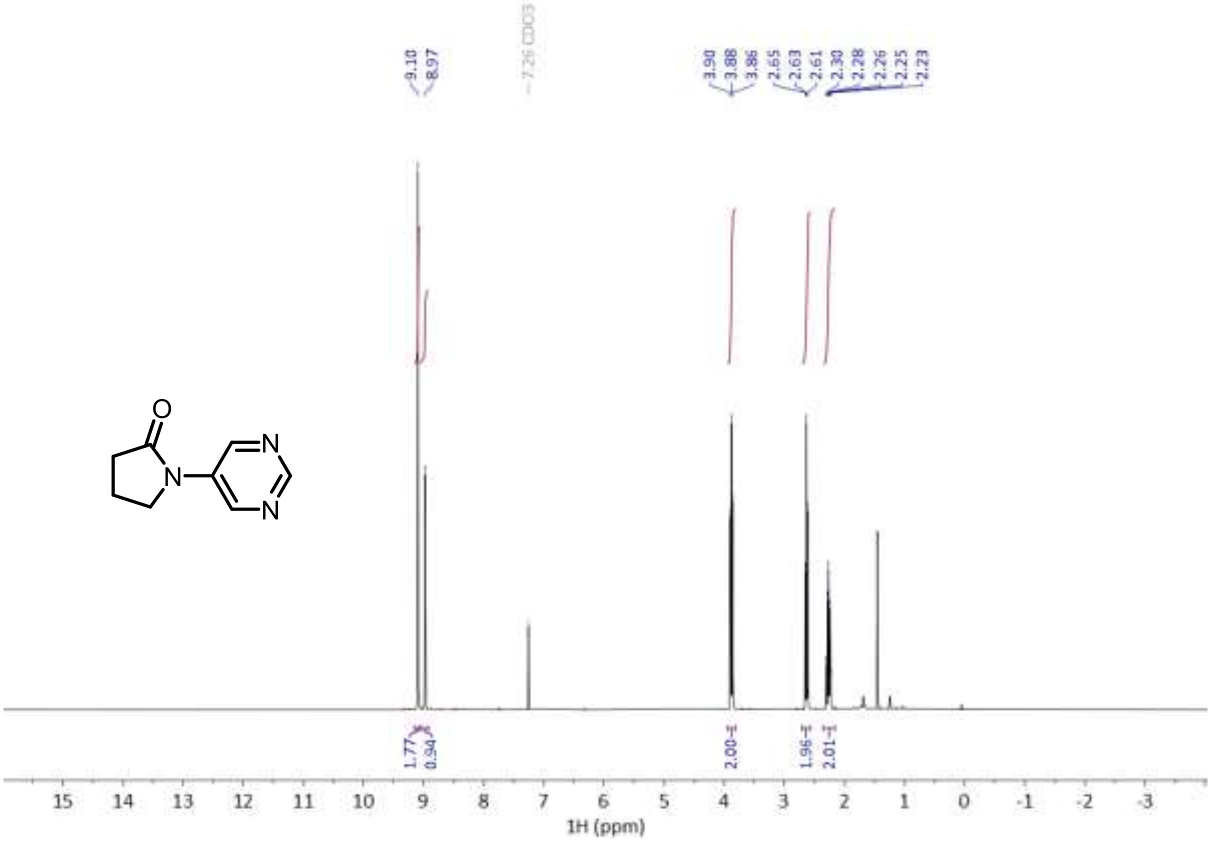

Compound 3v (^{13}C)

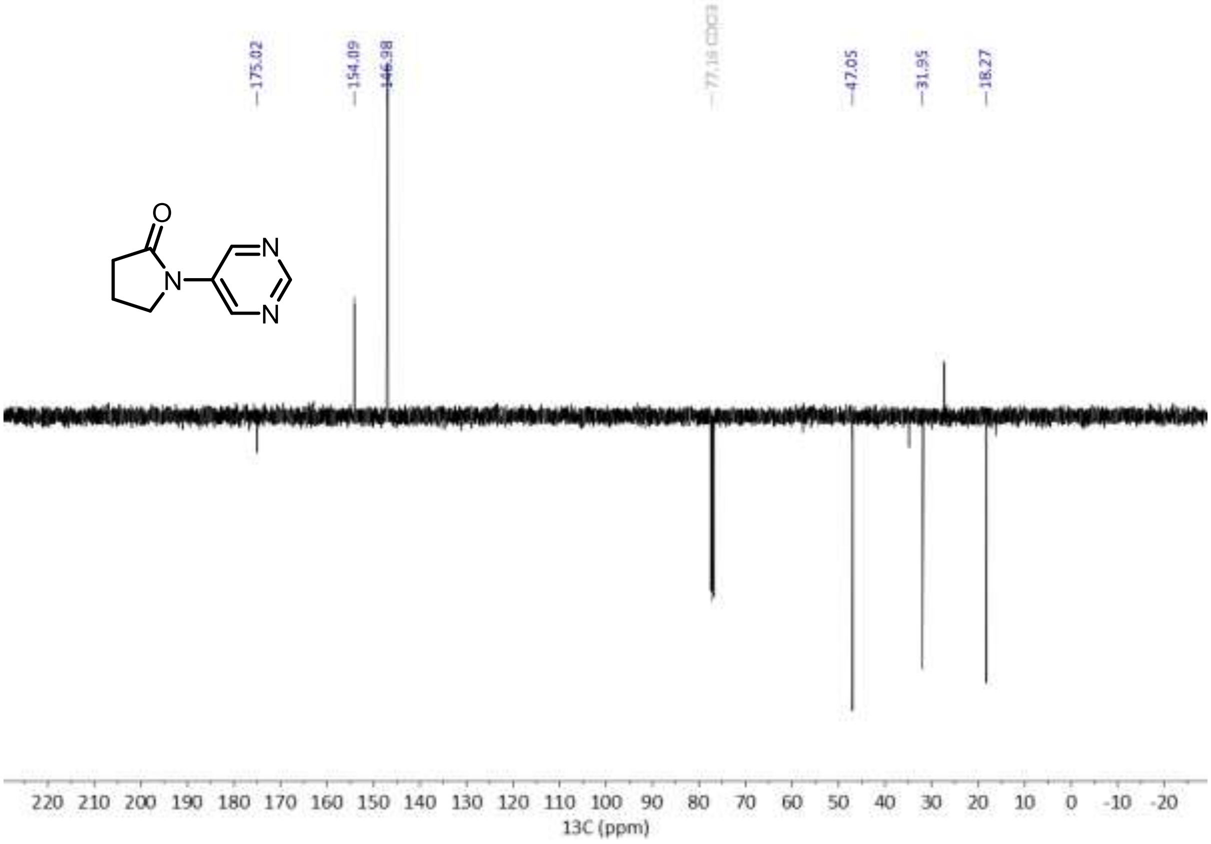

Compound 3w (^{1}H)

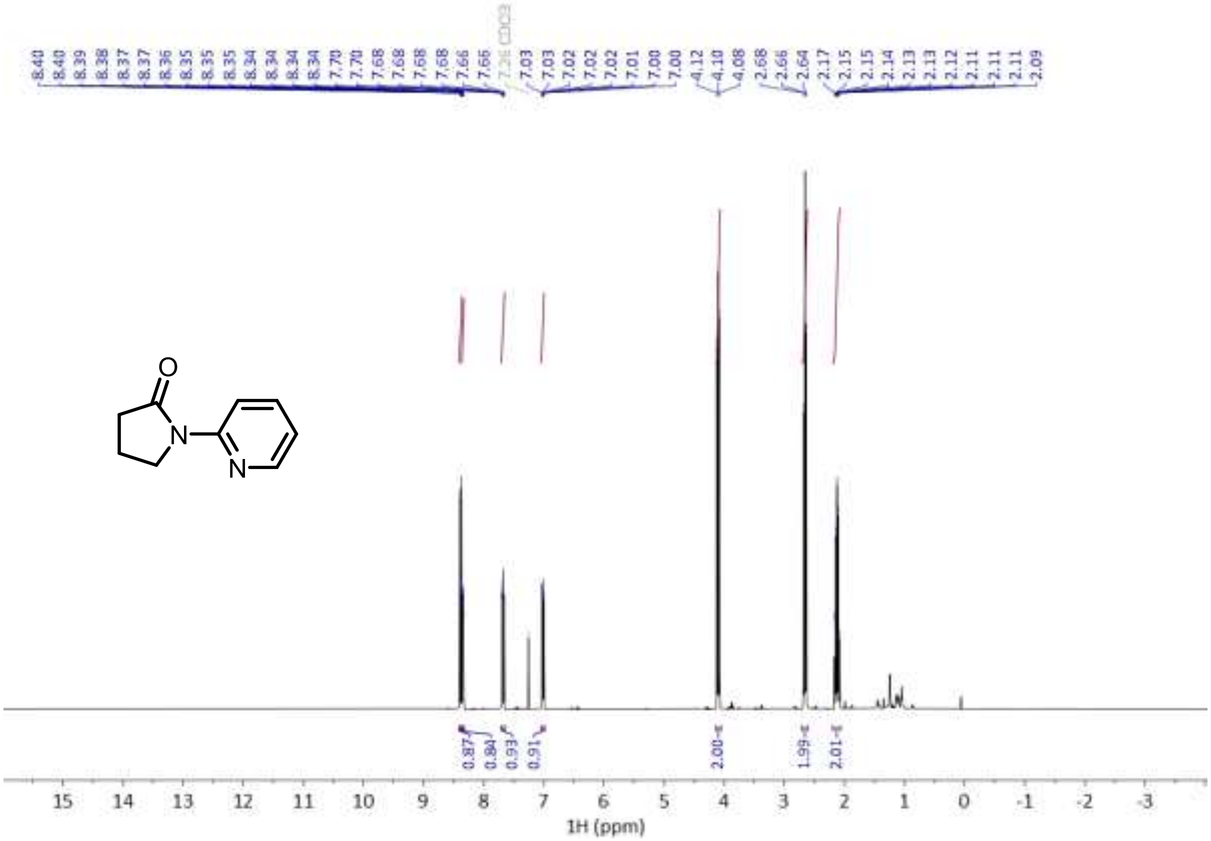

Compound 3w (^{13}C)

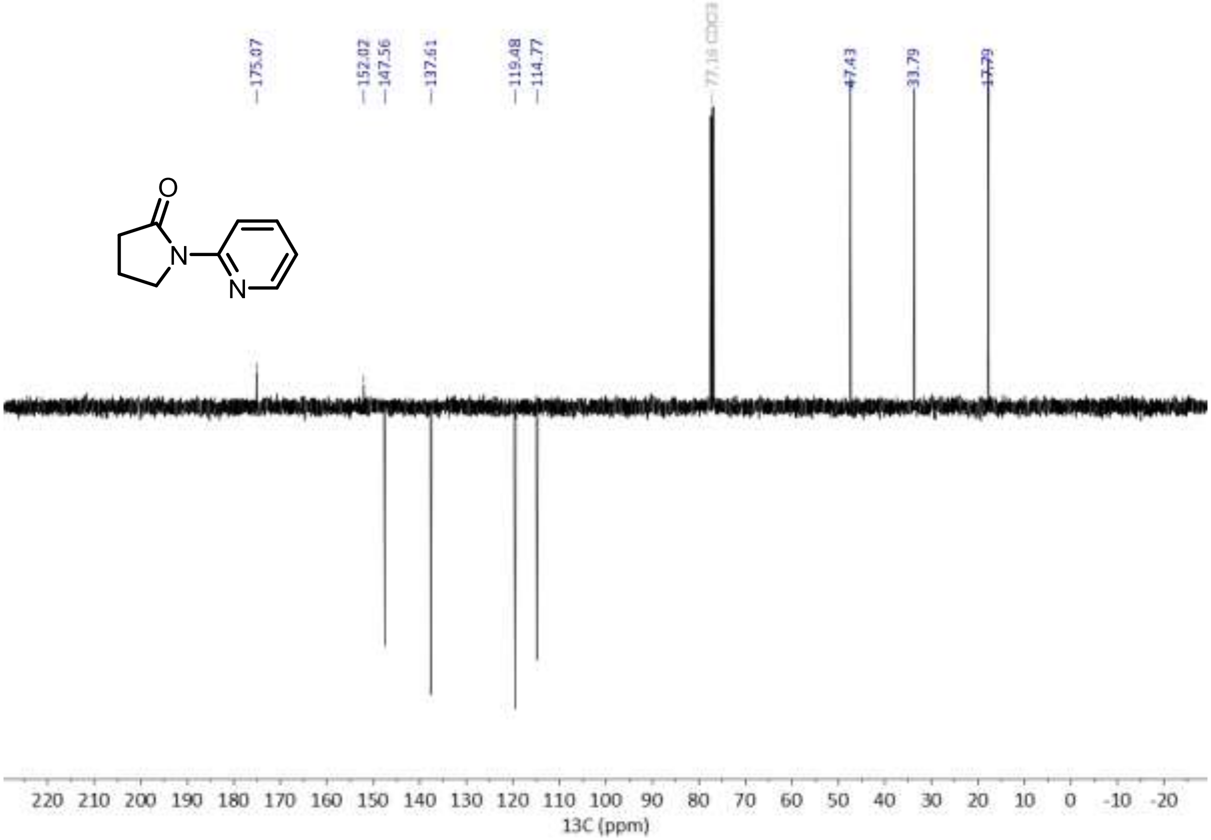

Compound 3x (^{1}H)

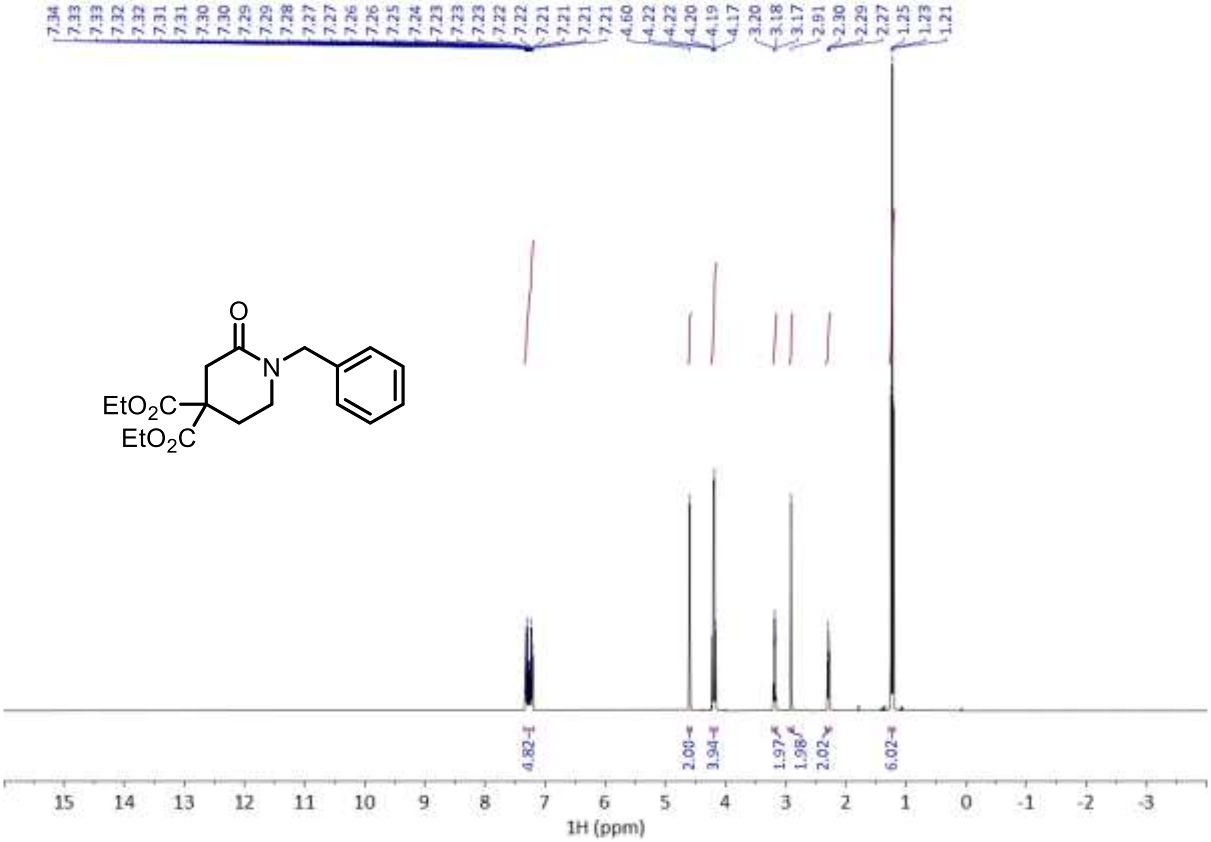

Compound 3x (^{13}C)

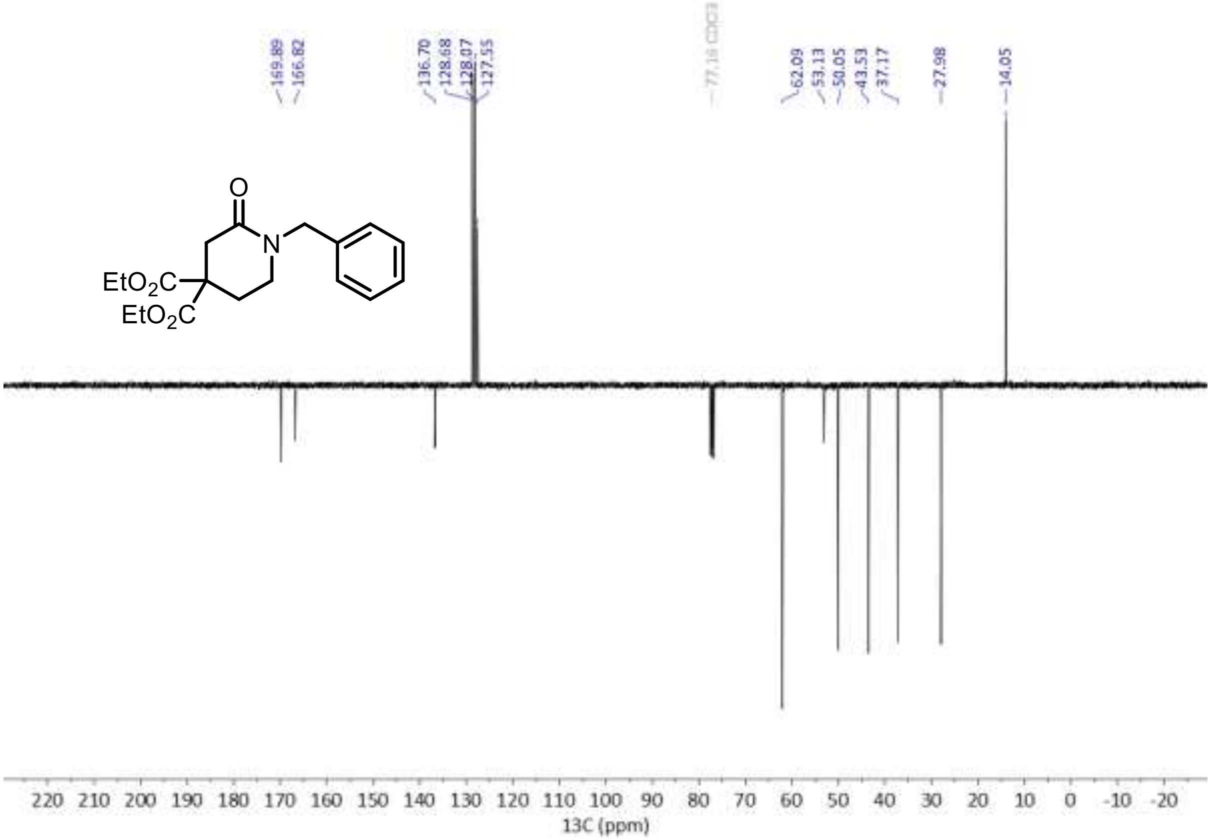

Compound 3y (^{1}H)

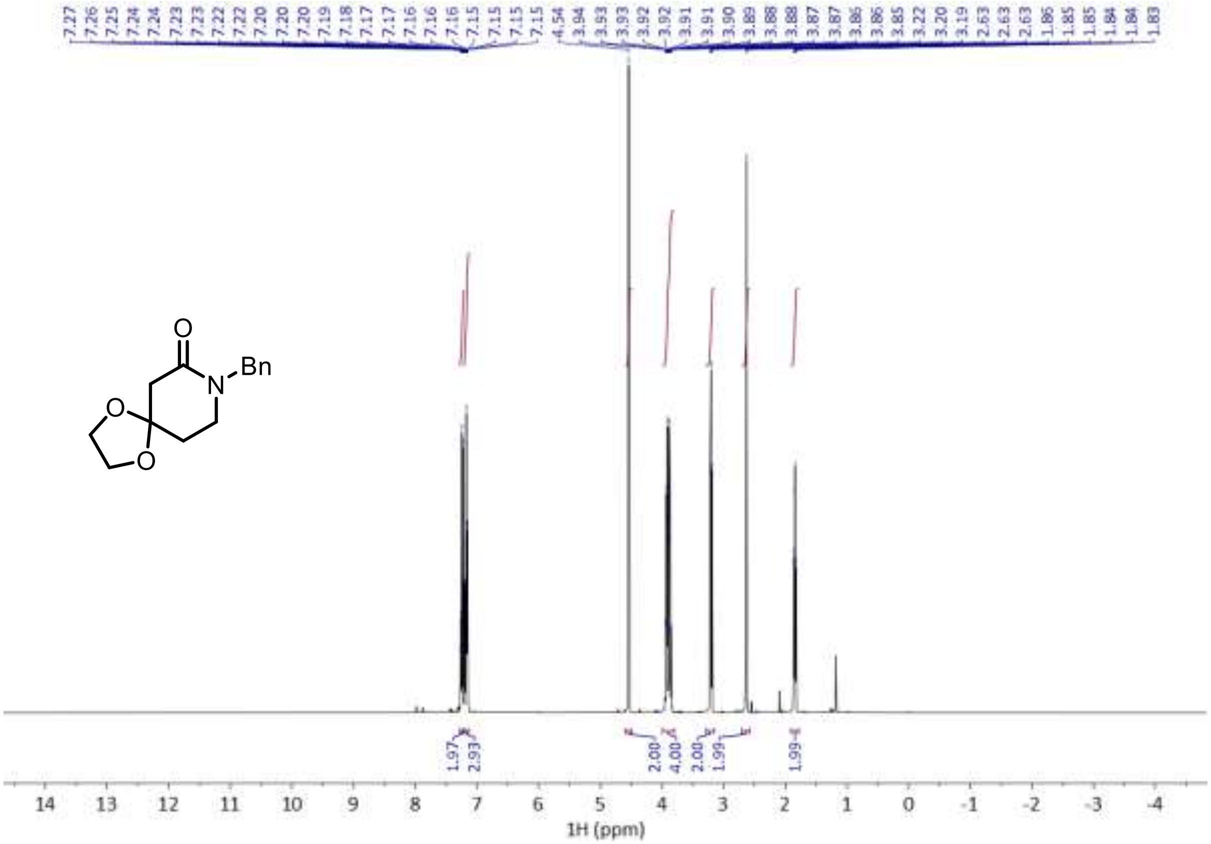

Compound 3y (^{13}C)

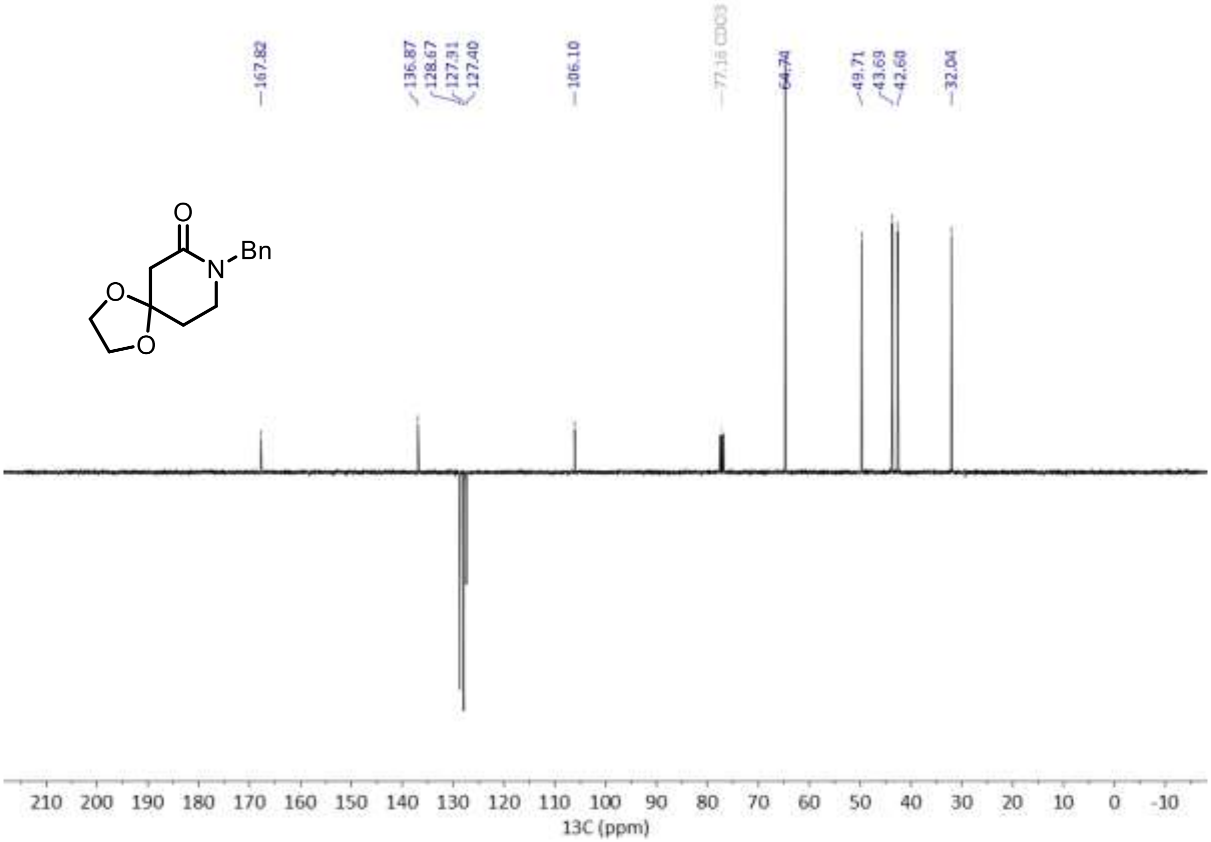

Compound 3z (¹H)

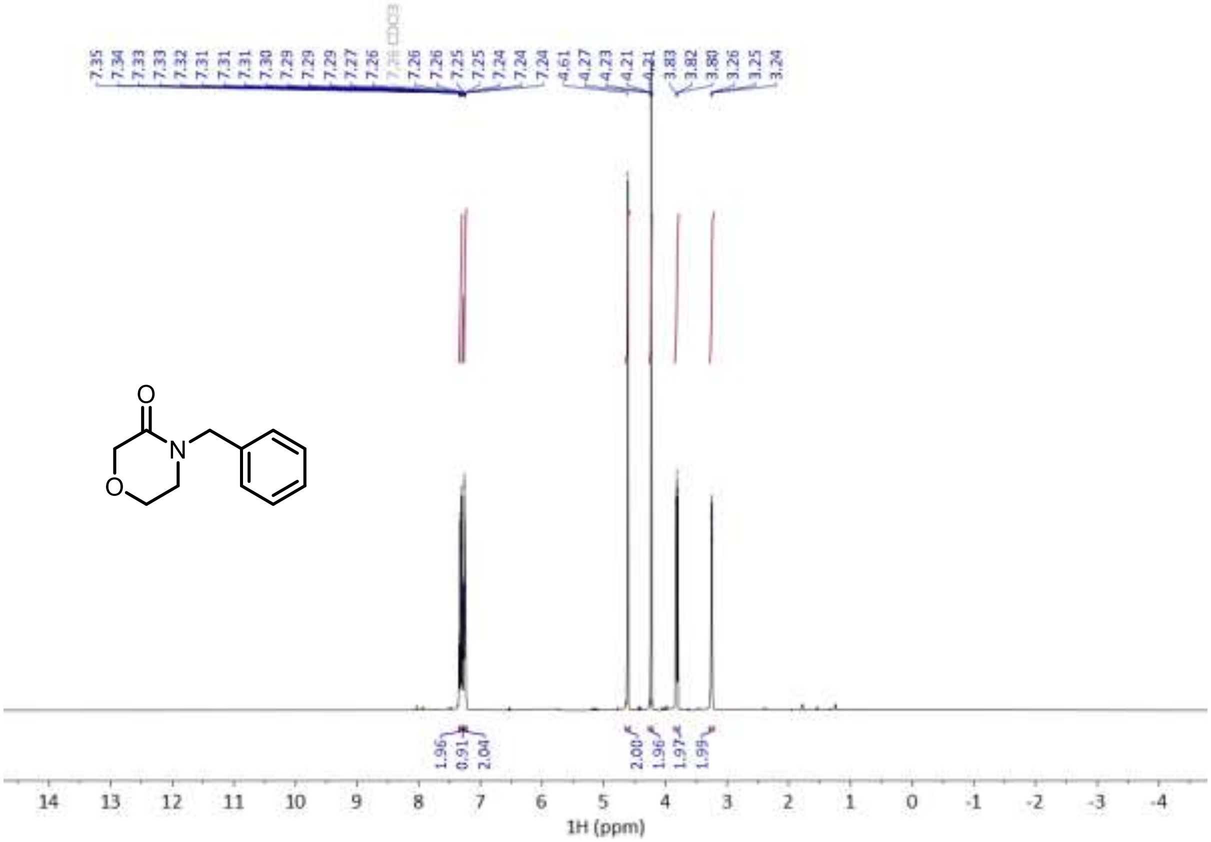

Compound 3z (¹³C)

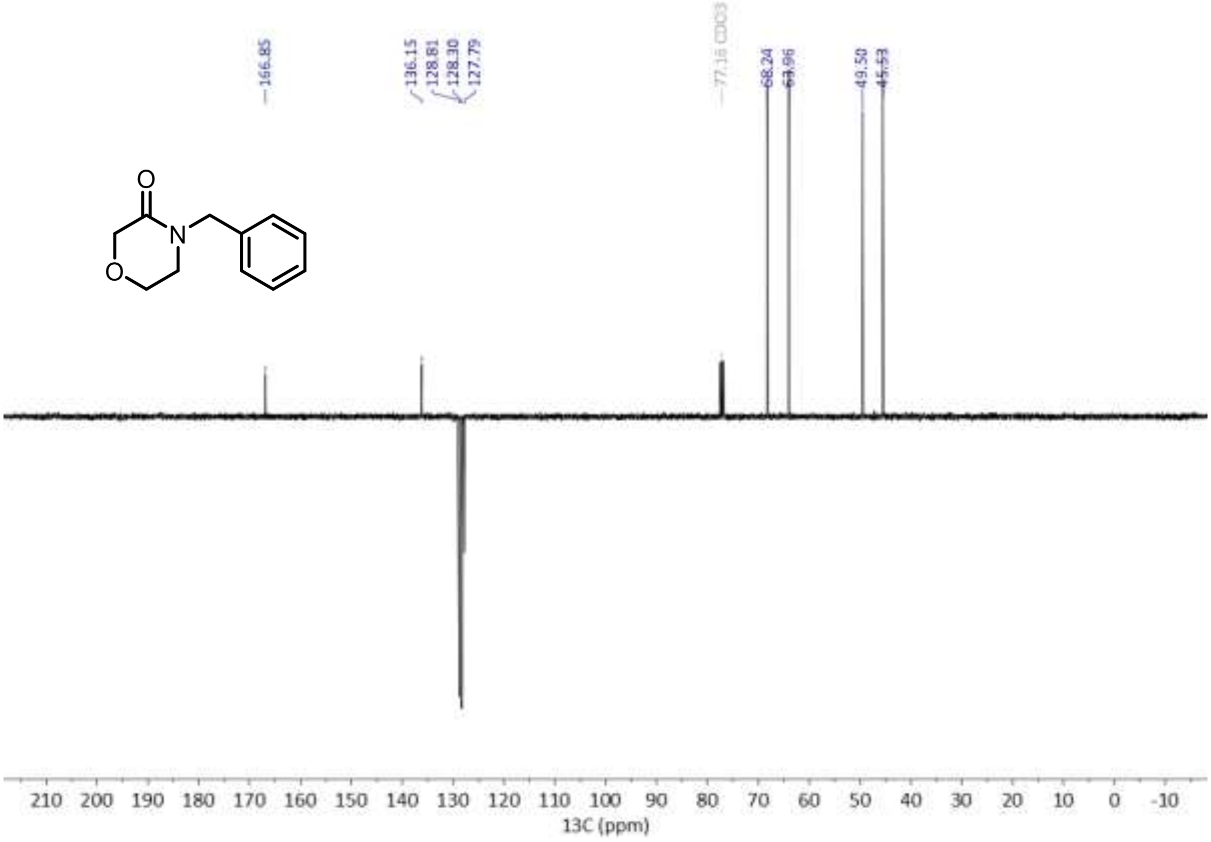

Compound 3aa (^{1}H)

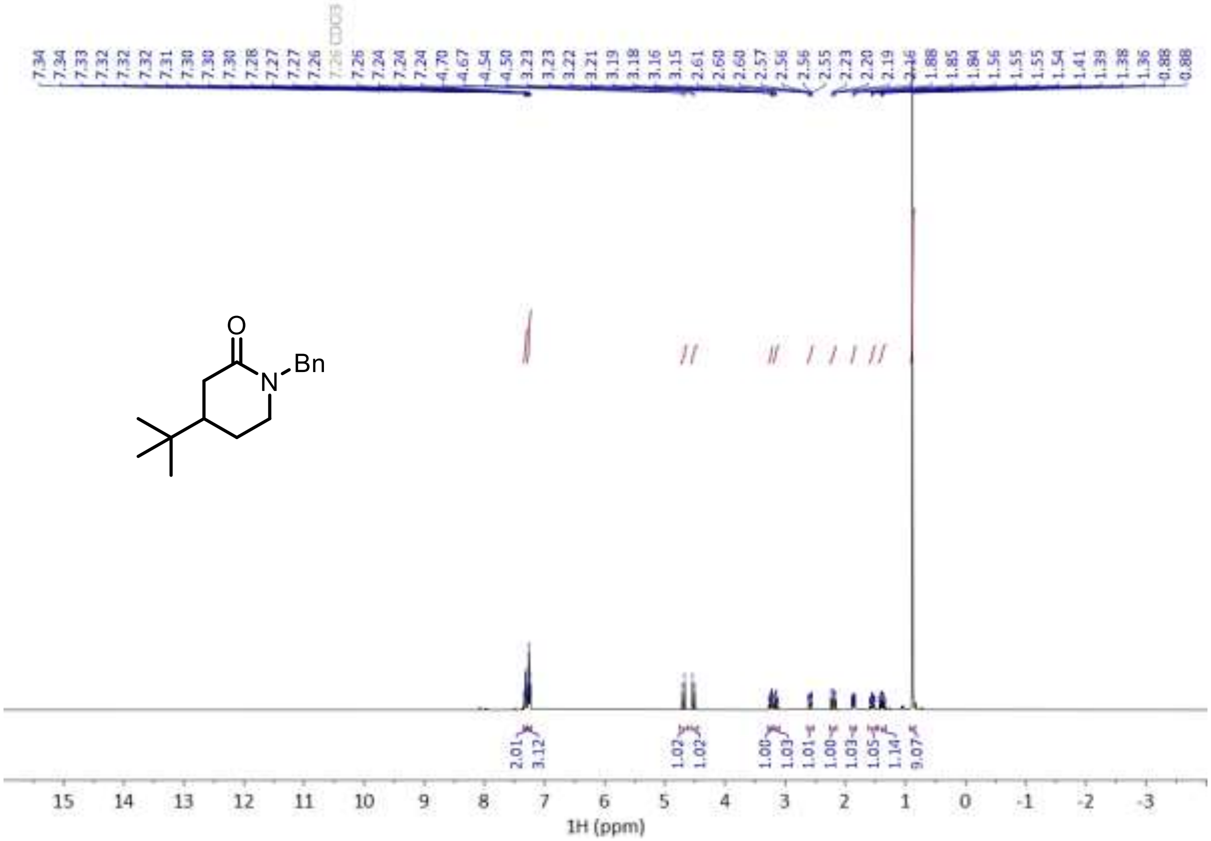

Compound 3aa (^{13}C)

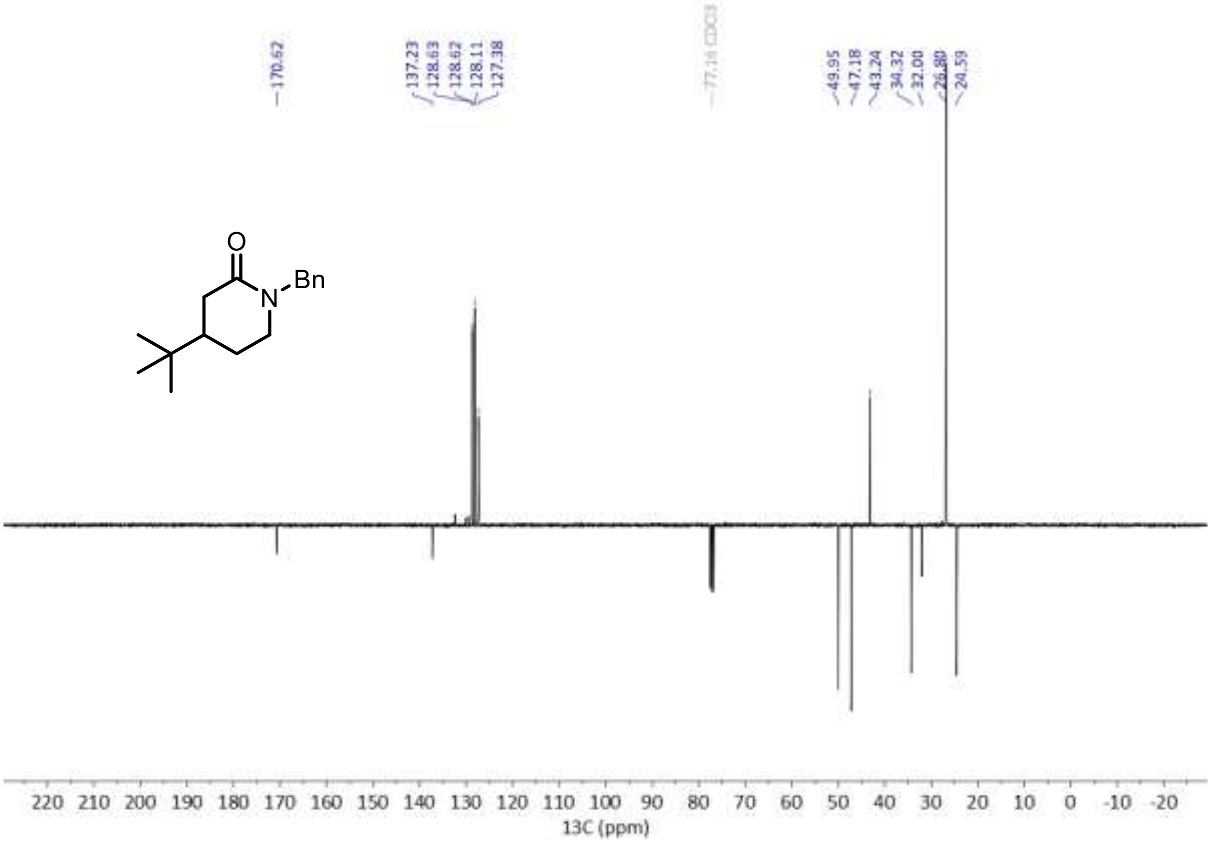

Compound 3ab (^{1}H)

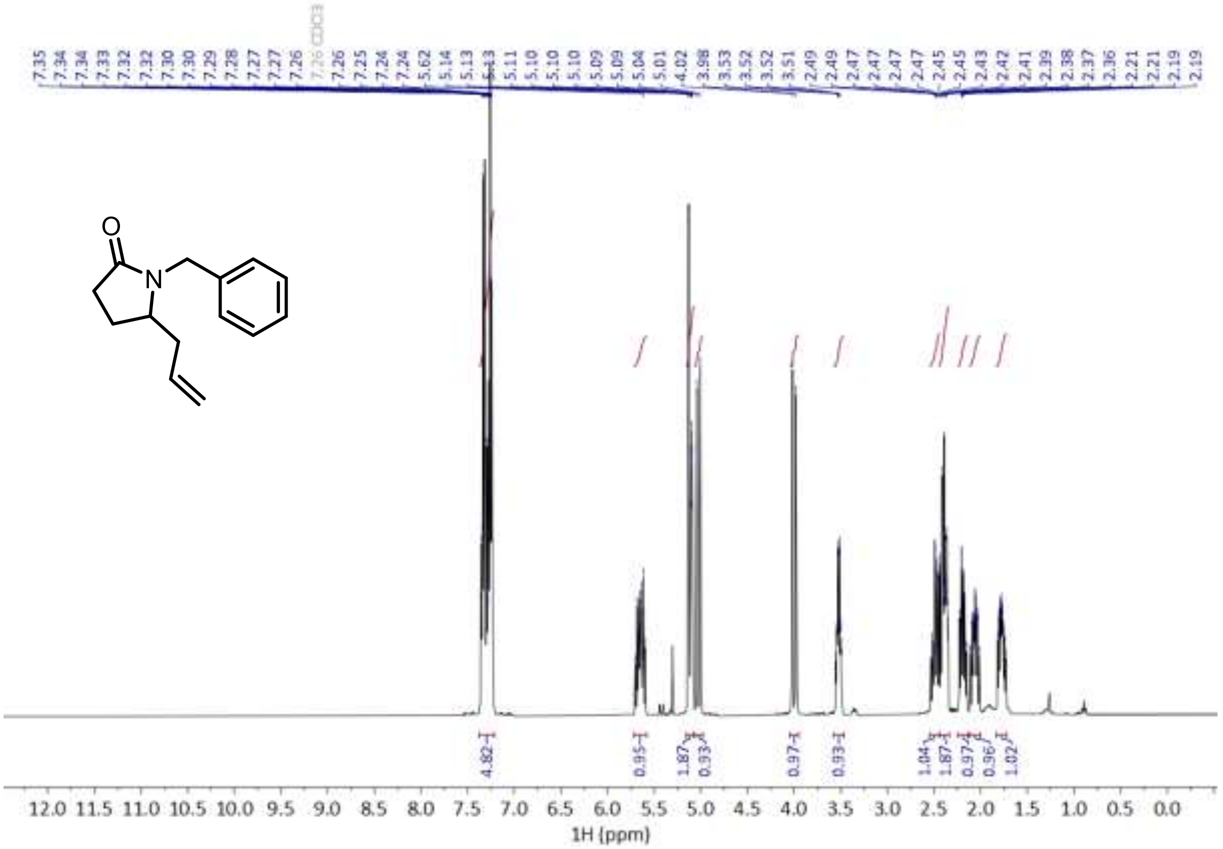

Compound 3ab (^{13}C)

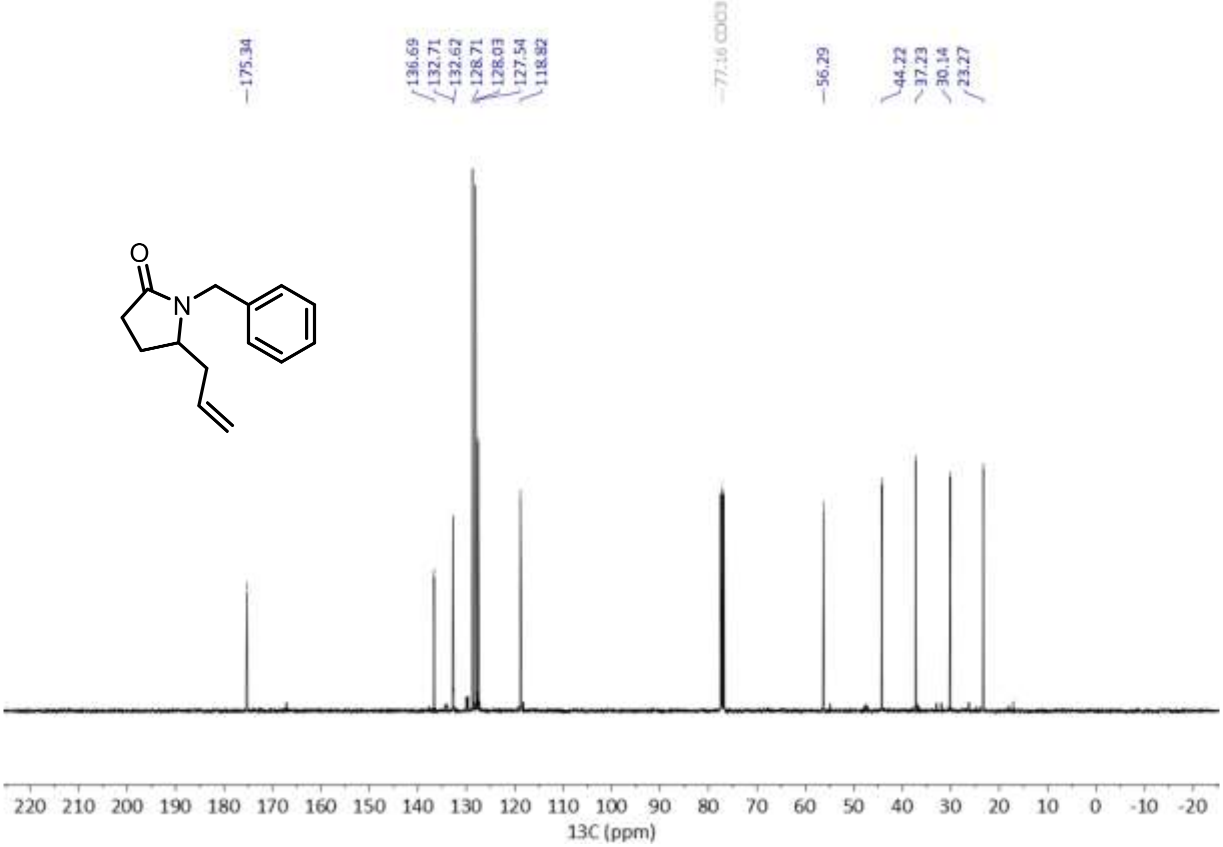

Compound 3ac (¹H)

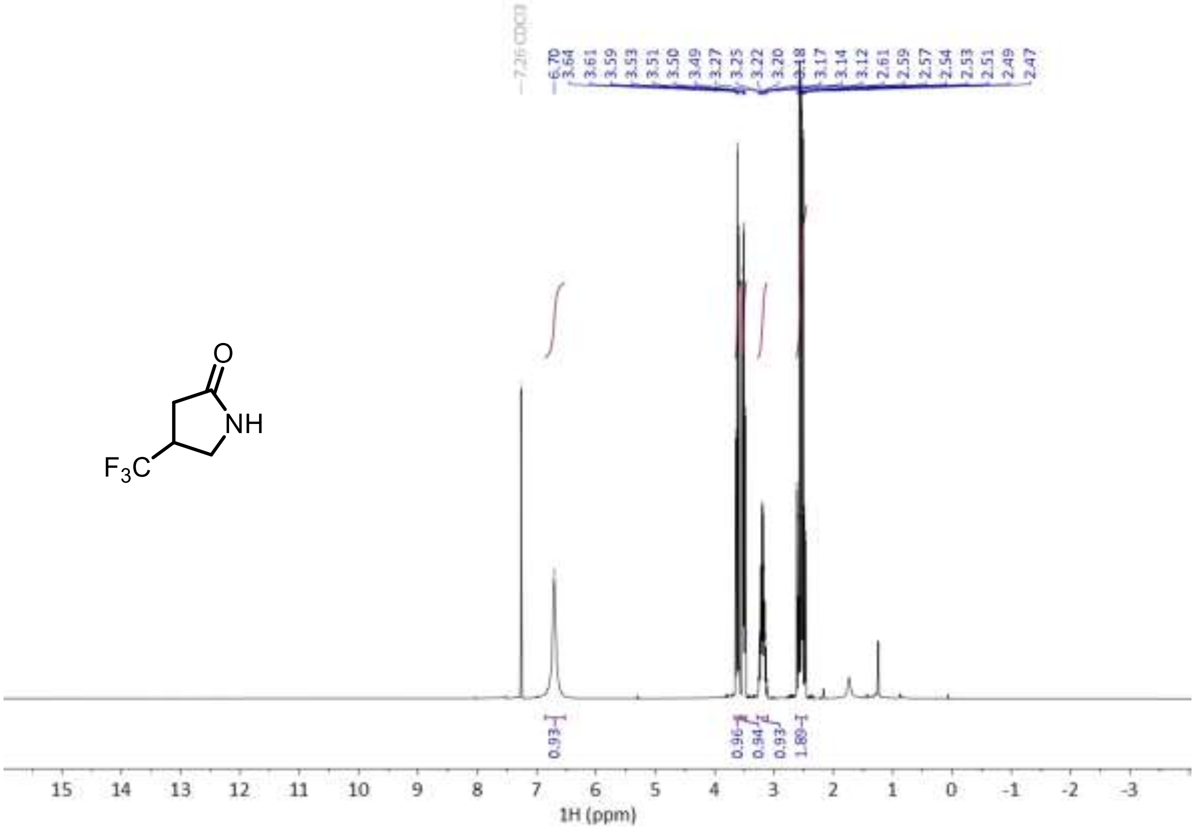

Compound 3ac (¹³C)

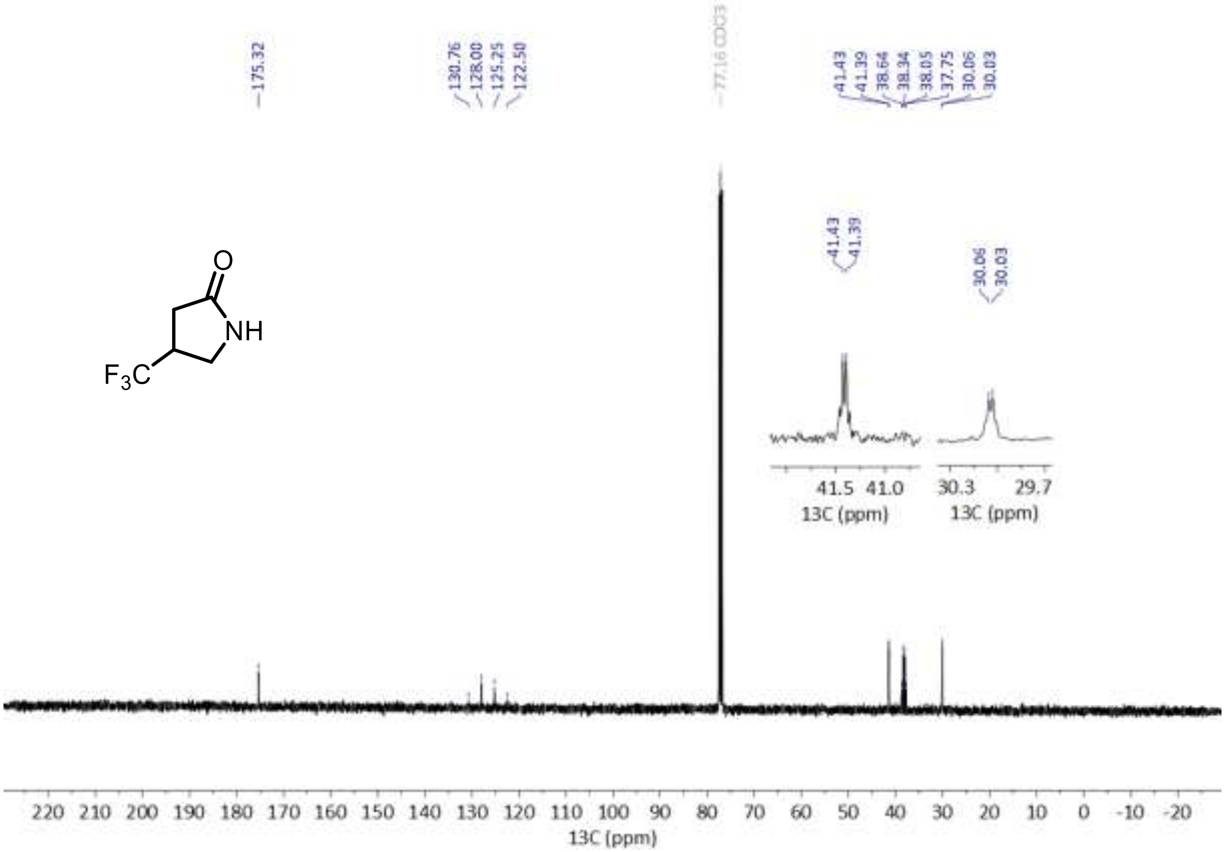

Compound 3ac (^{19}F)

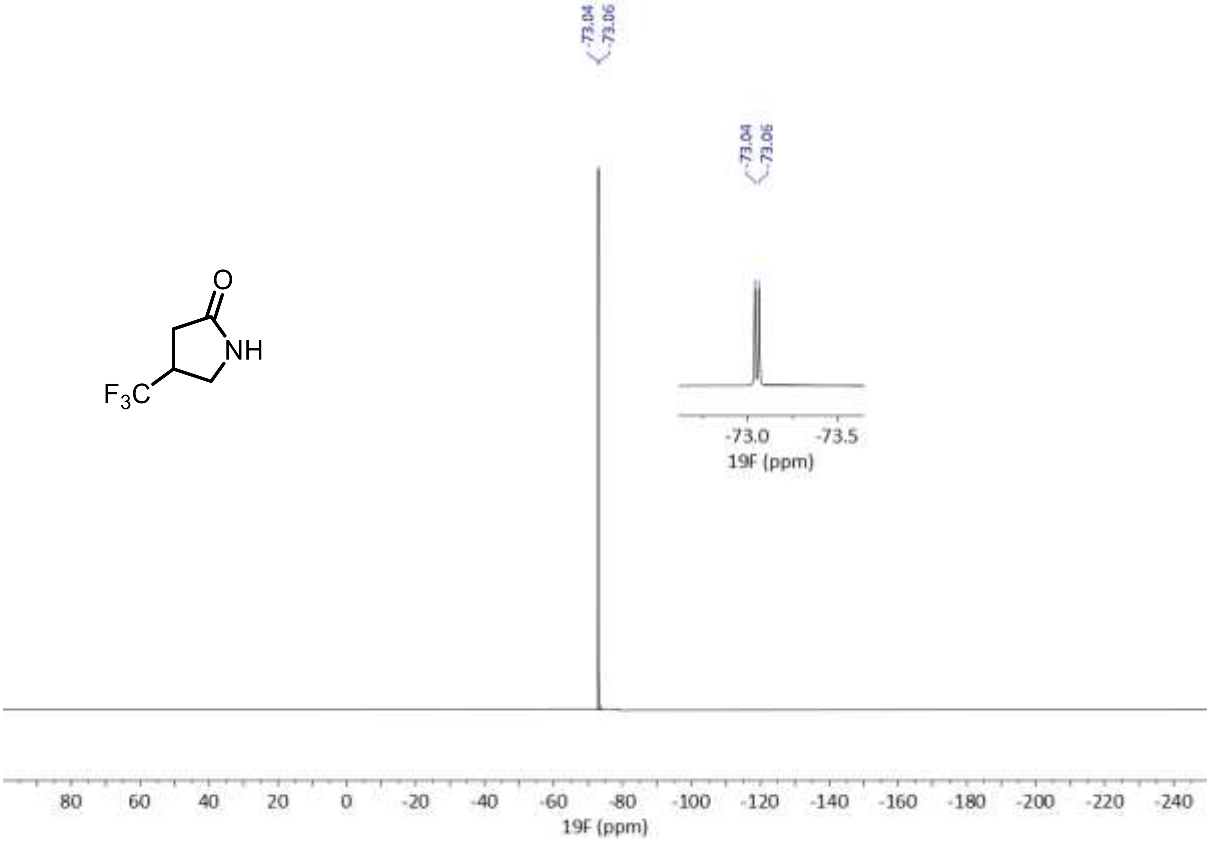

Compound 3ad (^{1}H)

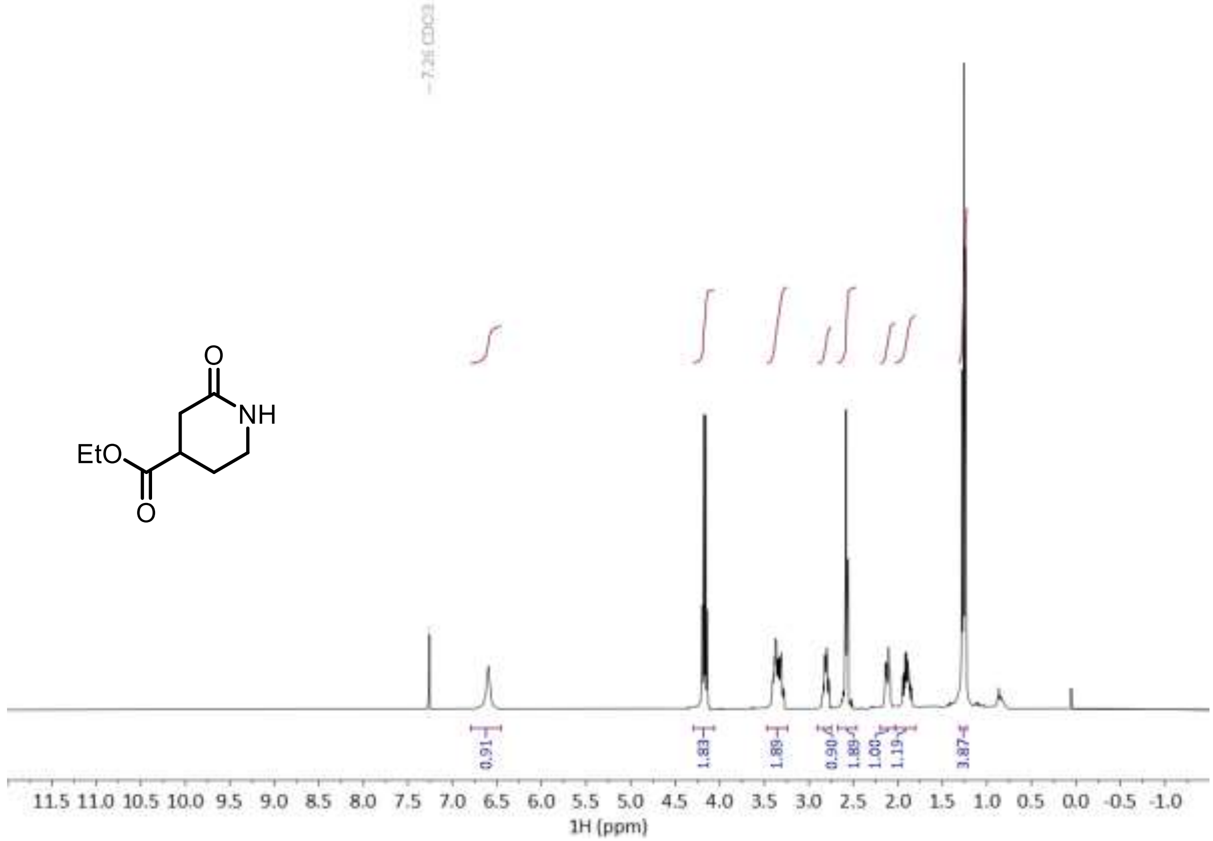

Compound 3ad (^{13}C)

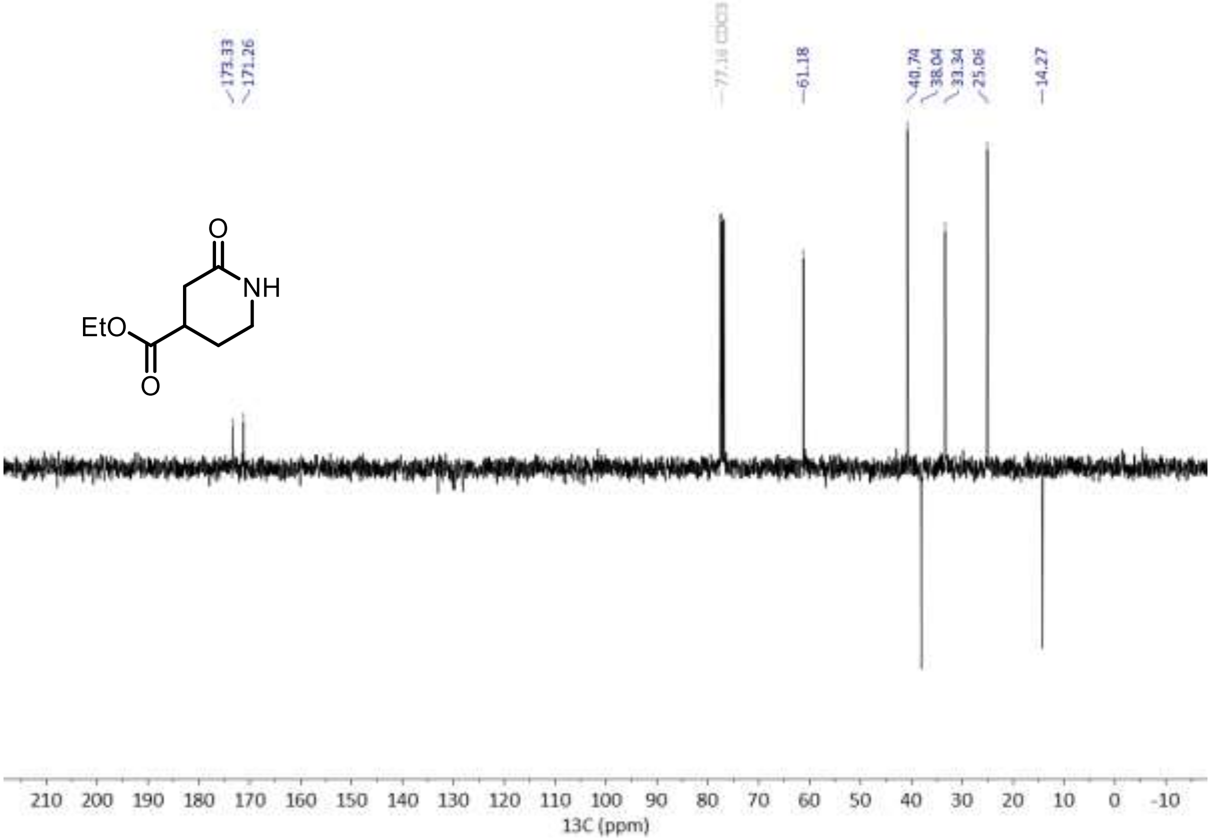

Compound 3ae (^{1}H)

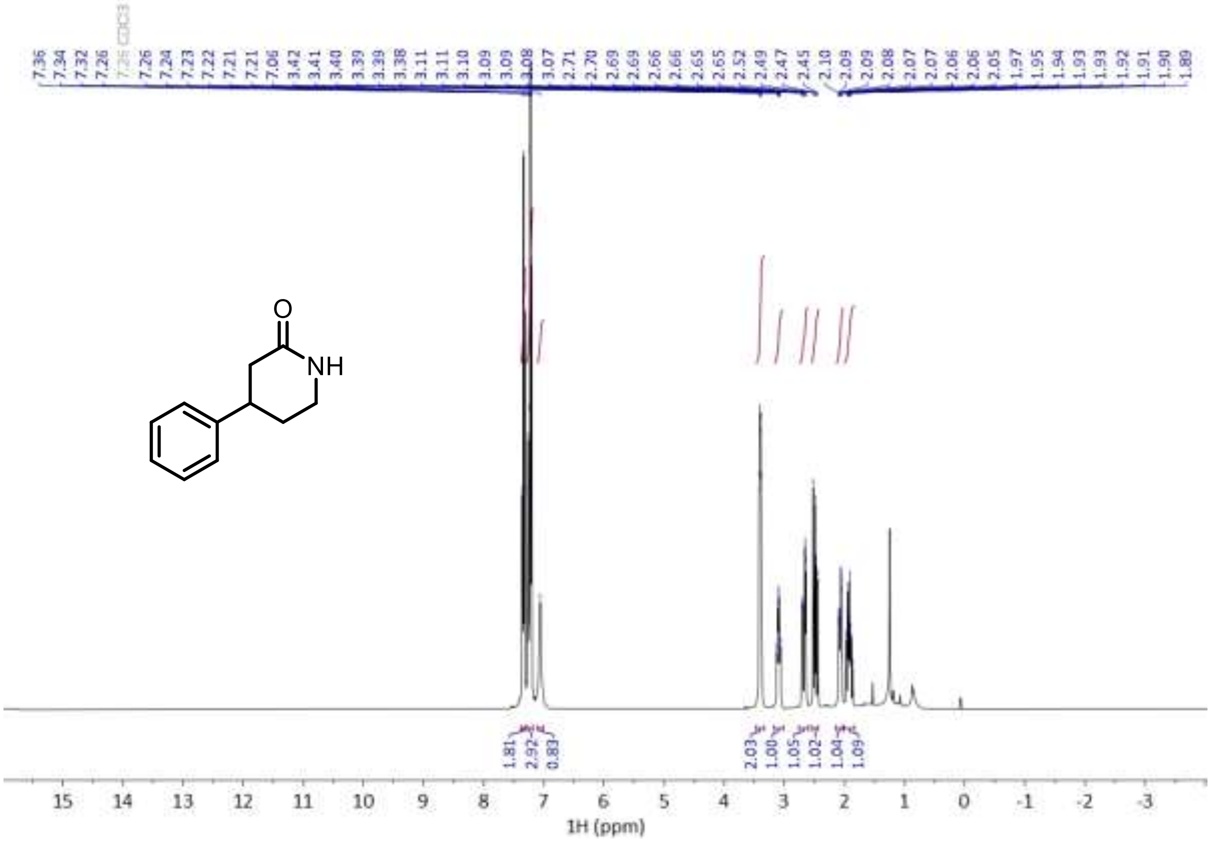

Compound 3ae (^{13}C)

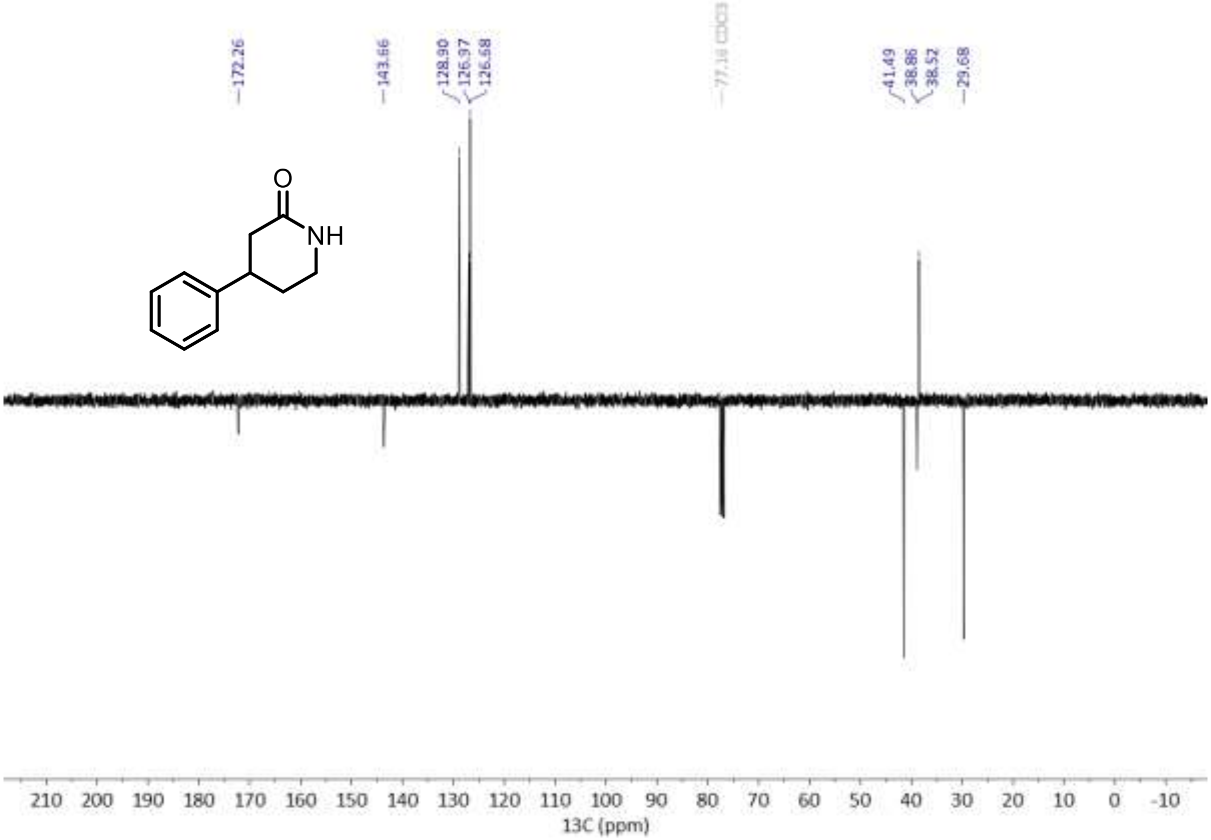

Compound 3af (^{1}H)

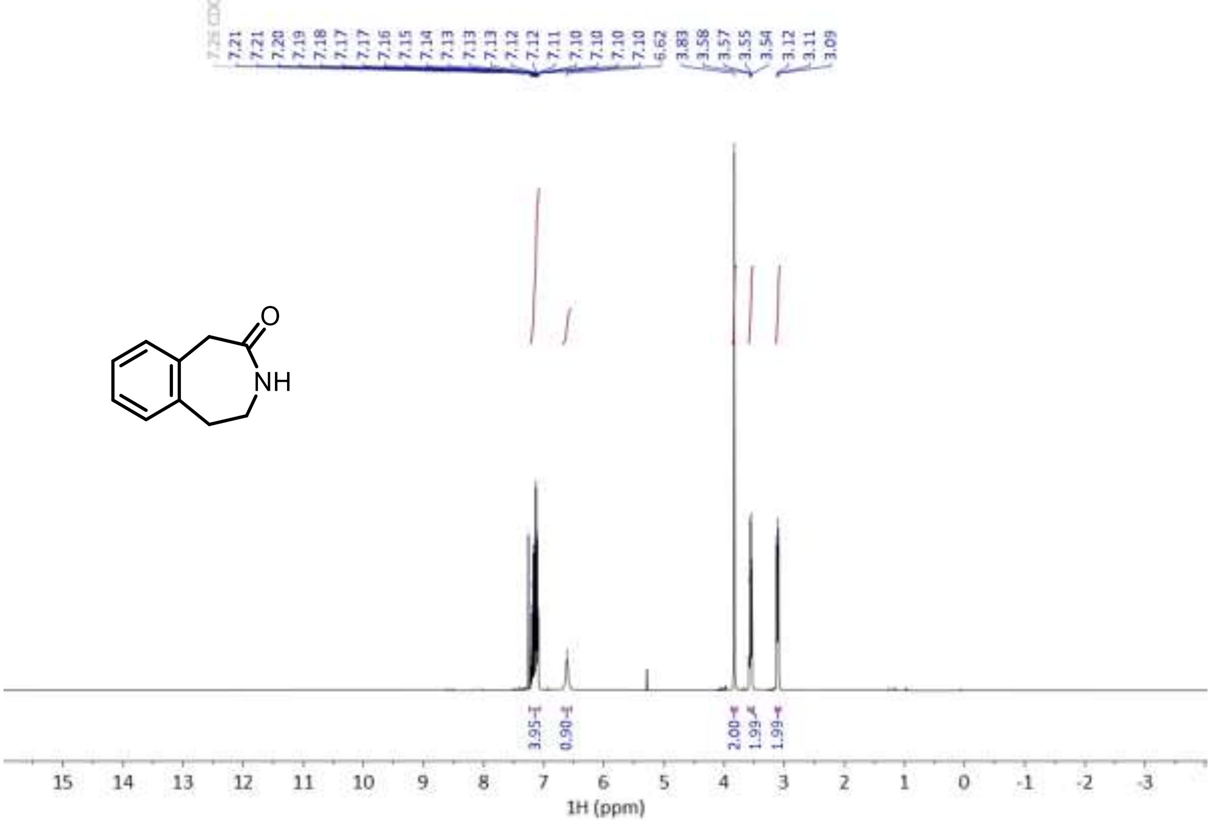

Compound 3af (^{13}C)

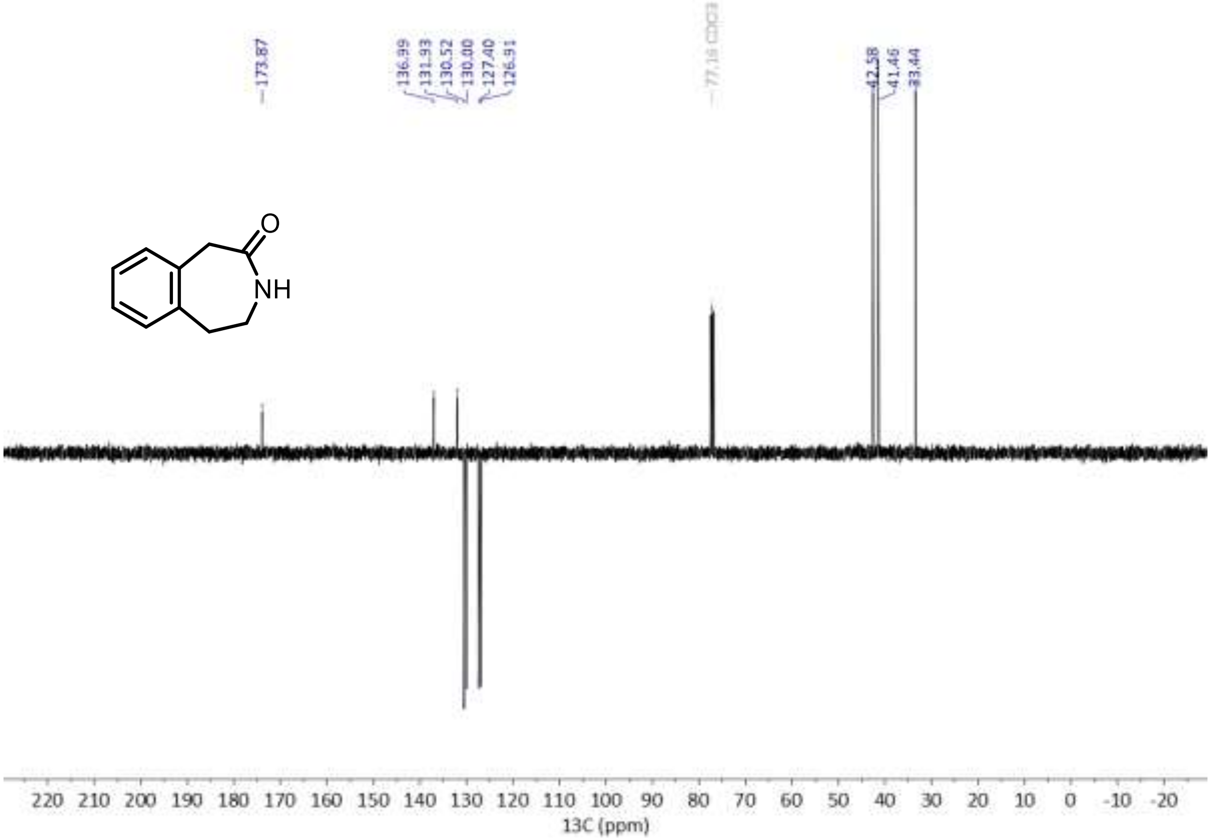

Compound 4b (^{1}H)

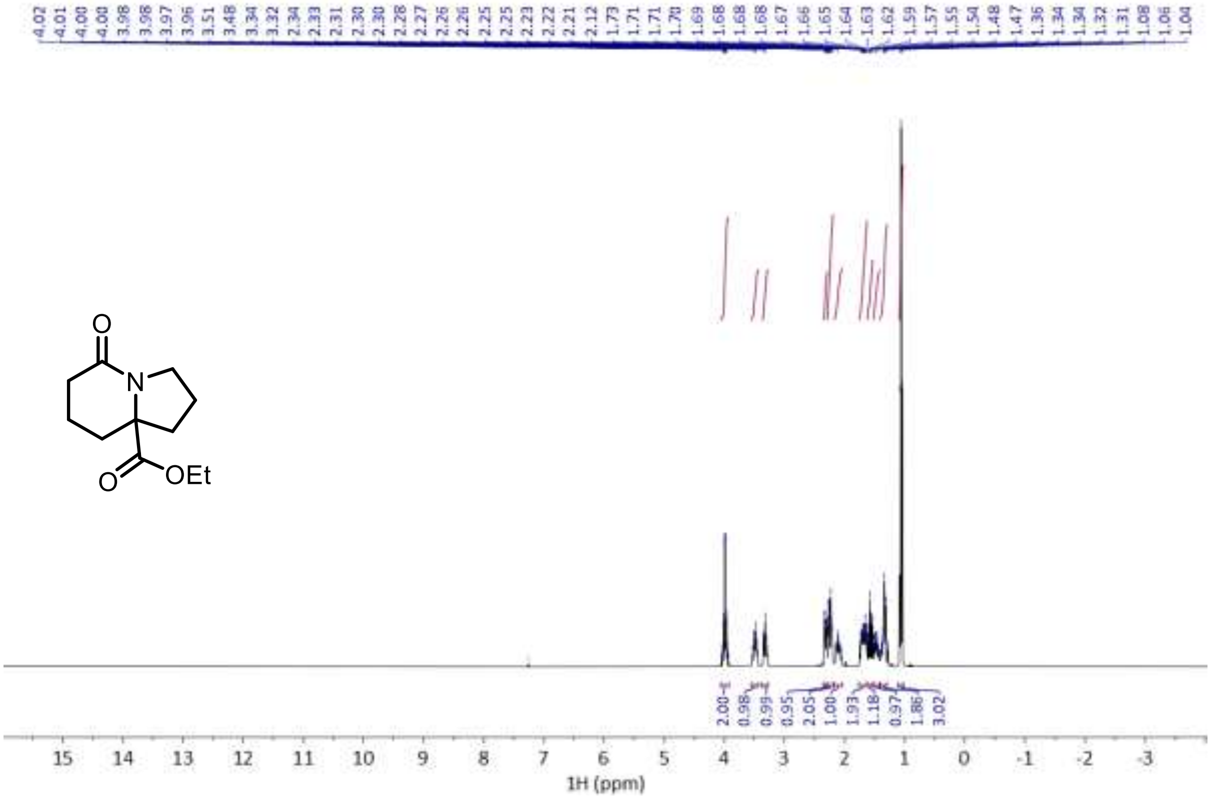

Compound 4b (^{13}C)

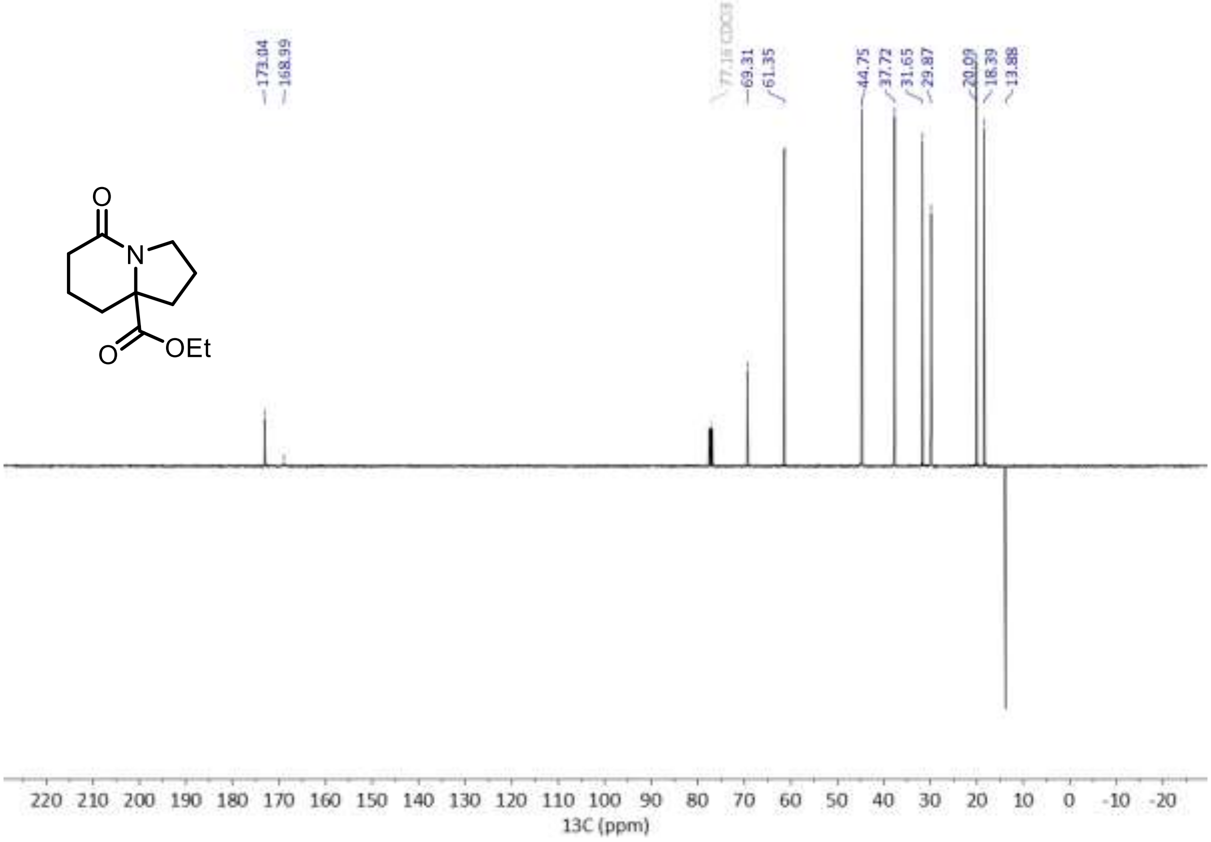

Compound 4c (^{1}H)

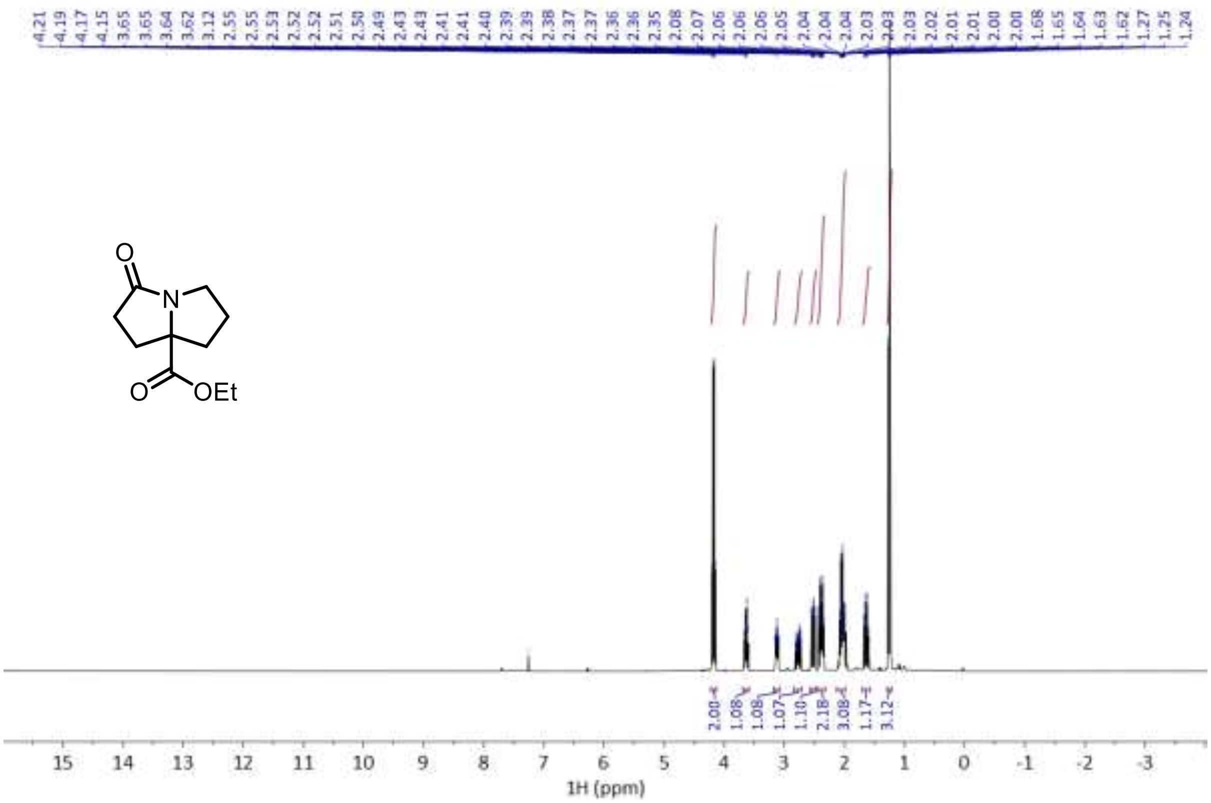

Compound 4c (^{13}C)

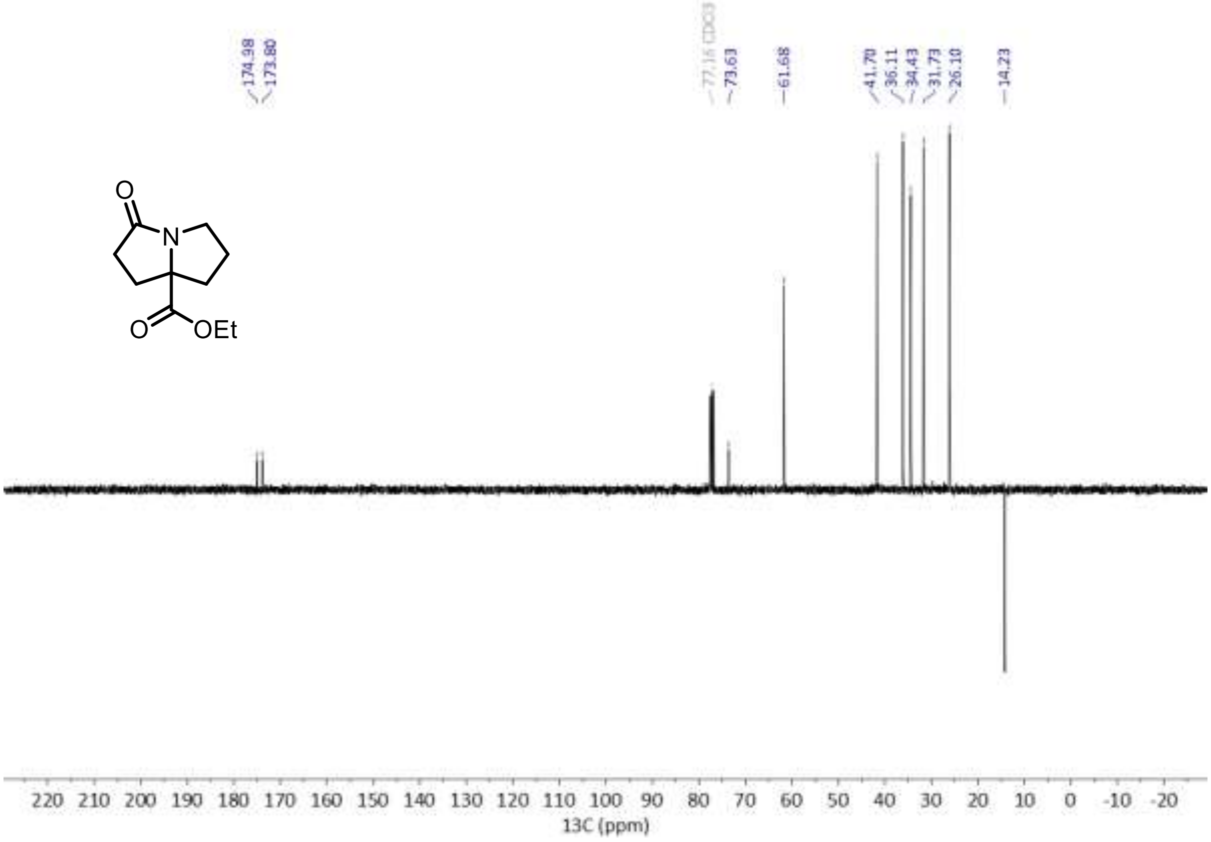

Compound 4e (^1H)

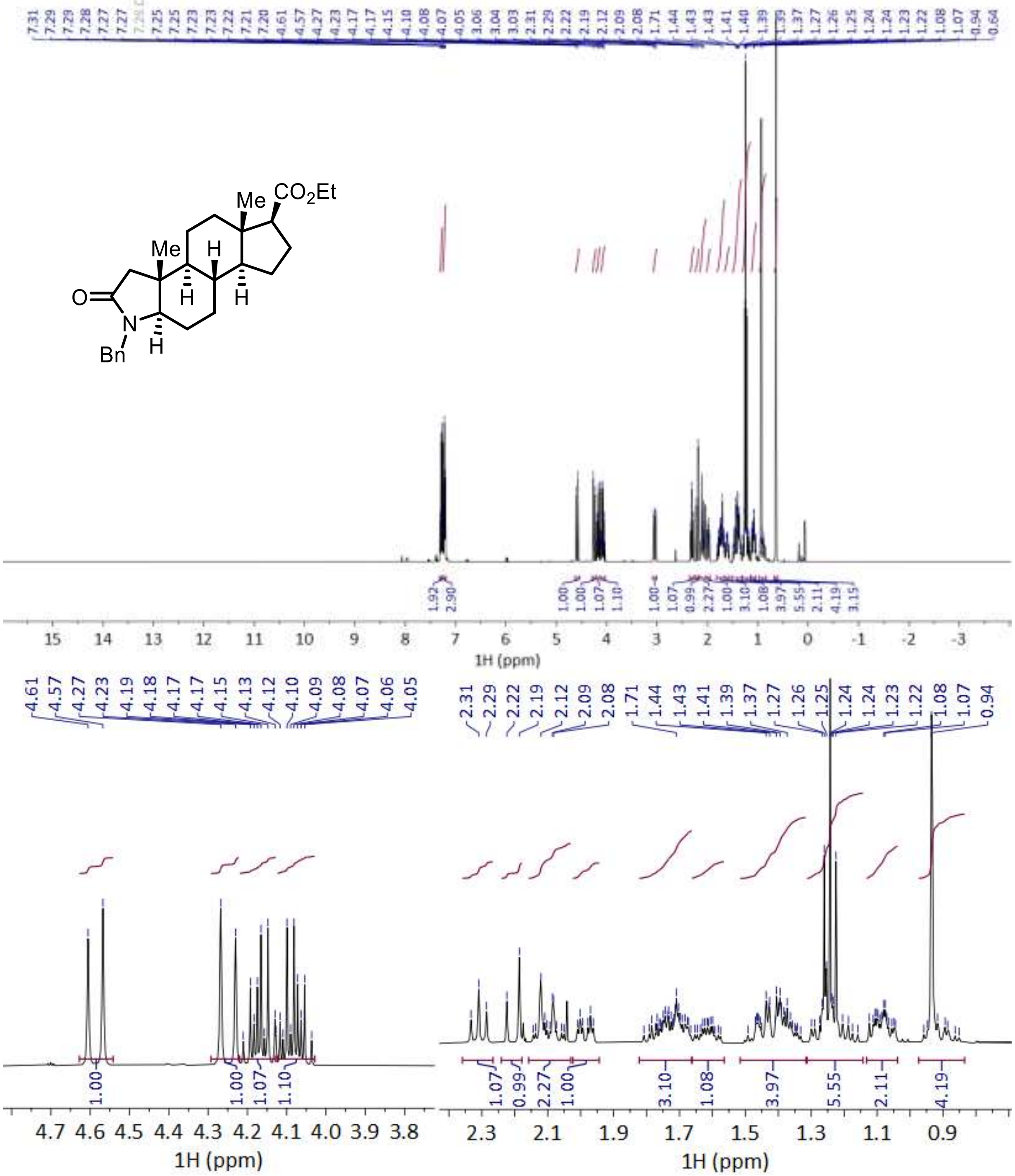

Compound 4e (^{13}C)

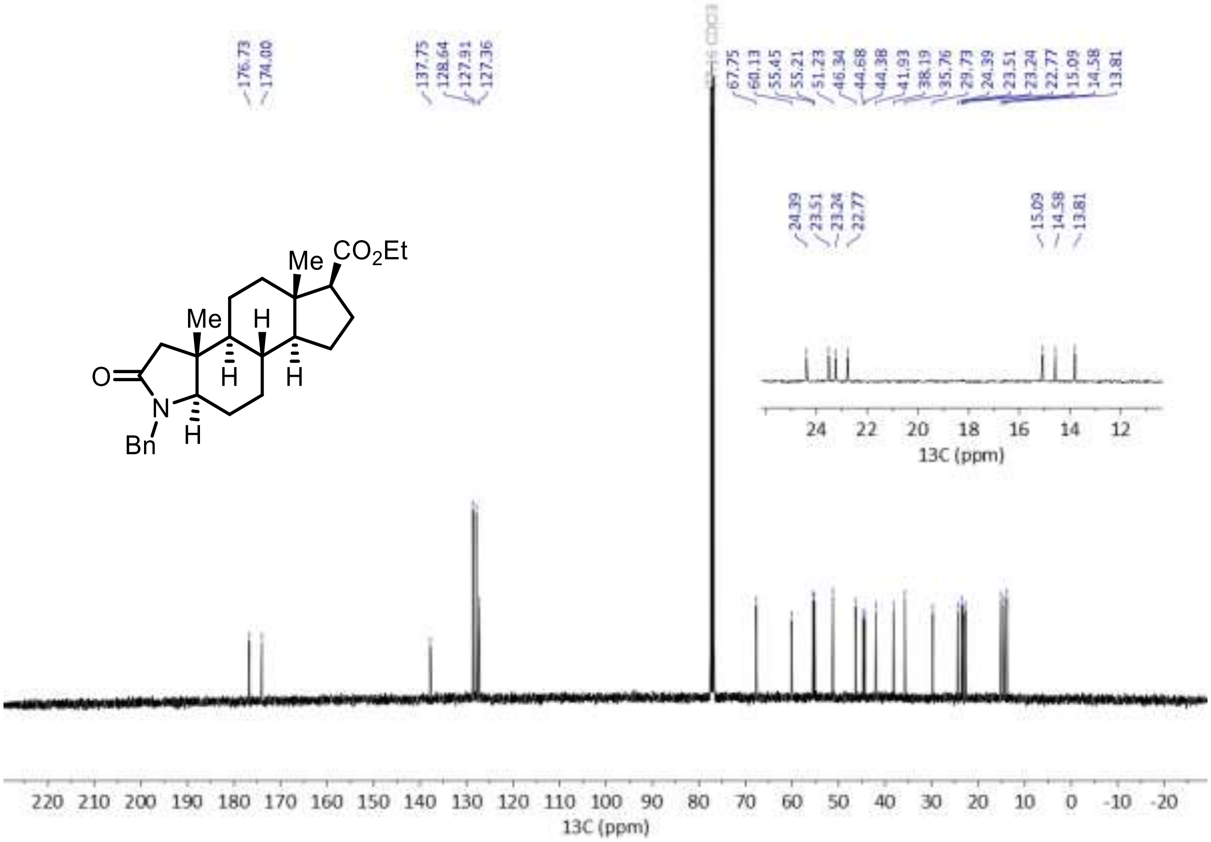

Compound 4g (^{1}H)

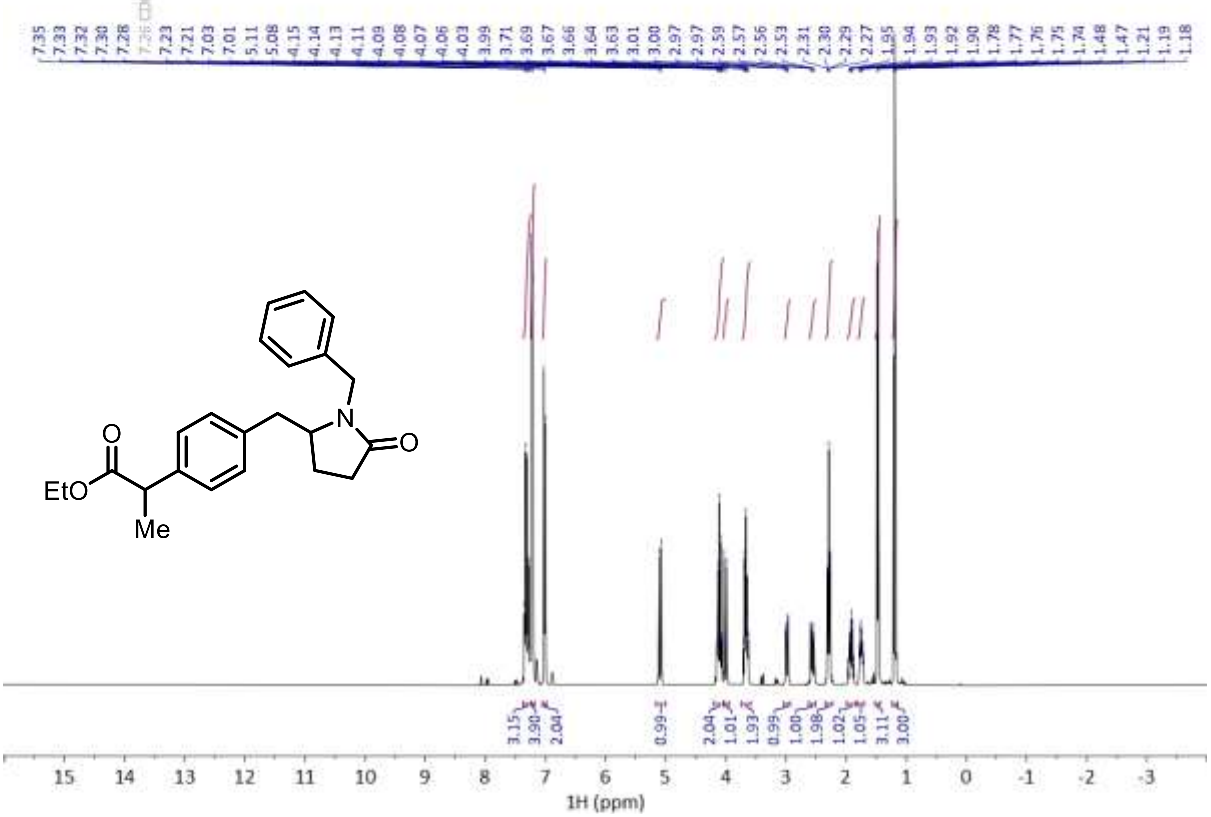

Compound 4g (^{13}C)

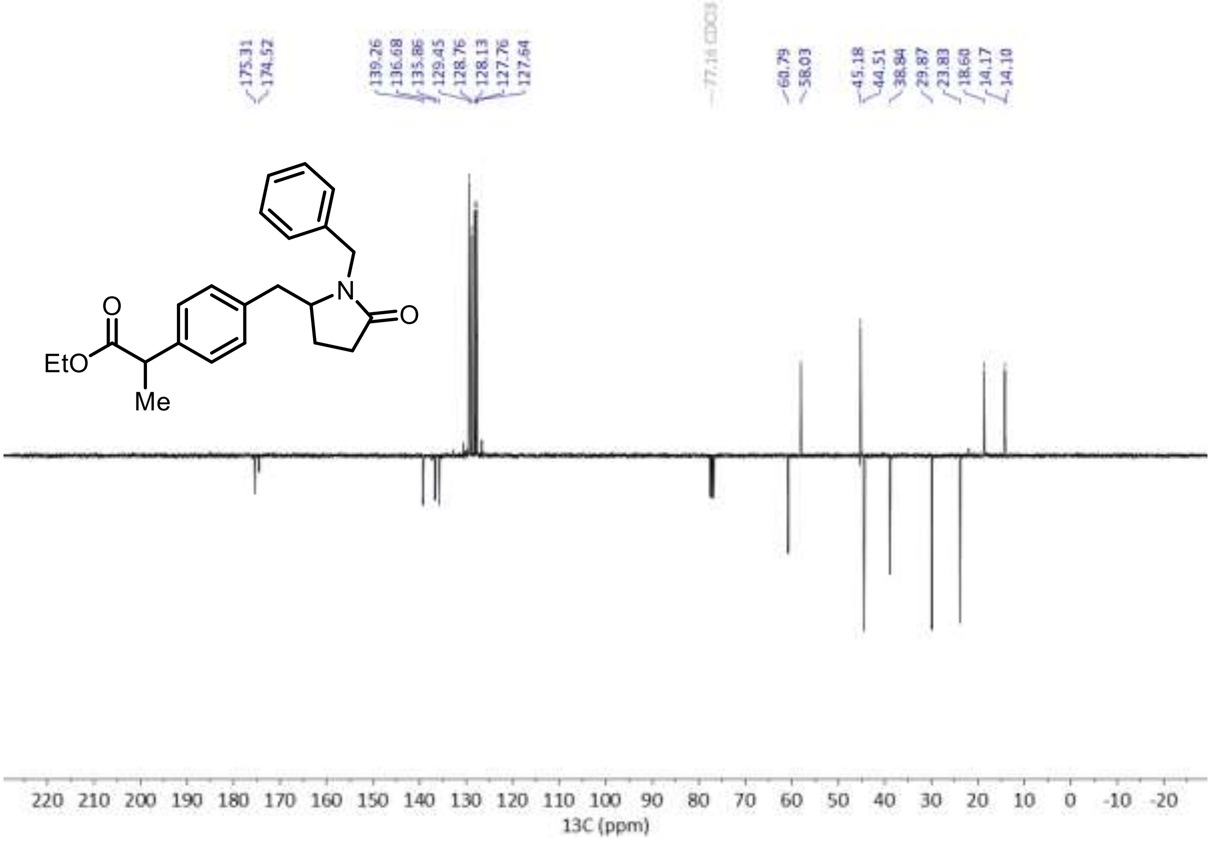

Compound 4j (¹H)

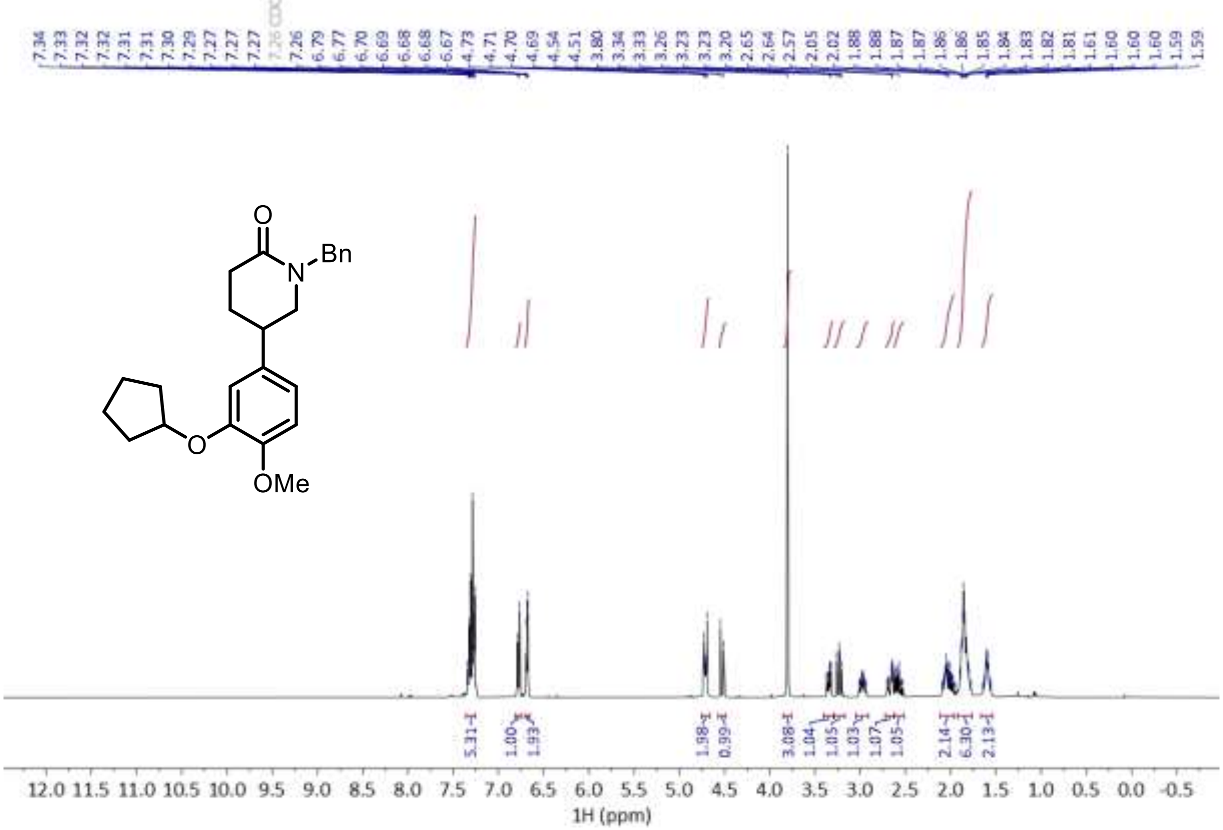

Compound 4j (¹³C)

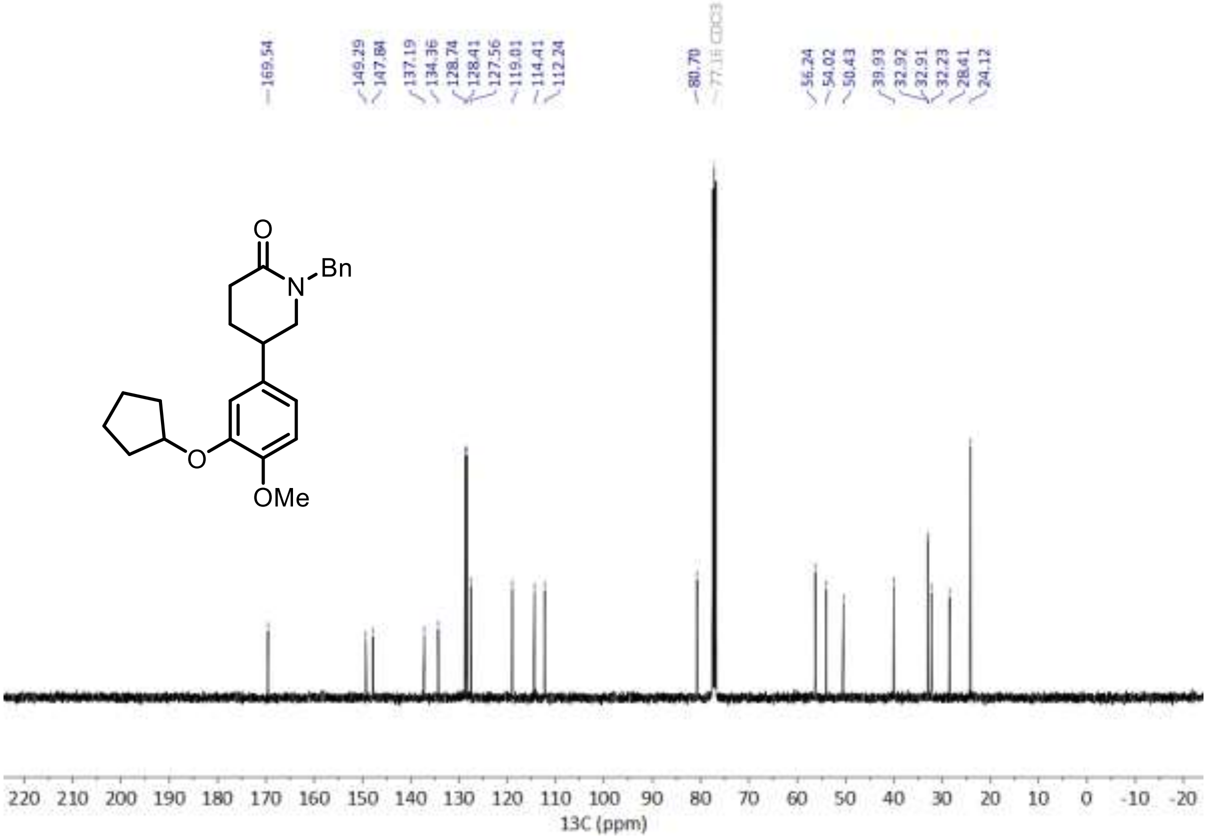

Compound 4k (^{1}H)

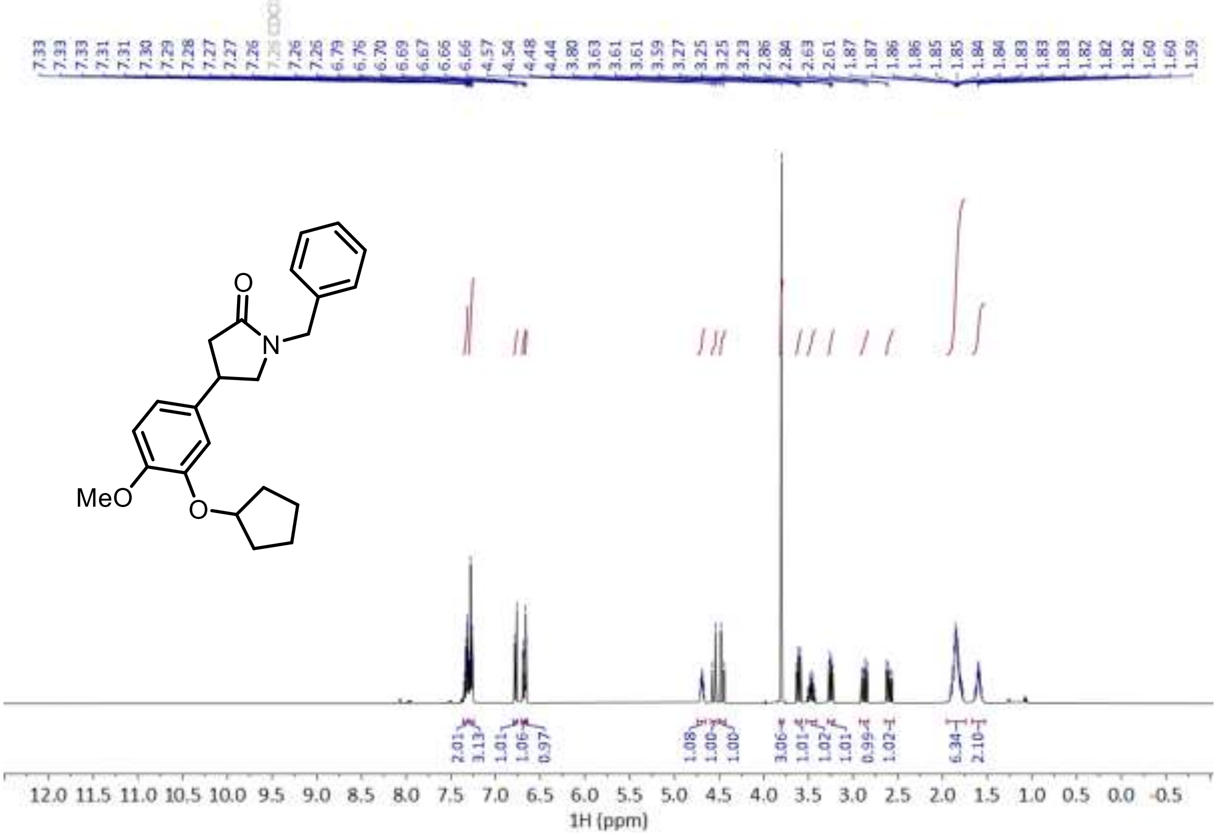

Compound 4k (^{13}C)

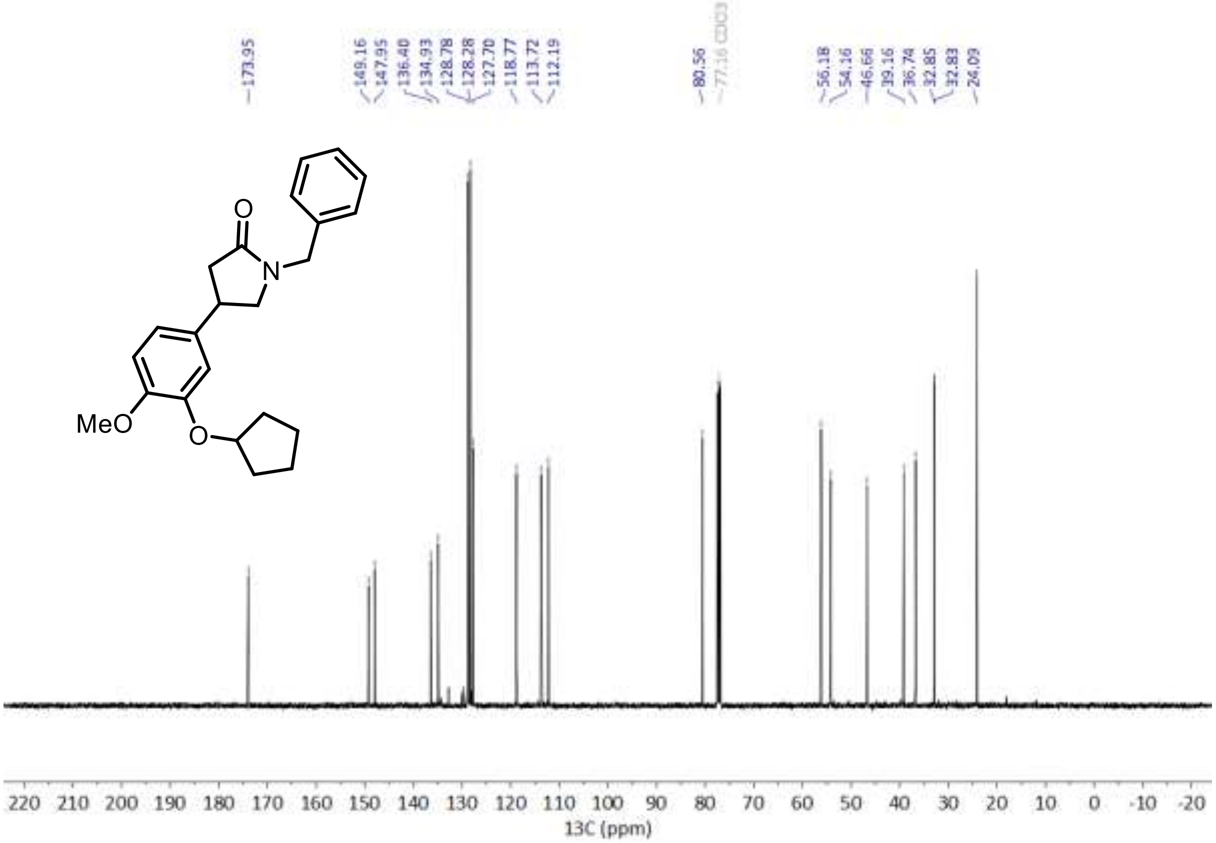

Compound 4m (^{1}H)

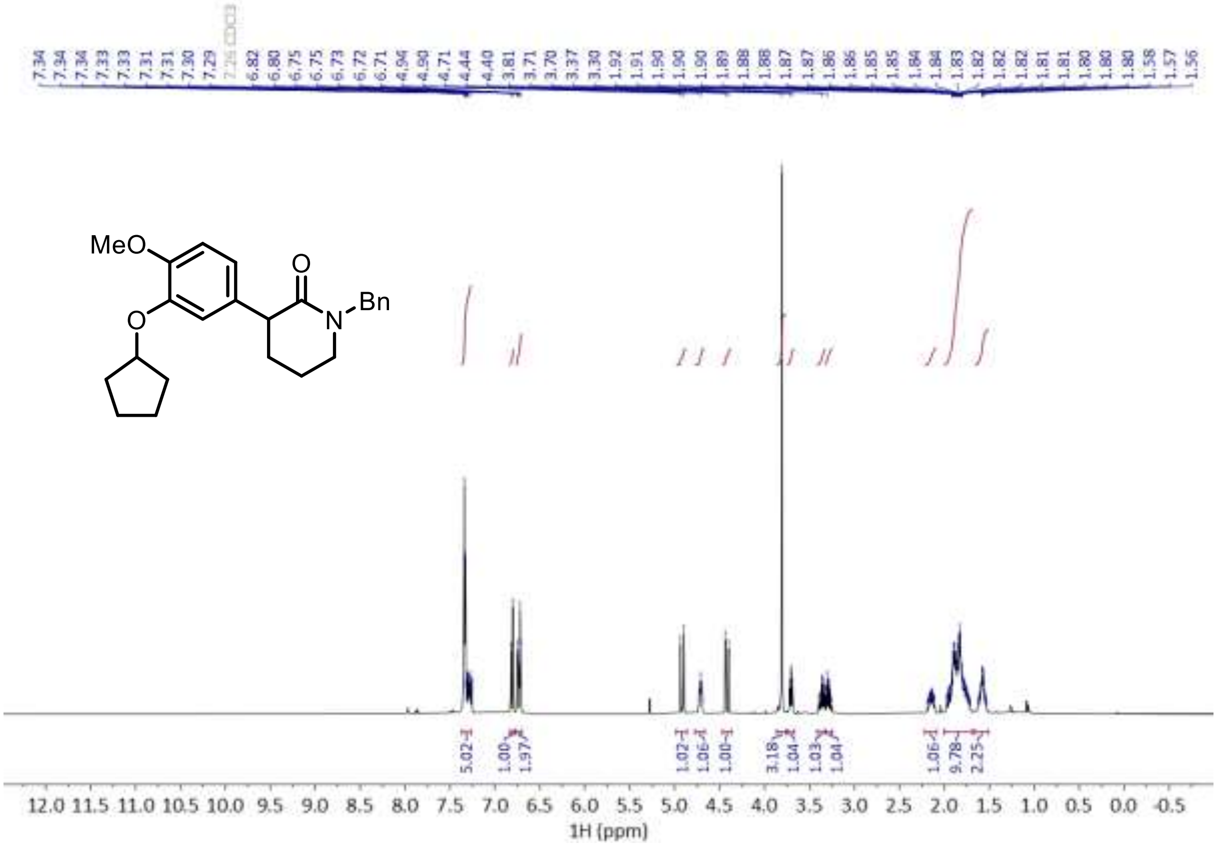

Compound 4m (^{13}C)

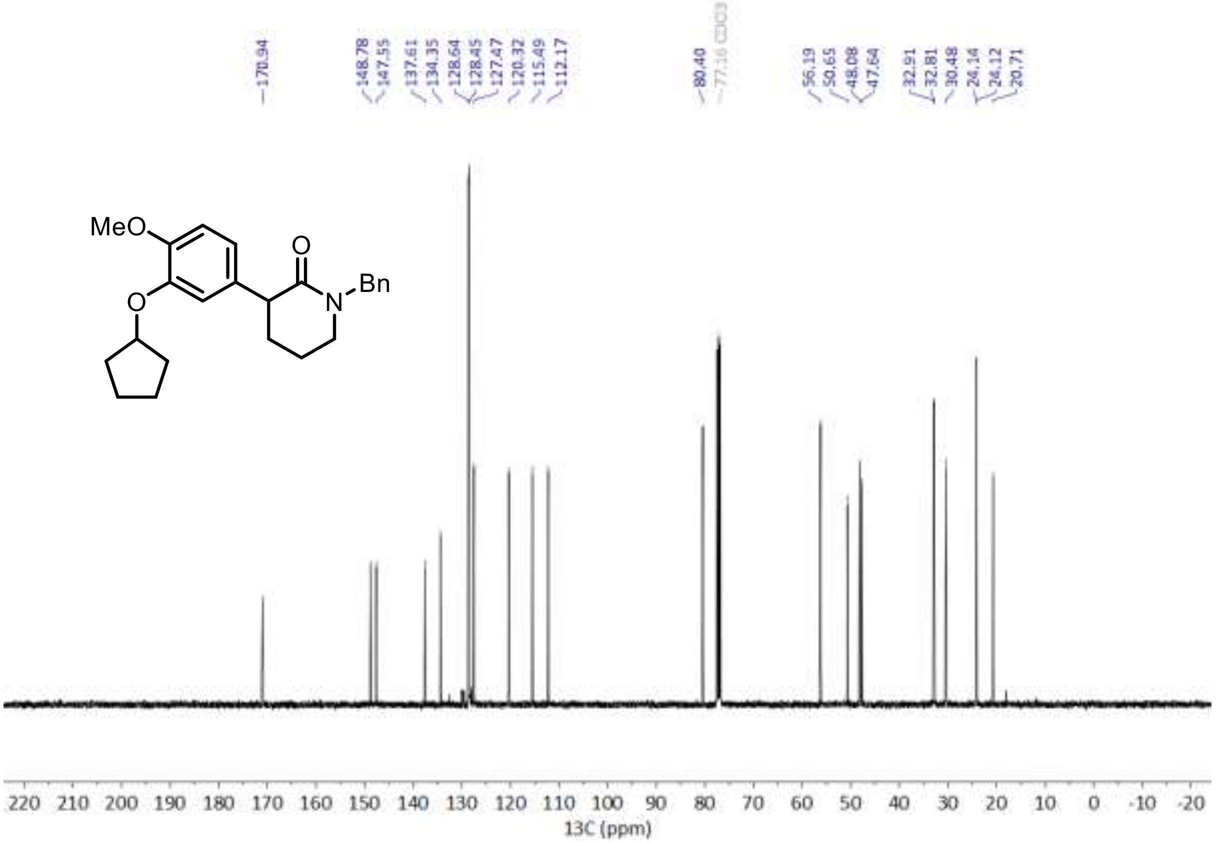

Compound 5b (^{1}H - crude)

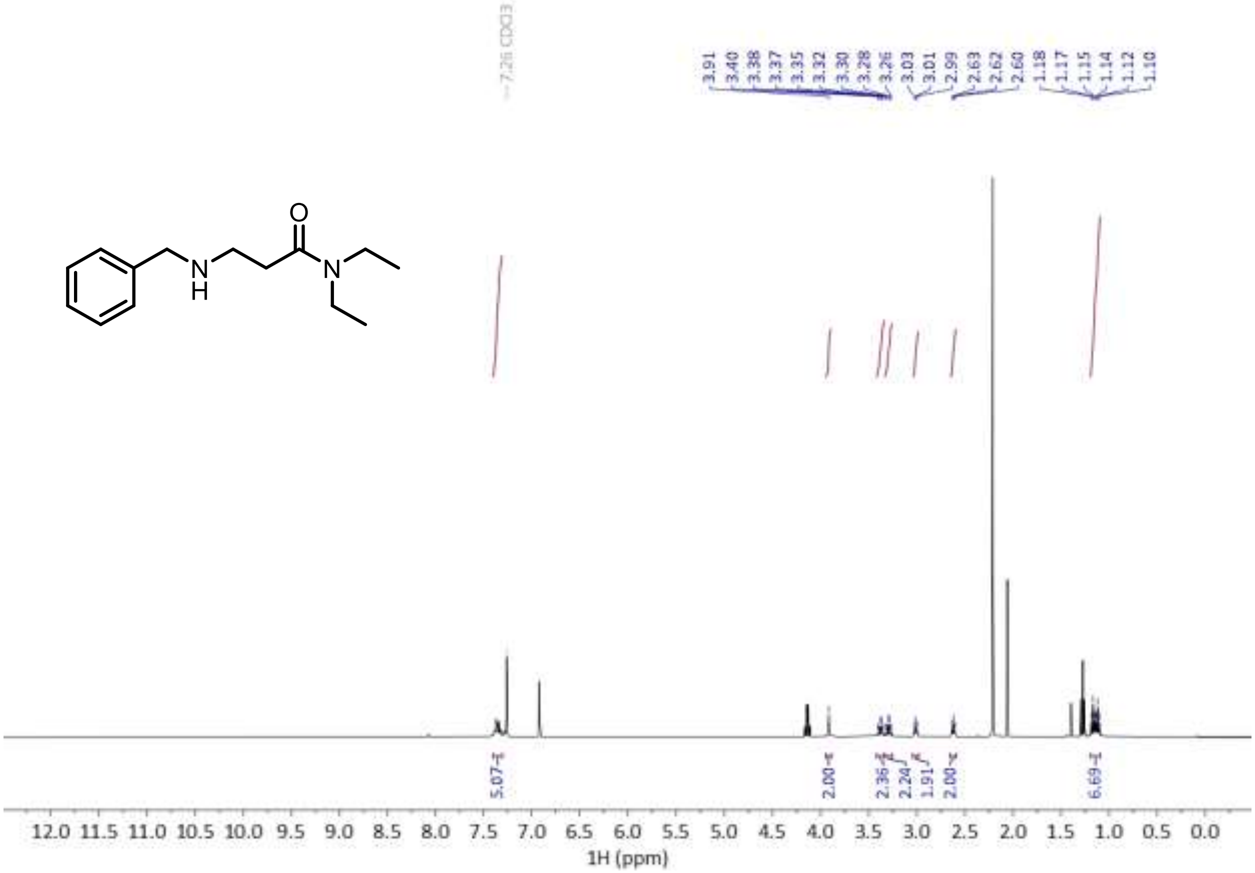

Compound 5e (^{1}H)

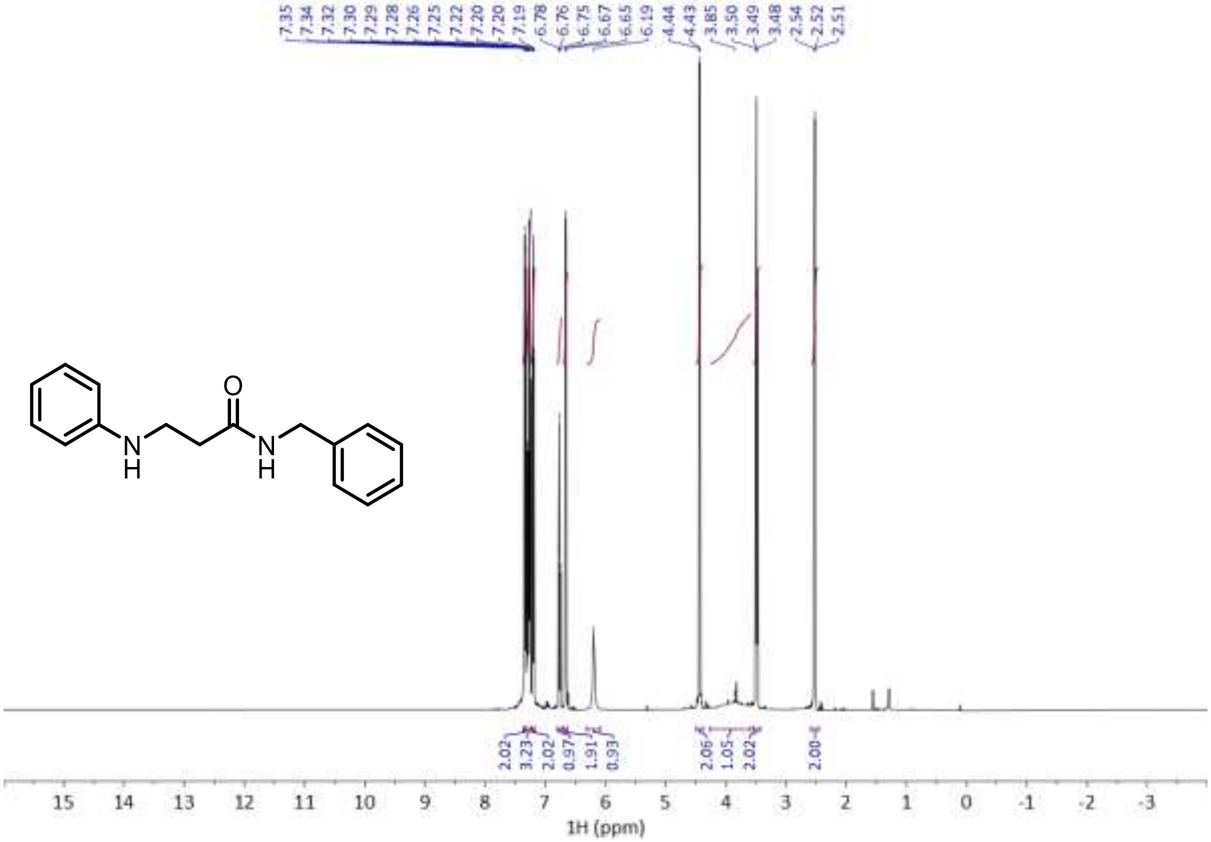

Compound 5e (^{13}C)

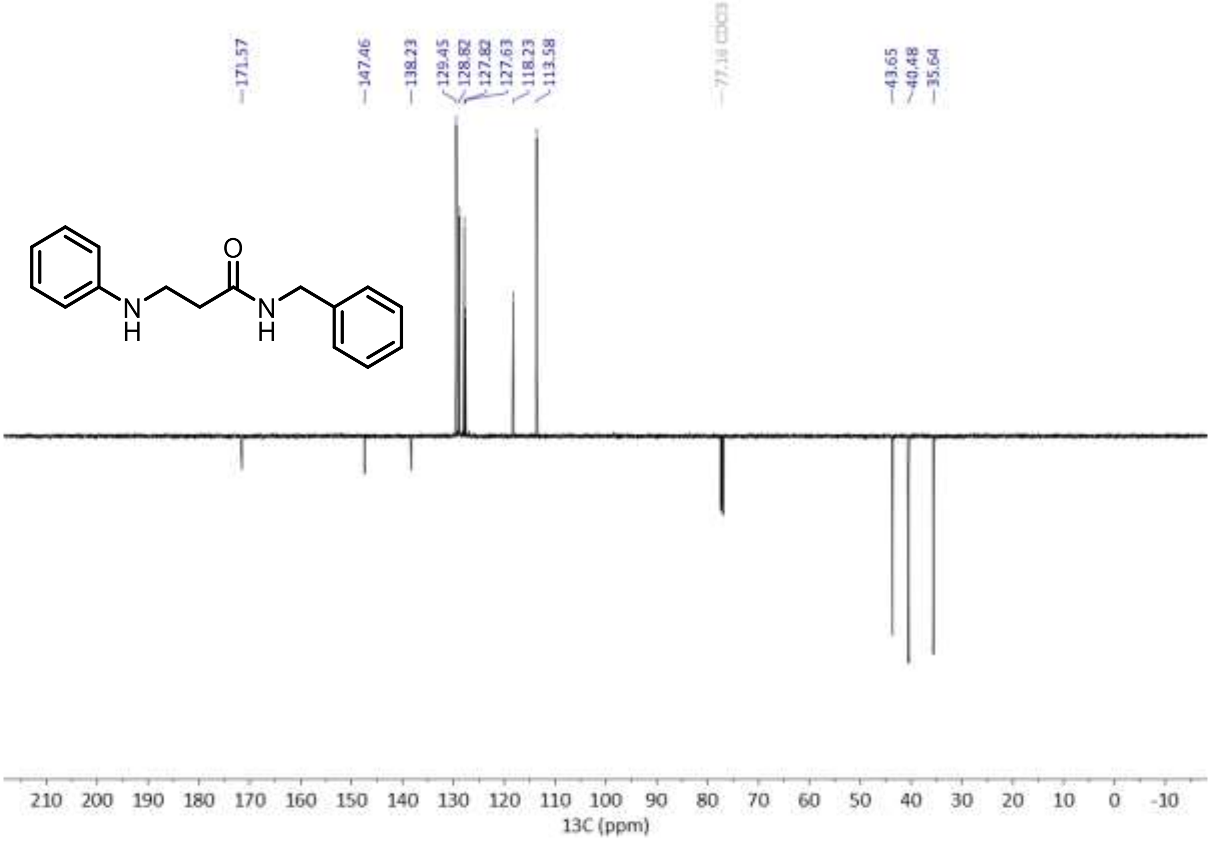

Compound 5f (^{1}H)

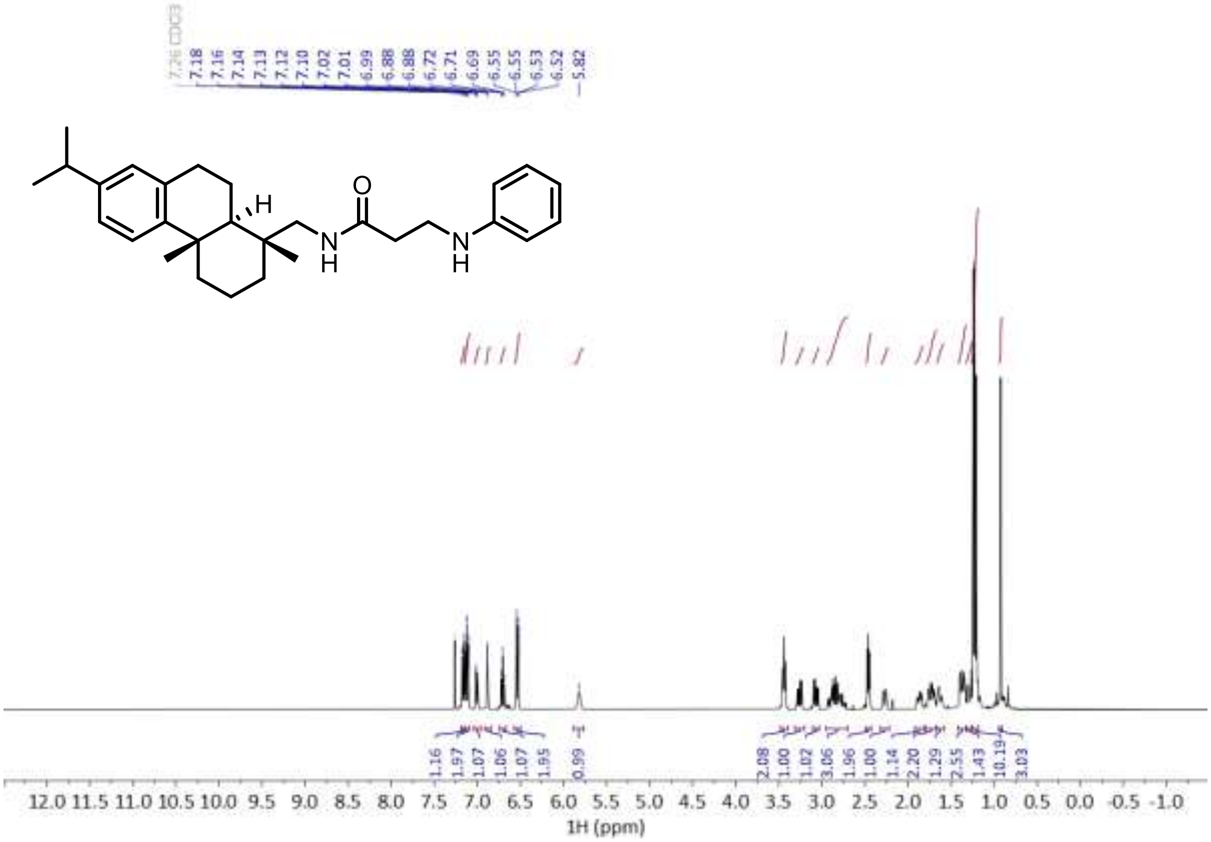

Compound 5f (^{13}C)

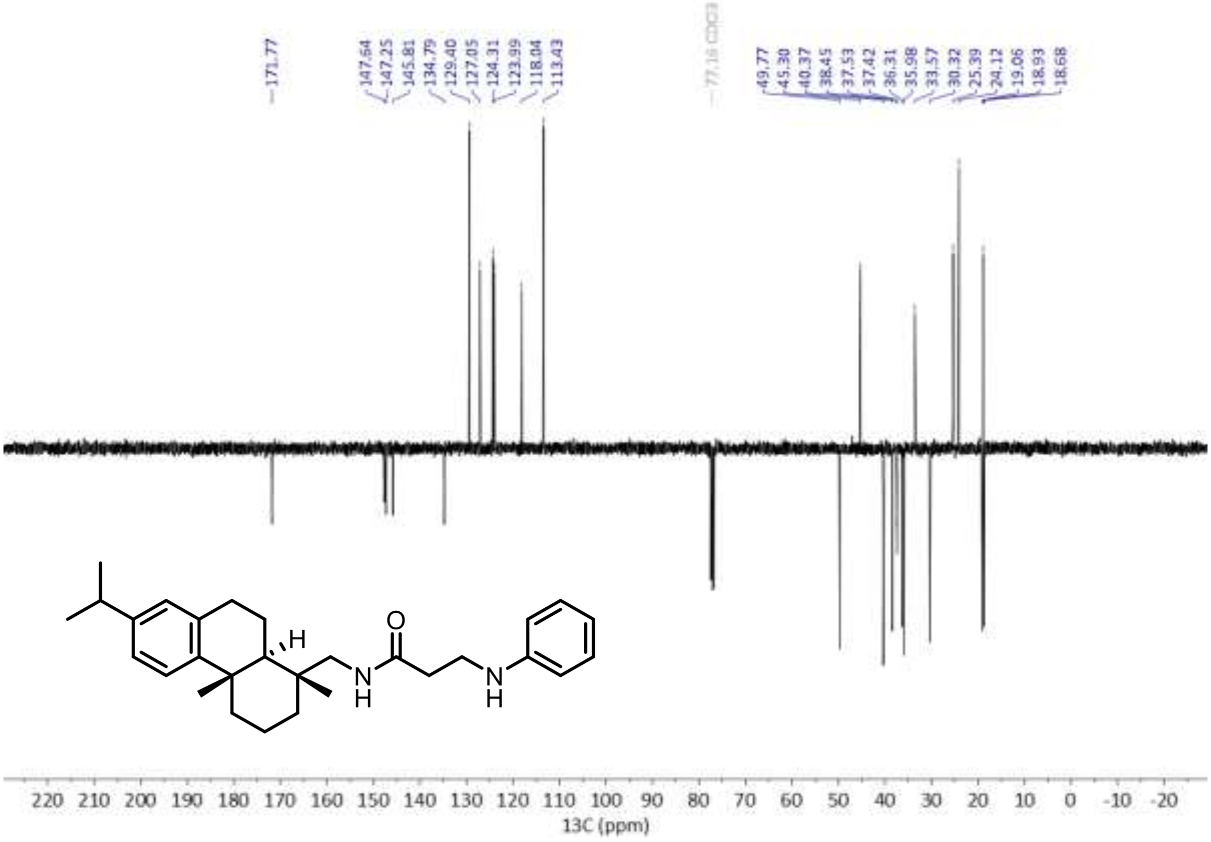

Compound 5g (^{1}H)

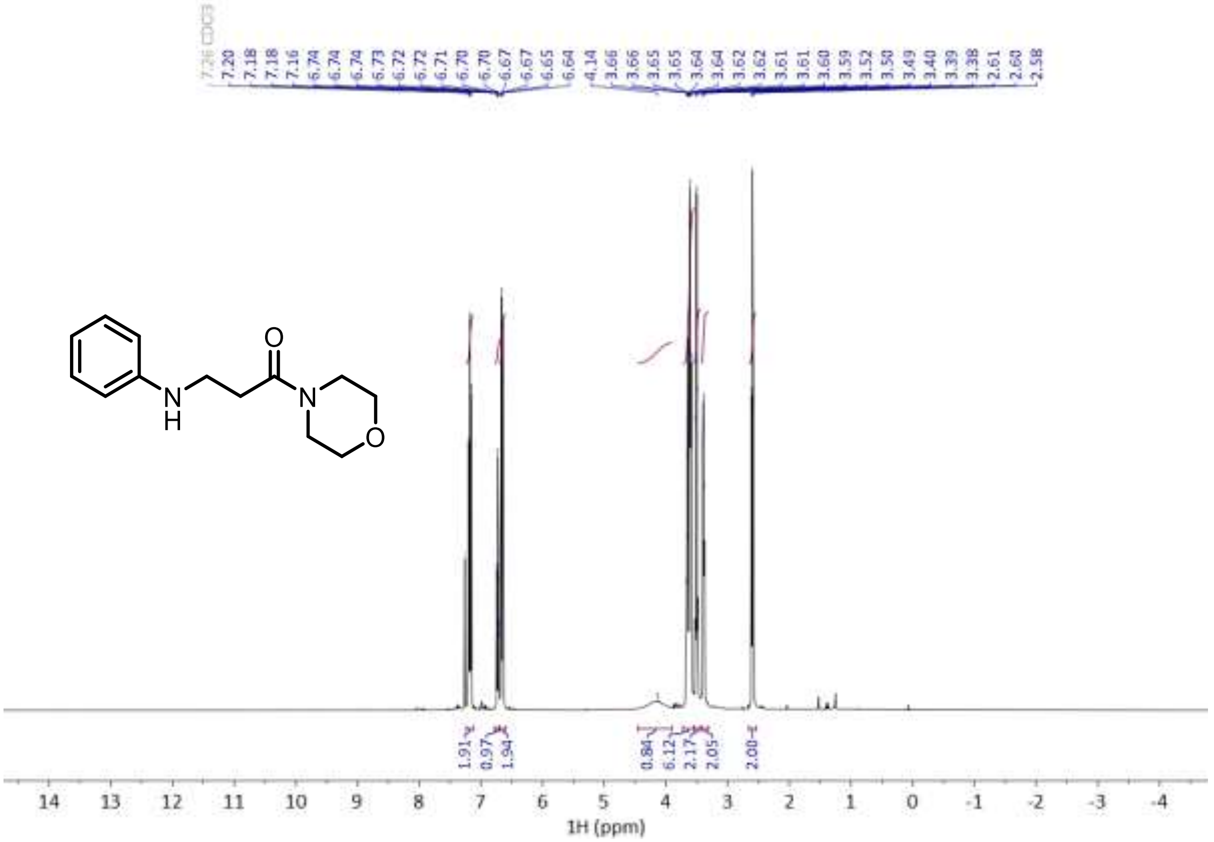

Compound 5g (^{13}C)

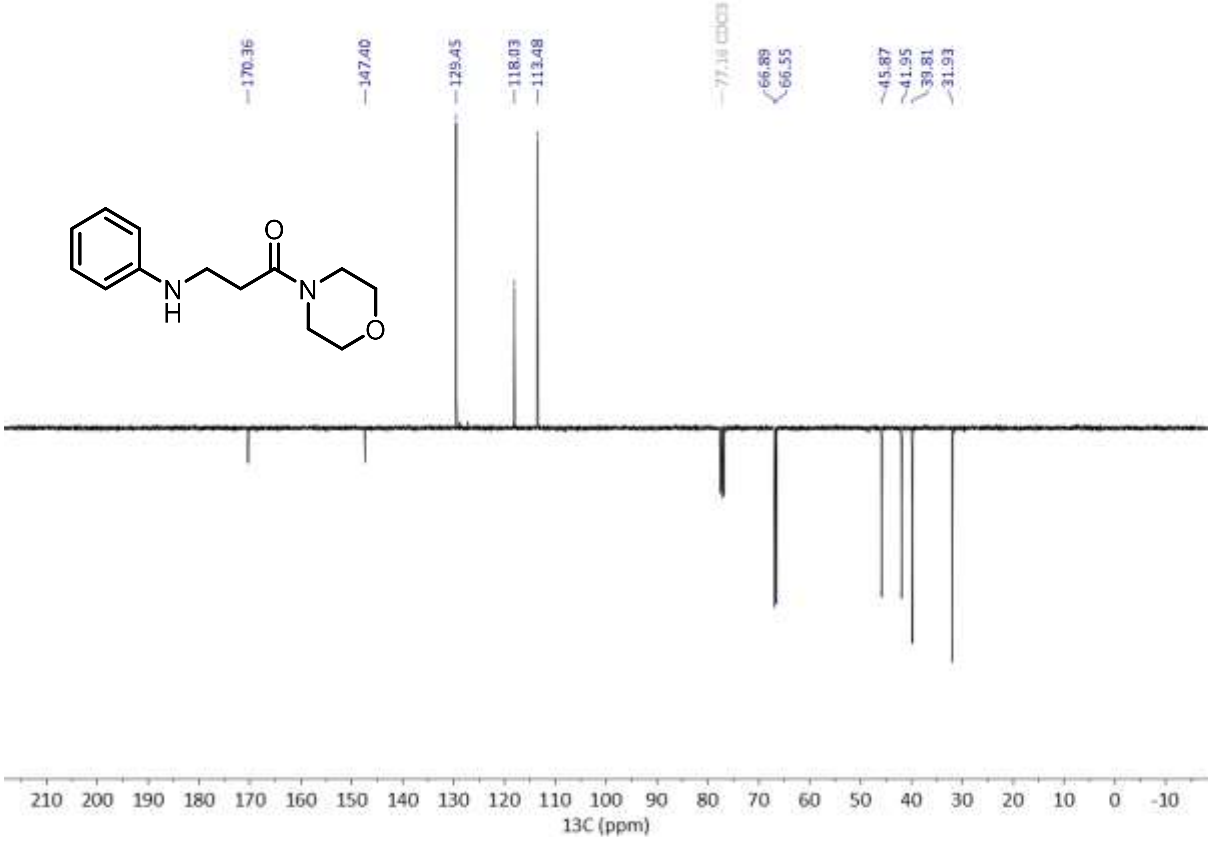

Compound 5h (^{1}H)

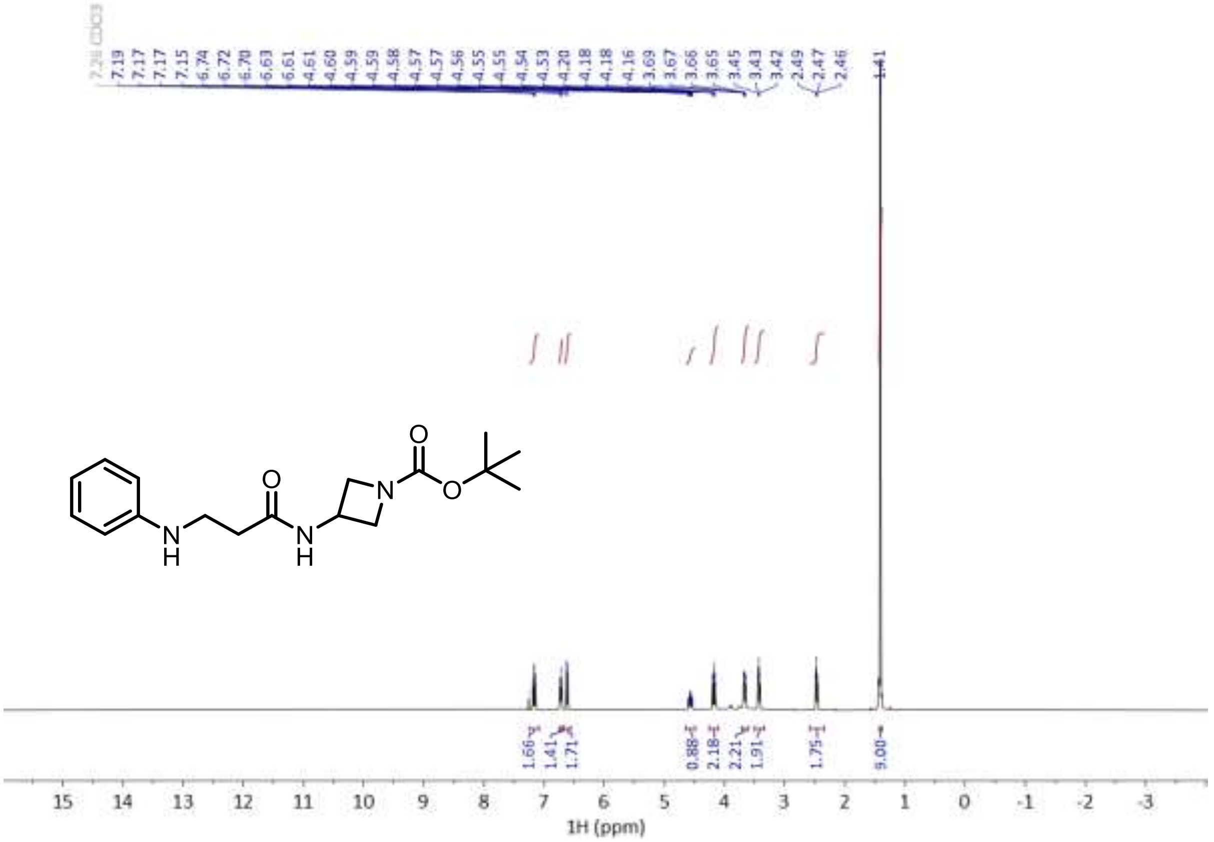

Compound 5h (^{13}C)

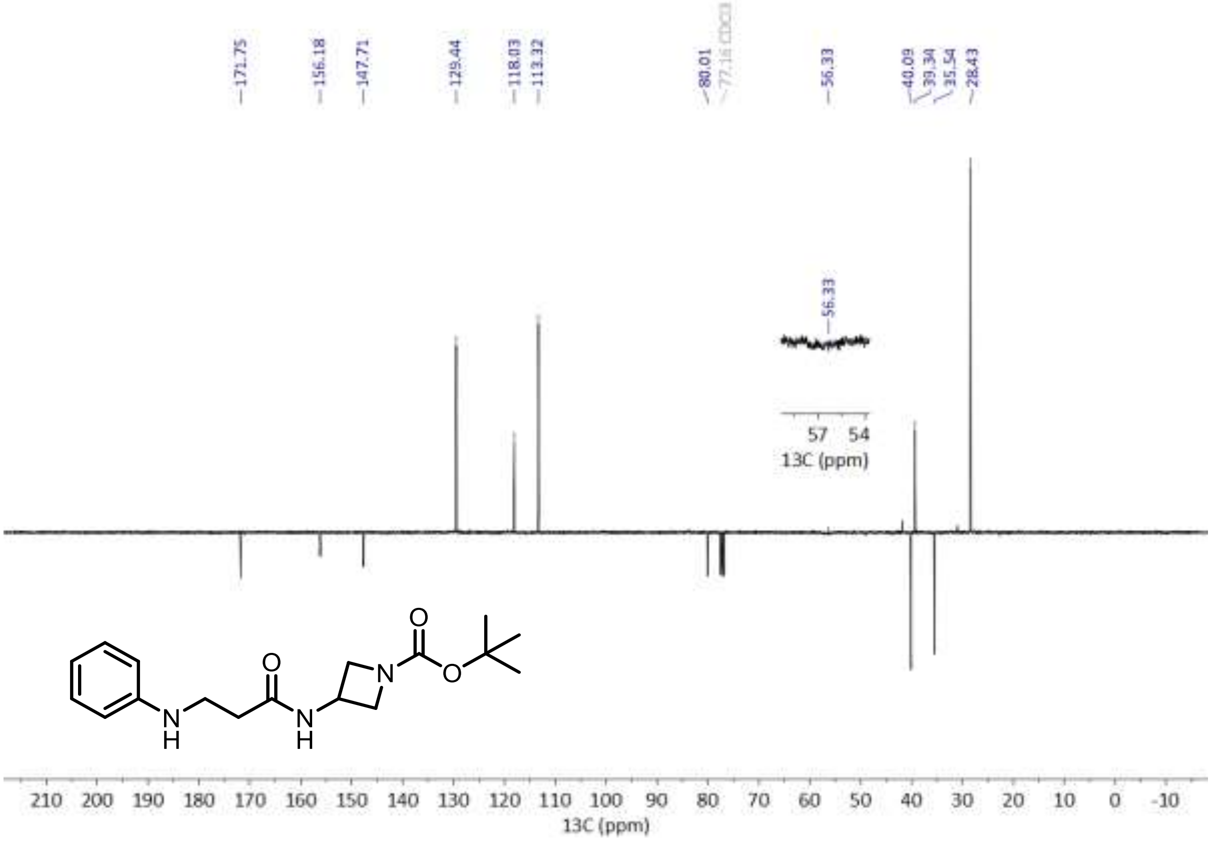

Compound 5i (1H)

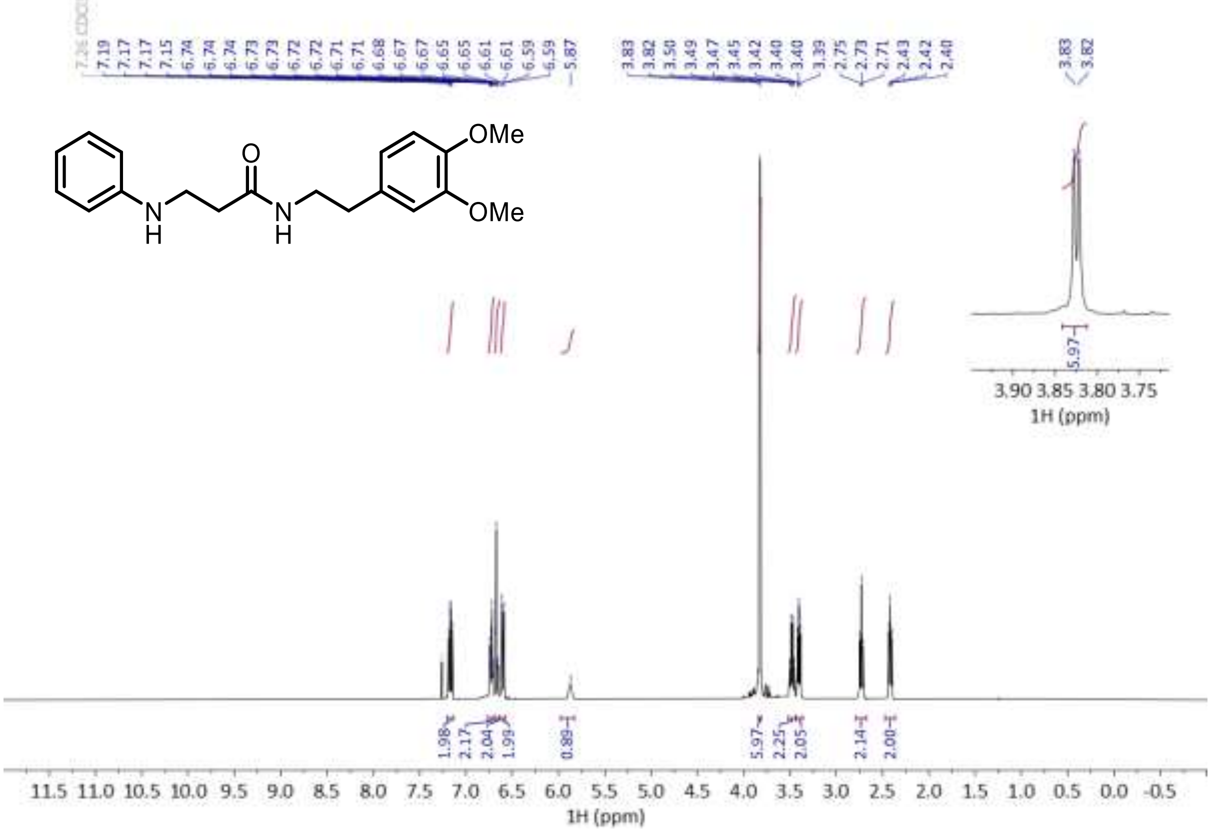

Compound 5i (13C)

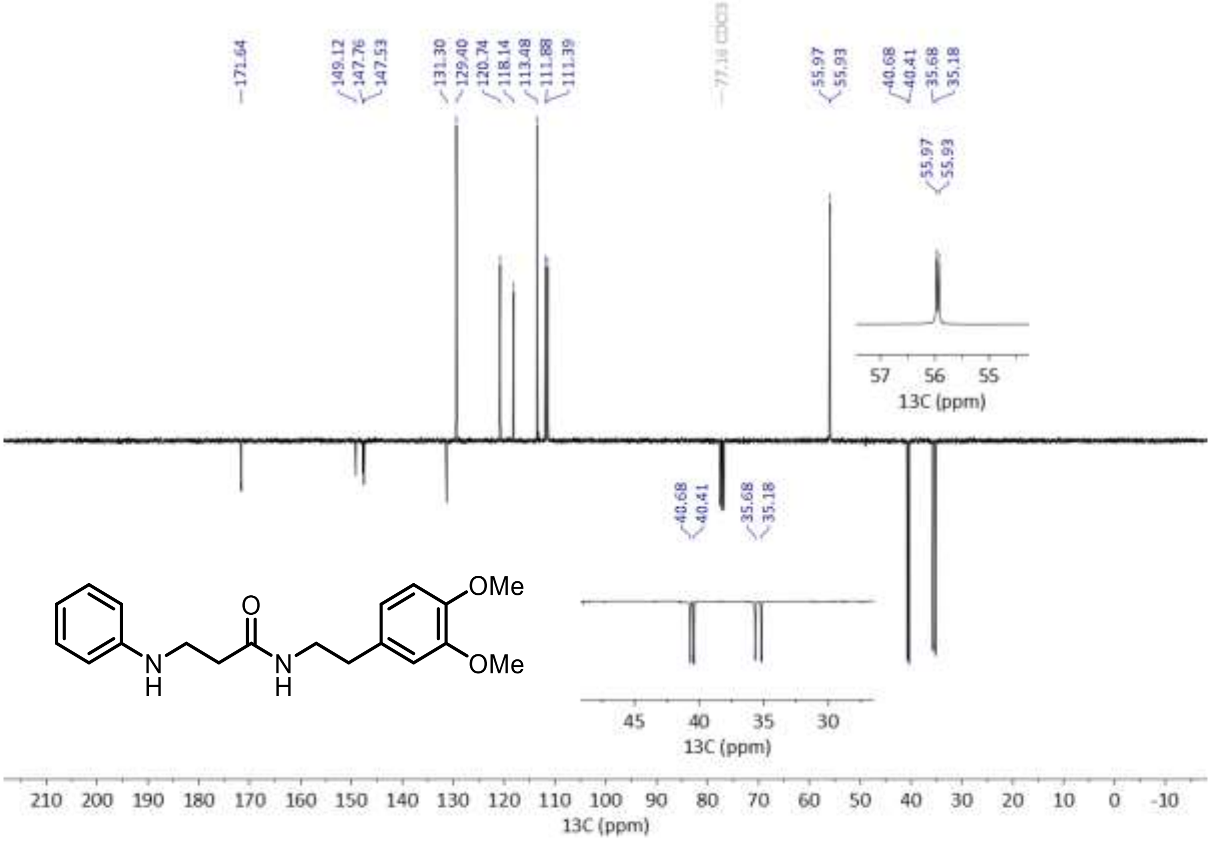

Compound S1-10 (^{1}H)

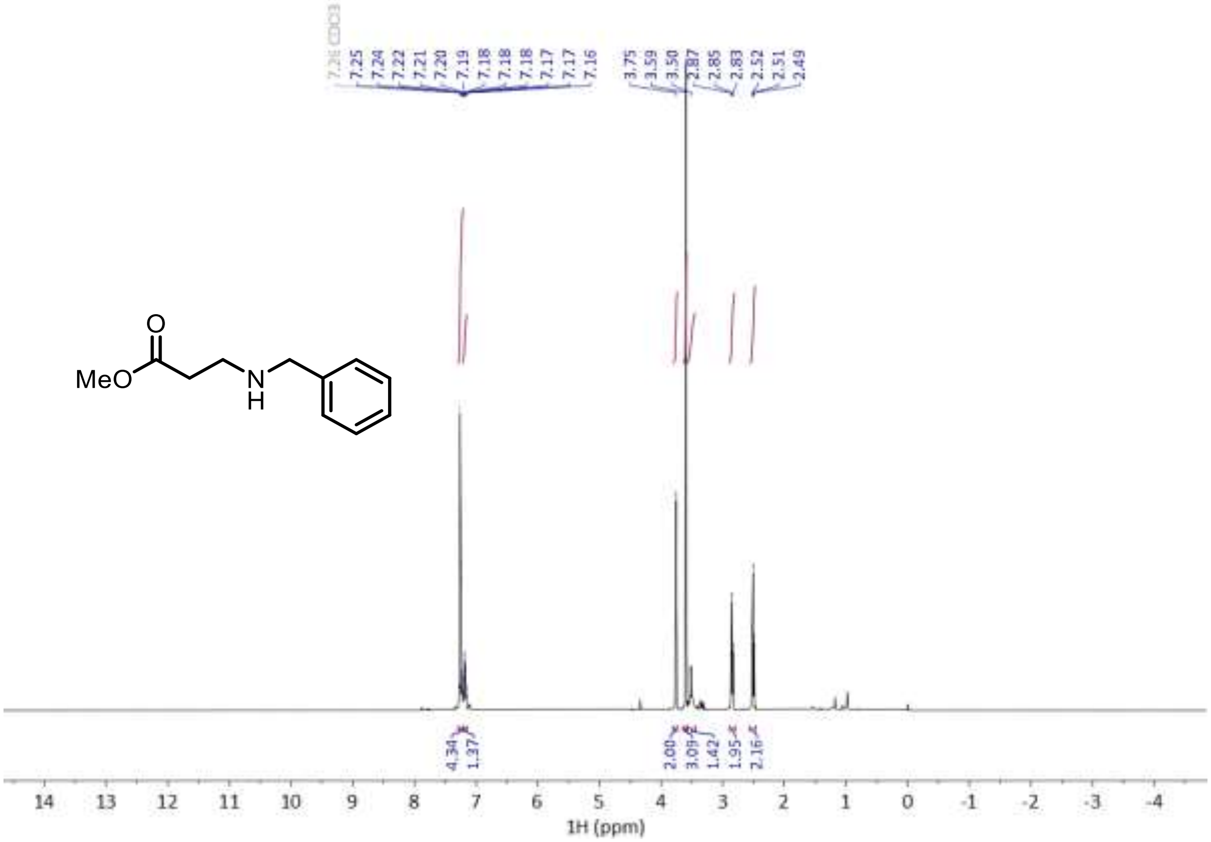

Compound S1-10 (^{13}C)

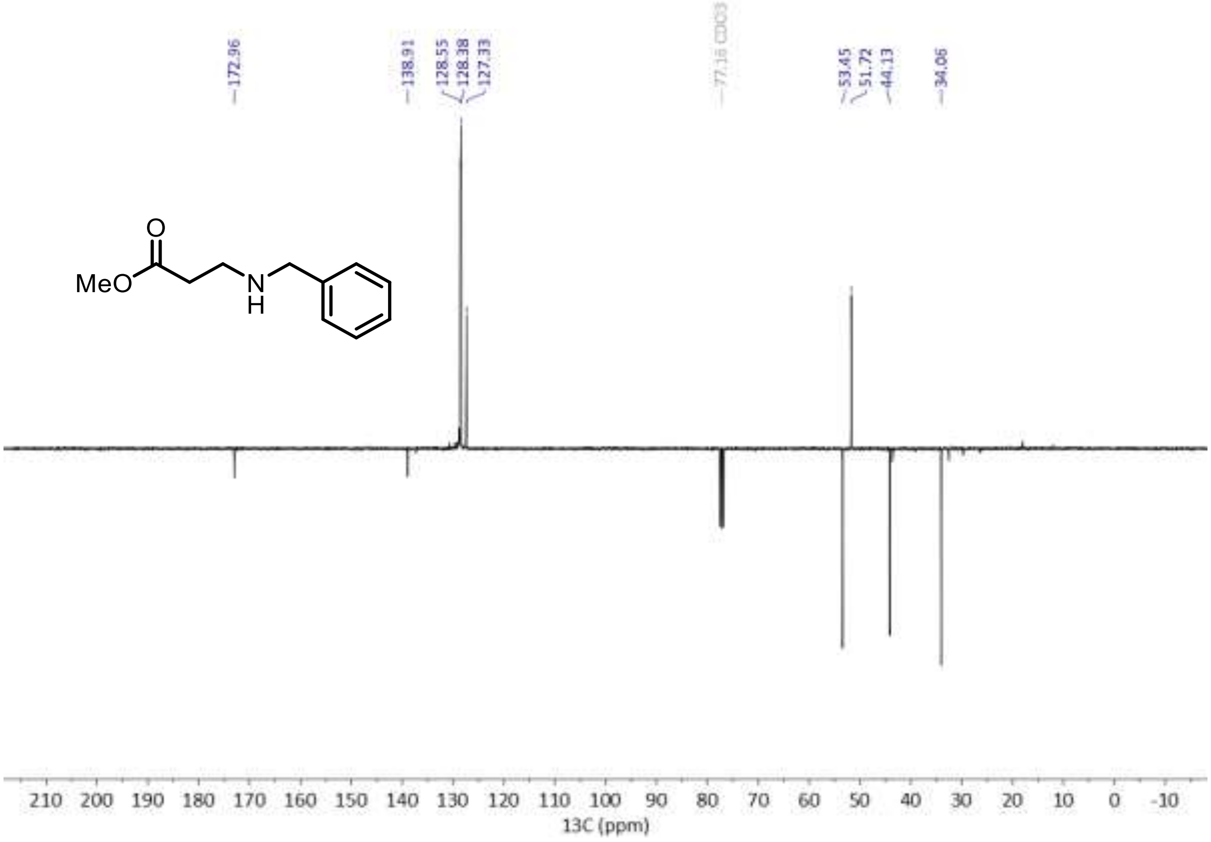

Compound 5l (^{1}H)

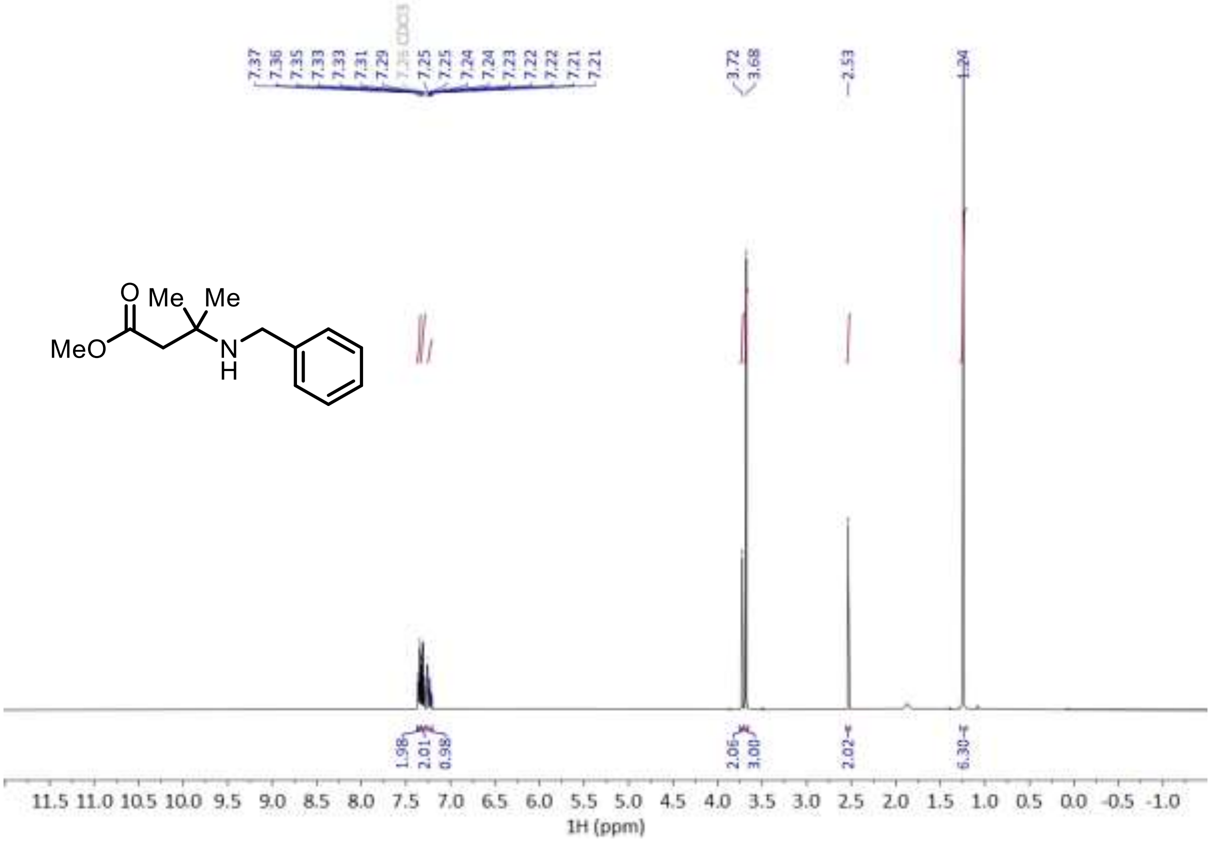

Compound 5l (^{13}C)

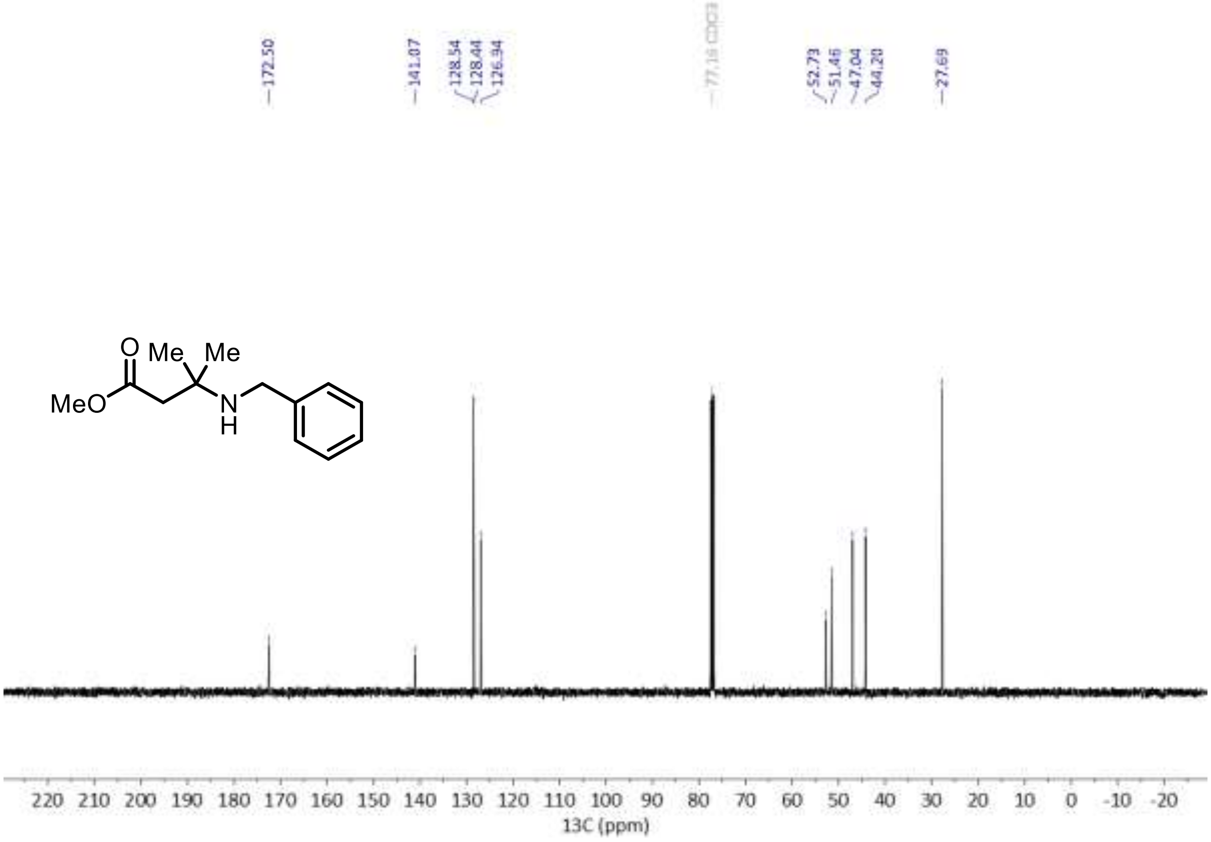

Compound 5m (^{1}H)

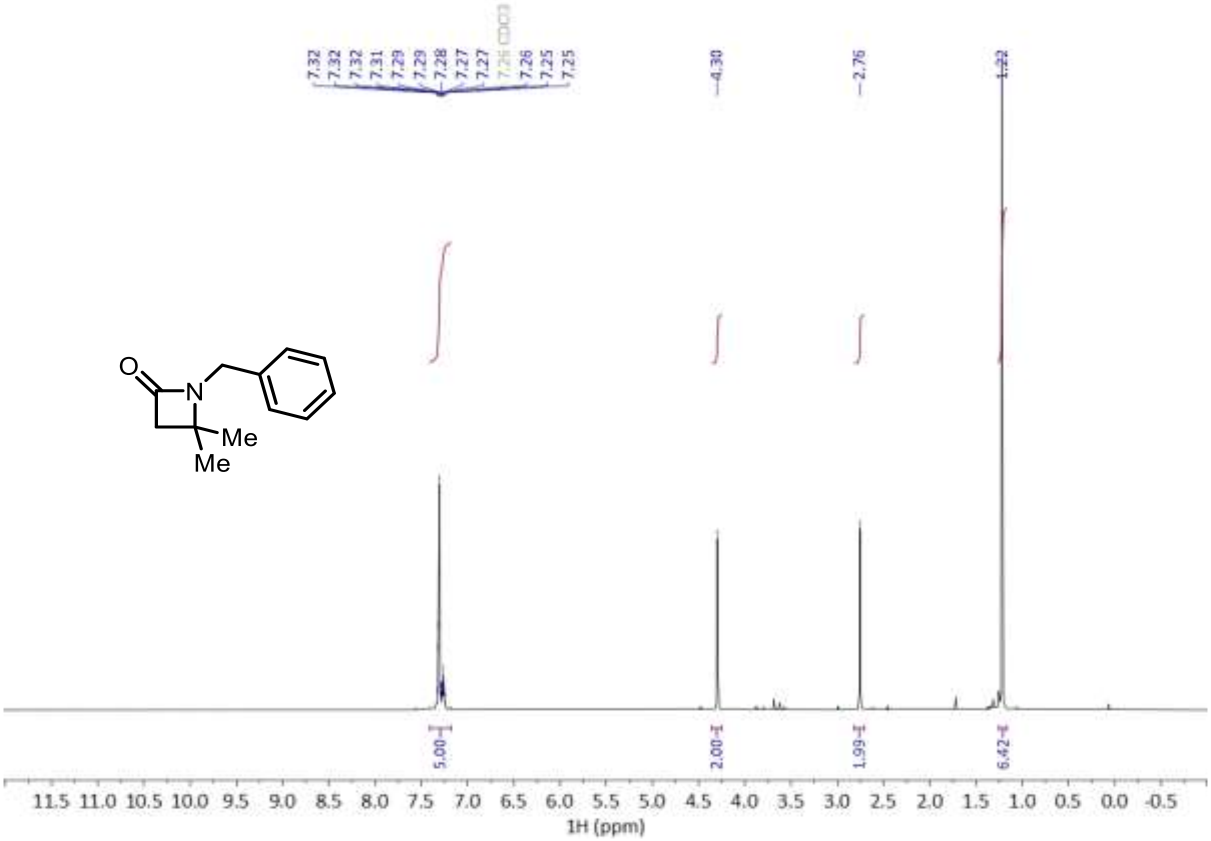

Compound 5m (^{13}C)

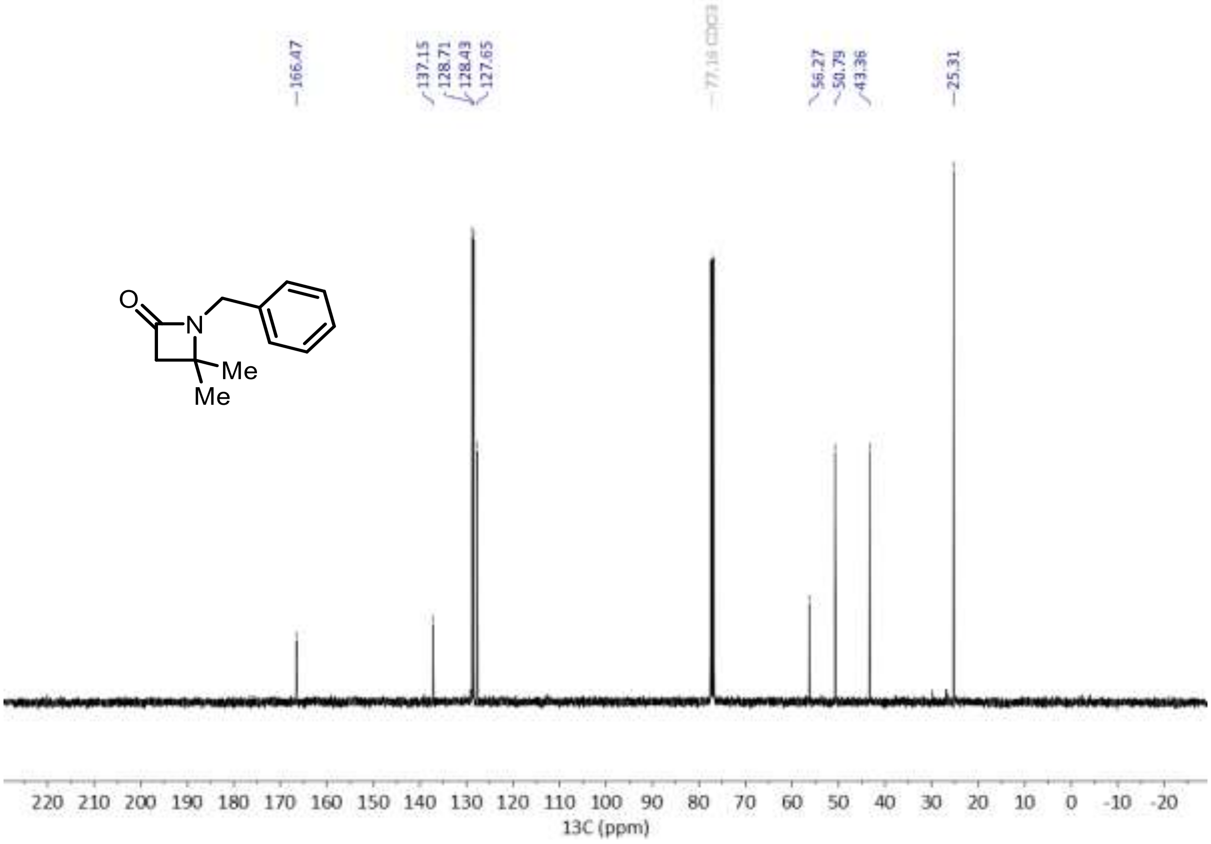

Supplementary References

1. Huo, H., Tang, X.-Y. & Gong, Y. Metal-Free Synthesis of Pyrrolo[1,2-a]quinoxalines Mediated by TEMPO Oxoammonium Salts. *Synthesis* **50**, 2727–2740 (2018).

2. Horn, A. & Kazmaier, U. Purified mCPBA, a Useful Reagent for the Oxidation of Aldehydes. *Eur. J. Org. Chem.* **2018**, 2531–2536 (2018).

3. Biallas, P., Yamazaki, K. & Dixon, D. J. Difluoroalkylation of Tertiary Amides and Lactams by an Iridium-Catalyzed Reductive Reformatsky Reaction. *Org. Lett.* **24**, 2002–2007 (2022).

4. Shennan, B. D. A., Sánchez-Alonso, S., Rossini, G. & Dixon, D. J. 1,2-Redox Transpositions of Tertiary Amides. *J. Am. Chem. Soc.* **145**, 21745-21751 (2023).

5. Matheau-Raven, D. & Dixon, D. J. General α-Amino 1,3,4-Oxadiazole Synthesis via Late-Stage Reductive Functionalization of Tertiary Amides and Lactams. *Angew. Chem. Int. Ed.* **60**, 19725–19729 (2021).

6. Gabriel, P., Gregory, A. W. & Dixon, D. J. Iridium-Catalyzed Aza-Spirocyclization of Indole-Tethered Amides: An Interrupted Pictet–Spengler Reaction. *Org. Lett.* **21**, 6658–6662 (2019).

7. Xue, Y. & Wang, S. Generation of Carbonyl Compounds from Oximes through Electrooxidative Deoximation. *J. Org. Chem.* **89**, 4199–4204 (2024).

8. Zhang, R., Wen, K. & Dong, G. Downsizing lactams via Rh-catalyzed C–C activation. *Chem* **11**, 102622 (2025).

9. Witosińska, A., Musielak, B., Serda, P., Owińska, M. & Rys, B. Conformation of Eight-Membered Benzoannulated Lactams by Combined NMR and DFT Studies. *J. Org. Chem.* 77, 9784–9794 (2012).

10. Deng, W. *et al.* Mild and Efficient CuI Catalyzed Coupling Reactions of Amides with Bromides. *Chin. J. Chem.* **23**, 1241–1246 (2005).

11. Peschiulli, A. *et al.* Ruthenium-Catalyzed α-(Hetero)Arylation of Saturated Cyclic Amines: Reaction Scope and Mechanism. *Chem. Eur. J.* **19**, 10378–10387 (2013).

12. Lu, Y. *et al.* Isolation and Reactions of Imidoyl Fluorides Generated from Oxime Using the Diethylaminosulfur Trifluoride/Tetrahydrofuran (DAST–THF) System. *Org. Lett.* **25**, 3482–3486 (2023).

13. Augustine, J. K., Kumar, R., Bombrun, A. & Mandal, A. B. An efficient catalytic method for the Beckmann rearrangement of ketoximes to amides and aldoximes to nitriles mediated by propylphosphonic anhydride (T3P®). *Tetrahedron Lett.* **52**, 1074–1077 (2011).

14. Desai, P. *et al.* Reactions of Alkyl Azides and Ketones as Mediated by Lewis Acids: Schmidt and Mannich Reactions Using Azide Precursors. *J. Am. Chem. Soc.* **122**, 7226–7232 (2000).

15. Sánchez-Bento, R., Bui, L., Duong, V. K., Ruffoni, A. & Leonori, D. A Photochemical Strategy for the Synthesis of Caprolactams via Dearomative Ring Expansion of Nitroarenes. *Synthesis* **56**, 2385-2391 (2024).

16. Winnacker, M., Tischner, A., Neumeier, M. & Rieger, B. New insights into synthesis and oligomerization of ε-lactams derived from the terpenoid ketone (−)-menthone. *RSC Adv.* **5**, 77699–77705 (2015).

17. Chmielewska, E. *et al.* Reaction of benzolactams with triethyl phosphite prompted by phosphoryl chloride affords benzoannulated monophosphonates instead of expected bisphoshonates. *J. Organomet. Chem.* **785**, 84–91 (2015).

18. Milligan, G. L., Mossman, C. J. & Aube, J. Intramolecular Schmidt Reactions of Alkyl Azides with Ketones: Scope and Stereochemical Studies. *J. Am. Chem. Soc.* **117**, 10449–10459 (1995).

19. Zeng, H., Yin, R., Zhao, Y., Ma, J.-A. & Wu, J. Modular alkene synthesis from carboxylic acids, alcohols and alkanes via integrated photocatalysis. *Nat. Chem.* **16**, 1822–1830 (2024).

20. Paraskar, A. S. & Sudalai, A. Co-catalyzed reductive cyclization of azido and cyano substituted α,β-unsaturated esters with NaBH4: enantioselective synthesis of (*R*)-baclofen and (*R*)-rolipram. *Tetrahedron* **62**, 4907–4916 (2006).

21. Zhang, Z. *et al.* Asymmetric Total Syntheses of Rhynchophylline and Isorhynchophylline. *J. Org. Chem.* **84**, 11359–11365 (2019).

22. Galvin, D. J. & Guiry, P. J. Enantioselective Synthesis of Sterically Hindered α-Allyl-α-Aryl Lactams via Palladium-Catalysed Decarboxylative Asymmetric Allylic Alkylation. *Eur. J. Org. Chem.* **27**, e202400314 (2024).

23. Miaskiewicz, S. *et al.* Gold(I)-Catalyzed N-Desulfonylative Amination versus N-to-O 1,5-Sulfonyl Migration: A Versatile Approach to 1-Azabicycloalkanes. *Angew. Chem. Int. Ed.* **55**, 9088–9092 (2016).

24. Nakagawa, Y., Chanthamath, S., Liang, Y., Shibatomi, K. & Iwasa, S. Regio- and Enantioselective Intramolecular Amide Carbene Insertion into Primary C–H Bonds Using Ru(II)-Pheox Catalyst. *J. Org. Chem.* **84**, 2607–2618 (2019).

25. Osorio-Nieto, U., Chamorro-Arenas, D., Quintero, L., Höpfl, H. & Sartillo-Piscil, F. Transition Metal-Free Selective Double sp3 C–H Oxidation of Cyclic Amines to 3-Alkoxyamine Lactams. *J. Org. Chem.* **81**, 8625–8632 (2016).

26. Nolasco-Hernández, Á. A., Quintero, L., Cruz-Gregorio, S. & Sartillo-Piscil, F. β-Alkenylation of Saturated N-Heterocycles via a C(sp3)–O Bond Wittig-like Olefination. *J. Org. Chem.* **89**, 1762–1768 (2024).

27. Bell-Tyrer, J. J., Hume, P. A., Grant, P. S., Brimble, M. A. & Furkert, D. P. Azide–Enolate Cycloaddition-Rearrangement Enables Direct α-Amination of Amides and Enelactam Synthesis from Esters. *Chem. Eur. J.* **29**, e202300261 (2023).

28. Griffiths, R. J., Burley, G. A. & Talbot, E. P. A. Transition-Metal-Free Amine Oxidation: A Chemoselective Strategy for the Late-Stage Formation of Lactams. *Org. Lett.* **19**, 870–873 (2017).

29. Ramachandran, P. V. & Choudhary, S. One-Pot, Tandem Reductive Amination/Alkylation–Cycloamidation for Lactam Synthesis from Keto or Amino Acids. *J. Org. Chem.* **88**, 15956–15963 (2023).

30. Knight, N. M. L. *et al.* Iridium-Catalysed C(sp3)−H Activation and Hydrogen Isotope Exchange via Nitrogen-Based Carbonyl Directing Groups. *Adv. Synth. Catal.* **366**, 2577–2586 (2024).

31. Prajapati, N., Giridhar, R., Sinha, A., Kanhed, A. M. & Yadav, M. R. Regioselective alkylation of 1,3,4,5-tetrahydrobenzo[d]azepin-2-one and biological evaluation of the resulting alkylated products as potentially selective 5-HT_{2C} agonists. *Mol. Divers.* **19**, 653–667 (2015).

32. Wang, W., *et al.* 3-Silaazetidine: An Unexplored yet Versatile Organosilane Species for Ring Expansion toward Silaazacycles, *J. Am. Chem. Soc.* **143**, 11141-11151 (2021).

33. Chowdhury, S., Chauhan, G., Kumar, A., Chaturvedi, B. & Behera, C. Copper-Mediated Intramolecular Amidation/C−N-Coupling Cascade Sequence: Straightforward One-Pot Synthesis of N-Aryl γ- and δ-Lactams by Using Amino Acids as Precursors. *Eur. J. Org. Chem.* **2022**, e202200850 (2022).

34. Wang, H. *et al.* Catalytic Cyanation Using CO2 and NH3. *Chem* **4**, 2883–2893 (2018).

35. Guazzelli, G., Duffy, L. A. & Procter, D. J. A Samarium(II)-Mediated, Stereoselective Cyclization for the Synthesis of Azaspirocycles. *Org. Lett.* **10**, 4291–4294 (2008).

36. Chen, J., Wang, J. & Tu, T. Ruthenium-Pincer-Catalyzed Hydrogenation of Lactams to Amino Alcohols. *Chem. Asian J.* **13**, 2559–2565 (2018).

37. Shen, Q. & Hartwig, J. F. Lewis Acid Acceleration of C−N Bond-Forming Reductive Elimination from Heteroarylpalladium Complexes and Catalytic Amidation of Heteroaryl Bromides. *J. Am. Chem. Soc.* **129**, 7734–7735 (2007).

38. Mantel, M. L. H., Lindhardt, A. T., Lupp, D. & Skrydstrup, T. Pd-Catalyzed C□N Bond Formation with Heteroaromatic Tosylates. *Chem. Eur. J.* **16**, 5437–5442 (2010).

39. Kutama, I. U. & Jones, S. Enantioselective Desymmetrization of Glutarimides Catalyzed by Oxazaborolidines Derived from cis-1-Amino-indan-2-ol. *J. Org. Chem.* **80**, 11468–11479 (2015).

40. Katritzky, A. R., Mehta, S., He, H.-Y. & Cui, X. Preparation of 1,5-Disubstituted Pyrrolidin-2-ones. *J. Org. Chem.* **65**, 4364–4369 (2000).

41. Rozsar, D. *et al.* Bifunctional Iminophosphorane-Catalyzed Enantioselective Nitroalkane Addition to Unactivated α,β-Unsaturated Esters. *Angew. Chem. Int. Ed.* **62**, e202303391 (2023).

42. Chen, M., Liu, F. & Dong, G. Direct Palladium-Catalyzed β-Arylation of Lactams. *Angew. Chem. Int. Ed.* **57**, 3815–3819 (2018).

43. Motiwala, H. F., Charaschanya, M., Day, V. W. & Aubé, J. Remodeling and Enhancing Schmidt Reaction Pathways in Hexafluoroisopropanol. *J. Org. Chem.* **81**, 1593–1609 (2016).

44. Frankowski, K. J., Liu, R., Milligan, G. L., Moeller, K. D. & Aubé, J. Practical Electrochemical Anodic Oxidation of Polycyclic Lactams for Late Stage Functionalization. *Angew. Chem. Int. Ed.* **54**, 10555–10558 (2015).

45. Tonogaki, K., Itami, K. & Yoshida, J. Catalytic Four-Component Assembly Based on Allenylboronate Platform: New Access to Privileged Allylic Amine Structures. *J. Am. Chem. Soc.* **128**, 1464–1465 (2006).

46. Steunenberg, P. *et al.* Lipase-Catalyzed Aza-Michael Reaction on Acrylate Derivatives. *J. Org. Chem.* **78**, 3802–3813 (2013).

47. Davies, S. G. *et al.* Asymmetric Synthesis of *syn*- and *anti*-α-deuterio-β^3-phenylalanine derivatives. *Tetrahedron: Asymmetry* **22**, 1035–1050 (2011).

48. Grohmann, M., Buck, S., Schäffler, L. & Maas, G. Diruthenium(I,I) Catalysts for the Formation of β- and γ-Lactams via Carbenoid C-H Insertion of α-Diazoacetamides. *Adv. Synth. Catal.* **348**, 2203–2211 (2006).
